# Enhancing Congruence between Implicit Motives and Explicit Goal Commitments: Results of a Randomized Controlled Trial

**DOI:** 10.3389/fpsyg.2017.01540

**Published:** 2017-09-12

**Authors:** Ramona M. Roch, Andreas G. Rösch, Oliver C. Schultheiss

**Affiliations:** Department of Psychology, Friedrich-Alexander University Erlangen-Nürnberg (FAU) Erlangen, Germany

**Keywords:** implicit motives, explicit motives, well-being, depression, motivational congruence

## Abstract

**Objective:** Theory and research suggest that the pursuit of personal goals that do not fit a person's affect-based implicit motives results in impaired emotional well-being, including increased symptoms of depression. The aim of this study was to evaluate an intervention designed to enhance motive-goal congruence and study its impact on well-being.

**Method:** Seventy-four German students (mean age = 22.91, *SD* = 3.68; 64.9% female) without current psychopathology, randomly allocated to three groups: motivational feedback (FB; *n* = 25; participants learned about the fit between their implicit motives and explicit goals), FB + congruence-enhancement training (CET; *n* = 22; participants also engaged in exercises to increase the fit between their implicit motives and goals), and a no-intervention control group (*n* = 27), were administered measures of implicit motives, personal goal commitments, happiness, depressive symptoms, and life satisfaction 3 weeks before (T1) and 6 weeks after (T2) treatment.

**Results:** On two types of congruence measures derived from motive and goal assessments, treated participants showed increases in agentic (power and achievement) congruence, with improvements being most consistent in the FB+CET group. Treated participants also showed a trend-level depressive symptom reduction, but no changes on other well-being measures. Although increases in overall and agentic motivational congruence were associated with increases in affective well-being, treatment-based reduction of depressive symptoms was not mediated by treatment-based agentic congruence changes.

**Conclusion:** These findings document that motivational congruence can be effectively enhanced, that changes in motivational congruence are associated with changes in affective well-being, and they suggest that individuals' implicit motives should be considered when personal goals are discussed in the therapeutic process.

## Introduction

Research conducted over the past two decades supports the notion that implicit motives, that is, dispositional capacities for experiencing certain classes of incentives as pleasurable and certain classes of disincentives as aversive, represent an important source of emotional well-being (Brunstein, 2010; Schultheiss and Köllner, in press). Specifically, a growing number of studies show that emotional well-being is high in individuals who pursue and realize personal goals that fit their implicit motives (i.e., that are *congruent* with them), whereas emotional well-being is impaired in those who fail to realize such goals or pursue goals that do not fit their motives (i.e., that are *incongruent* with them; e.g., (Brunstein et al., 1998); for recent reviews, see Brunstein, 2010; Thrash et al., 2012; Hofer and Busch, 2013; Schultheiss and Köllner, 2014). Recent research has extended these findings to the domain of depressive symptoms (Schultheiss et al., 2008; Pueschel et al., 2011) and linked implicit motives to clinical depression (Neumann and Schultheiss, 2015).

From a motivational perspective, emotional ill-being could easily be reduced if (a) people had insight into their implicit motives and (b) consequently chose or developed motive-congruent personal goals. But neither is the case. Independence between peoples' implicit motives and the goals and values they explicitly ascribe to themselves has been observed with great consistency by McClelland and colleagues since the 1950's (e.g., deCharms et al., 1955; McClelland, 1980; McClelland et al., 1989; Weinberger and McClelland, 1990), and a recent meta-analysis corroborates this observation: The beliefs that people explicitly hold about their motivational needs have virtually no statistical overlap with their implicit motives (Köllner and Schultheiss, 2014; see also Spangler, 1992). Moreover, a large-scale study with over 300 participants demonstrated that the goals that people set for themselves and that they identify with in their daily lives likewise do not converge with their implicit motives, but instead with their explicit, self-attributed motives (Rawolle et al., 2013; see also King, 1995), which appear to influence personal goal choices (see Hofer et al., 2010). In the present paper, we therefore describe an intervention that aims at making individuals aware of the (lack of) congruence between their personal goals and their implicit motives, provides them with tools to increase motivational congruence, and examines the impact of these interventions longitudinally on changes in motivational congruence and affective and cognitive well-being.

## Implicit motives, personal goals, and emotional well-being

For the past 60 years, implicit motive research has focused on three motivational needs: the need for power (often abbreviated as n Power), a capacity for deriving pleasure from having impact on others; the need for achievement (n Achievement), a capacity for enjoying the autonomous mastery of challenging tasks; and the need for affiliation (n Affiliation), a capacity for cherishing close, harmonious relationships (McClelland, 1987; Schultheiss and Köllner, in press). These motivational needs are measured through thematic apperception, that is, by coding specific types of imagery in stories that research participants write about pictures with ambiguous social content (e.g., a captain talking to a passenger). Such motive measures are causally valid, because motivational arousal leads to increases in motive-specific imagery (McClelland, 1958, 1987; Borsboom et al., 2004), and their predictive validity has been broadly documented in studies targeting such diverse endpoints as endocrinology and physiology, illness and health, career success, and learning and memory (McClelland, 1987; Schultheiss and Köllner, in press). For intervals of up to several months they have moderate to high retest reliability, and they are characterized by high coding objectivity, as assessed by inter-rater reliability (Schultheiss and Pang, 2007).

In line with an early characterization of motives as affect amplifiers (Atkinson, 1957), research provides robust evidence that a stronger motive results in more positive affective responses to motive-specific incentives, but also in more negative affective responses to disincentives. For instance, n Affiliation predicts more negative facial affect in response to others' lack of friendliness (Kordik et al., 2012), but also more positive facial affect in response to others' positive affiliative signals (Dufner et al., 2015). n Power predicts more negative facial affect in response to others' dominant behavior (Fodor et al., 2006) and n Achievement predicts greater anticipated satisfaction for mastery of a challenging goal (Brunstein and Maier, 2005).

More importantly for the present study, research by Brunstein and colleagues (Brunstein et al., 1995, 1998; Schultheiss et al., 2008; Schultheiss, 2013, Study 2) not only provided strong evidence in support of the motives-as-affect-amplifiers hypothesis, but also clarified how the goals that people set for themselves promote or prevent emotional well-being generated by the satisfaction of implicit motives. Summarizing research from longitudinal and cross-sectional studies, Brunstein (2010) describes the interplay between motives and goals as follows: Personal goals select the life domains into which people channel time and effort. If the goals people commit themselves to are congruent with their implicit motivational needs and thus provide opportunities for their gratification, then variations in goal progress are strongly predictive of variations in emotional well-being. This is because in line with the affect amplifier hypothesis, each successful step of goal implementation represents a motivationally rewarding experience and each failure is aversive. Although at first blush this may suggest that congruent goals do not result in more net emotional well-being if one is as likely to experience pleasure and pain alike, Schultheiss and Köllner (2014) have postulated the existence of a fundamental asymmetry between success and failure in the pursuit of motive-congruent goals, arguing that due to the skill-building properties of motives the former is more likely to occur than the latter.

In contrast, if the goals people commit to are incongruent with their implicit motivational needs, then emotional well-being is independent of variations in goal progress, because such goals do not provide opportunities for motive satisfaction; that is, successes and failures en route to goal implementation do not impinge on relevant motivational needs. However, because goals affect the time and effort people spend in motivationally relevant or irrelevant life domains, the pursuit of incongruent goals can also lead to impaired emotional well-being, including increased symptoms of depression, because it hampers the realization of goals in motivationally congruent domains and thus effectively leads to motivational frustration (see Brunstein et al., 1998). This conclusion is echoed in Michalak and Grosse Holtforth's (2006; see also Conrad et al., 2010; Hauke, 2010) discussion of the significance of Brunstein's (2010) model for the etiology of mental health problems and their treatment:

“[…] if a person strives for goals that have a low degree of fit with his or her personality, this might also have unavoidable consequences for his or her mental health because attaining these goals will probably not lead to the satisfaction of fundamental psychological needs. Personal goals and their characteristics can thus assume the role of a *pathogenic factor* that influences the development and maintenance of mental disorders.” (p. 350; italics in original).

To summarize, the pursuit of motive-congruent goals has been characterized as beneficial for emotional well-being, because it allows people to encounter motivationally relevant rewards more often than punishment. In contrast, the pursuit of motive-incongruent goals has an indirect negative effect on emotional well-being, because it prevents people from satisfying their implicit motives by engaging in motivationally congruent goal pursuit.

## How can motivational congruence be enhanced?

Previous research has examined some of the factors that influence motivationally congruent goal choices in the laboratory (e.g., Schultheiss and Brunstein, 1999; Job and Brandstätter, 2009; Rawolle et al., 2016; Strick and Papies, 2017) or has attempted to increase implicit motives through training directly (e.g., McClelland and Winter, 1971; deCharms, 1976). But so far no study has addressed the issue of how the personal goals that people pursue in their daily lives and that structure much of their everyday activities and experiences (Brunstein, 1993; Emmons, 1996) can be made more motivationally congruent.

If impaired emotional well-being is the consequence of the pursuit of motivationally incongruent goals, and the choice of such goals is in turn the result of a lack of valid introspective insight into one's motivational needs, then a sensible first step toward helping people pursue more congruent goals is to educate them individually about their motives. This is the approach we took in the present research. We provided participants with feedback about their motives in the domains of power, achievement, and affiliation, based on the stories they wrote on a standard picture-story exercise (PSE) measure of motivational needs. Because this feedback only told participants whether they were high or low in a given motive, but little about how it played out in their daily lives, we also asked them to remember peak experiences from their lives. The content of such experiences reflects individuals' dominant motivational needs (McAdams, 1982; Woike, 2008) and helps people get a better idea of how, for instance, the satisfaction of n Power plays out in their actual lives and affects their behavior and emotional experience.

Learning to recognize and understand one's implicit motivational needs may be a necessary, but perhaps not a sufficient prerequisite for committing to motivationally congruent goals. For this reason, we added a congruence-enhancement training (CET) module to our educational intervention for one group of participants that was directly aimed at reducing motivational incongruence for all three motive domains (achievement, power, affiliation). Incongruence can be the result of either a strong implicit motive, but low or absent goal commitments in a given domain, or a weak motive, combined with a strong commitment to a goal. In the former case, congruence can be enhanced by increasing one's subjective commitment to a suitable goal, promoted by becoming more mindful of the ways in which goal attainment will be pleasurable. In the latter case, because reducing goal commitments or giving up a goal may be neither feasible nor ethically advisable in the context of a non-clinical study, we opted for an enhancement strategy that aimed at helping people think of ways in which strong motives from other domains could be tied to the pursuit of an incongruent goal in a given domain and thus to activate existing resources. This approach is based on Schultheiss et al.'s (2008, p. 985) conclusion that even though personal goals may be identified with a focal motivational need, they may also contain additional incentives for other motivational needs. Finally, for those domains in which high congruence resulted from a strong motive paired with a strong goal commitment, participants were asked to elaborate on which aspects of goal implementation would be particularly likely to satisfy their motivational needs. For congruence based on weak goal commitments coupled to a weak motive, participants were instructed to be mindful of the possibility that future goals in this motivational domain could potentially be furnished with a strong commitment and thus lead to incongruence.

Thus, our approach toward enhancement of motivational congruence was two-pronged: First, create awareness of one's implicit motivational needs and the degree to which they fit one's goal commitments. Second, increase congruence where it is low and bolster it where it is already high. Enhancing motivational congruence in this manner was the primary goal of this study and we hypothesized that this combined approach would be particularly effective at increasing individuals' motivational congruence (*Hypothesis 1*). We also expected this to have a beneficial effect on well-being (*Hypothesis 2*) as measured via self-reported happiness and depressive symptoms (tapping the affective component of well-being) as well as satisfaction with life (tapping the cognitive component of well-being; see Andrews and McKennell, 1980) at follow-up. We also tested whether increases in motivational congruence mediated treatment effects on measures of well-being (*Hypothesis 3*). We expected our treatment effects to hold equally across the three motivational domains power, achievement, and affiliation.

## Overview of the present research

We tested our hypotheses in a longitudinal study with a net sample of 74 undergraduate and graduate students. One third participated in a no-treatment CG, one third received feedback about their implicit motives and how well they fit their personal goal commitments (FB), and one third received CET in addition to feedback (FB+CET). Participants' implicit motives, personal goal commitments, the degrees of congruence between both within all given motivational domains, as well as their current happiness, depressive symptoms, and life satisfaction were assessed at the beginning and at follow-up 9 weeks later. Within the first 3 weeks, participants in the training groups received FB or FB+CET. In testing our hypotheses, we focused on treatment-elicited changes in motivational congruence and well-being as well as the association between these changes.

## Method

### Sample

**Figure 1:**
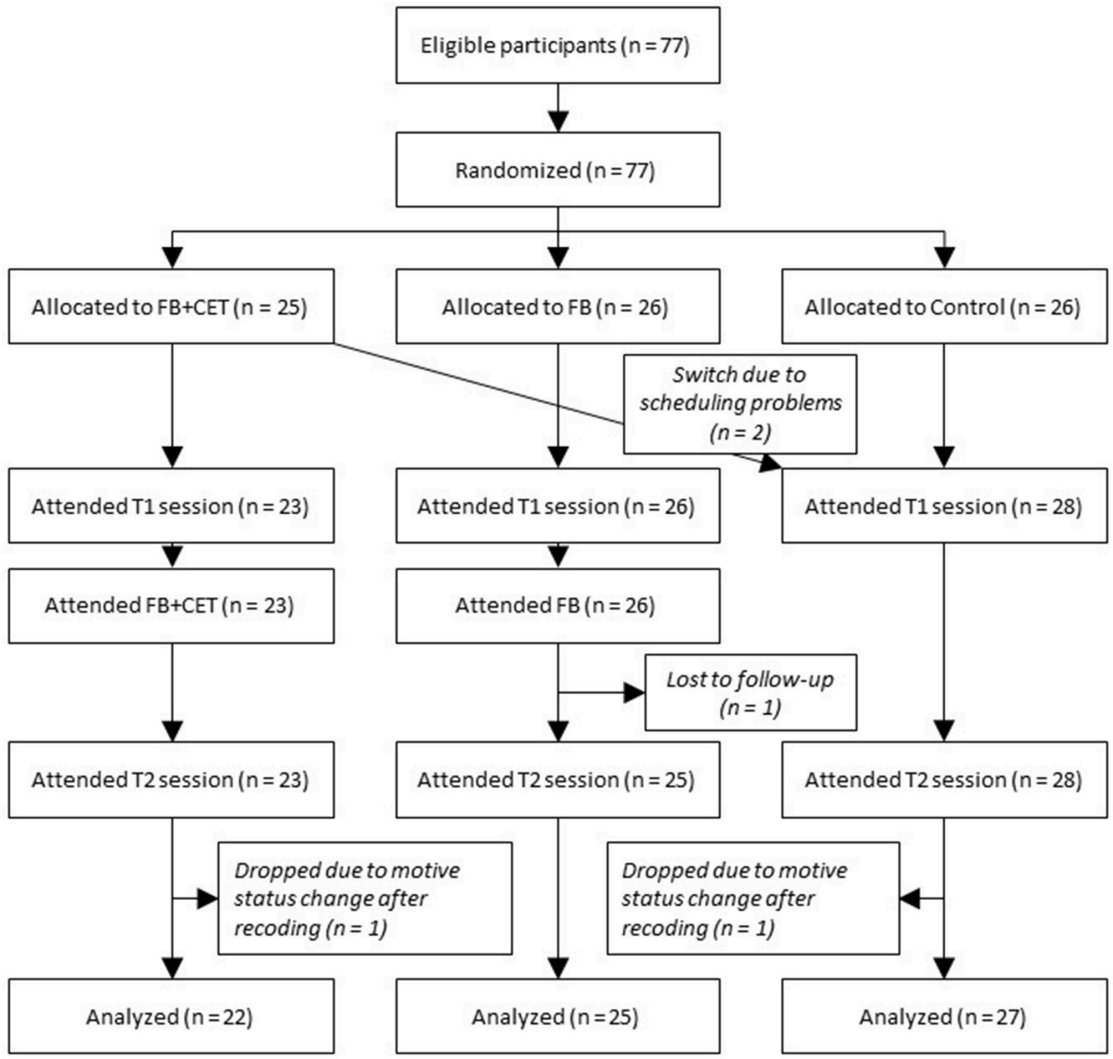
Consolidated Standards of Reporting Trials (CONSORT) diagram illustrating flow of participants through the study. FB, motivational feedback; FB+CET, motivational feedback plus congruence-enhancement training.

A CONSORT diagram (Schulz et al., 2010) illustrating participant flow throughout the study is presented in Figure 1. Seventy-seven individuals (most of them students at Friedrich-Alexander University, Erlangen, Germany; psychology students were excluded) were either recruited actively by approaching individuals on the premises of the university (e.g., in the cafeteria) or they responded via email to fliers posted on-campus and requested to participate in this study over the course of the 2008 summer semester. Participants were offered payment of 25€ and an individually tailored motivational feedback. Sample size was due to, and limited by, the requirement for completing a diploma thesis at Friedrich-Alexander University within 6 months, which includes devising a research project, recruiting and testing participants, coding, entering, and analyzing the data, and writing the thesis. Participants were randomly allocated to a control group (CG; *n* = 26) a feedback group (FB, *n* = 26) and a feedback with congruence-enhancement training group (FB+CET; *n* = 25). Due to scheduling problems, two participants assigned to the FB+CET condition had to be reassigned to the CG condition. One FB-group participant dropped out of the study after the first testing session. Two additional participants, one from the CG and one from the FB+CET condition, were excluded from further analysis because their motivational profiles changed after recoding of the picture-story exercise and the feedback and training they received was partially invalid as a consequence (see below). The remaining 74 participants (48 women and 26 men) had an average age of 22.91 years (*SD* = 3.68). All participants provided written informed consent prior to study commencement, were fully debriefed after completing the study, and were treated in accordance with the American Psychological Association's Ethics Code. At the time the study was conducted, neither Friedrich-Alexander University nor national regulations required ethics board approval for behavioral-science studies with healthy, consenting, adult populations. As a consequence, neither Friedrich-Alexander University nor the German Psychological Society had implemented an ethics review board for the behavioral sciences or required approval through such a board.

### Design

We used an experimental intervention approach, realized in a longitudinal study with three levels of the factor Condition: CG (*n* = 27), FB (*n* = 25), and FB+CET (*n* = 22). Dependent variables were changes in motivational congruence within the domains power, achievement, and affiliation, changes in mood, changes in life satisfaction, and changes in depressive symptoms, controlling for initial levels in these variables.

### Procedure

The study consisted of two measurement waves, Time 1 (T1) and Time 2 (T2), spaced 9 weeks apart and a training block within 2 weeks after T1. The timing reflected the need to code all participants' PSEs after the initial wave and to prepare individualized feedback materials, to then schedule and conduct all training sessions with all participants, and to allow for sufficient time for the expected training-induced changes to manifest themselves in participants' goal pursuits and subjective well-being. At T1 and T2, we assessed, in this sequence, implicit motives, personal goal commitments, happiness, depressive symptoms, and life satisfaction. Only individuals assigned to the FB and FB+CET conditions were invited to participate in the training, which was fully manualized (see Supplementary Material). CG participants received their motivational profiles and access to all FB+CET training materials, and FB participants received all CET training materials, after T2 assessments were completed.

### Treatment

Participants in the FB group attended one group session lasting 2.5 h during which they received basic information about implicit motives, explicit goals, and their conjoint effects on behavior and emotional well-being. This information was provided both in the form of a written booklet and presentations by and discussions with the instructor. The booklet also contained individualized feedback on the strength of each participant's implicit motives and personal goal commitments in the form of a motivational profile juxtaposing implicit motive and personal goal commitment strength, identified as high or low, separately for the domains power, achievement, and affiliation. The profile was accompanied by brief written explanations of the type and meaning of the (in-)congruence within each motivational domain. In addition, participants worked on a peak-experience recall task. They were asked to write down one peak experience each for the domains of power, achievement, and affiliation, including a description of what the event entailed and how they felt at the time. This exercise was designed to complement the normative information they received about the strength of their implicit motives with recalled autobiographical instances of each motive in action.

Participants in the FB+CET group attended one group session lasting 3.5 h that featured the same modules as the FB group, but additionally had participants think of ways in which they could increase motive-goal congruence in areas where a lack of congruence had been identified and elaborate on the ways in which a goal fits a given motive in domains where they had high levels of implicit motivation and personal goal commitment. One module aimed at reducing incongruence, termed “Discovering potentials,” targeted the case when a strong motive met a weak goal commitment in a given motivational domain. Participants were asked to recall the peak experience in the given domain listed previously and were instructed to think of ways in which this memory might help to bolster the corresponding goal (e.g., wanting to spend more time with a friend) with suitable incentives and thus make it more attractive (e.g., recalling an emotionally moving conversation with that friend).

Another module aimed at reducing incongruence, termed “Discovering opportunities for change,” targeted the case when a weak motive met a strong goal commitment in a given motivational domain. Here, participants were instructed to think of ways in which the goal could be enriched with incentives relevant for a strong motive in another domain (to continue the previous example, by spending more time with the friend by playing badminton to satisfy one's implicit need to achieve). But they were also encouraged to draw on the peak experience they had listed for this domain to identify potentially rewarding aspects of this goal. The latter strategy is based on the premise that a weak motive does not necessarily imply that the person cannot cherish the incentives associated with this motive, only that he or she responds to fewer of them (see Schultheiss and Schultheiss, 2014).

A third module aimed at increasing awareness of beneficial congruence, termed “Maximizing potentials,” targeted the case in which a person has high levels both in the motive and in the goal commitment domain. Participants were instructed to reflect on the unique experiential aspects of this combination. Specifically, they were asked to identify several specific actions aimed at goal attainment that fit their underlying motivational need particularly well and to anticipate the feelings likely to be generated by carrying out these actions. No module was devised for the case of low-motive/low-goal-commitment congruence.

Participants were asked to apply the three modules to each case in which their motivational profile featured a type of incongruence or beneficial congruence and to do this in writing in a training booklet they received. None of the participants had a low-motive/low-goal-commitment profile in all three motivational domains.

### Measures

#### Picture story exercise (PSE)

The PSE is a standard story-writing measure of implicit motives (Schultheiss and Pang, 2007). We used Pang and Schultheiss's (2005) computer-based version, with the pictures boxer, trapeze artists, women in laboratory, ship captain, nightclub scene, and couple by river (https://osf.io/6kfhz/). During both administrations, participants viewed each picture for 10 s and then had 4 min to write an imaginative story about the picture. Instructions at T2 were modified to allow participants to tell the same stories again or write different ones to avoid artificial lowering of retest reliability and score validity at T2 due to pressure to come up with new stories (see Schultheiss and Pang, 2007).

The content of the stories was later coded for achievement, power, and affiliation imagery according to Winter's (1991, 1994) Manual for Scoring Motive Imagery in Running Text. Winter's coding system integrates the needs for affiliation and intimacy into one overall affiliation category, because measures of n Intimacy, which emphasize the capacity for love more than harmonious relationships *per se*, show a large overlap with measures of n Affiliation (e.g., Hofer and Busch, 2011). A trained coder, who had exceeded 85% of interscorer agreement on expert-scored calibration materials before the study commenced, coded all PSEs. The coder was blind to participants' group allocation and scores on other measures and coded all T1 materials twice: In a first run, the coder had attained 91% intercoder agreement with German expert-coded PSE stories for n Achievement, 82% for n Affiliation, and 91% for n Power and aimed at providing motive scores based on T1 PSEs quickly enough to enable the provision of feedback and training for the FB and FB+CET conditions. In a second run, after the study was finished, the coder had undergone additional training and now reached 100% intercoder agreement for n Achievement, 92% for n Affiliation, and 100% for n Power and then (re)coded all PSE protocols for T1 and T2. Because two participants from the FB-CET group with motive scores at the classification border of low/high changed their motive score status as a result of recoding, the feedback that they had received with regard to their motivational congruence in one domain each was no longer valid and they were therefore excluded from all further analyses.

Motive raw scores at T1 (n Achievement: *M* = 4.80, *SD* = 2.33; n Power: *M* = 5.88, *SD* = 2.71; n Affiliation: *M* = 6.24, *SD* = 2.90) and at T2 (n Achievement: *M* = 5.03, *SD* = 2.58; n Power: *M* = 5.51, *SD* = 3.06; n Affiliation: *M* = 6.05, *SD* = 3.08) were comparable to previous studies using the same picture set (e.g., Schultheiss et al., 2011). Motive raw scores were significantly correlated with total PSE word count at T1 (*M* = 588, *SD* = 155; *r*_Achievement_ = 0.46, *r*_Power_ = 0.50, *r*_Affiliation_ = 0.41, *p*s < 0.0005) and T2 (*M* = 591, *SD* = 178; *r*_Achievement_ = 0.24, *r*_Power_ = 0.57, *r*_Affiliation_ = .49, *p*s < 0.05). We therefore corrected for word count by dividing the product of each total motive score and 1,000 by the total word count (Winter, 1994). These scores provided the basis of the motivational feedback and were used in subsequent statistical analyses. See Schultheiss and Pang (2007), for a discussion of these procedures.

#### Personal goals questionnaire (PGQ)

The PGQ by Brunstein et al. (1998; https://osf.io/zte8u/) provides an assessment of idiographic and nomothetic aspects of personal goals separately for each motivational domain. Participants first described in a couple of sentences one personal goal for each of the domains power, achievement, and affiliation that they intended to realize in the near future. Afterwards they rated their commitment to each goal on four items [“I fully identify with this goal”; “No matter what happens, I will not give up this goal”; “I can hardly wait to start working on this goal (again)”; “Even if it means a lot of effort, I will try everything necessary to accomplish this goal”]. Response scales ranged from 1 (*disagree strongly*) to 5 (*agree strongly*). Internal consistency of the commitment scale was satisfactory, with a Cronbach's alpha of 0.78 for achievement, 0.74 for affiliation, and 0.84 for power at T1 and 0.72 for achievement, 0.75 for affiliation, and 0.87 for power at T1. Scores were averaged across items for each motivational domain.

#### Derivation of congruence indices

Congruence indices were derived in the following manner: in keeping with the feedback participants received about their implicit motives on their motivational profiles, we classified them as high in a given domain if their motive score was above the sample median and otherwise as low. Similarly in keeping with the feedback they received about their goal commitment strength, we considered an average score of 4 or higher on the 5-point scale as evidence of high goal commitment and classified all scores below this cutoff as low goal commitment. This classification was primarily based on earlier research in our lab using the PGQ goal commitment scale, which had consistently suggested only values of 4 and up to indicate strong goal commitment as reflected in, for instance, substantial goal progress. It was also consistent with the goal commitment scale means and medians in each of the three motivational domains, whose values were at or slightly above 4. For each of the three domains, we derived a congruence score by assigning a value of 1 if a participant had been classified as high in the motive and high in goal commitment. However, a person can also be congruent if he or she has a very low goal commitment and a correspondingly low motive score. We conservatively deemed this to be the case whenever a participant had an average goal commitment score less than 4 and a word-count-corrected motive score lower than 2, which would roughly correspond to no more than 1 scored image on the entire PSE. There is evidence that motive scores this low on a standard PSE reflect not just an indifference to domain-specific incentives, but may indicate active avoidance (see Schultheiss and Köllner, in press). In this case, too, we assigned a score of 1. In all other cases, we assigned a score of 0, indicating low congruence.

We applied the same cutoffs and procedures for deriving congruence scores both at T1 and at T2 to ensure that the criteria used at T1 for classifying participants and providing them with feedback and training in the treatment groups would also apply when reassessing congruence at T2. Finally, we also calculated, separately for T1 and T2, a total congruence score by summing congruence indices across the three motivational domains. This score could range from 0 (no congruence at all) to 3 (congruence in all motivational domains).

#### Derivation of incongruence indices

Due to the pivotal role of motivational congruence assessment for determining the effectiveness of our interventions, we also included an alternative congruence measure. Indices of motivational *incongruence* have been frequently used in previous research to quantify the fit between people's motives and goal commitments (e.g., Schultheiss et al., 2011). Following procedures established in earlier research, we first converted participants' implicit motive scores, residualized for word count, and goal commitment scores to z scores. Next, we calculated absolute differences between motive and goal z scores within each domain. In a final step, we subjected these scores to a log transformation to ensure normal variable distributions. Thus, the algorithm for the latter two steps was: incongruence score = log (0.5 + |motive z score – goal commitment z score|). *Higher* scores on this measure indicate more motivational *incongruence*. Here, too, we calculated, separately for T1 and T2, a total incongruence score by summing incongruence indices across the three motivational domains. Thus, in contrast to congruence scores, which were based on fixed cutoff scores, featured a limited score differentiation, and represented the basis of our qualitative feedback (congruent vs. incongruent) to participants, incongruence scores were based on sample-dependent standardization of scores and featured a fine-grained, quantitative score distribution whose validity has been established in earlier research.

#### Hedonic tone (HT)

To assess HT, we used Brunstein et al.'s (1998, Study 2) German-language 12-item augmented adaptation of the HT scale by Matthews et al. (1990), which was designed to assess the degree of motivational gratification (or frustration) after incentive consummation (or failure to obtain an incentive). It consists of the items *happy, satisfied, contented, cheerful, pleasant, elated, sad, depressed, dissatisfied, gloomy, dejected*, and *sorry*. Items were presented in random order with the primer “During the past week I have felt…” and participants could endorse each item on a 5-point scale featuring the gradations very rarely (1), rarely (2), sometimes (3), often (4), and very often (5). After reversing negative-affect items, a hedonic tone average score was calculated separately for T1 and T2. Coefficient alpha of the HT scale was 0.93 at T1 and 0.92 at T2. One person failed to complete the HT scale at T2; hence *n* = 73 for all analyses involving this variable.

#### Beck depression inventory (BDI)

Participants completed the 21-item BDI (Beck et al., 1961; German version: Hautzinger et al., 1995), a well-validated and widely used measure of depressive symptoms. Item response scales ranged from 0 (description of absence of depressive symptom) to 3 (description of most severe form of symptom). Overall scores, summed across items, can vary between 0 and 63. For T1 BDI scores: *M* = 8.46, *SD* = 6.60, and *alpha* = 0.81; for T2 BDI scores: *M* = 7.21, *SD* = 5.80, and *alpha* = 0.82. Because BDI scores, which ranged from 0 to 29 at T1 and from 0 to 26 at T2, deviated from a normal distribution, we subjected them to square-root transformations after adding a constant of 1 and used these transformed scores in all further analyses. One person failed to complete the BDI at T2; hence *n* = 73 for all analyses involving this variable.

#### Satisfaction with life scale (SWLS)

The SWLS (Diener et al., 1985; German translation: Glaesmer et al., 2015) measures the cognitive-evaluative aspect of subjective well-being. It consists of 5 Items, such as “In most ways my life is close to my ideal,” and its response scale ranges from 1 (*strongly disagree)* to 7 (*strongly agree*). Coefficient alpha of the SWLS scale was 0.76 at T1 and 0.81 at T2.

## Results

### Descriptive statistics and correlations

Table 1 provides descriptive statistics and correlations for all main variables of this study (see the Supplementary for the data and the analysis script). Motive measures showed statistically significant, but moderate retest stability (rs for uncorrected raw scores: n Achievement 0.36, n Power 0.37, n Affiliation 0.44), comparable to what would be expected from earlier studies (Schultheiss and Pang, 2007) and for procedural measures generally (e.g., Strauss et al., 2005; Nosek et al., 2007; Reinecke et al., 2010). None of the three motive measures differed significantly between T1 and T2. Declarative measures of personality (i.e., goal commitment) and well-being (HT, BDI, SWLS) showed the expected substantial monotrait-monomethod variance overlap and featured significant retest stability. HT emerged as the least stable measure and BDI and SWLS as the most stable measures, with goal commitment measures in between. Like in previous studies (e.g., Rawolle et al., 2013), implicit motive and explicit goal commitment measures were not significantly associated within motivational domains.

**Table 1:** Summary of study variable intercorrelations, means, and standard deviations before (T1) and after (T2) treatment.

	**1**	**2**	**3**	**4**	**5**	**6**	**7**	**8**	**9**	**10**	**11**	**12**	**13**	**14**	**15**	**16**	**17**	**18**	**19**	**20**	**21**	**22**	**23**	**24**	**25**	**26**	**27**	**28**	**29**	**30**	**31**	**32**	**33**	**34**
1. n Pow T1	–																																	
2. n Ach T1	0.18	–																																
3. n Aff T1	−0.09	0.35	–																															
4. n Pow T2	0.27	0.13	−0.09	–																														
5. n Ach T2	0.03	0.28	0.40	−0.10	–																													
6. n Aff T2	−0.03	0.32	0.38	−0.26	0.25	–																												
7. Pow goal T1	−0.03	0.09	0.15	−0.17	0.13	0.10	–																											
8. Ach goal T1	−0.03	0.17	0.05	−0.04	−0.02	0.17	0.22	–																										
9. Aff goal T1	0.28	0.15	−0.01	0.06	−0.13	0.01	0.19	0.33	–																									
10. Pow goal T2	0.10	0.14	0.10	−0.01	0.03	−0.04	0.58	0.32	0.28	–																								
11. Ach goal T2	0.10	0.23	0.07	0.07	0.02	0.08	0.35	0.66	0.37	0.47	–																							
12. Aff goal T2	–0.07	0.08	0.02	0.00	−0.02	0.07	0.53	0.31	0.39	0.39	0.39	–																						
13. Pow con T1	0.52	0.14	0.16	0.03	0.08	0.06	0.28	0.05	0.17	0.20	0.10	0.13	–																					
14. Ach con T1	0.03	0.64	0.28	0.04	0.13	0.13	0.16	0.52	0.21	0.28	0.41	0.05	0.04	–																				
15. Aff con T1	−0.01	0.35	0.58	0.03	0.01	0.25	0.22	0.25	0.42	0.28	0.31	0.33	0.07	0.43	–																			
16. ∑ con T1	0.26	0.57	0.50	0.05	0.12	0.21	0.33	0.41	0.39	0.38	0.41	0.25	0.54	0.74	0.74	–																		
17. Pow con T2	0.16	0.23	−0.07	0.57	0.03	−0.08	0.09	0.06	0.21	0.44	0.20	0.14	0.12	0.10	0.07	0.14	–																	
18. Ach con T2	0.10	0.21	0.24	−0.02	0.55	0.36	0.09	0.31	0.05	0.25	0.33	0.14	0.12	0.30	0.20	0.31	0.18	–																
19. Aff con T2	−0.12	0.29	0.27	−0.22	0.14	0.59	0.38	0.26	0.07	0.19	0.27	0.48	0.12	0.22	0.27	0.30	0.05	0.24	–															
20. ∑ con T2	0.07	0.37	0.23	0.16	0.37	0.44	0.28	0.32	0.17	0.44	0.41	0.38	0.18	0.31	0.27	0.38	0.61	0.73	0.64	–														
21. Pow inc T1	−0.02	−0.06	−0.15	0.00	0.01	0.11	−0.23	0.04	−0.03	−0.01	−0.05	−0.08	−0.43	0.01	−0.16	−0.28	−0.03	0.05	0.07	0.05	–													
22. Ach inc T1	0.06	0.07	0.08	−0.06	0.11	0.17	0.16	−0.08	0.03	0.02	0.01	0.20	0.09	−0.29	−0.09	−0.15	−0.01	−0.05	0.20	0.07	0.00	–												
23. Aff inc T1	0.08	0.05	0.10	−0.15	0.18	0.11	0.09	−0.01	−0.02	0.12	−0.02	−0.14	0.17	−0.07	−0.29	−0.09	−0.01	0.00	−0.01	−0.01	0.09	0.44	−											
24. ∑ inc T1	0.07	0.03	0.03	−0.12	0.16	0.19	0.02	−0.02	−0.01	0.07	−0.03	−0.02	−0.06	−0.17	−0.27	−0.25	−0.02	0.00	0.12	0.05	0.50	0.71	0.80	–										
25. Pow inc T2	0.00	−0.13	−0.03	−0.08	−0.05	−0.03	0.01	0.04	−0.15	−0.14	−0.14	0.13	−0.15	−0.11	−0.09	−0.17	−0.37	−0.10	0.09	−0.19	0.26	0.02	−0.05	0.09	–									
26. Ach inc T2	0.07	−0.10	−0.04	−0.08	0.05	−0.22	−0.01	−0.15	−0.07	−0.06	−0.17	−0.29	−0.02	−0.11	−0.27	−0.19	−0.16	−0.41	−0.22	−0.40	0.06	0.10	0.07	0.11	0.04	–								
27. Aff inc T2	0.06	−0.12	−0.11	0.11	0.00	−0.07	−0.18	−0.11	0.01	−0.17	−0.16	−0.13	−0.05	−0.20	−0.17	−0.21	0.03	−0.01	−0.46	−0.21	−0.06	0.10	0.19	0.12	−0.03	−0.06	–							
28. ∑ inc T2	0.07	−0.21	−0.10	−0.03	0.00	−0.19	−0.10	−0.13	−0.13	−0.21	−0.28	−0.18	−0.13	−0.24	−0.31	−0.34	−0.29	−0.31	−0.34	−0.47	0.15	0.13	0.12	0.19	0.59	0.61	0.51	–						
29. HT T1	0.02	0.18	0.10	0.23	0.06	0.07	0.09	0.18	0.20	0.24	0.23	0.07	0.01	0.21	0.07	0.15	0.16	0.25	−0.02	0.20	0.14	−0.11	0.10	0.07	0.01	−0.13	−0.09	−0.13	–					
30. HT T2	0.03	0.18	0.17	0.01	0.19	0.24	0.23	0.16	0.46	0.22	0.07	0.35	0.10	0.14	0.32	0.27	0.15	0.32	0.20	0.34	0.15	0.18	0.13	0.22	−0.11	−0.21	0.08	−0.15	0.29	–				
31. BDI T1	−0.19	−0.35	−0.08	−0.01	−0.11	−0.13	−0.18	−0.26	−0.16	−0.28	−0.29	−0.06	−0.10	−0.33	−0.10	−0.26	−0.01	−0.18	−0.11	−0.16	−0.20	0.08	−0.18	−0.15	−0.11	0.14	0.21	0.14	−0.63	−0.26	–			
32. BDI T2	−0.13	−0.33	−0.13	−0.12	−0.16	−0.17	−0.26	−0.23	−0.24	−0.33	−0.34	−0.22	−0.02	−0.23	−0.28	−0.26	−0.20	−0.24	−0.16	−0.31	−0.08	0.04	−0.02	−0.03	0.09	0.21	0.11	0.24	−0.54	−0.49	0.70	–		
33. SWLS T1	0.10	0.17	0.00	0.24	−0.01	0.09	0.22	0.36	0.21	0.31	0.32	0.28	0.12	0.37	0.19	0.34	0.24	0.22	0.21	0.34	−0.02	−0.04	0.10	0.03	0.02	−0.21	−0.17	−0.21	0.46	0.28	−0.47	−0.44	−	
34. SWLS T2	0.10	0.22	0.07	0.21	0.01	0.16	0.30	0.23	0.18	0.33	0.26	0.36	0.00	0.30	0.24	0.27	0.20	0.17	0.28	0.32	0.09	0.02	0.11	0.11	0.15	−0.14	−0.12	−0.07	0.38	0.35	−0.40	−0.47	0.85	–
*M*	10.02	8.29	10.87	9.26	8.89	10.43	4.07	4.21	3.93	3.97	4.22	4.03	0.31	0.41	0.30	1.01	0.31	0.39	0.31	1.01	0.45	0.34	0.33	0.37	0.35	0.27	0.39	0.34	3.54	3.65	2.88	2.69	4.76	4.86
*SD*	3.93	3.48	4.48	4.15	4.38	4.78	0.80	0.69	0.77	0.83	0.62	0.64	0.47	0.49	0.46	0.96	0.47	0.49	0.47	0.94	0.44	0.45	0.55	0.33	0.51	0.54	0.49	0.29	0.80	0.70	1.09	0.98	1.03	1.06

*n Pow, need for power; n Ach, need for achievement; n Aff, need for affiliation; Pow (ach, aff) goal, power (achievement, affiliation) goal commitment; pow (ach, aff) con, motivational congruence in the power (achievement, affiliation) domain; ∑ con, motivational congruence sum score; pow (ach, aff) inc, motivational incongruence in the power (achievement, affiliation) domain; ∑ inc, motivational incongruence sum score; HT, hedonic tone; BDI, Beck Depression Inventory; SWLS, Satisfaction With Life Scale*.

*Underlined correlations are significant at p < 0.05*.

Congruence indices across all three domains correlated significantly and positively with their constituting motive and goal commitment variables, indicating that part of their variance simply reflected whether a person was high in a given motive or goal commitment. This was not the case for incongruence indices, which were not significantly associated with their constituting motive or goal variables. This suggests that they predominantly captured variance uniquely related to the (lack of) fit between motives and goal commitments, regardless of the absolute levels of their constituting measures. All congruence and incongruence indices, both at the single-domain and aggregated levels, had significant correlations in the expected direction. This bolsters the notion that both index types converged on capturing valid variance reflecting the degree of motivational congruence. At the aggregate level, congruence scores showed significant retest stability, whereas incongruence retest stability, although positive, was not significant and thus appeared to be more variable over time.

Looking at the associations between motives, goal commitments, and their (in)congruence on the one hand and well-being measures on the other, some distinct patterns emerge from the correlation matrix. First, like in the study by Schultheiss et al. (2008), higher levels of implicit motives were associated almost without exception with more well-being. However, replicating earlier findings (see Neumann and Schultheiss, 2015, for an overview), only the negative correlation between n Achievement at T1 and BDI scores (T1 and T2) became significant. Second, higher goal commitment scores were also associated with better well-being, with more than half of the correlations being significant within as well as across T1 and T2. Third, higher congruence scores were associated with higher well-being scores. Notably, with the exception of HT at T1, congruence sum scores at T1 and T2 were significantly correlated with higher concurrent well-being scores. Fourth, at the aggregate sum score level, higher incongruence indices at T2, but not at T1, tended to be associated with lower well-being scores, although this correlation was significant in the case of depressive symptoms only.

### Testing hypothesis 1: does treatment increase motivational congruence?

We ran a repeated-measures ANOVA on congruence scores with Time (T1, T2) and Domain (Power, Achievement, Affiliation) as within-subjects factors and Condition (CG, FB, FB+CET) as between-subjects factor. The Time x Condition effect was not significant, *F*_(2, 71)_ = 2.06, *p* = 0.14, partial η^2^ = 0.055, whereas the Domain × Time × Condition effect was highly significant, *F*_(4, 142)_ = 3.91, *p* = 0.005, partial η^2^ = 0.099, suggesting that treatment effects were not uniform across motivational domains. Because we had observed substantial overlap between all congruence indices and the motive and goal commitment variables constituting them, we repeated our analysis and this time included all six goal commitment and all six implicit motive variables as covariates. Despite the loss of degrees of freedom and massive control for variance overlap between congruence measures and their source variables inherent in this destructive testing approach, the Domain × Time × Condition remained significant, *F*_(4, 118)_ = 2.74, *p* = 0.03, partial η^2^ = 0.085, indicating that the effect cannot be attributed to mere main effects of the variables going into the construction of congruence indices.

**Figure 2:**
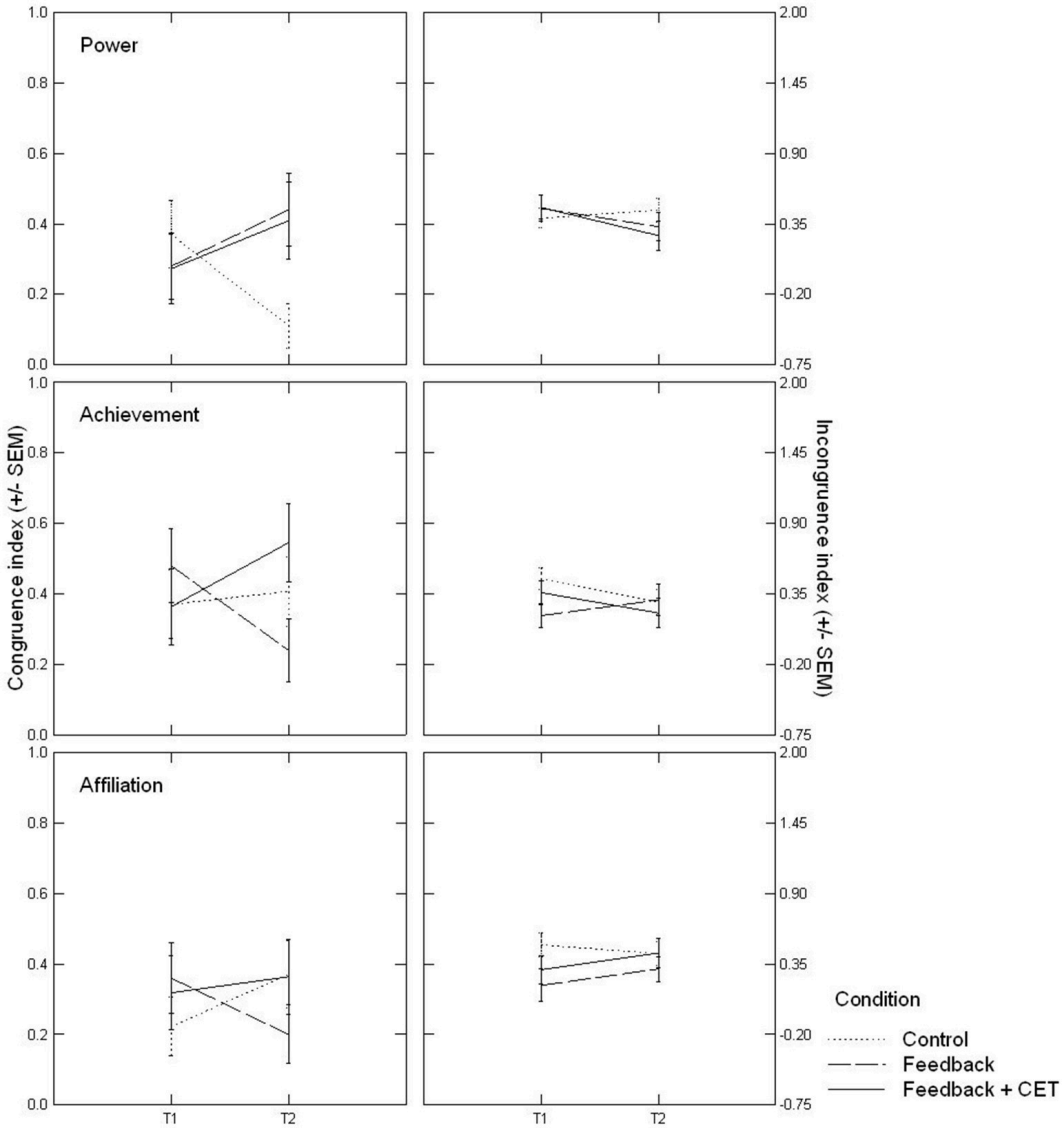
Time × Condition effects, depicted separately by domain, on motivational congruence **(Left)** and motivational incongruence **(Right)**. CET, Congruence-enhancement training.

Follow-up analyses for the original model indicated that at T1, participants did not significantly differ from each other on any congruence measure by Condition or by the Condition × Domain interaction (*p*s > 0.63), thus ruling out the possibility that treatment effects were primarily due to initial between-group differences. Separate repeated-measures ANOVAs for each motivational domain revealed significant Time × Condition effects for power, *F*_(2, 71)_ = 4.04, *p* = 0.02, partial η^2^ = 0.102, and achievement, *F*_(2, 71)_ = 3.41, *p* = 0.04, partial η^2^ = 0.088, but not for affiliation, *F*_(2, 71)_ = 2.07, *p* = 0.13, partial η^2^ = 0.055. Figure 2 depicts the differential changes from T1 to T2 in the three groups separately for each domain.

As illustrated, congruence increased in the power domain both for participants in the FB group, *t*_(24)_ = 1.28, 95% CI = [−0.10; 0.42], *p* = 0.21, and for participants in the FB+CET group, *t*_(21)_ = 1.14, 95% CI = [−0.11; 0.38], *p* = 0.27 [for both treatment groups combined: *t*_(46)_ = 1.73, 95% CI = [−0.02; 0.32], *p* = 0.09], whereas it decreased for control-group participants, *t*_(26)_ = –2.27, 95% CI = [−0.49; –0.02], *p* = 0.03. As a consequence, the three groups significantly differed in their power congruence scores at T2, *F*_(2, 71)_ = 4.28, *p* = 0.02, η^2^ = 0.108, and *post-hoc* tests (Tukey) indicated that the control group had significantly lower scores than the FB group, *p* = 0.03, 95% CI = [−0.63; –0.03] and, at the trend level, the FB+CET group, *p* = 0.06, 95% CI = [−0.60; 0.01], which in turn did not differ from each other, *p* = 0.97, 95% CI = [−0.28; 0.34].

In the achievement domain, congruence increased only for FB+CET participants, *t*_(24)_ = 1.70, 95% CI = [−0.04; 0.40], *p* = 0.10, whereas it decreased for FB participants, *t*_(24)_ = −2.30, 95% CI = [−0.46; −0.02], *p* = 0.03, and remained unchanged for controls, *t*_(26)_ = 0.30, 95% CI = [−0.22; 0.29], *p* = 0.77. The three groups showed a difference trend in their achievement congruence scores at T2, *F*_(2, 71)_ = 2.37, *p* = 0.10, η^2^ = 0.062. *Post-hoc* tests indicated that the main difference was between the FB and the FB+CET groups, *p* = 0.08, 95% CI = [−0.03; 0.64], with the control group not being significantly different from either the FB group, *p* = 0.43, 95% CI = [−0.15; 0.49], or the FB+CET group, *p* = 0.58, 95% CI = [−0.47; 0.19]. With the exception of the domain of affiliation, then, Hypothesis 1 received support for congruence scores, with post-treatment congruence increases observed for the FB+CET group.

We also ran a repeated-measures ANOVA on incongruence scores with Time (T1, T2) and Domain as within-subjects factors and Condition as between-subjects factor. However, neither the Time × Condition effect, *F*_(2, 71)_ = 0.63, *p* = 0.54, partial η^2^ = 0.017, nor the Domain × Time × Condition effect became significant, *F*_(4, 142)_ = 1.75, *p* = 0.14, partial η^2^ = 0.047. When we simplified the factor condition into one combined (FB and FB+CET) treatment level vs. the control group, the Domain × Time × Condition effect approached significance, *F*_(2, 142)_ = 2.84, *p* = 0.06, partial η^2^ = 0.038, suggesting that the combined treatment effect was not uniform across motivational domains. Follow-up analyses revealed a Time × Condition effect in the power domain, *F*_(1, 72)_ = 3.08, *p* = 0.08, partial η^2^ = 0.041, that did not emerge for achievement, *F*_(1, 72)_ = 1.15, *p* = 0.29, partial η^2^ = 0.016, or affiliation, *F*_(1, 72)_ = 1.53, *p* = 0.22, partial η^2^ = 0.021. As illustrated in Figure 2, the interaction effect in the power domain was due to a significant reduction of incongruence scores in the combined treatment groups, *t*_(46)_ = −2.07, 95% CI = [−0.35; −0.00], *p* = 0.04, that did not occur in the control group, *t*_(26)_ = 0.62, 95% CI = [−0.15; 0.27], *p* = 0.54. However, the between-group difference at T2 was not significant, *t*_(72)_ = 1.33, 95% CI = [−0.08; 0.40], *p* = 0.19. Thus, Hypothesis 1 could only be tentatively confirmed for the power domain with regard to incongruence scores, but not for the achievement or the affiliation domain.

### Testing hypothesis 2: does treatment increase well-being?

**Figure 3:**
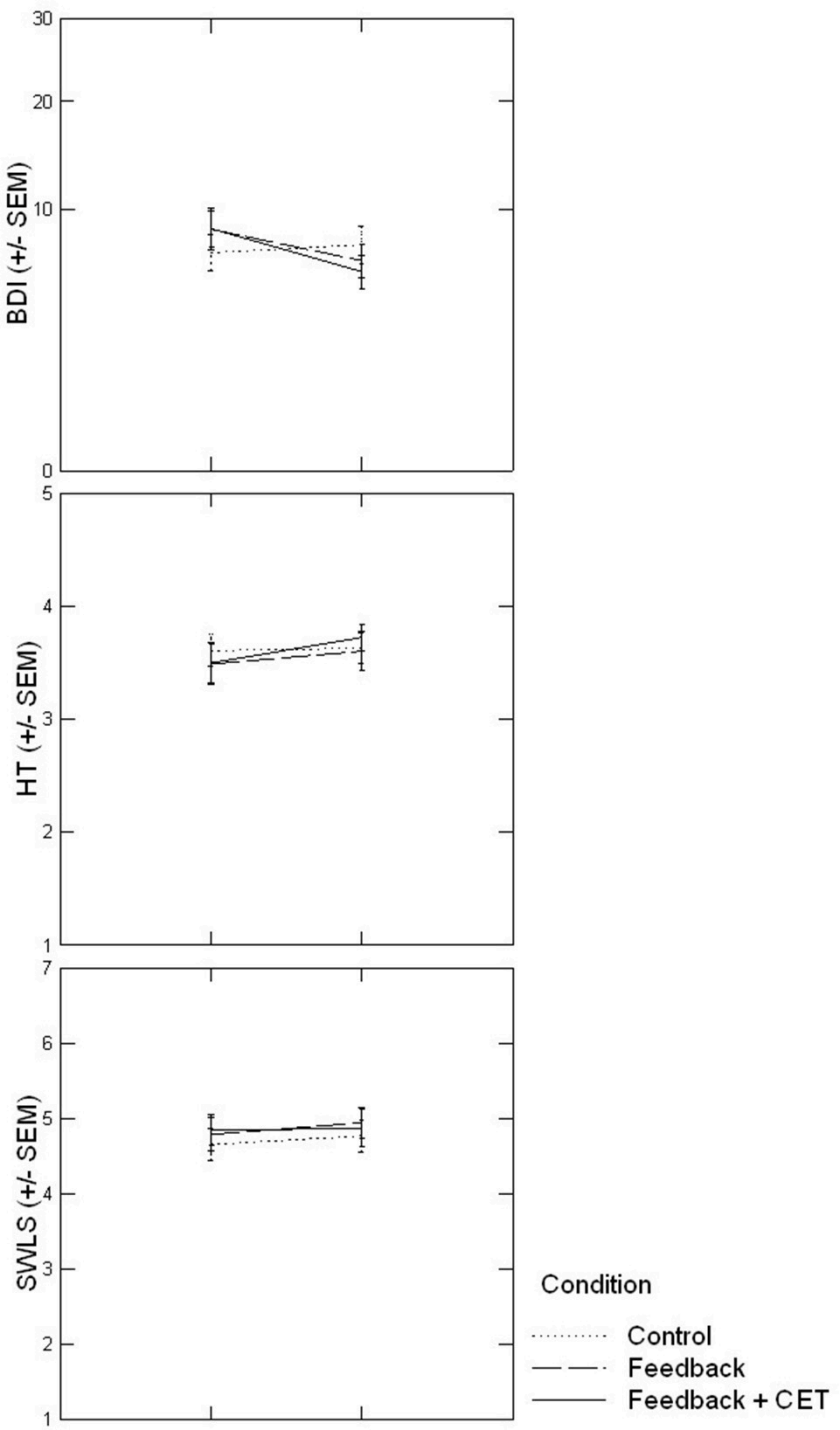
Time × Condition effects on three measures of well-being. CET, Congruence-enhancement training.

A MANOVA with the three well-being measures at T1 as dependent variables and Condition and Measure as factors indicated neither a main effect of Condition nor a Condition x Measure interaction (*p*s > 0.83), which suggests that the groups did not systematically differ on well-being measures at T1. Repeated-measures ANOVAs on HT, BDI, or SWLS did not yield evidence for a Time × Condition effect in support of Hypothesis 2, *F*_(2, 70)_ = 0.31, *p* = 0.73, partial η^2^ = 0.009, *F*_(2, 70)_ = 1.05, *p* = 0.35, partial η^2^ = 0.029, and *F*_(2, 71)_ = 0.29, *p* = 0.75, partial η^2^ = 0.008, respectively. However, when we changed our analytical approach and controlled for baseline variance in BDI scores in an ANCOVA, the combination of both treatment groups, relative to the control group, had a marginally significant effect on BDI scores at T2, *F*_(1, 70)_ = 2.78, *p* = 0.099, partial η^2^ = 0.038. As illustrated in Figure 3, BDI scores declined from T1 to T2 in the FB and FB+CET groups, *t*_(45)_ = −2.44, 95% CI = [−0.50; −0.05], *p* = 0.02, but not in controls, *t*_(26)_ = 0.09, 95% CI = [−0.34; 0.37], *p* = 0.93. Despite this, BDI scores did not significantly differ by condition at T2, *t*_(71)_ = 1.18, 95% CI = [−0.19; 0.76], *p* = 0.24 [for T1, *t*_(72)_ = 0.13, 95% CI = [−0.56; 0.50], *p* = 0.90]. Thus, we found very limited support for Hypothesis 2.

### Testing hypothesis 3: do changes in motivational congruence mediate treatment effects on well-being?

We first explored whether changes in overall (in)congruence and more specifically changes in power and achievement indices (i.e., the two domains that were most sensitive to the treatment), combined into agentic (in)congruence indices, were associated with changes in HT, BDI, and SWLS scores from T1 to T2. We used bipartial correlation analysis, which allowed us to determine the degree of association between two variables, with each being partialed for its own initial levels (see Cohen and Cohen, 1983).

**Figure 4:**
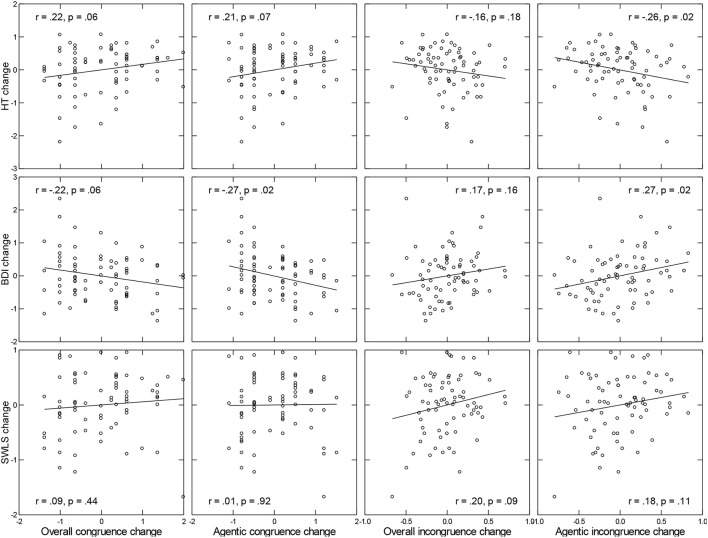
Associations between changes in indicators of well-being (horizontal) and indicators of congruence (**Left** two columns) and incongruence (**Right** two columns). Coefficients provided in each panel represent bipartial correlations.

As illustrated in Figure 4, changes in BDI and HT were systematically associated with both congruence and incongruence measures. Echoing the results reported above, these associations were stronger for agentic (in)congruence indices than overall indices. In general, greater increases in motivational congruence (or decreases in motivational incongruence) were associated with greater increases in HT and greater decreases in BDI scores. Associations between changes in (in)congruence indices and changes in SWLS were weak and mostly non-significant. We also tested whether changes of incongruence scores in the power domain alone, for which we had observed a treatment effect, were associated with changes in any of the well-being measures and thus potentially qualified as a mediator of treatment effects on well-being, but found no significant associations.

However, only the effect of treatment on BDI changes reported in the previous section qualified for potential mediation and only changes in agentic congruence and in power-related incongruence scores qualified as potential mediators (see Baron and Kenny, 1986). Changes in both measures of motivational congruence were related to reductions in BDI scores in the combined treatment groups (FB and FB+CET), with bipartial rs of −0.29 for agentic congruence changes and 0.27 for power incongruence changes (*p*s < 0.08), but not in the control group (bipartial rs = −0.19 and 0.09, respectively; *p*s > 0.35). Again using an ANCOVA approach, we next tested whether condition (combined FB and FB+CET groups vs. control group) had an effect on agentic congruence and power incongruence at T2, with T1 held constant. This was the case at the trend level for agentic congruence, *F*_(1, 71)_ = 3.21, *p* = 0.08, partial η^2^ = 0.090, but not for power incongruence, *F*_(1, 71)_ = 2.50, *p* = 0.12, partial η^2^ = 0.075, thus ruling out the latter as a mediator. When we finally repeated the original ANCOVA with treatment as predictor, BDI at T2 as criterion, BDI at T1 as covariate and, in addition, agentic congruence indices for T1 and T2 to represent changes in congruence, the effect of treatment was no longer significant, *p* = 0.14, whereas agentic congruence at T2 was, *p* = 0.02. However, the Sobel test statistic (−1.36, SE = 0.06, *p* = 0.17) failed to support the notion that treatment effects of feedback and congruence training decreased depressive symptoms through increases in agentic congruence. Thus, despite evidence for associated changes in motivational (in)congruence and affective facets of well-being, hypothesis 3 was not supported.

### Additional analyses

To examine the possibility that our interventions changed implicit motives or explicit goal commitments *per se* (which was, after all, not the intention of our approach), we ran two additional repeated-measures ANOVAs, with Domain and Time as within-subjects factors, Condition as between-subjects factor, and (1) implicit motive scores and (2) goal commitment scores as dependent variables. The Time × Condition and the Domain × Time × Condition effects were not significant for either implicit motives or for goal commitments (*p*s >.26). These results indicate that neither motives nor goal commitments changed, globally or differentially, as a function of experimental condition.

## Discussion

In this longitudinal study, we tested for the first time whether congruence between implicit motives and explicit personal goal commitments, which in this and other studies typically fail to correlate, can be increased by (a) providing people with feedback about their implicit motives and their fit to their goal commitments and (b) additionally training some of these people in how to enhance motive-goal congruence. We expected these interventions, when compared to a control group, to increase motivational congruence (Hypothesis 1) and well-being (Hypothesis 2), and motivational congruence changes to mediate the effect of treatment on well-being changes (Hypothesis 3).

We obtained partial support for Hypothesis 1 and thus for the primary goal of this study: participants in the treatment groups, and most consistently those who received both motivational feedback and congruence enhancement training (FB+CET), showed an increase in motivational congruence indices in the domains of power and achievement and a decrease in power-related incongruence scores. None of these changes could be attributed to direct changes in goal commitments or implicit motive levels, which we did not aim for with our intervention strategy, or, in the case of congruence scores, to the variance overlap between the motive and goal commitment variables constituting these scores.

However, we failed to observe treatment effects on congruence in the affiliation domain for both types of scores and on achievement-related incongruence scores. Whereas in the case of achievement incongruence our study probably lacked the necessary power to bring the hypothesized effect over the statistical threshold (participants in the FB+CET group showed the predicted incongruence decrease after all), the affiliation domain results are unexpected. Perhaps our proactive, problem-solving-oriented approach to congruence enhancement was more suitable for the agentic domains of power and achievement than for the more communal, being-oriented affiliation-intimacy domain (see McAdams and Constantian, 1983, p. 852). This explanation is supported by the observation that whereas power-motivated individuals prefer activities that are engaging and exciting (e.g., analyzing one's motive-goal fit and coming up with ways to enhance it in the present study), affiliation-motivated individuals seem to be more at one with themselves when they can feel calm and relaxed (Job et al., 2012). Future research could explore whether the latter would benefit more from a congruence-enhancing approach that utilizes relaxation and mindfulness (see Strick and Papies, 2017).

But perhaps our present findings are due to a methodological shortcoming of Winter's (1994) coding system. As reported by Winter (1991), the criteria for coding n Affiliation with his running-text system failed to differentiate affiliation-arousal from control-group participants in one study and despite good statistical power only reached the 0.05 probability level for discriminating the two groups in another study. In other words, Winter's n Affiliation content-coding measure may not be well-suited for differentiating between people who are in a transient or chronic state of affiliation motivation from those who are not. Future studies exploring effects of CET should therefore consider using the original coding systems for affiliation motivation for more valid measurement of this domain (Atkinson et al., 1958; McAdams, 1980; McKay, 1992).

Evidence supporting Hypothesis 2 was limited to reductions in depressive symptoms. We found that, overall, participants in the treatment groups showed a trend toward greater symptom reduction than controls. That we did not see a stronger effect for BDI score changes may be due to the limited score range in the non-clinical student sample we tested. However, treatment had no discernible impact on changes in felt happiness, despite the fact that we had used the same hedonic-tone scale that had been sensitive to motive-goal interactions in Brunstein et al.'s (1998) work (see also Schultheiss et al., 2008; Schultheiss, 2013, Study 2). However, in their research emotional well-being not only depended on the fit between implicit motives and personal goal commitments. The effect of this fit was in turn moderated by goal attainability, with motive-goal congruence only predicting more happiness if goal attainability was high, too, because only then did participants also report high rates of goal progress, the proximal predictor of emotional well-being in these studies. In contrast, our interventions focused on the degree of commitment participants felt toward their goals, but did not consider to what extent these goals were easy or hard to realize in participants' everyday lives. We therefore suggest that while our intervention approach was effective in reducing symptoms of depression, it may not have been strong enough to positively increase happiness by facilitating goal progress. To achieve greater impact on all facets of affective well-being, future studies should therefore complement the congruence enhancement approach we used with interventions that target better goal attainability and directly promote goal implementation (e.g., Brunstein et al., 2008).

Treatment effects were also absent for changes in life satisfaction. However, this measure is generally viewed as a cognitive more than as an affective facet of well-being (Andrews and McKennell, 1980; Diener et al., 1985) and hence principally less susceptible to the influence of affect-generating implicit motives and their interactions with goal processes.

Because treatment effects on well-being were limited, so were our odds for supporting Hypothesis 3. Neither of two potential mediators for the treatment effect on depressive symptom reduction—agentic congruence and power-related incongruence—reached statistical significance in our mediation analysis. Nevertheless, changes in both were more strongly associated with changes in depressive symptoms in the treatment conditions than in the control condition. This suggests that insufficient test power may have been another limiting factor for documenting mediation.

In exploring associations between changes in motivational congruence and changes in well-being in preparation for mediation analyses, we found surprisingly robust and consistent result patterns. We documented for the first time that increases in overall motivational congruence, and even more so in agentic congruence, are associated with increased emotional well-being, as assessed through changes in happiness and depressive symptoms. As suggested by the above, these associations could only partially be attributed to our interventions, because neither did several of the domain-specific (in)congruence scores included in these overall scores show a treatment effect, nor were changes in hedonic tone sensitive to our interventions. Thus, additional factors must have contributed to changes in these measures as well as to their covariation. Our longitudinal findings thus reinforce a fundamental notion of implicit motive research (McClelland et al., 1989) and, indeed, clinical psychology (Fries and Grawe, 2006)—that alignment between what people desire in their heart of hearts vs. what they think they ought to pursue in life is emotionally salubrious, whereas a lack of such alignment is not. In contrast, changes in participants' life satisfaction (which was itself highly stable from T1 to T2) were not related in any systematic or meaningful manner to changes in motivational congruence indices, supporting the previously mentioned view that life satisfaction represents a cognitive rather than an affective facet of well-being.

To summarize, the present research provides (a) preliminary evidence of congruence-enhancing effects of our intervention approach, particularly for the full combination of feedback and CET and particularly in the agentic domain, (b) limited evidence for an effect on well-being which is restricted to the reduction of depressive symptoms, and (c) no systematic evidence for congruence increases mediating the effect of treatment on depressive symptom reduction. Despite this, our findings also provide (d) substantial support for the idea that changes in motivational congruence are generally associated with changes in emotional well-being over time.

## Limitations and future directions

The validity of our study and its findings is limited by the following factors. First, we did not include a control group receiving placebo training. We therefore cannot say for certain to what extent the treatment effects we observed were due to the specific content of our intervention or the fact that participants simply met an additional time with the experimenter and other participants between T1 and T2 assessments.

Second, although our findings document some degree of effectiveness of our interventions for increasing agentic motivational congruence, a detailed dissection of the various components, such as goal clarification, psychoeducation, and resource activation, included in our interventions will be necessary in future research to pinpoint whether all interventions contributed equally to this effect or whether some were more effective than others. Moreover, to the extent that the lack of effect in the affiliation domain is not simply due to the previously discussed restricted validity of the n Affiliation measure, future studies need to analyze the reasons why it may be difficult to increase motivational congruence in this domain and design suitable interventions to address this issue.

Third, the experimenter was not blind to the hypotheses of the study, and this may have influenced the results in covert ways. However, our results suggest that random participant allocation to conditions was mostly successful. Moreover, all PSE content coding was done blindly with regard to participant group assignment and all other data acquisition, processing, and analysis was carried out based on automatic algorithms written and fully documented as code (see Supplement Material). It is therefore difficult to pinpoint any obvious step at which bias associated with the hypotheses may have compromised the integrity of our data.

Fourth, the size of treatment effects on indices of motivational congruence and well-being measures in most cases accounted for 5–10% of variance in this study. Although they thus exceed the threshold for being practically useful (Ferguson, 2009), they represent only a first stab at estimating the true effect of congruence-enhancing interventions. Even when 10% of accounted variance is viewed as an accurate estimate of treatment effect sizes, the *post-hoc* power of our present study, calculated with G^*^Power (Faul et al., 2009) was at 70% and thus somewhat low to reliably detect such effects. Hence, future studies on congruence-enhancement interventions should be more adequately powered, with cell sample sizes exceeding 31 to ensure a power of 80% to properly test their effectiveness.

## Conclusion

The present study is the first to describe and evaluate strategies that aim at helping people attain better congruence between their implicit motivational needs and their explicit personal goal commitments. Our findings suggest that motivational congruence *can* be effectively enhanced and that changes in motivational congruence are associated with changes in affective well-being. While more research is needed to increase the impact of interventions on well-being and extend them to the affiliative domain, we view our results as evidence for the importance and usefulness of considering individuals' implicit motives when discussing their personal goals with them in therapeutic contexts.

## Ethics statement

This study was carried out in accordance with the recommendations of the American Psychological Association's Ethics Code, with written informed consent from all subjects. All subjects gave written informed consent in accordance with the Declaration of Helsinki.

## Author contributions

OS and RR designed the study; AR programmed all computer tasks and RR created all training materials, recruited, and tested all participants, coded all picture stories, and processed and integrated all data files; RR and OS conducted all statistical analyses; OS and RR wrote the manuscript, with contributions by AR during the editing phase; OS revised the manuscript in response to two reviewers' comments and suggestions; all authors read and approved of the final manuscript.

### Conflict of interest statement

The authors declare that the research was conducted in the absence of any commercial or financial relationships that could be construed as a potential conflict of interest.
