# Supplementary material for: Enhancing Congruence between Implicit Motives and Explicit Goal Commitments: Results of a Randomized Controlled Trial
Source: Front Psychol. 2017 Sep 12;8:1540. doi: 10.3389/fpsyg.2017.01540 (PMC5600949; doi:10.3389/fpsyg.2017.01540)
Supplement: Supplementary file 2 [file DataSheet1.ZIP › Roch_et_al_Frontiers_statistical_output.docx]

> *!!!!!!!!!!!!!!!!!!!!!!!!!!!!!!!!!!!!!!!!!!!!!!!!!!!!!!!!!!!!!!!!!!!!!!!!!!!!!!!!!!!!!!!!!!!!!!!!!!!!!!!!!!!!!!!!!!!!!!!!!!!!!!*

> *!!*

> *!!*

> *!! D A T A A N A L Y S E S F O R R O C H , R Ö S C H , & S C H U L T H E I S S*

> *!!*

> *!! M A R C H 1 3 , 2 0 1 7 , U S I N G S Y S T A T 1 3*

> *!!*

> *!! D A T A F I L E : R O C H _ E T _ A L _ S Y S T A T _ D A T A . S Y Z ( S Y S T A T )*

> *!!*

> *!!*

> *!!!!!!!!!!!!!!!!!!!!!!!!!!!!!!!!!!!!!!!!!!!!!!!!!!!!!!!!!!!!!!!!!!!!!!!!!!!!!!!!!!!!!!!!!!!!!!!!!!!!!!!!!!!!!!!!!!!!!!!!!!!!!!*

> *use " Roch_et_al_Frontiers_SYSTAT_data.syz"*

[▼File: Roch_et_al_Frontiers_SYSTAT_data.syz](file:///\\Untitled.syo)

| Number of Variables | : | 280 |
| --- | --- | --- |
| Number of Cases | : | 74 |

SYSTAT Rectangular file Roch_et_al_Frontiers_SYSTAT_data.syz,

Created data file Mon Mar 13 12:55:51 2017 containing variables:

| SUBJECT | ACH_1_T2 | ACH_2_T2 | ACH_3_T2 | ACH_4_T2 | ACH_5_T2 |
| --- | --- | --- | --- | --- | --- |
| ACH_6_T2 | ACH_7_T2 | ACH_8_T2 | ACH_9_T2 | ACH_10_T2 | ACH_11_T2 |
| ACH_12_T2 | ACH_13_T2 | ACH_14_T2 | AFF_1_T2 | AFF_2_T2 | AFF_3_T2 |
| AFF_4_T2 | AFF_5_T2 | AFF_6_T2 | AFF_7_T2 | AFF_8_T2 | AFF_9_T2 |
| AFF_10_T2 | AFF_11_T2 | AFF_12_T2 | AFF_13_T2 | AFF_14_T2 | IND_1_T2 |
| IND_2_T2 | IND_3_T2 | IND_4_T2 | IND_5_T2 | IND_6_T2 | IND_7_T2 |
| IND_8_T2 | IND_9_T2 | IND_10_T2 | IND_11_T2 | IND_12_T2 | IND_13_T2 |
| IND_14_T2 | PSE_1_ACHT1 | PSE_2_ACHT1 | PSE_3_ACHT1 | PSE_4_ACHT1 | PSE_5_ACHT1 |
| PSE_6_ACHT1 | PSE_1_AFFT1 | PSE_2_AFFT1 | PSE_3_AFFT1 | PSE_4_AFFT1 | PSE_5_AFFT1 |
| PSE_6_AFFT1 | PSE_1_INDT1 | PSE_2_INDT1 | PSE_3_INDT1 | PSE_4_INDT1 | PSE_5_INDT1 |
| PSE_6_INDT1 | WORDCOUNT_TOTAL- T1 | __STORIES_WRITT- ENT1 | INHIBITION_TOTA- LT1 | WORDCOUNT1_LABT- 1 | WORDCOUNT2_BOXE- RT1 |
| WORDCOUNT3_TRAP- T1 | WORDCOUNT4_BRID- GET1 | WORDCOUNT5_CAPT- T1 | WORDCOUNT6_NIGH- TT1 | INHIBITION1_LAB- T1 | INHIBITION2_BOX- ERT1 |
| INHIBITION3_TRA- PT1 | INHIBITION4_BRI- DGET1 | INHIBITION5_CAP- TT1 | INHIBITION6_NIG- HTT1 | BDI | BDI1 |
| BDI2 | BDI3 | BDI4 | BDI5 | BDI6 | BDI7 |
| BDI8 | BDI9 | BDI10 | BDI11 | BDI12 | BDI13 |
| BDI14 | BDI15 | BDI16 | BDI17 | BDI18 | BDI19 |
| BDI20 | BDI21 | SWLS | SWLS1 | SWLS2 | SWLS3 |
| SWLS4 | SWLS5 | ZUFRIEDEN | AKTIV | ANGENEHM | AUFGEREGT |
| BEDRUCKT | BETRUBT | DEPRIMIERT | ENERGIELOS | ENTSPANNT | ENTTAUSCHT |
| FREUDIG | FRUSTRIERT | GELASSEN | GLUCKLICH | GUTGELAUNT | HEITER |
| HELLWACH | NERVOS | PASSIV | RUHIG | TATKRAFTIG | TRAURIG |
| TRAGE | UNRUHIG | UMACL_E_MEAN | UMACL_A_MEAN | UMACL_HT_MEAN | UMACL_HTPLUS_ME- AN |
| UMACL_HTMINUS_M- EAN | ALTER | BRILLE | BRILLE2 | GESCHLECHT | HAND |
| HERKUNFT | SPRACHE | STUDIUM$ | BDI_T2 | BDI1_T2 | BDI2_T2 |
| BDI3_T2 | BDI4_T2 | BDI5_T2 | BDI6_T2 | BDI7_T2 | BDI8_T2 |
| BDI9_T2 | BDI10_T2 | BDI11_T2 | BDI12_T2 | BDI13_T2 | BDI14_T2 |
| BDI15_T2 | BDI16_T2 | BDI17_T2 | BDI18_T2 | BDI19_T2 | BDI20_T2 |
| BDI21_T2 | SWLS_T2 | SWLS1_T2 | SWLS2_T2 | SWLS3_T2 | SWLS4_T2 |
| SWLS5_T2 | ZUFRIEDEN_T2 | AKTIV_T2 | ANGENEHM_T2 | AUFGEREGT_T2 | BEDRüCKT_T2 |
| BETRüBT_T2 | DEPRIMIERT_T2 | ENERGIELOS_T2 | ENTSPANNT_T2 | ENTTäUSCHT_T2 | FREUDIG_T2 |
| FRUSTRIERT_T2 | GELASSEN_T2 | GLüCKLICH_T2 | GUT_GELAUNT_T2 | HEITER_T2 | HELLWACH_T2 |
| NERVöS_T2 | PASSIV_T2 | RUHIG_T2 | TATKRäFTIG_T2 | TRAURIG_T2 | TRäGE_T2 |
| UNRUHIG_T2 | UMACL_E_MEAN_T2 | UMACL_A_MEAN_T2 | UMACL_HT_MEAN_T- 2 | UMACL_HTPLUS_ME- AN_T2 | UMACL_HTMINUS_M- EAN_T2 |
| WORDCOUNT_TOTAL- _T2 | INHIBITION_TOTA- L_T2 | BLOCKNR1_LAB_T2 | BLOCKNR2_BOXER_- T2 | BLOCKNR3_TRAP_T- 2 | BLOCKNR4_BRIDGE- _T2 |
| BLOCKNR5_CAPT_T- 2 | BLOCKNR6_NIGHT_- T2 | WORDCOUNT1_LAB_- T2 | WORDCOUNT2_BOXE- R_T2 | WORDCOUNT3_TRAP- _T2 | WORDCOUNT4_BRID- GE_T2 |
| WORDCOUNT5_CAPT- _T2 | WORDCOUNT6_NIGH- T_T2 | INHIBITION1_LAB- _T2 | INHIBITION2_BOX- ER_T2 | INHIBITION3_TRA- P_T2 | INHIBITION4_BRI- DGE_T2 |
| INHIBITION5_CAP- T_T2 | INHIBITION6_NIG- HT_T2 | PSE_S1_ACH_T2 | PSE_S2_ACH_T2 | PSE_S3_ACH_T2 | PSE_S4_ACH_T2 |
| PSE_S5_ACH_T2 | PSE_S6_ACH_T2 | PSE_S1_AFF_T2 | PSE_S2_AFF_T2 | PSE_S3_AFF_T2 | PSE_S4_AFF_T2 |
| PSE_S5_AFF_T2 | PSE_S6_AFF_T2 | PSE_S1_POW_T2 | PSE_S2_POW_T2 | PSE_S3_POW_T2 | PSE_S4_POW_T2 |
| PSE_S5_POW_T2 | PSE_S6_POW_T2 | CONDITION | TREAT | ACH_1_T1 | ACH_2_T1 |
| ACH_3_T1 | ACH_4_T1 | ACH_5_T1 | ACH_6_T1 | ACH_7_T1 | ACH_8_T1 |
| ACH_9_T1 | ACH_10_T1 | ACH_11_T1 | ACH_12_T1 | ACH_13_T1 | ACH_14_T1 |
| AFF_1_T1 | AFF_2_T1 | AFF_3_T1 | AFF_4_T1 | AFF_5_T1 | AFF_6_T1 |
| AFF_7_T1 | AFF_8_T1 | AFF_9_T1 | AFF_10_T1 | AFF_11_T1 | AFF_12_T1 |
| AFF_13_T1 | AFF_14_T1 | IND_1_T1 | IND_2_T1 | IND_3_T1 | IND_4_T1 |
| IND_5_T1 | IND_6_T1 | IND_7_T1 | IND_8_T1 | IND_9_T1 | IND_10_T1 |
| IND_11_T1 | IND_12_T1 | IND_13_T1 | IND_14_T1 |  |  |

> *!! Note on variable nomenclature:*

> *!!*

> *!! Variables named Ach_1_T1 etc. refer to Personal Goals Questionaire items 1 through 14 for the domains achievement (ACH), affiliation-intimacy (AFF), and independence
(IND) for initial (T1) and follow-up (T2) assessment.*

> *!! Items 4, 7, 10, and 13 represent the 4 commitment items (other items: goal progress, goal attainability). Aggregated commitment scale names are PGQ_ACH_C_T1 (etc).*

> *!!*

> *!! Implicit motive variables start with PSE_ and are using the same sublabels as the PGQ scales (note that we continued the use of the word "independence" for power, as
introduced by Brunstein, Schultheiss & Grässmann, 1998).*

> *!!*

> *!! WORDCOUNT_ and INHIBITION_ variables represent single-picture word counts and activity inhibition raw scores (see Schultheiss, Riebel, & Jones, 2009).*

> *!!*

> *!! BDI: Beck Depression Inventory (single-item and overall-score variables); SWLS: Satisfaction with life scale (single-item and overall-score variables); UMACL_E/A/HT:
University of Wales Mood Adjective Check List scales*

> *!! energization/tension/hedonic tone (variables "zufrieden" to "unruhig" represent individual UMACL items)*

> *!!*

> *!! ALTER: Age in years; GESCHLECHT: Gender (1 = female, 2 = male); CONDITION: treatment condition (1 = control group, 2 = feedback, 3 = feedback + congruence-enhancement
training); TREAT: simplified treatment condition*

> *!! (0 = control group, 1 = FB/FB+CET)*

> *!!*

> *!!*

> *!! The following two participants were deleted previously because they switched categories after recoding (see paper)*

> *!!*

> *!! if subject = 103 then delete*

> *!! if subject = 203 then delete*

> *idvar subject*

> *format 12, 8*

> *echo on*

> *categ condition treat*

> *LABEL treat / 0 = "CG" 1 = "FB/FB+CET"*

> *!!!!!!!!!!!!!!!!!!!!!!!!!!!!!!!!!!!!!!!!!!!!!!!!!!!!!!!!!!!!!!!!!!!!!!!!!!!!!!!!!!!!!!!!!!!!!*

> *!!*

> *!! M E T H O D*

> *!!*

> *!!!!!!!!!!!!!!!!!!!!!!!!!!!!!!!!!!!!!!!!!!!!!!!!!!!!!!!!!!!!!!!!!!!!!!!!!!!!!!!!!!!!!!!!!!!!!*

> *!! Check for subject # duplication due to errors in data aggregation*

> *XTAB*

> *PLENGTH NONE / FREQ*

> *TABULATE SUBJECT*

[▼One-Way Frequency Distribution](file:///\\Untitled.syo)

**Counts**

| **Values for Subject** | | | | | | | | | | | | | | | | | | | | |
| --- | --- | --- | --- | --- | --- | --- | --- | --- | --- | --- | --- | --- | --- | --- | --- | --- | --- | --- | --- | --- |
| **102** | **104** | **105** | **106** | **107** | **202** | **205** | **206** | **207** | **208** | **209** | **210** | **302** | **303** | **304** | **305** | **306** | **307** | **308** | **309** | **403** |
| 1 | 1 | 1 | 1 | 1 | 1 | 1 | 1 | 1 | 1 | 1 | 1 | 1 | 1 | 1 | 1 | 1 | 1 | 1 | 1 | 1 |

| **Values for Subject** | | | | | | | | | | | | | | | | | | | | |
| --- | --- | --- | --- | --- | --- | --- | --- | --- | --- | --- | --- | --- | --- | --- | --- | --- | --- | --- | --- | --- |
| **404** | **405** | **406** | **407** | **408** | **409** | **410** | **501** | **502** | **503** | **504** | **505** | **506** | **507** | **508** | **509** | **601** | **602** | **603** | **604** | **605** |
| 1 | 1 | 1 | 1 | 1 | 1 | 1 | 1 | 1 | 1 | 1 | 1 | 1 | 1 | 1 | 1 | 1 | 1 | 1 | 1 | 1 |

| **Values for Subject** | | | | | | | | | | | | | | | | | | | | |
| --- | --- | --- | --- | --- | --- | --- | --- | --- | --- | --- | --- | --- | --- | --- | --- | --- | --- | --- | --- | --- |
| **606** | **607** | **608** | **701** | **702** | **703** | **705** | **706** | **707** | **801** | **802** | **803** | **804** | **805** | **806** | **807** | **808** | **901** | **902** | **903** | **904** |
| 1 | 1 | 1 | 1 | 1 | 1 | 1 | 1 | 1 | 1 | 1 | 1 | 1 | 1 | 1 | 1 | 1 | 1 | 1 | 1 | 1 |

| **Values for Subject** | | | | | | | | | | | |
| --- | --- | --- | --- | --- | --- | --- | --- | --- | --- | --- | --- |
| **905** | **906** | **907** | **1,001** | **1,002** | **1,003** | **1,101** | **1,102** | **1,103** | **1,201** | **1,202** | **Total** |
| 1 | 1 | 1 | 1 | 1 | 1 | 1 | 1 | 1 | 1 | 1 | 74 |

> *PLENGTH SHORT*

> *!! Gender breakdown*

> *XTAB*

> *PLENGTH NONE / FREQ*

> *TABULATE geschlecht*

[▼One-Way Frequency Distribution](file:///\\Untitled.syo)

**Counts**

| **Values for GESCHLECHT** | | |
| --- | --- | --- |
| **1** | **2** | **Total** |
| 48 | 26 | 74 |

> *PLENGTH SHORT*

> *!! Condition breakdown*

> *XTAB*

> *PLENGTH NONE / FREQ*

> *TABULATE CONDITION*

[▼One-Way Frequency Distribution](file:///\\Untitled.syo)

**Counts**

| **Values for CONDITION** | | | |
| --- | --- | --- | --- |
| **KG** | **FB** | **FB + IV** | **Total** |
| 27 | 25 | 22 | 74 |

> *PLENGTH SHORT*

> *!! Age descriptives*

> *cstats alter /mean sd*

[▼Descriptive Statistics](file:///\\Untitled.syo)

|  | **ALTER** |
| --- | --- |
| Arithmetic Mean | 22.90540541 |
| Standard Deviation | 3.67579614 |

> *!! Calculating motive total scores from picture scores*

> *let afft1 = sum (pse_1_afft1 .. pse_6_afft1)*

> *let acht1 = sum (pse_1_acht1 .. pse_6_acht1)*

> *let powt1 = sum (pse_1_indt1 .. pse_6_indt1)*

> *let afft2 = sum (pse_s1_aff_t2 .. pse_s6_aff_t2)*

> *let acht2 = sum (pse_s1_ach_t2 .. pse_s6_ach_t2)*

> *let powt2 = sum (pse_s1_pow_t2 .. pse_s6_pow_t2)*

> *!! Motive score descriptives*

> *cstats afft1 .. powt2 wordcount_totalt1 wordcount_total_t2 /mean sd*

[▼Descriptive Statistics](file:///\\Untitled.syo)

|  | **AFFT1** | **ACHT1** | **POWT1** | **AFFT2** | **ACHT2** | **POWT2** | **WORDCOUNT_TOTAL- T1** | **WORDCOUNT_TOTAL- _T2** |
| --- | --- | --- | --- | --- | --- | --- | --- | --- |
| Arithmetic Mean | 6.24324324 | 4.79729730 | 5.87837838 | 6.05405405 | 5.02702703 | 5.51351351 | 588.13513514 | 591.33783784 |
| Standard Deviation | 2.89454286 | 2.32897020 | 2.71451049 | 3.08283756 | 2.58007631 | 3.05763470 | 155.41789220 | 178.49141624 |

> *!! Motive score correlations*

> *corr*

> *pearson afft1 .. powt2 * wordcount_totalt1 wordcount_total_t2 /prob*

[▼Correlation: Pearson](file:///\\Untitled.syo)

Number of Non-Missing Cases: 74

| **Pearson Correlation Matrix** | | |
| --- | --- | --- |
|  | **WORDCOUNT_TOTAL- T1** | **WORDCOUNT_TOTAL- _T2** |
| AFFT1 | 0.40635036 | 0.34465696 |
| ACHT1 | 0.46440096 | 0.43972798 |
| POWT1 | 0.50465845 | 0.30769365 |
| AFFT2 | 0.35816941 | 0.49330720 |
| ACHT2 | 0.30348617 | 0.24041609 |
| POWT2 | 0.41685479 | 0.56723960 |

| Bartlett Chi-Square Statistic | : | 244.58259570 |
| --- | --- | --- |
| df | : | 12 |
| p-Value | : | 0.00000000 |

| **Matrix of Probabilities** | | |
| --- | --- | --- |
|  | **WORDCOUNT_TOTAL- T1** | **WORDCOUNT_TOTAL- _T2** |
| AFFT1 | 0.00032768 | 0.00263760 |
| ACHT1 | 0.00003073 | 0.00008856 |
| POWT1 | 0.00000454 | 0.00765655 |
| AFFT2 | 0.00172861 | 0.00000798 |
| ACHT2 | 0.00857502 | 0.03908452 |
| POWT2 | 0.00022027 | 0.00000014 |

> *!! Correction for word count that preserves participants' absolute scores*

> *let cat1 = (acht1*1000) / wordcount_totalt1*

> *let cat2 = (acht2*1000) / wordcount_total_t2*

> *let cpt1 = (powt1*1000) / wordcount_totalt1*

> *let cpt2 = (powt2*1000) / wordcount_total_t2*

> *let cit1 = (afft1*1000) / wordcount_totalt1*

> *let cit2 = (afft2*1000) / wordcount_total_t2*

> *cstats cat1 cat2 cpt1 cpt2 cit1 cit2 /mean median sd*

[▼Descriptive Statistics](file:///\\Untitled.syo)

|  | **CAT1** | **CAT2** | **CPT1** | **CPT2** | **CIT1** | **CIT2** |
| --- | --- | --- | --- | --- | --- | --- |
| Median | 8.29548508 | 7.68939429 | 9.71737009 | 9.47888589 | 10.28284430 | 10.73552462 |
| Arithmetic Mean | 8.28784544 | 8.89398305 | 10.02356219 | 9.25674348 | 10.87390467 | 10.42541606 |
| Standard Deviation | 3.47987508 | 4.38308775 | 3.93039331 | 4.14625447 | 4.47767820 | 4.78185373 |

> *corr*

> *pearson cat1 .. cit2 * wordcount_totalt1 wordcount_total_t2 /prob*

[▼Correlation: Pearson](file:///\\Untitled.syo)

Number of Non-Missing Cases: 74

| **Pearson Correlation Matrix** | | |
| --- | --- | --- |
|  | **WORDCOUNT_TOTAL- T1** | **WORDCOUNT_TOTAL- _T2** |
| CAT1 | -0.14446487 | -0.05579648 |
| CAT2 | -0.14700026 | -0.30102398 |
| CPT1 | -0.02793024 | -0.15227382 |
| CPT2 | 0.00383900 | 0.05431102 |
| CIT1 | -0.22153034 | -0.15111898 |
| CIT2 | -0.13762598 | -0.13169930 |

| Bartlett Chi-Square Statistic | : | 155.44726222 |
| --- | --- | --- |
| df | : | 12 |
| p-Value | : | 0.00000000 |

| **Matrix of Probabilities** | | |
| --- | --- | --- |
|  | **WORDCOUNT_TOTAL- T1** | **WORDCOUNT_TOTAL- _T2** |
| CAT1 | 0.21943544 | 0.63680114 |
| CAT2 | 0.21136596 | 0.00915607 |
| CPT1 | 0.81326148 | 0.19525951 |
| CPT2 | 0.97410347 | 0.64581301 |
| CIT1 | 0.05784456 | 0.19870895 |
| CIT2 | 0.24227387 | 0.26334796 |

> *FITDIST*

> *CONTINUOUS cat1 .. cit2 / DISTRIBUTION = {Z}*

[▼Fitting Continuous Distribution](file:///\\Untitled.syo)

| Variable Name | : | CAT1 |
| --- | --- | --- |

| Distribution | : | Normal |
| --- | --- | --- |

**Estimated Parameter(s)**

| Location or Mean (mu) | : | 8.28784544 |
| --- | --- | --- |
| Scale or SD (sigma) | : | 3.45628243 |

Estimation of Parameter(s): Maximum Likelihood Method

**Test Results**

| **Lower Limit** | **Upper Limit** | **Observed** | **Expected** |
| --- | --- | --- | --- |
| . | 4.74741543 | 12 | 11.30985479 |
| 4.74741543 | 6.48499084 | 13 | 10.96182305 |
| 6.48499084 | 8.22256624 | 11 | 14.17077500 |
| 8.22256624 | 9.96014164 | 15 | 14.30313306 |
| 9.96014164 | 11.69771704 | 11 | 11.27187219 |
| 11.69771704 | 13.43529244 | 6 | 6.93544728 |
| 13.43529244 | . | 6 | 5.04709463 |
|  |  | 74 | 74.00000000 |

| Chi-Square Test Statistic | : | 1.47714882 |
| --- | --- | --- |
| Degrees of Freedom | : | 4 |
| p-Value | : | 0.83068141 |

| Kolmogorov-Smirnov Test Statistic | : | 0.08500304 |
| --- | --- | --- |
| Lilliefors Probability | : | 0.19299288 |

| Shapiro-Wilk Test Statistic | : | 0.98172367 |
| --- | --- | --- |
| p-Value | : | 0.36112018 |

| Variable Name | : | CAT2 |
| --- | --- | --- |

| Distribution | : | Normal |
| --- | --- | --- |

**Estimated Parameter(s)**

| Location or Mean (mu) | : | 8.89398305 |
| --- | --- | --- |
| Scale or SD (sigma) | : | 4.35337156 |

Estimation of Parameter(s): Maximum Likelihood Method

**Test Results**

| **Lower Limit** | **Upper Limit** | **Observed** | **Expected** |
| --- | --- | --- | --- |
| . | 4.63576159 | 8 | 12.13612683 |
| 4.63576159 | 6.95364238 | 21 | 12.12872609 |
| 6.95364238 | 9.27152318 | 15 | 15.29217260 |
| 9.27152318 | 11.58940397 | 13 | 14.61789840 |
| 11.58940397 | 13.90728477 | 9 | 10.59397015 |
| 13.90728477 | . | 8 | 9.23110594 |
|  |  | 74 | 74.00000000 |

| Chi-Square Test Statistic | : | 8.48698985 |
| --- | --- | --- |
| Degrees of Freedom | : | 3 |
| p-Value | : | 0.03694958 |

| Kolmogorov-Smirnov Test Statistic | : | 0.12279013 |
| --- | --- | --- |
| Lilliefors Probability | : | 0.00759084 |

| Shapiro-Wilk Test Statistic | : | 0.94556952 |
| --- | --- | --- |
| p-Value | : | 0.00310799 |

| Variable Name | : | CPT1 |
| --- | --- | --- |

| Distribution | : | Normal |
| --- | --- | --- |

**Estimated Parameter(s)**

| Location or Mean (mu) | : | 10.02356219 |
| --- | --- | --- |
| Scale or SD (sigma) | : | 3.90374627 |

Estimation of Parameter(s): Maximum Likelihood Method

**Test Results**

| **Lower Limit** | **Upper Limit** | **Observed** | **Expected** |
| --- | --- | --- | --- |
| . | 5.11569913 | 8 | 7.72095395 |
| 5.11569913 | 6.80398348 | 6 | 7.43124089 |
| 6.80398348 | 8.49226783 | 11 | 10.55777548 |
| 8.49226783 | 10.18055218 | 15 | 12.47693239 |
| 10.18055218 | 11.86883653 | 11 | 12.26511055 |
| 11.86883653 | 13.55712088 | 8 | 10.02912294 |
| 13.55712088 | 15.24540523 | 5 | 6.82146342 |
| 15.24540523 | . | 10 | 6.69740038 |
|  |  | 74 | 74.00000000 |

| Chi-Square Test Statistic | : | 3.47043713 |
| --- | --- | --- |
| Degrees of Freedom | : | 5 |
| p-Value | : | 0.62786551 |

| Kolmogorov-Smirnov Test Statistic | : | 0.06907336 |
| --- | --- | --- |
| Lilliefors Probability | : | 0.47627094 |

| Shapiro-Wilk Test Statistic | : | 0.98655996 |
| --- | --- | --- |
| p-Value | : | 0.62364031 |

| Variable Name | : | CPT2 |
| --- | --- | --- |

| Distribution | : | Normal |
| --- | --- | --- |

**Estimated Parameter(s)**

| Location or Mean (mu) | : | 9.25674348 |
| --- | --- | --- |
| Scale or SD (sigma) | : | 4.11814395 |

Estimation of Parameter(s): Maximum Likelihood Method

**Test Results**

| **Lower Limit** | **Upper Limit** | **Observed** | **Expected** |
| --- | --- | --- | --- |
| . | 3.70994941 | 9 | 6.58626938 |
| 3.70994941 | 5.56492411 | 5 | 7.10366823 |
| 5.56492411 | 7.41989882 | 9 | 10.56617103 |
| 7.41989882 | 9.27487352 | 13 | 12.87385975 |
| 9.27487352 | 11.12984823 | 17 | 12.84878335 |
| 11.12984823 | 12.98482293 | 7 | 10.50454574 |
| 12.98482293 | 14.83979764 | 4 | 7.03474796 |
| 14.83979764 | . | 10 | 6.48195455 |
|  |  | 74 | 74.00000000 |

| Chi-Square Test Statistic | : | 7.46989071 |
| --- | --- | --- |
| Degrees of Freedom | : | 5 |
| p-Value | : | 0.18797268 |

| Kolmogorov-Smirnov Test Statistic | : | 0.07647032 |
| --- | --- | --- |
| Lilliefors Probability | : | 0.32392327 |

| Shapiro-Wilk Test Statistic | : | 0.98582863 |
| --- | --- | --- |
| p-Value | : | 0.57905183 |

| Variable Name | : | CIT1 |
| --- | --- | --- |

| Distribution | : | Normal |
| --- | --- | --- |

**Estimated Parameter(s)**

| Location or Mean (mu) | : | 10.87390467 |
| --- | --- | --- |
| Scale or SD (sigma) | : | 4.44732071 |

Estimation of Parameter(s): Maximum Likelihood Method

**Test Results**

| **Lower Limit** | **Upper Limit** | **Observed** | **Expected** |
| --- | --- | --- | --- |
| . | 4.88879815 | 3 | 6.59984213 |
| 4.88879815 | 6.99595514 | 15 | 7.57938580 |
| 6.99595514 | 9.10311212 | 11 | 11.36943498 |
| 9.10311212 | 11.21026910 | 15 | 13.68202856 |
| 11.21026910 | 13.31742609 | 9 | 13.20919530 |
| 13.31742609 | 15.42458307 | 9 | 10.23092870 |
| 15.42458307 | . | 12 | 11.32918453 |
|  |  | 74 | 74.00000000 |

| Chi-Square Test Statistic | : | 10.89674796 |
| --- | --- | --- |
| Degrees of Freedom | : | 4 |
| p-Value | : | 0.02774926 |

| Kolmogorov-Smirnov Test Statistic | : | 0.09340525 |
| --- | --- | --- |
| Lilliefors Probability | : | 0.10735135 |

| Shapiro-Wilk Test Statistic | : | 0.95724238 |
| --- | --- | --- |
| p-Value | : | 0.01356632 |

| Variable Name | : | CIT2 |
| --- | --- | --- |

| Distribution | : | Normal |
| --- | --- | --- |

**Estimated Parameter(s)**

| Location or Mean (mu) | : | 10.42541606 |
| --- | --- | --- |
| Scale or SD (sigma) | : | 4.74943401 |

Estimation of Parameter(s): Maximum Likelihood Method

**Test Results**

| **Lower Limit** | **Upper Limit** | **Observed** | **Expected** |
| --- | --- | --- | --- |
| . | 4.17117471 | 7 | 6.95202239 |
| 4.17117471 | 6.25029921 | 7 | 7.08428582 |
| 6.25029921 | 8.32942370 | 14 | 10.34615883 |
| 8.32942370 | 10.40854820 | 8 | 12.51268528 |
| 10.40854820 | 12.48767270 | 17 | 12.53184521 |
| 12.48767270 | 14.56679720 | 9 | 10.39375994 |
| 14.56679720 | 16.64592170 | 4 | 7.13869408 |
| 16.64592170 | . | 8 | 7.04054846 |
|  |  | 74 | 74.00000000 |

| Chi-Square Test Statistic | : | 6.20995732 |
| --- | --- | --- |
| Degrees of Freedom | : | 5 |
| p-Value | : | 0.28632198 |

| Kolmogorov-Smirnov Test Statistic | : | 0.07185984 |
| --- | --- | --- |
| Lilliefors Probability | : | 0.41475626 |

| Shapiro-Wilk Test Statistic | : | 0.97274883 |
| --- | --- | --- |
| p-Value | : | 0.10992085 |

> *!! Calculating personal goal commitment scores*

> *cronbach ach_4_T1, ach_7_T1, ach_10_T1, ach_13_T1*

[▼Cronbach's Alpha](file:///\\Untitled.syo)

| Number of Variables | 4 |  |  |  |
| --- | --- | --- | --- | --- |
| Number of Cases | 74 |  |  |  |
| Variables | ACH_4_T1 | ACH_7_T1 | ACH_10_T1 | ACH_13_T1 |
| Cronbach's Alpha | 0.77911706 |  |  |  |

> *cronbach aff_4_T1, aff_7_T1, aff_10_T1, aff_13_T1*

[▼Cronbach's Alpha](file:///\\Untitled.syo)

| Number of Variables | 4 |  |  |  |
| --- | --- | --- | --- | --- |
| Number of Cases | 74 |  |  |  |
| Variables | AFF_4_T1 | AFF_7_T1 | AFF_10_T1 | AFF_13_T1 |
| Cronbach's Alpha | 0.73956857 |  |  |  |

> *cronbach ind_4_T1, ind_7_T1, ind_10_T1, ind_13_T1*

[▼Cronbach's Alpha](file:///\\Untitled.syo)

| Number of Variables | 4 |  |  |  |
| --- | --- | --- | --- | --- |
| Number of Cases | 74 |  |  |  |
| Variables | IND_4_T1 | IND_7_T1 | IND_10_T1 | IND_13_T1 |
| Cronbach's Alpha | 0.83549279 |  |  |  |

> *cronbach ach_4_T2, ach_7_T2, ach_10_T2, ach_13_T2*

[▼Cronbach's Alpha](file:///\\Untitled.syo)

| Number of Variables | 4 |  |  |  |
| --- | --- | --- | --- | --- |
| Number of Cases | 74 |  |  |  |
| Variables | ACH_4_T2 | ACH_7_T2 | ACH_10_T2 | ACH_13_T2 |
| Cronbach's Alpha | 0.72111606 |  |  |  |

> *cronbach aff_4_T2, aff_7_T2, aff_10_T2, aff_13_T2*

[▼Cronbach's Alpha](file:///\\Untitled.syo)

| Number of Variables | 4 |  |  |  |
| --- | --- | --- | --- | --- |
| Number of Cases | 74 |  |  |  |
| Variables | AFF_4_T2 | AFF_7_T2 | AFF_10_T2 | AFF_13_T2 |
| Cronbach's Alpha | 0.74676385 |  |  |  |

> *cronbach ind_4_T2, ind_7_T2, ind_10_T2, ind_13_T2*

[▼Cronbach's Alpha](file:///\\Untitled.syo)

| Number of Variables | 4 |  |  |  |
| --- | --- | --- | --- | --- |
| Number of Cases | 74 |  |  |  |
| Variables | IND_4_T2 | IND_7_T2 | IND_10_T2 | IND_13_T2 |
| Cronbach's Alpha | 0.87178022 |  |  |  |

> *Let PGQ_ach_C1 = avg (ach_4_T1, ach_7_T1, ach_10_T1, ach_13_T1)*

> *Let PGQ_AFF_C1 = avg (aff_4_T1, aff_7_T1, aff_10_T1, aff_13_T1)*

> *Let PGQ_IND_C1 = avg (ind_4_T1, ind_7_T1, ind_10_T1, ind_13_T1)*

> *cstats PGQ_ach_C1 PGQ_AFF_C1 PGQ_IND_C1 /mean median sd*

[▼Descriptive Statistics](file:///\\Untitled.syo)

|  | **PGQ_ACH_C1** | **PGQ_AFF_C1** | **PGQ_IND_C1** |
| --- | --- | --- | --- |
| Median | 4.25000000 | 4.25000000 | 4.25000000 |
| Arithmetic Mean | 4.20608108 | 3.93243243 | 4.06756757 |
| Standard Deviation | 0.68790693 | 0.76670584 | 0.79736005 |

> *Let PGQ_ach_C2 = avg (ach_4_T2, ach_7_T2, ach_10_T2, ach_13_T2)*

> *Let PGQ_AFF_C2 = avg (aff_4_T2, aff_7_T2, aff_10_T2, aff_13_T2)*

> *Let PGQ_IND_C2 = avg (ind_4_T2, ind_7_T2, ind_10_T2, ind_13_T2)*

> *cstats PGQ_ach_C2 PGQ_AFF_C2 PGQ_IND_C2 /mean median sd*

[▼Descriptive Statistics](file:///\\Untitled.syo)

|  | **PGQ_ACH_C2** | **PGQ_AFF_C2** | **PGQ_IND_C2** |
| --- | --- | --- | --- |
| Median | 4.37500000 | 4.25000000 | 4.12500000 |
| Arithmetic Mean | 4.21959459 | 4.03378378 | 3.96959459 |
| Standard Deviation | 0.61787404 | 0.63747727 | 0.82962351 |

> *FITDIST*

> *CONTINUOUS PGQ_ach_C1 PGQ_AFF_C1 PGQ_IND_C1 PGQ_ach_C2 PGQ_AFF_C2 PGQ_IND_C2/ DISTRIBUTION = {Z}*

[▼Fitting Continuous Distribution](file:///\\Untitled.syo)

| Variable Name | : | PGQ_ACH_C1 |
| --- | --- | --- |

| Distribution | : | Normal |
| --- | --- | --- |

**Estimated Parameter(s)**

| Location or Mean (mu) | : | 4.20608108 |
| --- | --- | --- |
| Scale or SD (sigma) | : | 0.68324310 |

Estimation of Parameter(s): Maximum Likelihood Method

**Test Results**

| **Lower Limit** | **Upper Limit** | **Observed** | **Expected** |
| --- | --- | --- | --- |
| . | 3.25000000 | 10 | 5.98344379 |
| 3.25000000 | 3.50000000 | 6 | 5.16852833 |
| 3.50000000 | 3.75000000 | 6 | 7.51221771 |
| 3.75000000 | 4.00000000 | 7 | 9.56460168 |
| 4.00000000 | 4.25000000 | 10 | 10.66756147 |
| 4.25000000 | 4.50000000 | 11 | 10.42230807 |
| 4.50000000 | 4.75000000 | 7 | 8.91996075 |
| 4.75000000 | 5.00000000 | 17 | 6.68745573 |
| 5.00000000 | . | 0 | 9.07392247 |
|  |  | 74 | 74.00000000 |

| Chi-Square Test Statistic | : | 29.28572918 |
| --- | --- | --- |
| Degrees of Freedom | : | 6 |
| p-Value | : | 0.00005371 |

| Kolmogorov-Smirnov Test Statistic | : | 0.13944137 |
| --- | --- | --- |
| Lilliefors Probability | : | 0.00111967 |

| Shapiro-Wilk Test Statistic | : | 0.91256323 |
| --- | --- | --- |
| p-Value | : | 0.00008157 |

| Variable Name | : | PGQ_AFF_C1 |
| --- | --- | --- |

| Distribution | : | Normal |
| --- | --- | --- |

**Estimated Parameter(s)**

| Location or Mean (mu) | : | 3.93243243 |
| --- | --- | --- |
| Scale or SD (sigma) | : | 0.76150777 |

Estimation of Parameter(s): Maximum Likelihood Method

**Test Results**

| **Lower Limit** | **Upper Limit** | **Observed** | **Expected** |
| --- | --- | --- | --- |
| . | 2.90000000 | 9 | 6.48133024 |
| 2.90000000 | 3.25000000 | 9 | 7.21487005 |
| 3.25000000 | 3.60000000 | 4 | 10.81412912 |
| 3.60000000 | 3.95000000 | 7 | 13.17066032 |
| 3.95000000 | 4.30000000 | 22 | 13.03416395 |
| 4.30000000 | 4.65000000 | 8 | 10.48137087 |
| 4.65000000 | 5.00000000 | 15 | 6.84864894 |
| 5.00000000 | . | 0 | 5.95482652 |
|  |  | 74 | 74.00000000 |

| Chi-Square Test Statistic | : | 31.01663432 |
| --- | --- | --- |
| Degrees of Freedom | : | 5 |
| p-Value | : | 0.00000930 |

| Kolmogorov-Smirnov Test Statistic | : | 0.17518335 |
| --- | --- | --- |
| Lilliefors Probability | : | 0.00000671 |

| Shapiro-Wilk Test Statistic | : | 0.92881862 |
| --- | --- | --- |
| p-Value | : | 0.00044772 |

| Variable Name | : | PGQ_IND_C1 |
| --- | --- | --- |

| Distribution | : | Normal |
| --- | --- | --- |

**Estimated Parameter(s)**

| Location or Mean (mu) | : | 4.06756757 |
| --- | --- | --- |
| Scale or SD (sigma) | : | 0.79195415 |

Estimation of Parameter(s): Maximum Likelihood Method

**Test Results**

| **Lower Limit** | **Upper Limit** | **Observed** | **Expected** |
| --- | --- | --- | --- |
| . | 2.90000000 | 7 | 5.19496217 |
| 2.90000000 | 3.50000000 | 6 | 12.32747813 |
| 3.50000000 | 3.80000000 | 12 | 9.68997393 |
| 3.80000000 | 4.10000000 | 6 | 10.99623391 |
| 4.10000000 | 4.40000000 | 14 | 10.82896266 |
| 4.40000000 | 4.70000000 | 7 | 9.25446049 |
| 4.70000000 | 5.00000000 | 22 | 6.86333262 |
| 5.00000000 | . | 0 | 8.84459609 |
|  |  | 74 | 74.00000000 |

| Chi-Square Test Statistic | : | 50.40112068 |
| --- | --- | --- |
| Degrees of Freedom | : | 5 |
| p-Value | : | 0.00000000 |

| Kolmogorov-Smirnov Test Statistic | : | 0.17217402 |
| --- | --- | --- |
| Lilliefors Probability | : | 0.00001089 |

| Shapiro-Wilk Test Statistic | : | 0.90990710 |
| --- | --- | --- |
| p-Value | : | 0.00006267 |

| Variable Name | : | PGQ_ACH_C2 |
| --- | --- | --- |

| Distribution | : | Normal |
| --- | --- | --- |

**Estimated Parameter(s)**

| Location or Mean (mu) | : | 4.21959459 |
| --- | --- | --- |
| Scale or SD (sigma) | : | 0.61368501 |

Estimation of Parameter(s): Maximum Likelihood Method

**Test Results**

| **Lower Limit** | **Upper Limit** | **Observed** | **Expected** |
| --- | --- | --- | --- |
| . | 3.50000000 | 14 | 8.91568243 |
| 3.50000000 | 3.75000000 | 4 | 7.51785726 |
| 3.75000000 | 4.00000000 | 9 | 10.22388438 |
| 4.00000000 | 4.25000000 | 10 | 11.80465013 |
| 4.25000000 | 4.50000000 | 14 | 11.57198900 |
| 4.50000000 | 4.75000000 | 15 | 9.63119725 |
| 4.75000000 | 5.00000000 | 8 | 6.80560750 |
| 5.00000000 | . | 0 | 7.52913205 |
|  |  | 74 | 74.00000000 |

| Chi-Square Test Statistic | : | 16.20890645 |
| --- | --- | --- |
| Degrees of Freedom | : | 5 |
| p-Value | : | 0.00627227 |

| Kolmogorov-Smirnov Test Statistic | : | 0.17613599 |
| --- | --- | --- |
| Lilliefors Probability | : | 0.00000575 |

| Shapiro-Wilk Test Statistic | : | 0.91207262 |
| --- | --- | --- |
| p-Value | : | 0.00007767 |

| Variable Name | : | PGQ_AFF_C2 |
| --- | --- | --- |

| Distribution | : | Normal |
| --- | --- | --- |

**Estimated Parameter(s)**

| Location or Mean (mu) | : | 4.03378378 |
| --- | --- | --- |
| Scale or SD (sigma) | : | 0.63315534 |

Estimation of Parameter(s): Maximum Likelihood Method

**Test Results**

| **Lower Limit** | **Upper Limit** | **Observed** | **Expected** |
| --- | --- | --- | --- |
| . | 3.25000000 | 13 | 7.98284916 |
| 3.25000000 | 3.50000000 | 5 | 6.78749753 |
| 3.50000000 | 3.75000000 | 11 | 9.42783106 |
| 3.75000000 | 4.00000000 | 6 | 11.22735452 |
| 4.00000000 | 4.25000000 | 15 | 11.46328264 |
| 4.25000000 | 4.50000000 | 9 | 10.03475488 |
| 4.50000000 | 4.75000000 | 10 | 7.53128226 |
| 4.75000000 | . | 5 | 9.54514796 |
|  |  | 74 | 74.00000000 |

| Chi-Square Test Statistic | : | 10.49133915 |
| --- | --- | --- |
| Degrees of Freedom | : | 5 |
| p-Value | : | 0.06245188 |

| Kolmogorov-Smirnov Test Statistic | : | 0.16065966 |
| --- | --- | --- |
| Lilliefors Probability | : | 0.00006339 |

| Shapiro-Wilk Test Statistic | : | 0.94990831 |
| --- | --- | --- |
| p-Value | : | 0.00531014 |

| Variable Name | : | PGQ_IND_C2 |
| --- | --- | --- |

| Distribution | : | Normal |
| --- | --- | --- |

**Estimated Parameter(s)**

| Location or Mean (mu) | : | 3.96959459 |
| --- | --- | --- |
| Scale or SD (sigma) | : | 0.82399888 |

Estimation of Parameter(s): Maximum Likelihood Method

**Test Results**

| **Lower Limit** | **Upper Limit** | **Observed** | **Expected** |
| --- | --- | --- | --- |
| . | 3.05000000 | 10 | 9.78339140 |
| 3.05000000 | 3.37500000 | 8 | 7.62668770 |
| 3.37500000 | 3.70000000 | 6 | 10.10064173 |
| 3.70000000 | 4.02500000 | 13 | 11.47281544 |
| 4.02500000 | 4.35000000 | 12 | 11.17637101 |
| 4.35000000 | 4.67500000 | 6 | 9.33772372 |
| 4.67500000 | 5.00000000 | 19 | 6.69095644 |
| 5.00000000 | . | 0 | 7.81141257 |
|  |  | 74 | 74.00000000 |

| Chi-Square Test Statistic | : | 33.60067001 |
| --- | --- | --- |
| Degrees of Freedom | : | 5 |
| p-Value | : | 0.00000286 |

| Kolmogorov-Smirnov Test Statistic | : | 0.13318405 |
| --- | --- | --- |
| Lilliefors Probability | : | 0.00238041 |

| Shapiro-Wilk Test Statistic | : | 0.92872111 |
| --- | --- | --- |
| p-Value | : | 0.00044295 |

> *!! Calculating congruence indices based on motive medians from initial assessment (BEFORE recoding) -- these are the scores based on which participants received
feedback/training*

> *!! Because subjects 103 and 203 each switched 1 congruence category based on their recoded motive scores, they were removed from the sample*

> *LET iemach1 = 0*

> *IF (cat1 > 8.066895 and PGQ_ach_C1 >= 4)then LET iemach1 = 1*

> *IF (cat1 < 2 and PGQ_ach_C1 < 4)then LET iemach1 = 1*

> *LET iemach2 = 0*

> *IF (cat2 > 8.066895 and PGQ_ACH_C2 >= 4)then LET iemach2 = 1*

> *IF (cat2 < 2 and PGQ_ACH_C2 < 4)then LET iemach2 = 1*

> *LET iemaff1 = 0*

> *IF (cit1 > 10.282844 and pgq_aff_C1 >= 4)then LET iemaff1 = 1*

> *IF (cit1 < 2 and pgq_aff_C1 < 4)then LET iemaff1 = 1*

> *LET iemaff2 = 0*

> *IF (cit2 > 10.282844 and PGQ_AFF_C2 >= 4)then LET iemaff2 = 1*

> *IF (cit2 < 2 and PGQ_AFF_C2 < 4)then LET iemaff2 = 1*

> *LET iemind1 = 0*

> *IF (cpt1 > 9.803922 and pgq_ind_C1 >= 4)then LET iemind1 = 1*

> *IF (cpt1 < 2 and pgq_ind_C1 < 4)then LET iemind1 = 1*

> *LET iemind2 = 0*

> *IF (cpt2 > 9.803922 and PGQ_IND_C2 >= 4)then LET iemind2 = 1*

> *IF (cpt2 < 2 and PGQ_IND_C2 < 4)then LET iemind2 = 1*

> *!! Calculating overall congruence scores*

> *LET kongruenz1 = Sum (iemach1, iemaff1, iemind1)*

> *LET kongruenz2 = Sum (iemach2, iemaff2, iemind2)*

> *!!Agentic congruence*

> *LET agkon1 = Sum (iemach1, iemind1)*

> *LET agkon2 = Sum (iemach2, iemind2)*

> *cstats iemach1 iemach2 iemaff1 iemaff2 iemind1 iemind2 kongruenz1 kongruenz2 /mean sd*

[▼Descriptive Statistics](file:///\\Untitled.syo)

|  | **IEMACH1** | **IEMACH2** | **IEMAFF1** | **IEMAFF2** | **IEMIND1** | **IEMIND2** | **KONGRUENZ1** | **KONGRUENZ2** |
| --- | --- | --- | --- | --- | --- | --- | --- | --- |
| Arithmetic Mean | 0.40540541 | 0.39189189 | 0.29729730 | 0.31081081 | 0.31081081 | 0.31081081 | 1.01351351 | 1.01351351 |
| Standard Deviation | 0.49432170 | 0.49150503 | 0.46018846 | 0.46598476 | 0.46598476 | 0.46598476 | 0.95792646 | 0.94351780 |

> *corr*

> *pearson iemach1 iemach2 iemaff1 iemaff2 iemind1 iemind2 kongruenz1 kongruenz2/prob*

[▼Correlation: Pearson](file:///\\Untitled.syo)

Number of Non-Missing Cases: 74

| **Pearson Correlation Matrix** | | | | | | | | |
| --- | --- | --- | --- | --- | --- | --- | --- | --- |
|  | **IEMACH1** | **IEMACH2** | **IEMAFF1** | **IEMAFF2** | **IEMIND1** | **IEMIND2** | **KONGRUENZ1** | **KONGRUENZ2** |
| IEMACH1 | 1.00000000 |  |  |  |  |  |  |  |
| IEMACH2 | 0.29562387 | 1.00000000 |  |  |  |  |  |  |
| IEMAFF1 | 0.42641386 | 0.20460759 | 1.00000000 |  |  |  |  |  |
| IEMAFF2 | 0.21859133 | 0.23843376 | 0.26588185 | 1.00000000 |  |  |  |  |
| IEMIND1 | 0.04018223 | 0.11881276 | 0.07423974 | 0.11679454 | 1.00000000 |  |  |  |
| IEMIND2 | 0.09965193 | 0.17862326 | 0.07423974 | 0.05370844 | 0.11679454 | 1.00000000 |  |  |
| KONGRUENZ1 | 0.74042922 | 0.30864194 | 0.73655828 | 0.29734503 | 0.54285167 | 0.14390338 | 1.00000000 |  |
| KONGRUENZ2 | 0.31117286 | 0.72690441 | 0.27456518 | 0.64461261 | 0.17725794 | 0.61345563 | 0.37870415 | 1.00000000 |

WARNING Correlation matrix is not positive definite.

Individual significance tests are suspect.

| **Matrix of Probabilities** | | | | | | | | |
| --- | --- | --- | --- | --- | --- | --- | --- | --- |
|  | **IEMACH1** | **IEMACH2** | **IEMAFF1** | **IEMAFF2** | **IEMIND1** | **IEMIND2** | **KONGRUENZ1** | **KONGRUENZ2** |
| IEMACH1 | 0.00000000 |  |  |  |  |  |  |  |
| IEMACH2 | 0.01055218 | 0.00000000 |  |  |  |  |  |  |
| IEMAFF1 | 0.00015171 | 0.08034509 | 0.00000000 |  |  |  |  |  |
| IEMAFF2 | 0.06133440 | 0.04077874 | 0.02204198 | 0.00000000 |  |  |  |  |
| IEMIND1 | 0.73392192 | 0.31333792 | 0.52959118 | 0.32168725 | 0.00000000 |  |  |  |
| IEMIND2 | 0.39825067 | 0.12783618 | 0.52959118 | 0.64948337 | 0.32168725 | 0.00000000 |  |  |
| KONGRUENZ1 | 0.00000000 | 0.00746188 | 0.00000000 | 0.01008833 | 0.00000058 | 0.22125140 | 0.00000000 |  |
| KONGRUENZ2 | 0.00696344 | 0.00000000 | 0.01791451 | 0.00000000 | 0.13082339 | 0.00000001 | 0.00087748 | 0.00000000 |

> *FITDIST*

> *CONTINUOUS iemach1 iemach2 iemaff1 iemaff2 iemind1 iemind2 kongruenz1 kongruenz2 agkon1 agkon2/ DISTRIBUTION = {Z}*

[▼Fitting Continuous Distribution](file:///\\Untitled.syo)

| Variable Name | : | IEMACH1 |
| --- | --- | --- |

| Distribution | : | Normal |
| --- | --- | --- |

**Estimated Parameter(s)**

| Location or Mean (mu) | : | 0.40540541 |
| --- | --- | --- |
| Scale or SD (sigma) | : | 0.49097033 |

Estimation of Parameter(s): Maximum Likelihood Method

**Test Results**

| **Lower Limit** | **Upper Limit** | **Observed** | **Expected** |
| --- | --- | --- | --- |
| . | 0.00000000 | 44 | 15.13156966 |
| 0.00000000 | 0.20000000 | 0 | 9.86856713 |
| 0.20000000 | 0.30000000 | 1 | 5.71025654 |
| 0.30000000 | 0.40000000 | 1 | 5.96458971 |
| 0.40000000 | 0.50000000 | 0 | 5.97793358 |
| 0.50000000 | 0.60000000 | 1 | 5.74866699 |
| 0.60000000 | 0.80000000 | 1 | 10.00039284 |
| 0.80000000 | 1.00000000 | 28 | 7.24074639 |
| 1.00000000 | . | 0 | 8.35727717 |
|  |  | 76 | 74.00000000 |

| Chi-Square Test Statistic | : | 158.83728584 |
| --- | --- | --- |
| Degrees of Freedom | : | 6 |
| p-Value | : | 0.00000000 |

| Kolmogorov-Smirnov Test Statistic | : | 0.39011392 |
| --- | --- | --- |
| Lilliefors Probability | : | 0.00000000 |

| Shapiro-Wilk Test Statistic | : | 0.62333604 |
| --- | --- | --- |
| p-Value | : | 0.00000000 |

| Variable Name | : | IEMACH2 |
| --- | --- | --- |

| Distribution | : | Normal |
| --- | --- | --- |

**Estimated Parameter(s)**

| Location or Mean (mu) | : | 0.39189189 |
| --- | --- | --- |
| Scale or SD (sigma) | : | 0.48817275 |

Estimation of Parameter(s): Maximum Likelihood Method

**Test Results**

| **Lower Limit** | **Upper Limit** | **Observed** | **Expected** |
| --- | --- | --- | --- |
| . | 0.00000000 | 45 | 15.61792078 |
| 0.00000000 | 0.20000000 | 0 | 10.06966089 |
| 0.20000000 | 0.30000000 | 1 | 5.78799728 |
| 0.30000000 | 0.40000000 | 0 | 6.01472773 |
| 0.40000000 | 0.50000000 | 1 | 5.99436982 |
| 0.50000000 | 0.60000000 | 1 | 5.72942446 |
| 0.60000000 | 0.80000000 | 1 | 9.86898370 |
| 0.80000000 | 1.00000000 | 26 | 7.04031493 |
| 1.00000000 | . | 0 | 7.87660041 |
|  |  | 75 | 74.00000000 |

| Chi-Square Test Statistic | : | 150.29263612 |
| --- | --- | --- |
| Degrees of Freedom | : | 6 |
| p-Value | : | 0.00000000 |

| Kolmogorov-Smirnov Test Statistic | : | 0.39705512 |
| --- | --- | --- |
| Lilliefors Probability | : | 0.00000000 |

| Shapiro-Wilk Test Statistic | : | 0.61925884 |
| --- | --- | --- |
| p-Value | : | 0.00000000 |

| Variable Name | : | IEMAFF1 |
| --- | --- | --- |

| Distribution | : | Normal |
| --- | --- | --- |

**Estimated Parameter(s)**

| Location or Mean (mu) | : | 0.29729730 |
| --- | --- | --- |
| Scale or SD (sigma) | : | 0.45706850 |

Estimation of Parameter(s): Maximum Likelihood Method

**Test Results**

| **Lower Limit** | **Upper Limit** | **Observed** | **Expected** |
| --- | --- | --- | --- |
| . | 0.00000000 | 52 | 19.07001086 |
| 0.00000000 | 0.10000000 | 0 | 5.57161771 |
| 0.10000000 | 0.20000000 | 1 | 6.12114977 |
| 0.20000000 | 0.30000000 | 0 | 6.41178628 |
| 0.30000000 | 0.40000000 | 1 | 6.40352967 |
| 0.40000000 | 0.50000000 | 1 | 6.09753314 |
| 0.50000000 | 0.70000000 | 1 | 10.32772406 |
| 0.70000000 | 0.90000000 | 1 | 7.06677190 |
| 0.90000000 | . | 18 | 6.92987660 |
|  |  | 75 | 74.00000000 |

| Chi-Square Test Statistic | : | 113.26925796 |
| --- | --- | --- |
| Degrees of Freedom | : | 6 |
| p-Value | : | 0.00000000 |

| Kolmogorov-Smirnov Test Statistic | : | 0.44499985 |
| --- | --- | --- |
| Lilliefors Probability | : | 0.00000000 |

| Shapiro-Wilk Test Statistic | : | 0.57378247 |
| --- | --- | --- |
| p-Value | : | 0.00000000 |

| Variable Name | : | IEMAFF2 |
| --- | --- | --- |

| Distribution | : | Normal |
| --- | --- | --- |

**Estimated Parameter(s)**

| Location or Mean (mu) | : | 0.31081081 |
| --- | --- | --- |
| Scale or SD (sigma) | : | 0.46282551 |

Estimation of Parameter(s): Maximum Likelihood Method

**Test Results**

| **Lower Limit** | **Upper Limit** | **Observed** | **Expected** |
| --- | --- | --- | --- |
| . | 0.00000000 | 51 | 18.56918072 |
| 0.00000000 | 0.10000000 | 0 | 5.43491024 |
| 0.10000000 | 0.20000000 | 1 | 5.99469629 |
| 0.20000000 | 0.30000000 | 1 | 6.31169845 |
| 0.30000000 | 0.40000000 | 0 | 6.34350905 |
| 0.40000000 | 0.50000000 | 1 | 6.08579269 |
| 0.50000000 | 0.60000000 | 1 | 5.57325605 |
| 0.60000000 | 0.80000000 | 1 | 8.93739393 |
| 0.80000000 | 1.00000000 | 20 | 5.70041422 |
| 1.00000000 | . | 0 | 5.04914836 |
|  |  | 76 | 74.00000000 |

| Chi-Square Test Statistic | : | 133.02202432 |
| --- | --- | --- |
| Degrees of Freedom | : | 7 |
| p-Value | : | 0.00000000 |

| Kolmogorov-Smirnov Test Statistic | : | 0.43825431 |
| --- | --- | --- |
| Lilliefors Probability | : | 0.00000000 |

| Shapiro-Wilk Test Statistic | : | 0.58221821 |
| --- | --- | --- |
| p-Value | : | 0.00000000 |

| Variable Name | : | IEMIND1 |
| --- | --- | --- |

| Distribution | : | Normal |
| --- | --- | --- |

**Estimated Parameter(s)**

| Location or Mean (mu) | : | 0.31081081 |
| --- | --- | --- |
| Scale or SD (sigma) | : | 0.46282551 |

Estimation of Parameter(s): Maximum Likelihood Method

**Test Results**

| **Lower Limit** | **Upper Limit** | **Observed** | **Expected** |
| --- | --- | --- | --- |
| . | 0.00000000 | 51 | 18.56918072 |
| 0.00000000 | 0.10000000 | 0 | 5.43491024 |
| 0.10000000 | 0.20000000 | 1 | 5.99469629 |
| 0.20000000 | 0.30000000 | 1 | 6.31169845 |
| 0.30000000 | 0.40000000 | 0 | 6.34350905 |
| 0.40000000 | 0.50000000 | 1 | 6.08579269 |
| 0.50000000 | 0.60000000 | 1 | 5.57325605 |
| 0.60000000 | 0.80000000 | 1 | 8.93739393 |
| 0.80000000 | 1.00000000 | 20 | 5.70041422 |
| 1.00000000 | . | 0 | 5.04914836 |
|  |  | 76 | 74.00000000 |

| Chi-Square Test Statistic | : | 133.02202432 |
| --- | --- | --- |
| Degrees of Freedom | : | 7 |
| p-Value | : | 0.00000000 |

| Kolmogorov-Smirnov Test Statistic | : | 0.43825431 |
| --- | --- | --- |
| Lilliefors Probability | : | 0.00000000 |

| Shapiro-Wilk Test Statistic | : | 0.58221821 |
| --- | --- | --- |
| p-Value | : | 0.00000000 |

| Variable Name | : | IEMIND2 |
| --- | --- | --- |

| Distribution | : | Normal |
| --- | --- | --- |

**Estimated Parameter(s)**

| Location or Mean (mu) | : | 0.31081081 |
| --- | --- | --- |
| Scale or SD (sigma) | : | 0.46282551 |

Estimation of Parameter(s): Maximum Likelihood Method

**Test Results**

| **Lower Limit** | **Upper Limit** | **Observed** | **Expected** |
| --- | --- | --- | --- |
| . | 0.00000000 | 51 | 18.56918072 |
| 0.00000000 | 0.10000000 | 0 | 5.43491024 |
| 0.10000000 | 0.20000000 | 1 | 5.99469629 |
| 0.20000000 | 0.30000000 | 1 | 6.31169845 |
| 0.30000000 | 0.40000000 | 0 | 6.34350905 |
| 0.40000000 | 0.50000000 | 1 | 6.08579269 |
| 0.50000000 | 0.60000000 | 1 | 5.57325605 |
| 0.60000000 | 0.80000000 | 1 | 8.93739393 |
| 0.80000000 | 1.00000000 | 20 | 5.70041422 |
| 1.00000000 | . | 0 | 5.04914836 |
|  |  | 76 | 74.00000000 |

| Chi-Square Test Statistic | : | 133.02202432 |
| --- | --- | --- |
| Degrees of Freedom | : | 7 |
| p-Value | : | 0.00000000 |

| Kolmogorov-Smirnov Test Statistic | : | 0.43825431 |
| --- | --- | --- |
| Lilliefors Probability | : | 0.00000000 |

| Shapiro-Wilk Test Statistic | : | 0.58221821 |
| --- | --- | --- |
| p-Value | : | 0.00000000 |

| Variable Name | : | KONGRUENZ1 |
| --- | --- | --- |

| Distribution | : | Normal |
| --- | --- | --- |

**Estimated Parameter(s)**

| Location or Mean (mu) | : | 1.01351351 |
| --- | --- | --- |
| Scale or SD (sigma) | : | 0.95143197 |

Estimation of Parameter(s): Maximum Likelihood Method

**Test Results**

| **Lower Limit** | **Upper Limit** | **Observed** | **Expected** |
| --- | --- | --- | --- |
| . | 0.00000000 | 28 | 10.61021334 |
| 0.00000000 | 0.30000000 | 0 | 6.16162691 |
| 0.30000000 | 0.60000000 | 1 | 7.79011519 |
| 0.60000000 | 0.90000000 | 1 | 8.92420224 |
| 0.90000000 | 1.20000000 | 22 | 9.26344334 |
| 1.20000000 | 1.50000000 | 1 | 8.71270783 |
| 1.50000000 | 1.80000000 | 1 | 7.42525357 |
| 1.80000000 | 2.40000000 | 17 | 9.74581585 |
| 2.40000000 | . | 5 | 5.36662172 |
|  |  | 76 | 74.00000000 |

| Chi-Square Test Statistic | : | 82.94150690 |
| --- | --- | --- |
| Degrees of Freedom | : | 6 |
| p-Value | : | 0.00000000 |

| Kolmogorov-Smirnov Test Statistic | : | 0.23499712 |
| --- | --- | --- |
| Lilliefors Probability | : | 0.00000000 |

| Shapiro-Wilk Test Statistic | : | 0.83756910 |
| --- | --- | --- |
| p-Value | : | 0.00000015 |

| Variable Name | : | KONGRUENZ2 |
| --- | --- | --- |

| Distribution | : | Normal |
| --- | --- | --- |

**Estimated Parameter(s)**

| Location or Mean (mu) | : | 1.01351351 |
| --- | --- | --- |
| Scale or SD (sigma) | : | 0.93712100 |

Estimation of Parameter(s): Maximum Likelihood Method

**Test Results**

| **Lower Limit** | **Upper Limit** | **Observed** | **Expected** |
| --- | --- | --- | --- |
| . | 0.00000000 | 28 | 10.34026501 |
| 0.00000000 | 0.30000000 | 0 | 6.17745344 |
| 0.30000000 | 0.60000000 | 1 | 7.86621265 |
| 0.60000000 | 0.90000000 | 1 | 9.04882592 |
| 0.90000000 | 1.20000000 | 21 | 9.40350314 |
| 1.20000000 | 1.50000000 | 1 | 8.82791524 |
| 1.50000000 | 1.80000000 | 1 | 7.48682065 |
| 1.80000000 | 2.40000000 | 19 | 9.70592330 |
| 2.40000000 | . | 4 | 5.14308065 |
|  |  | 76 | 74.00000000 |

| Chi-Square Test Statistic | : | 85.50676417 |
| --- | --- | --- |
| Degrees of Freedom | : | 6 |
| p-Value | : | 0.00000000 |

| Kolmogorov-Smirnov Test Statistic | : | 0.23864507 |
| --- | --- | --- |
| Lilliefors Probability | : | 0.00000000 |

| Shapiro-Wilk Test Statistic | : | 0.83400422 |
| --- | --- | --- |
| p-Value | : | 0.00000012 |

| Variable Name | : | AGKON1 |
| --- | --- | --- |

| Distribution | : | Normal |
| --- | --- | --- |

**Estimated Parameter(s)**

| Location or Mean (mu) | : | 0.71621622 |
| --- | --- | --- |
| Scale or SD (sigma) | : | 0.68812849 |

Estimation of Parameter(s): Maximum Likelihood Method

**Test Results**

| **Lower Limit** | **Upper Limit** | **Observed** | **Expected** |
| --- | --- | --- | --- |
| . | 0.00000000 | 31 | 11.02452843 |
| 0.00000000 | 0.20000000 | 0 | 5.74201474 |
| 0.20000000 | 0.40000000 | 1 | 7.13002002 |
| 0.40000000 | 0.60000000 | 1 | 8.14119023 |
| 0.60000000 | 0.80000000 | 0 | 8.54783339 |
| 0.80000000 | 1.00000000 | 33 | 8.25268549 |
| 1.00000000 | 1.20000000 | 1 | 7.32665197 |
| 1.20000000 | 1.60000000 | 1 | 10.47108945 |
| 1.60000000 | . | 8 | 7.36398629 |
|  |  | 76 | 74.00000000 |

| Chi-Square Test Statistic | : | 150.31232582 |
| --- | --- | --- |
| Degrees of Freedom | : | 6 |
| p-Value | : | 0.00000000 |

| Kolmogorov-Smirnov Test Statistic | : | 0.26993880 |
| --- | --- | --- |
| Lilliefors Probability | : | 0.00000000 |

| Shapiro-Wilk Test Statistic | : | 0.78057973 |
| --- | --- | --- |
| p-Value | : | 0.00000000 |

| Variable Name | : | AGKON2 |
| --- | --- | --- |

| Distribution | : | Normal |
| --- | --- | --- |

**Estimated Parameter(s)**

| Location or Mean (mu) | : | 0.70270270 |
| --- | --- | --- |
| Scale or SD (sigma) | : | 0.73023006 |

Estimation of Parameter(s): Maximum Likelihood Method

**Test Results**

| **Lower Limit** | **Upper Limit** | **Observed** | **Expected** |
| --- | --- | --- | --- |
| . | 0.00000000 | 34 | 12.42820141 |
| 0.00000000 | 0.20000000 | 0 | 5.74583946 |
| 0.20000000 | 0.40000000 | 1 | 6.92992498 |
| 0.40000000 | 0.60000000 | 1 | 7.75761900 |
| 0.60000000 | 0.80000000 | 0 | 8.06034066 |
| 0.80000000 | 1.00000000 | 28 | 7.77326447 |
| 1.00000000 | 1.20000000 | 1 | 6.95790559 |
| 1.20000000 | 1.60000000 | 1 | 10.23829417 |
| 1.60000000 | . | 10 | 8.10861026 |
|  |  | 76 | 74.00000000 |

| Chi-Square Test Statistic | : | 128.71996056 |
| --- | --- | --- |
| Degrees of Freedom | : | 6 |
| p-Value | : | 0.00000000 |

| Kolmogorov-Smirnov Test Statistic | : | 0.29151079 |
| --- | --- | --- |
| Lilliefors Probability | : | 0.00000000 |

| Shapiro-Wilk Test Statistic | : | 0.77370652 |
| --- | --- | --- |
| p-Value | : | 0.00000000 |

> *!! Calculating incongruence scores based on residualized, z-standardized motive scores and z-standardized goal commitment scores (see Schultheiss et al., 2011, JRP)*

> *regress*

> *model afft1 = constant wordcount_totalt1*

> *estimate*

[▼OLS Regression](file:///\\Untitled.syo)

| Dependent Variable | AFFT1 |
| --- | --- |
| N | 74 |
| Multiple R | 0.40635036 |
| Squared Multiple R | 0.16512061 |
| Adjusted Squared Multiple R | 0.15352507 |
| Standard Error of Estimate | 2.66309731 |

| **Regression Coefficients B = (X'X)^-1^X'Y** | | | | | | |
| --- | --- | --- | --- | --- | --- | --- |
| **Effect** | **Coefficient** | **Standard Error** | **Std. Coefficient** | **Tolerance** | **t** | **p-Value** |
| CONSTANT | 1.79225199 | 1.21946030 | 0.00000000 | . | 1.46970918 | 0.14599682 |
| WORDCOUNT_TOTALT1 | 0.00756797 | 0.00200551 | 0.40635036 | 1.00000000 | 3.77359271 | 0.00032768 |

| **Analysis of Variance** | | | | | |
| --- | --- | --- | --- | --- | --- |
| **Source** | **SS** | **df** | **Mean Squares** | **F-Ratio** | **p-Value** |
| Regression | 100.99133670 | 1 | 100.99133670 | 14.24000193 | 0.00032768 |
| Residual | 510.63028492 | 72 | 7.09208729 |  |  |

WARNING

| Case | 405.00000000 | is an Outlier | (Studentized Residual | : | 3.46166027) |
| --- | --- | --- | --- | --- | --- |

| Durbin-Watson D-Statistic | 1.54115603 |
| --- | --- |
| First Order Autocorrelation | 0.20241834 |

| **Information Criteria** | |
| --- | --- |
| AIC | 358.93987636 |
| AIC (Corrected) | 359.28273350 |
| Schwarz's BIC | 365.85207164 |

> *regress*

> *model acht1 = constant wordcount_totalt1*

> *estimate*

[▼OLS Regression](file:///\\Untitled.syo)

| Dependent Variable | ACHT1 |
| --- | --- |
| N | 74 |
| Multiple R | 0.46440096 |
| Squared Multiple R | 0.21566825 |
| Adjusted Squared Multiple R | 0.20477475 |
| Standard Error of Estimate | 2.07686856 |

| **Regression Coefficients B = (X'X)^-1^X'Y** | | | | | | |
| --- | --- | --- | --- | --- | --- | --- |
| **Effect** | **Coefficient** | **Standard Error** | **Std. Coefficient** | **Tolerance** | **t** | **p-Value** |
| CONSTANT | 0.70437832 | 0.95101998 | 0.00000000 | . | 0.74065564 | 0.46131090 |
| WORDCOUNT_TOTALT1 | 0.00695915 | 0.00156404 | 0.46440096 | 1.00000000 | 4.44948203 | 0.00003073 |

| **Analysis of Variance** | | | | | |
| --- | --- | --- | --- | --- | --- |
| **Source** | **SS** | **df** | **Mean Squares** | **F-Ratio** | **p-Value** |
| Regression | 85.39588345 | 1 | 85.39588345 | 19.79789030 | 0.00003073 |
| Residual | 310.56357601 | 72 | 4.31338300 |  |  |

| Durbin-Watson D-Statistic | 2.09080051 |
| --- | --- |
| First Order Autocorrelation | -0.06427696 |

| **Information Criteria** | |
| --- | --- |
| AIC | 322.14284494 |
| AIC (Corrected) | 322.48570208 |
| Schwarz's BIC | 329.05504022 |

> *regress*

> *model powt1 = constant wordcount_totalt1*

> *estimate*

[▼OLS Regression](file:///\\Untitled.syo)

| Dependent Variable | POWT1 |
| --- | --- |
| N | 74 |
| Multiple R | 0.50465845 |
| Squared Multiple R | 0.25468015 |
| Adjusted Squared Multiple R | 0.24432848 |
| Standard Error of Estimate | 2.35970683 |

| **Regression Coefficients B = (X'X)^-1^X'Y** | | | | | | |
| --- | --- | --- | --- | --- | --- | --- |
| **Effect** | **Coefficient** | **Standard Error** | **Std. Coefficient** | **Tolerance** | **t** | **p-Value** |
| CONSTANT | 0.69437612 | 1.08053461 | 0.00000000 | . | 0.64262275 | 0.52251007 |
| WORDCOUNT_TOTALT1 | 0.00881430 | 0.00177703 | 0.50465845 | 1.00000000 | 4.96012306 | 0.00000454 |

| **Analysis of Variance** | | | | | |
| --- | --- | --- | --- | --- | --- |
| **Source** | **SS** | **df** | **Mean Squares** | **F-Ratio** | **p-Value** |
| Regression | 136.99382862 | 1 | 136.99382862 | 24.60282075 | 0.00000454 |
| Residual | 400.91157678 | 72 | 5.56821634 |  |  |

| Durbin-Watson D-Statistic | 2.05482373 |
| --- | --- |
| First Order Autocorrelation | -0.03908911 |

| **Information Criteria** | |
| --- | --- |
| AIC | 341.03891234 |
| AIC (Corrected) | 341.38176948 |
| Schwarz's BIC | 347.95110762 |

> *let zafft1 = afft1 - (1.79225199 +0.00756797 *wordcount_totalt1)*

> *let zacht1 = acht1 - (0.70437832 +0.00695915*wordcount_totalt1)*

> *let zpowt1 = powt1 - (0.69437612 +0.00881430*wordcount_totalt1)*

> *CSTATISTICS zafft1 zacht1 zpowt1 / N MIN MAX MEAN SD*

[▼Descriptive Statistics](file:///\\Untitled.syo)

|  | **ZAFFT1** | **ZACHT1** | **ZPOWT1** |
| --- | --- | --- | --- |
| N of Cases | 74 | 74 | 74 |
| Minimum | -5.23362242 | -5.17427022 | -5.04067212 |
| Maximum | 8.45609081 | 4.80273473 | 6.44809848 |
| Arithmetic Mean | 0.00000219 | -0.00000165 | 0.00000274 |
| Standard Deviation | 2.64479402 | 2.06259438 | 2.34348873 |

> *stand zafft1 zacht1 zpowt1 /sd*

> *CSTATISTICS zafft1 zacht1 zpowt1 / N MIN MAX MEAN SD*

[▼Descriptive Statistics](file:///\\Untitled.syo)

|  | **ZAFFT1** | **ZACHT1** | **ZPOWT1** |
| --- | --- | --- | --- |
| N of Cases | 74 | 74 | 74 |
| Minimum | -1.97884016 | -2.50862149 | -2.15092772 |
| Maximum | 3.19725792 | 2.32849291 | 2.75149425 |
| Arithmetic Mean | 0.00000000 | 0.00000000 | 0.00000000 |
| Standard Deviation | 1.00000000 | 1.00000000 | 1.00000000 |

> *regress*

> *model afft2 = constant wordcount_total_t2*

> *estimate*

[▼OLS Regression](file:///\\Untitled.syo)

| Dependent Variable | AFFT2 |
| --- | --- |
| N | 74 |
| Multiple R | 0.49330720 |
| Squared Multiple R | 0.24335199 |
| Adjusted Squared Multiple R | 0.23284299 |
| Standard Error of Estimate | 2.70018033 |

| **Regression Coefficients B = (X'X)^-1^X'Y** | | | | | | |
| --- | --- | --- | --- | --- | --- | --- |
| **Effect** | **Coefficient** | **Standard Error** | **Std. Coefficient** | **Tolerance** | **t** | **p-Value** |
| CONSTANT | 1.01572612 | 1.09304656 | 0.00000000 | . | 0.92926154 | 0.35585863 |
| WORDCOUNT_TOTAL_T2 | 0.00852022 | 0.00177057 | 0.49330720 | 1.00000000 | 4.81212342 | 0.00000798 |

| **Analysis of Variance** | | | | | |
| --- | --- | --- | --- | --- | --- |
| **Source** | **SS** | **df** | **Mean Squares** | **F-Ratio** | **p-Value** |
| Regression | 168.83366778 | 1 | 168.83366778 | 23.15653185 | 0.00000798 |
| Residual | 524.95011601 | 72 | 7.29097383 |  |  |

| Durbin-Watson D-Statistic | 2.19119416 |
| --- | --- |
| First Order Autocorrelation | -0.10468846 |

| **Information Criteria** | |
| --- | --- |
| AIC | 360.98652585 |
| AIC (Corrected) | 361.32938299 |
| Schwarz's BIC | 367.89872113 |

> *regress*

> *model acht2 = constant wordcount_total_t2*

> *estimate*

[▼OLS Regression](file:///\\Untitled.syo)

| Dependent Variable | ACHT2 |
| --- | --- |
| N | 74 |
| Multiple R | 0.24041609 |
| Squared Multiple R | 0.05779990 |
| Adjusted Squared Multiple R | 0.04471378 |
| Standard Error of Estimate | 2.52173419 |

| **Regression Coefficients B = (X'X)^-1^X'Y** | | | | | | |
| --- | --- | --- | --- | --- | --- | --- |
| **Effect** | **Coefficient** | **Standard Error** | **Std. Coefficient** | **Tolerance** | **t** | **p-Value** |
| CONSTANT | 2.97201479 | 1.02081066 | 0.00000000 | . | 2.91142608 | 0.00478618 |
| WORDCOUNT_TOTAL_T2 | 0.00347519 | 0.00165356 | 0.24041609 | 1.00000000 | 2.10163939 | 0.03908452 |

| **Analysis of Variance** | | | | | |
| --- | --- | --- | --- | --- | --- |
| **Source** | **SS** | **df** | **Mean Squares** | **F-Ratio** | **p-Value** |
| Regression | 28.08762487 | 1 | 28.08762487 | 4.41688815 | 0.03908452 |
| Residual | 457.85832107 | 72 | 6.35914335 |  |  |

WARNING

| Case | 904.00000000 | is an Outlier | (Studentized Residual | : | 3.90130446) |
| --- | --- | --- | --- | --- | --- |

| Durbin-Watson D-Statistic | 2.38582408 |
| --- | --- |
| First Order Autocorrelation | -0.20547144 |

| **Information Criteria** | |
| --- | --- |
| AIC | 350.86751075 |
| AIC (Corrected) | 351.21036789 |
| Schwarz's BIC | 357.77970602 |

> *regress*

> *model powt2 = constant wordcount_total_t2*

> *estimate*

[▼OLS Regression](file:///\\Untitled.syo)

| Dependent Variable | POWT2 |
| --- | --- |
| N | 74 |
| Multiple R | 0.56723960 |
| Squared Multiple R | 0.32176077 |
| Adjusted Squared Multiple R | 0.31234078 |
| Standard Error of Estimate | 2.53555032 |

| **Regression Coefficients B = (X'X)^-1^X'Y** | | | | | | |
| --- | --- | --- | --- | --- | --- | --- |
| **Effect** | **Coefficient** | **Standard Error** | **Std. Coefficient** | **Tolerance** | **t** | **p-Value** |
| CONSTANT | -0.23255072 | 1.02640350 | 0.00000000 | . | -0.22656852 | 0.82140129 |
| WORDCOUNT_TOTAL_T2 | 0.00971706 | 0.00166262 | 0.56723960 | 1.00000000 | 5.84441893 | 0.00000014 |

| **Analysis of Variance** | | | | | |
| --- | --- | --- | --- | --- | --- |
| **Source** | **SS** | **df** | **Mean Squares** | **F-Ratio** | **p-Value** |
| Regression | 219.59737576 | 1 | 219.59737576 | 34.15723267 | 0.00000014 |
| Residual | 462.88911072 | 72 | 6.42901543 |  |  |

| Durbin-Watson D-Statistic | 1.92074441 |
| --- | --- |
| First Order Autocorrelation | 0.03683502 |

| **Information Criteria** | |
| --- | --- |
| AIC | 351.67616277 |
| AIC (Corrected) | 352.01901992 |
| Schwarz's BIC | 358.58835805 |

> *let zafft2 = afft2 - (1.01572612 +0.00852022 *wordcount_total_t2)*

> *let zacht2 = acht2 - (2.97201479 +0.00347519*wordcount_total_t2)*

> *let zpowt2 = powt2 - (-0.23255072 +0.00971706*wordcount_total_t2)*

> *CSTATISTICS zafft2 zacht2 zpowt2 / N MIN MAX MEAN SD*

[▼Descriptive Statistics](file:///\\Untitled.syo)

|  | **ZAFFT2** | **ZACHT2** | **ZPOWT2** |
| --- | --- | --- | --- |
| N of Cases | 74 | 74 | 74 |
| Minimum | -5.80634146 | -5.79386907 | -7.19214030 |
| Maximum | 6.88066210 | 8.92897045 | 5.55521614 |
| Arithmetic Mean | -0.00000054 | 0.00000090 | -0.00000102 |
| Standard Deviation | 2.68162217 | 2.50440248 | 2.51812365 |

> *stand zafft2 zacht2 zpowt2 /sd*

> *CSTATISTICS zafft2 zacht2 zpowt2 / N MIN MAX MEAN SD*

[▼Descriptive Statistics](file:///\\Untitled.syo)

|  | **ZAFFT2** | **ZACHT2** | **ZPOWT2** |
| --- | --- | --- | --- |
| N of Cases | 74 | 74 | 74 |
| Minimum | -2.16523453 | -2.31347397 | -2.85615017 |
| Maximum | 2.56585835 | 3.56530934 | 2.20609387 |
| Arithmetic Mean | 0.00000000 | 0.00000000 | 0.00000000 |
| Standard Deviation | 1.00000000 | 1.00000000 | 1.00000000 |

> *!! check*

> *corr*

> *pearson afft1 acht1 powt1 zafft1 zacht1 zpowt1 wordcount_totalt1 /prob*

[▼Correlation: Pearson](file:///\\Untitled.syo)

Number of Non-Missing Cases: 74

| **Pearson Correlation Matrix** | | | | | | | |
| --- | --- | --- | --- | --- | --- | --- | --- |
|  | **AFFT1** | **ACHT1** | **POWT1** | **ZAFFT1** | **ZACHT1** | **ZPOWT1** | **WORDCOUNT_TOTAL- T1** |
| AFFT1 | 1.00000000 |  |  |  |  |  |  |
| ACHT1 | 0.46259219 | 1.00000000 |  |  |  |  |  |
| POWT1 | 0.14852177 | 0.37307271 | 1.00000000 |  |  |  |  |
| ZAFFT1 | 0.91371743 | 0.29974564 | -0.06188595 | 1.00000000 |  |  |  |
| ZACHT1 | 0.30925347 | 0.88562496 | 0.15662243 | 0.33845648 | 1.00000000 |  |  |
| ZPOWT1 | -0.06549868 | 0.16066941 | 0.86331925 | -0.07168388 | 0.18141906 | 1.00000000 |  |
| WORDCOUNT_TOTALT1 | 0.40635036 | 0.46440096 | 0.50465845 | 0.00000022 | -0.00000021 | 0.00000031 | 1.00000000 |

WARNING Correlation matrix is not positive definite.

Individual significance tests are suspect.

| **Matrix of Probabilities** | | | | | | | |
| --- | --- | --- | --- | --- | --- | --- | --- |
|  | **AFFT1** | **ACHT1** | **POWT1** | **ZAFFT1** | **ZACHT1** | **ZPOWT1** | **WORDCOUNT_TOTAL- T1** |
| AFFT1 | 0.00000000 |  |  |  |  |  |  |
| ACHT1 | 0.00003331 | 0.00000000 |  |  |  |  |  |
| POWT1 | 0.20662546 | 0.00106146 | 0.00000000 |  |  |  |  |
| ZAFFT1 | 0.00000000 | 0.00947110 | 0.60041606 | 0.00000000 |  |  |  |
| ZACHT1 | 0.00733866 | 0.00000000 | 0.18265638 | 0.00318264 | 0.00000000 |  |  |
| ZPOWT1 | 0.57927470 | 0.17146769 | 0.00000000 | 0.54389643 | 0.12188158 | 0.00000000 |  |
| WORDCOUNT_TOTALT1 | 0.00032768 | 0.00003073 | 0.00000454 | 0.99999852 | 0.99999858 | 0.99999792 | 0.00000000 |

> *pearson afft2 acht2 powt2 zafft2 zacht2 zpowt2 wordcount_total_t2 /prob*

[▼Correlation: Pearson](file:///\\Untitled.syo)

Number of Non-Missing Cases: 74

| **Pearson Correlation Matrix** | | | | | | | |
| --- | --- | --- | --- | --- | --- | --- | --- |
|  | **AFFT2** | **ACHT2** | **POWT2** | **ZAFFT2** | **ZACHT2** | **ZPOWT2** | **WORDCOUNT_TOTAL- _T2** |
| AFFT2 | 1.00000000 |  |  |  |  |  |  |
| ACHT2 | 0.24609429 | 1.00000000 |  |  |  |  |  |
| POWT2 | 0.13362062 | 0.13192227 | 1.00000000 |  |  |  |  |
| ZAFFT2 | 0.86985513 | 0.14657071 | -0.16807717 | 1.00000000 |  |  |  |
| ZACHT2 | 0.13134779 | 0.97066995 | -0.00458570 | 0.15099955 | 1.00000000 |  |  |
| ZPOWT2 | -0.17752694 | -0.00540497 | 0.82355274 | -0.20408786 | -0.00556826 | 1.00000000 |  |
| WORDCOUNT_TOTAL_T2 | 0.49330720 | 0.24041609 | 0.56723960 | 0.00000000 | 0.00000011 | -0.00000012 | 1.00000000 |

WARNING Correlation matrix is not positive definite.

Individual significance tests are suspect.

| **Matrix of Probabilities** | | | | | | | |
| --- | --- | --- | --- | --- | --- | --- | --- |
|  | **AFFT2** | **ACHT2** | **POWT2** | **ZAFFT2** | **ZACHT2** | **ZPOWT2** | **WORDCOUNT_TOTAL- _T2** |
| AFFT2 | 0.00000000 |  |  |  |  |  |  |
| ACHT2 | 0.03455250 | 0.00000000 |  |  |  |  |  |
| POWT2 | 0.25638470 | 0.26253337 | 0.00000000 |  |  |  |  |
| ZAFFT2 | 0.00000000 | 0.21271813 | 0.15230274 | 0.00000000 |  |  |  |
| ZACHT2 | 0.26463557 | 0.00000000 | 0.96906877 | 0.19906814 | 0.00000000 |  |  |
| ZPOWT2 | 0.13023069 | 0.96354610 | 0.00000000 | 0.08113306 | 0.96244558 | 0.00000000 |  |
| WORDCOUNT_TOTAL_T2 | 0.00000798 | 0.03908452 | 0.00000014 | 1.00000000 | 0.99999927 | 0.99999918 | 0.00000000 |

> *let zpgqachct1 = pgq_ach_c1*

> *let zpgqaffct1 = pgq_aff_c1*

> *let zpgqindct1 = pgq_ind_c1*

> *let zpgqachct2 = pgq_ach_c2*

> *let zpgqaffct2 = pgq_aff_c2*

> *let zpgqindct2 = pgq_ind_c2*

> *stand zpgqachct1 .. zpgqindct2/sd*

> *cstats zpgqachct1 .. zpgqindct2 / mean sd*

[▼Descriptive Statistics](file:///\\Untitled.syo)

|  | **ZPGQACHCT1** | **ZPGQAFFCT1** | **ZPGQINDCT1** | **ZPGQACHCT2** | **ZPGQAFFCT2** | **ZPGQINDCT2** |
| --- | --- | --- | --- | --- | --- | --- |
| Arithmetic Mean | 0.00000000 | 0.00000000 | 0.00000000 | 0.00000000 | 0.00000000 | 0.00000000 |
| Standard Deviation | 1.00000000 | 1.00000000 | 1.00000000 | 1.00000000 | 1.00000000 | 1.00000000 |

> *LET Inkon_ind1 = log (0.5 + abs (zpowt1 - zpgqindct1))*

> *LET Inkon_ind2 = log (0.5 + abs (zpowt2 - zpgqindct2))*

> *LET Inkon_ach1 = log (0.5 + abs (zacht1 - zpgqachct1))*

> *LET Inkon_ach2 = log (0.5 + abs (zacht2 - zpgqachct2))*

> *LET Inkon_aff1 = log (0.5 + abs (zafft1 - zpgqaffct1))*

> *LET Inkon_aff2 = log (0.5 + abs (zafft2 - zpgqaffct2))*

> *LET Inkon_all1 = avg (Inkon_ind1, Inkon_ach1, Inkon_aff1)*

> *LET Inkon_all2 = avg (Inkon_ind2, Inkon_ach2, Inkon_aff2)*

> *LET Inkon_ag1 = avg (Inkon_ind1, Inkon_ach1)*

> *LET Inkon_ag2 = avg (Inkon_ind2, Inkon_ach2)*

> *FITDIST*

> *CONTINUOUS inkon_ind1 .. inkon_aff2 Inkon_all1 Inkon_all2 Inkon_ag1 Inkon_ag2/ DISTRIBUTION = {Z}*

[▼Fitting Continuous Distribution](file:///\\Untitled.syo)

| Variable Name | : | INKON_IND1 |
| --- | --- | --- |

| Distribution | : | Normal |
| --- | --- | --- |

**Estimated Parameter(s)**

| Location or Mean (mu) | : | 0.44518095 |
| --- | --- | --- |
| Scale or SD (sigma) | : | 0.43464864 |

Estimation of Parameter(s): Maximum Likelihood Method

**Test Results**

| **Lower Limit** | **Upper Limit** | **Observed** | **Expected** |
| --- | --- | --- | --- |
| . | -0.02946264 | 11 | 10.16855259 |
| -0.02946264 | 0.16376191 | 7 | 8.97273670 |
| 0.16376191 | 0.35698645 | 10 | 11.90931388 |
| 0.35698645 | 0.55021100 | 12 | 13.01431198 |
| 0.55021100 | 0.74343555 | 17 | 11.70929650 |
| 0.74343555 | 0.93666010 | 11 | 8.67386336 |
| 0.93666010 | . | 6 | 9.55192499 |
|  |  | 74 | 74.00000000 |

| Chi-Square Test Statistic | : | 5.22202225 |
| --- | --- | --- |
| Degrees of Freedom | : | 4 |
| p-Value | : | 0.26526570 |

| Kolmogorov-Smirnov Test Statistic | : | 0.08558017 |
| --- | --- | --- |
| Lilliefors Probability | : | 0.18582326 |

| Shapiro-Wilk Test Statistic | : | 0.97185064 |
| --- | --- | --- |
| p-Value | : | 0.09719680 |

| Variable Name | : | INKON_IND2 |
| --- | --- | --- |

| Distribution | : | Normal |
| --- | --- | --- |

**Estimated Parameter(s)**

| Location or Mean (mu) | : | 0.35487061 |
| --- | --- | --- |
| Scale or SD (sigma) | : | 0.50314563 |

Estimation of Parameter(s): Maximum Likelihood Method

**Test Results**

| **Lower Limit** | **Upper Limit** | **Observed** | **Expected** |
| --- | --- | --- | --- |
| . | -0.38124290 | 4 | 5.30806278 |
| -0.38124290 | -0.18315603 | 9 | 5.23408315 |
| -0.18315603 | 0.01493084 | 9 | 7.93107500 |
| 0.01493084 | 0.21301771 | 6 | 10.31261564 |
| 0.21301771 | 0.41110457 | 13 | 11.50679702 |
| 0.41110457 | 0.60919144 | 10 | 11.01768591 |
| 0.60919144 | 0.80727831 | 8 | 9.05265677 |
| 0.80727831 | 1.00536518 | 5 | 6.38276451 |
| 1.00536518 | . | 10 | 7.25425922 |
|  |  | 74 | 74.00000000 |

| Chi-Square Test Statistic | : | 6.72847188 |
| --- | --- | --- |
| Degrees of Freedom | : | 6 |
| p-Value | : | 0.34668947 |

| Kolmogorov-Smirnov Test Statistic | : | 0.06096508 |
| --- | --- | --- |
| Lilliefors Probability | : | 0.67915219 |

| Shapiro-Wilk Test Statistic | : | 0.97843103 |
| --- | --- | --- |
| p-Value | : | 0.23674026 |

| Variable Name | : | INKON_ACH1 |
| --- | --- | --- |

| Distribution | : | Normal |
| --- | --- | --- |

**Estimated Parameter(s)**

| Location or Mean (mu) | : | 0.33877977 |
| --- | --- | --- |
| Scale or SD (sigma) | : | 0.45149059 |

Estimation of Parameter(s): Maximum Likelihood Method

**Test Results**

| **Lower Limit** | **Upper Limit** | **Observed** | **Expected** |
| --- | --- | --- | --- |
| . | -0.26609997 | 9 | 6.67221298 |
| -0.26609997 | -0.05456610 | 7 | 7.52232560 |
| -0.05456610 | 0.15696777 | 7 | 11.23090895 |
| 0.15696777 | 0.36850164 | 14 | 13.51658158 |
| 0.36850164 | 0.58003552 | 13 | 13.11339224 |
| 0.58003552 | 0.79156939 | 13 | 10.25553449 |
| 0.79156939 | 1.00310326 | 6 | 6.46527942 |
| 1.00310326 | . | 5 | 5.22376474 |
|  |  | 74 | 74.00000000 |

| Chi-Square Test Statistic | : | 3.23803105 |
| --- | --- | --- |
| Degrees of Freedom | : | 5 |
| p-Value | : | 0.66334163 |

| Kolmogorov-Smirnov Test Statistic | : | 0.05279274 |
| --- | --- | --- |
| Lilliefors Probability | : | 0.90403585 |

| Shapiro-Wilk Test Statistic | : | 0.99114542 |
| --- | --- | --- |
| p-Value | : | 0.88978746 |

| Variable Name | : | INKON_ACH2 |
| --- | --- | --- |

| Distribution | : | Normal |
| --- | --- | --- |

**Estimated Parameter(s)**

| Location or Mean (mu) | : | 0.26605341 |
| --- | --- | --- |
| Scale or SD (sigma) | : | 0.53519974 |

Estimation of Parameter(s): Maximum Likelihood Method

**Test Results**

| **Lower Limit** | **Upper Limit** | **Observed** | **Expected** |
| --- | --- | --- | --- |
| . | -0.43071882 | 10 | 7.13927309 |
| -0.43071882 | -0.17584838 | 7 | 7.99327139 |
| -0.17584838 | 0.07902206 | 8 | 11.75696014 |
| 0.07902206 | 0.33389250 | 17 | 13.84251756 |
| 0.33389250 | 0.58876294 | 10 | 13.04642462 |
| 0.58876294 | 0.84363339 | 14 | 9.84287206 |
| 0.84363339 | . | 8 | 10.37868115 |
|  |  | 74 | 74.00000000 |

| Chi-Square Test Statistic | : | 6.20278246 |
| --- | --- | --- |
| Degrees of Freedom | : | 4 |
| p-Value | : | 0.18450753 |

| Kolmogorov-Smirnov Test Statistic | : | 0.05963402 |
| --- | --- | --- |
| Lilliefors Probability | : | 0.71503677 |

| Shapiro-Wilk Test Statistic | : | 0.97746831 |
| --- | --- | --- |
| p-Value | : | 0.20837075 |

| Variable Name | : | INKON_AFF1 |
| --- | --- | --- |

| Distribution | : | Normal |
| --- | --- | --- |

**Estimated Parameter(s)**

| Location or Mean (mu) | : | 0.33486522 |
| --- | --- | --- |
| Scale or SD (sigma) | : | 0.54434734 |

Estimation of Parameter(s): Maximum Likelihood Method

**Test Results**

| **Lower Limit** | **Upper Limit** | **Observed** | **Expected** |
| --- | --- | --- | --- |
| . | -0.43439129 | 9 | 5.83138901 |
| -0.43439129 | -0.23390759 | 6 | 5.12366503 |
| -0.23390759 | -0.03342390 | 6 | 7.49606080 |
| -0.03342390 | 0.16705980 | 7 | 9.59035803 |
| 0.16705980 | 0.36754350 | 8 | 10.72971290 |
| 0.36754350 | 0.56802720 | 13 | 10.49769502 |
| 0.56802720 | 0.76851089 | 4 | 8.98156824 |
| 0.76851089 | 0.96899459 | 12 | 6.71987768 |
| 0.96899459 | . | 9 | 9.02967329 |
|  |  | 74 | 74.00000000 |

| Chi-Square Test Statistic | : | 11.07271408 |
| --- | --- | --- |
| Degrees of Freedom | : | 6 |
| p-Value | : | 0.08615495 |

| Kolmogorov-Smirnov Test Statistic | : | 0.09428518 |
| --- | --- | --- |
| Lilliefors Probability | : | 0.10051260 |

| Shapiro-Wilk Test Statistic | : | 0.96406138 |
| --- | --- | --- |
| p-Value | : | 0.03357994 |

| Variable Name | : | INKON_AFF2 |
| --- | --- | --- |

| Distribution | : | Normal |
| --- | --- | --- |

**Estimated Parameter(s)**

| Location or Mean (mu) | : | 0.39177101 |
| --- | --- | --- |
| Scale or SD (sigma) | : | 0.48668419 |

Estimation of Parameter(s): Maximum Likelihood Method

**Test Results**

| **Lower Limit** | **Upper Limit** | **Observed** | **Expected** |
| --- | --- | --- | --- |
| . | -0.17324939 | 14 | 9.08936709 |
| -0.17324939 | -0.00202479 | 5 | 6.39270733 |
| -0.00202479 | 0.16919981 | 10 | 8.47320178 |
| 0.16919981 | 0.34042441 | 3 | 9.93586382 |
| 0.34042441 | 0.51164901 | 5 | 10.30766164 |
| 0.51164901 | 0.68287361 | 11 | 9.46044077 |
| 0.68287361 | 0.85409821 | 10 | 7.68172395 |
| 0.85409821 | 1.02532281 | 10 | 5.51824153 |
| 1.02532281 | . | 6 | 7.14079208 |
|  |  | 74 | 74.00000000 |

| Chi-Square Test Statistic | : | 15.57865318 |
| --- | --- | --- |
| Degrees of Freedom | : | 6 |
| p-Value | : | 0.01620337 |

| Kolmogorov-Smirnov Test Statistic | : | 0.10866484 |
| --- | --- | --- |
| Lilliefors Probability | : | 0.03046106 |

| Shapiro-Wilk Test Statistic | : | 0.94142991 |
| --- | --- | --- |
| p-Value | : | 0.00188935 |

| Variable Name | : | INKON_ALL1 |
| --- | --- | --- |

| Distribution | : | Normal |
| --- | --- | --- |

**Estimated Parameter(s)**

| Location or Mean (mu) | : | 0.37294198 |
| --- | --- | --- |
| Scale or SD (sigma) | : | 0.32493432 |

Estimation of Parameter(s): Maximum Likelihood Method

**Test Results**

| **Lower Limit** | **Upper Limit** | **Observed** | **Expected** |
| --- | --- | --- | --- |
| . | -0.05429443 | 8 | 6.97686504 |
| -0.05429443 | 0.07745709 | 7 | 6.45990231 |
| 0.07745709 | 0.20920860 | 10 | 9.29358602 |
| 0.20920860 | 0.34096012 | 9 | 11.36863727 |
| 0.34096012 | 0.47271164 | 14 | 11.82508937 |
| 0.47271164 | 0.60446315 | 6 | 10.45856303 |
| 0.60446315 | 0.73621467 | 7 | 7.86518976 |
| 0.73621467 | . | 13 | 9.75216720 |
|  |  | 74 | 74.00000000 |

| Chi-Square Test Statistic | : | 4.21994975 |
| --- | --- | --- |
| Degrees of Freedom | : | 5 |
| p-Value | : | 0.51820266 |

| Kolmogorov-Smirnov Test Statistic | : | 0.07220237 |
| --- | --- | --- |
| Lilliefors Probability | : | 0.40752977 |

| Shapiro-Wilk Test Statistic | : | 0.97855662 |
| --- | --- | --- |
| p-Value | : | 0.24068893 |

| Variable Name | : | INKON_ALL2 |
| --- | --- | --- |

| Distribution | : | Normal |
| --- | --- | --- |

**Estimated Parameter(s)**

| Location or Mean (mu) | : | 0.33756501 |
| --- | --- | --- |
| Scale or SD (sigma) | : | 0.28943145 |

Estimation of Parameter(s): Maximum Likelihood Method

**Test Results**

| **Lower Limit** | **Upper Limit** | **Observed** | **Expected** |
| --- | --- | --- | --- |
| . | -0.08960787 | 6 | 5.17888291 |
| -0.08960787 | 0.04760604 | 7 | 6.52900031 |
| 0.04760604 | 0.18481995 | 10 | 10.40622473 |
| 0.18481995 | 0.32203386 | 14 | 13.30248981 |
| 0.32203386 | 0.45924777 | 13 | 13.63879106 |
| 0.45924777 | 0.59646168 | 10 | 11.21561374 |
| 0.59646168 | 0.73367559 | 6 | 7.39718484 |
| 0.73367559 | . | 8 | 6.33181261 |
|  |  | 74 | 74.00000000 |

| Chi-Square Test Statistic | : | 1.08167586 |
| --- | --- | --- |
| Degrees of Freedom | : | 5 |
| p-Value | : | 0.95571276 |

| Kolmogorov-Smirnov Test Statistic | : | 0.05112855 |
| --- | --- | --- |
| Lilliefors Probability | : | 0.94984618 |

| Shapiro-Wilk Test Statistic | : | 0.99125976 |
| --- | --- | --- |
| p-Value | : | 0.89503395 |

| Variable Name | : | INKON_AG1 |
| --- | --- | --- |

| Distribution | : | Normal |
| --- | --- | --- |

**Estimated Parameter(s)**

| Location or Mean (mu) | : | 0.39198036 |
| --- | --- | --- |
| Scale or SD (sigma) | : | 0.31361795 |

Estimation of Parameter(s): Maximum Likelihood Method

**Test Results**

| **Lower Limit** | **Upper Limit** | **Observed** | **Expected** |
| --- | --- | --- | --- |
| . | 0.02007011 | 7 | 8.71991919 |
| 0.02007011 | 0.17600858 | 10 | 9.44878301 |
| 0.17600858 | 0.33194704 | 15 | 13.21451772 |
| 0.33194704 | 0.48788550 | 12 | 14.50584342 |
| 0.48788550 | 0.64382397 | 14 | 12.49844373 |
| 0.64382397 | 0.79976243 | 9 | 8.45244010 |
| 0.79976243 | . | 7 | 7.16005284 |
|  |  | 74 | 74.00000000 |

| Chi-Square Test Statistic | : | 1.26496257 |
| --- | --- | --- |
| Degrees of Freedom | : | 4 |
| p-Value | : | 0.86729148 |

| Kolmogorov-Smirnov Test Statistic | : | 0.06114162 |
| --- | --- | --- |
| Lilliefors Probability | : | 0.67443344 |

| Shapiro-Wilk Test Statistic | : | 0.98826605 |
| --- | --- | --- |
| p-Value | : | 0.72946553 |

| Variable Name | : | INKON_AG2 |
| --- | --- | --- |

| Distribution | : | Normal |
| --- | --- | --- |

**Estimated Parameter(s)**

| Location or Mean (mu) | : | 0.31046201 |
| --- | --- | --- |
| Scale or SD (sigma) | : | 0.37482894 |

Estimation of Parameter(s): Maximum Likelihood Method

**Test Results**

| **Lower Limit** | **Upper Limit** | **Observed** | **Expected** |
| --- | --- | --- | --- |
| . | -0.16656055 | 8 | 7.51638659 |
| -0.16656055 | -0.01123764 | 8 | 6.94137978 |
| -0.01123764 | 0.14408526 | 12 | 9.85618543 |
| 0.14408526 | 0.29940817 | 8 | 11.81556795 |
| 0.29940817 | 0.45473107 | 10 | 11.95876463 |
| 0.45473107 | 0.61005398 | 12 | 10.21890197 |
| 0.61005398 | 0.76537688 | 6 | 7.37234215 |
| 0.76537688 | . | 10 | 8.32047151 |
|  |  | 74 | 74.00000000 |

| Chi-Square Test Statistic | : | 3.11676268 |
| --- | --- | --- |
| Degrees of Freedom | : | 5 |
| p-Value | : | 0.68199001 |

| Kolmogorov-Smirnov Test Statistic | : | 0.05370443 |
| --- | --- | --- |
| Lilliefors Probability | : | 0.87877140 |

| Shapiro-Wilk Test Statistic | : | 0.98686282 |
| --- | --- | --- |
| p-Value | : | 0.64236045 |

> *!! Aggregating hedonic tone scale*

> *cronbach ZUFRIEDEN ANGENEHM BEDRUCKT BETRUBT DEPRIMIERT ENTTAUSCHT FREUDIG FRUSTRIERT GLUCKLICH GUTGELAUNT HEITER TRAURIG*

[▼Cronbach's Alpha](file:///\\Untitled.syo)

| Number of Variables | 12 |  |  |  |  |  |  |  |  |  |  |
| --- | --- | --- | --- | --- | --- | --- | --- | --- | --- | --- | --- |
| Number of Cases | 74 |  |  |  |  |  |  |  |  |  |  |
| Variables | ZUFRIEDEN | ANGENEHM | BEDRUCKT | BETRUBT | DEPRIMIERT | ENTTAUSCHT | FREUDIG | FRUSTRIERT | GLUCKLICH | GUTGELAUNT | HEITER |
| Cronbach's Alpha | 0.93135361 |  |  |  |  |  |  |  |  |  |  |

| Number of Variables |  |
| --- | --- |
| Number of Cases |  |
| Variables | TRAURIG |
| Cronbach's Alpha |  |

> *cronbach ZUFRIEDEN_t2 ANGENEHM_t2 BEDRüCKT_t2 BETRüBT_t2 DEPRIMIERT_t2 ENTTäUSCHT_t2 FREUDIG_t2 FRUSTRIERT_t2 GLüCKLICH_t2 GUT_GELAUNT_t2 HEITER_t2 TRAURIG_t2*

[▼Cronbach's Alpha](file:///\\Untitled.syo)

| Number of Variables | 12 |  |  |  |  |  |  |  |
| --- | --- | --- | --- | --- | --- | --- | --- | --- |
| Number of Cases | 73 |  |  |  |  |  |  |  |
| Variables | ZUFRIEDEN_T2 | ANGENEHM_T2 | BEDRüCKT_T2 | BETRüBT_T2 | DEPRIMIERT_T2 | ENTTäUSCHT_T2 | FREUDIG_T2 | FRUSTRIERT_T2 |
| Cronbach's Alpha | 0.91896179 |  |  |  |  |  |  |  |

| Number of Variables |  |  |  |  |
| --- | --- | --- | --- | --- |
| Number of Cases |  |  |  |  |
| Variables | GLüCKLICH_T2 | GUT_GELAUNT_T2 | HEITER_T2 | TRAURIG_T2 |
| Cronbach's Alpha |  |  |  |  |

> *let ht1 = avg (ZUFRIEDEN ,ANGENEHM ,BEDRUCKT ,BETRUBT ,DEPRIMIERT ,ENTTAUSCHT ,FREUDIG ,FRUSTRIERT ,GLUCKLICH ,GUTGELAUNT ,HEITER ,TRAURIG)*

> *let ht2 = avg (ZUFRIEDEN_t2 ,ANGENEHM_t2 ,BEDRüCKT_t2 ,BETRüBT_t2 ,DEPRIMIERT_t2 ,ENTTäUSCHT_t2 ,FREUDIG_t2 ,FRUSTRIERT_t2 ,GLüCKLICH_t2 ,GUT_GELAUNT_t2 ,HEITER_t2
,TRAURIG_t2)*

> *FITDIST*

> *CONTINUOUS ht1 ht2 / DISTRIBUTION = {Z}*

[▼Fitting Continuous Distribution](file:///\\Untitled.syo)

| Variable Name | : | HT1 |
| --- | --- | --- |

| Distribution | : | Normal |
| --- | --- | --- |

**Estimated Parameter(s)**

| Location or Mean (mu) | : | 3.53603604 |
| --- | --- | --- |
| Scale or SD (sigma) | : | 0.79330450 |

Estimation of Parameter(s): Maximum Likelihood Method

**Test Results**

| **Lower Limit** | **Upper Limit** | **Observed** | **Expected** |
| --- | --- | --- | --- |
| . | 2.59166667 | 11 | 8.65354014 |
| 2.59166667 | 2.90000000 | 7 | 6.98615857 |
| 2.90000000 | 3.20833333 | 10 | 9.50342433 |
| 3.20833333 | 3.51666667 | 8 | 11.13614431 |
| 3.51666667 | 3.82500000 | 9 | 11.24099080 |
| 3.82500000 | 4.13333333 | 7 | 9.77438551 |
| 4.13333333 | 4.44166667 | 11 | 7.32129205 |
| 4.44166667 | . | 11 | 9.38406429 |
|  |  | 74 | 74.00000000 |

| Chi-Square Test Statistic | : | 4.90637101 |
| --- | --- | --- |
| Degrees of Freedom | : | 5 |
| p-Value | : | 0.42741344 |

| Kolmogorov-Smirnov Test Statistic | : | 0.09931630 |
| --- | --- | --- |
| Lilliefors Probability | : | 0.06789153 |

| Shapiro-Wilk Test Statistic | : | 0.96009813 |
| --- | --- | --- |
| p-Value | : | 0.01975623 |

| Variable Name | : | HT2 |
| --- | --- | --- |

| Distribution | : | Normal |
| --- | --- | --- |

**Estimated Parameter(s)**

| Location or Mean (mu) | : | 3.64840183 |
| --- | --- | --- |
| Scale or SD (sigma) | : | 0.69670438 |

Estimation of Parameter(s): Maximum Likelihood Method

**Test Results**

| **Lower Limit** | **Upper Limit** | **Observed** | **Expected** |
| --- | --- | --- | --- |
| . | 2.66666667 | 7 | 5.79626469 |
| 2.66666667 | 3.00000000 | 7 | 7.05262297 |
| 3.00000000 | 3.33333333 | 7 | 10.91646300 |
| 3.33333333 | 3.66666667 | 12 | 13.49804646 |
| 3.66666667 | 4.00000000 | 17 | 13.33296409 |
| 4.00000000 | 4.33333333 | 14 | 10.52080057 |
| 4.33333333 | 4.66666667 | 7 | 6.63173217 |
| 4.66666667 | . | 2 | 5.25110605 |
|  |  | 73 | 73.00000000 |

| Chi-Square Test Statistic | : | 6.01415739 |
| --- | --- | --- |
| Degrees of Freedom | : | 5 |
| p-Value | : | 0.30484378 |

| Kolmogorov-Smirnov Test Statistic | : | 0.10663062 |
| --- | --- | --- |
| Lilliefors Probability | : | 0.03891526 |

| Shapiro-Wilk Test Statistic | : | 0.96016993 |
| --- | --- | --- |
| p-Value | : | 0.02122270 |

> *!! Aggregating BDI scale*

> *cronbach bdi1 .. bdi21*

[▼Cronbach's Alpha](file:///\\Untitled.syo)

| Number of Variables | 21 |  |  |  |  |  |  |  |  |  |  |  |  |  |  |  |  |  |  |
| --- | --- | --- | --- | --- | --- | --- | --- | --- | --- | --- | --- | --- | --- | --- | --- | --- | --- | --- | --- |
| Number of Cases | 74 |  |  |  |  |  |  |  |  |  |  |  |  |  |  |  |  |  |  |
| Variables | BDI1 | BDI2 | BDI3 | BDI4 | BDI5 | BDI6 | BDI7 | BDI8 | BDI9 | BDI10 | BDI11 | BDI12 | BDI13 | BDI14 | BDI15 | BDI16 | BDI17 | BDI18 | BDI19 |
| Cronbach's Alpha | 0.82021775 |  |  |  |  |  |  |  |  |  |  |  |  |  |  |  |  |  |  |

| Number of Variables |  |  |
| --- | --- | --- |
| Number of Cases |  |  |
| Variables | BDI20 | BDI21 |
| Cronbach's Alpha |  |  |

> *cronbach bdi1_t2 .. bdi21_t2*

[▼Cronbach's Alpha](file:///\\Untitled.syo)

| Number of Variables | 21 |  |  |  |  |  |  |  |  |  |  |  |
| --- | --- | --- | --- | --- | --- | --- | --- | --- | --- | --- | --- | --- |
| Number of Cases | 73 |  |  |  |  |  |  |  |  |  |  |  |
| Variables | BDI1_T2 | BDI2_T2 | BDI3_T2 | BDI4_T2 | BDI5_T2 | BDI6_T2 | BDI7_T2 | BDI8_T2 | BDI9_T2 | BDI10_T2 | BDI11_T2 | BDI12_T2 |
| Cronbach's Alpha | 0.81805276 |  |  |  |  |  |  |  |  |  |  |  |

| Number of Variables |  |  |  |  |  |  |  |  |  |
| --- | --- | --- | --- | --- | --- | --- | --- | --- | --- |
| Number of Cases |  |  |  |  |  |  |  |  |  |
| Variables | BDI13_T2 | BDI14_T2 | BDI15_T2 | BDI16_T2 | BDI17_T2 | BDI18_T2 | BDI19_T2 | BDI20_T2 | BDI21_T2 |
| Cronbach's Alpha |  |  |  |  |  |  |  |  |  |

> *let bdi_t1 = sum (bdi1 .. bdi21)*

> *let bdi_t2 = sum (bdi1_t2 .. bdi21_t2)*

> *cstats bdi_t1 bdi_t2 /mean sd*

[▼Descriptive Statistics](file:///\\Untitled.syo)

|  | **BDI_T1** | **BDI_T2** |
| --- | --- | --- |
| Arithmetic Mean | 8.45945946 | 7.20547945 |
| Standard Deviation | 6.60259225 | 5.80459326 |

> *let sqr_bdi_t1 = sqr (1 + bdi_t1)*

> *let sqr_bdi_t2 = sqr (1 + bdi_t2)*

> *FITDIST*

> *CONTINUOUS bdi_t1 bdi_t2 sqr_bdi_t1 sqr_bdi_t2 / DISTRIBUTION = {Z}*

[▼Fitting Continuous Distribution](file:///\\Untitled.syo)

| Variable Name | : | BDI_T1 |
| --- | --- | --- |

| Distribution | : | Normal |
| --- | --- | --- |

**Estimated Parameter(s)**

| Location or Mean (mu) | : | 8.45945946 |
| --- | --- | --- |
| Scale or SD (sigma) | : | 6.55782839 |

Estimation of Parameter(s): Maximum Likelihood Method

**Test Results**

| **Lower Limit** | **Upper Limit** | **Observed** | **Expected** |
| --- | --- | --- | --- |
| . | 0.00000000 | 5 | 7.29114699 |
| 0.00000000 | 2.90000000 | 9 | 7.38201999 |
| 2.90000000 | 5.80000000 | 19 | 10.67482545 |
| 5.80000000 | 8.70000000 | 11 | 12.73461910 |
| 8.70000000 | 11.60000000 | 7 | 12.53301300 |
| 11.60000000 | 14.50000000 | 6 | 10.17580895 |
| 14.50000000 | 17.40000000 | 8 | 6.81586104 |
| 17.40000000 | . | 9 | 6.39270549 |
|  |  | 74 | 74.00000000 |

| Chi-Square Test Statistic | : | 13.22899487 |
| --- | --- | --- |
| Degrees of Freedom | : | 5 |
| p-Value | : | 0.02132478 |

| Kolmogorov-Smirnov Test Statistic | : | 0.14703359 |
| --- | --- | --- |
| Lilliefors Probability | : | 0.00042370 |

| Shapiro-Wilk Test Statistic | : | 0.92021869 |
| --- | --- | --- |
| p-Value | : | 0.00017829 |

| Variable Name | : | BDI_T2 |
| --- | --- | --- |

| Distribution | : | Normal |
| --- | --- | --- |

**Estimated Parameter(s)**

| Location or Mean (mu) | : | 7.20547945 |
| --- | --- | --- |
| Scale or SD (sigma) | : | 5.76469867 |

Estimation of Parameter(s): Maximum Likelihood Method

**Test Results**

| **Lower Limit** | **Upper Limit** | **Observed** | **Expected** |
| --- | --- | --- | --- |
| . | 0.00000000 | 4 | 7.71334457 |
| 0.00000000 | 2.60000000 | 10 | 7.77514552 |
| 2.60000000 | 5.20000000 | 20 | 11.08069941 |
| 5.20000000 | 7.80000000 | 11 | 12.92896402 |
| 7.80000000 | 10.40000000 | 11 | 12.35102078 |
| 10.40000000 | 13.00000000 | 7 | 9.66013432 |
| 13.00000000 | 15.60000000 | 4 | 6.18581960 |
| 15.60000000 | . | 6 | 5.30487179 |
|  |  | 73 | 73.00000000 |

| Chi-Square Test Statistic | : | 11.63538923 |
| --- | --- | --- |
| Degrees of Freedom | : | 5 |
| p-Value | : | 0.04014016 |

| Kolmogorov-Smirnov Test Statistic | : | 0.14381943 |
| --- | --- | --- |
| Lilliefors Probability | : | 0.00072739 |

| Shapiro-Wilk Test Statistic | : | 0.89583238 |
| --- | --- | --- |
| p-Value | : | 0.00001856 |

| Variable Name | : | SQR_BDI_T1 |
| --- | --- | --- |

| Distribution | : | Normal |
| --- | --- | --- |

**Estimated Parameter(s)**

| Location or Mean (mu) | : | 2.87720761 |
| --- | --- | --- |
| Scale or SD (sigma) | : | 1.08680072 |

Estimation of Parameter(s): Maximum Likelihood Method

**Test Results**

| **Lower Limit** | **Upper Limit** | **Observed** | **Expected** |
| --- | --- | --- | --- |
| . | 1.44772256 | 8 | 6.97095285 |
| 1.44772256 | 1.89544512 | 6 | 6.58361405 |
| 1.89544512 | 2.34316767 | 12 | 9.50208039 |
| 2.34316767 | 2.79089023 | 11 | 11.60110008 |
| 2.79089023 | 3.23861279 | 10 | 11.98144750 |
| 3.23861279 | 3.68633535 | 4 | 10.46766021 |
| 3.68633535 | 4.13405790 | 11 | 7.73602348 |
| 4.13405790 | . | 12 | 9.15712144 |
|  |  | 74 | 74.00000000 |

| Chi-Square Test Statistic | : | 7.47502788 |
| --- | --- | --- |
| Degrees of Freedom | : | 5 |
| p-Value | : | 0.18763995 |

| Kolmogorov-Smirnov Test Statistic | : | 0.09899183 |
| --- | --- | --- |
| Lilliefors Probability | : | 0.06968878 |

| Shapiro-Wilk Test Statistic | : | 0.96677452 |
| --- | --- | --- |
| p-Value | : | 0.04852106 |

| Variable Name | : | SQR_BDI_T2 |
| --- | --- | --- |

| Distribution | : | Normal |
| --- | --- | --- |

**Estimated Parameter(s)**

| Location or Mean (mu) | : | 2.69269447 |
| --- | --- | --- |
| Scale or SD (sigma) | : | 0.97717755 |

Estimation of Parameter(s): Maximum Likelihood Method

**Test Results**

| **Lower Limit** | **Upper Limit** | **Observed** | **Expected** |
| --- | --- | --- | --- |
| . | 1.41961524 | 11 | 7.03131437 |
| 1.41961524 | 1.83923048 | 3 | 6.92799034 |
| 1.83923048 | 2.25884573 | 16 | 10.02323826 |
| 2.25884573 | 2.67846097 | 8 | 12.09327167 |
| 2.67846097 | 3.09807621 | 13 | 12.16798791 |
| 3.09807621 | 3.51769145 | 8 | 10.21017236 |
| 3.51769145 | 3.93730670 | 7 | 7.14467977 |
| 3.93730670 | . | 7 | 7.40134532 |
|  |  | 73 | 73.00000000 |

| Chi-Square Test Statistic | : | 9.97648557 |
| --- | --- | --- |
| Degrees of Freedom | : | 5 |
| p-Value | : | 0.07590427 |

| Kolmogorov-Smirnov Test Statistic | : | 0.09081313 |
| --- | --- | --- |
| Lilliefors Probability | : | 0.13543744 |

| Shapiro-Wilk Test Statistic | : | 0.97431798 |
| --- | --- | --- |
| p-Value | : | 0.14173476 |

> *!! Aggregating SWLS scale*

> *cronbach swls1 .. swls5*

[▼Cronbach's Alpha](file:///\\Untitled.syo)

| Number of Variables | 5 |  |  |  |  |
| --- | --- | --- | --- | --- | --- |
| Number of Cases | 74 |  |  |  |  |
| Variables | SWLS1 | SWLS2 | SWLS3 | SWLS4 | SWLS5 |
| Cronbach's Alpha | 0.75567184 |  |  |  |  |

> *cronbach swls1_t2 .. swls5_t2*

[▼Cronbach's Alpha](file:///\\Untitled.syo)

| Number of Variables | 5 |  |  |  |  |
| --- | --- | --- | --- | --- | --- |
| Number of Cases | 74 |  |  |  |  |
| Variables | SWLS1_T2 | SWLS2_T2 | SWLS3_T2 | SWLS4_T2 | SWLS5_T2 |
| Cronbach's Alpha | 0.80524469 |  |  |  |  |

> *let swls_t1 = AVG ( swls1 .. swls5)*

> *let swls_t2 = avg (swls1_t2 .. swls5_t2)*

> *cstats swls_t1 swls_t2 /mean sd*

[▼Descriptive Statistics](file:///\\Untitled.syo)

|  | **SWLS_T1** | **SWLS_T2** |
| --- | --- | --- |
| Arithmetic Mean | 4.75945946 | 4.85945946 |
| Standard Deviation | 1.03260794 | 1.05556826 |

> *FITDIST*

> *CONTINUOUS swls_t1 swls_t2 / DISTRIBUTION = {Z}*

[▼Fitting Continuous Distribution](file:///\\Untitled.syo)

| Variable Name | : | SWLS_T1 |
| --- | --- | --- |

| Distribution | : | Normal |
| --- | --- | --- |

**Estimated Parameter(s)**

| Location or Mean (mu) | : | 4.75945946 |
| --- | --- | --- |
| Scale or SD (sigma) | : | 1.02560713 |

Estimation of Parameter(s): Maximum Likelihood Method

**Test Results**

| **Lower Limit** | **Upper Limit** | **Observed** | **Expected** |
| --- | --- | --- | --- |
| . | 3.38000000 | 8 | 6.60892545 |
| 3.38000000 | 3.84000000 | 8 | 7.08049699 |
| 3.84000000 | 4.30000000 | 4 | 10.51457216 |
| 4.30000000 | 4.76000000 | 11 | 12.81156467 |
| 4.76000000 | 5.22000000 | 21 | 12.80858695 |
| 5.22000000 | 5.68000000 | 8 | 10.50724216 |
| 5.68000000 | 6.14000000 | 9 | 7.07227185 |
| 6.14000000 | . | 5 | 6.59633978 |
|  |  | 74 | 74.00000000 |

| Chi-Square Test Statistic | : | 11.45330155 |
| --- | --- | --- |
| Degrees of Freedom | : | 5 |
| p-Value | : | 0.04309732 |

| Kolmogorov-Smirnov Test Statistic | : | 0.10622860 |
| --- | --- | --- |
| Lilliefors Probability | : | 0.03787832 |

| Shapiro-Wilk Test Statistic | : | 0.96580680 |
| --- | --- | --- |
| p-Value | : | 0.04253513 |

| Variable Name | : | SWLS_T2 |
| --- | --- | --- |

| Distribution | : | Normal |
| --- | --- | --- |

**Estimated Parameter(s)**

| Location or Mean (mu) | : | 4.85945946 |
| --- | --- | --- |
| Scale or SD (sigma) | : | 1.04841179 |

Estimation of Parameter(s): Maximum Likelihood Method

**Test Results**

| **Lower Limit** | **Upper Limit** | **Observed** | **Expected** |
| --- | --- | --- | --- |
| . | 3.50000000 | 8 | 7.20536953 |
| 3.50000000 | 4.00000000 | 6 | 8.05141928 |
| 4.00000000 | 4.50000000 | 12 | 11.81622484 |
| 4.50000000 | 5.00000000 | 16 | 13.87258023 |
| 5.00000000 | 5.50000000 | 11 | 13.02910901 |
| 5.50000000 | 6.00000000 | 12 | 9.78922950 |
| 6.00000000 | . | 9 | 10.23606761 |
|  |  | 74 | 74.00000000 |

| Chi-Square Test Statistic | : | 1.90396520 |
| --- | --- | --- |
| Degrees of Freedom | : | 4 |
| p-Value | : | 0.75341654 |

| Kolmogorov-Smirnov Test Statistic | : | 0.07548270 |
| --- | --- | --- |
| Lilliefors Probability | : | 0.34219569 |

| Shapiro-Wilk Test Statistic | : | 0.98254037 |
| --- | --- | --- |
| p-Value | : | 0.39907618 |

> *!!!!!!!!!!!!!!!!!!!!!!!!!!!!!!!!!!!!!!!!!!!!!!!!!!!!!!!!!!!!!!!!!!!!!!!!!!!!!!!!!!!!!!!!!!!!!*

> *!!*

> *!! R E S U L T S*

> *!!*

> *!!!!!!!!!!!!!!!!!!!!!!!!!!!!!!!!!!!!!!!!!!!!!!!!!!!!!!!!!!!!!!!!!!!!!!!!!!!!!!!!!!!!!!!!!!!!!*

> *!! Generating Table 1*

> *format 12, 2*

> *corr*

> *pearson cpt1 cat1 cit1 cpt2 cat2 cit2 PGQ_IND_C1 PGQ_ach_C1 PGQ_AFF_C1 PGQ_IND_C2 PGQ_ach_C2 PGQ_AFF_C2 iemind1 iemach1 iemaff1 kongruenz1 iemind2 iemach2 iemaff2
kongruenz2 Inkon_ind1 Inkon_ach1 Inkon_aff1 inkon_all1 Inkon_ind2 Inkon_ach2 Inkon_aff2 inkon_all2 ht1 ht2 sqr_bdi_t1 sqr_bdi_t2 swls_t1 swls_t2 /prob pairwise*

[▼Correlation: Pearson](file:///\\Untitled.syo)

| **Pearson Correlation Matrix** | | | | | | | | | | | | | | |
| --- | --- | --- | --- | --- | --- | --- | --- | --- | --- | --- | --- | --- | --- | --- |
|  | **CPT1** | **CAT1** | **CIT1** | **CPT2** | **CAT2** | **CIT2** | **PGQ_IND_C1** | **PGQ_ACH_C1** | **PGQ_AFF_C1** | **PGQ_IND_C2** | **PGQ_ACH_C2** | **PGQ_AFF_C2** | **IEMIND1** | **IEMACH1** |
| CPT1 | 1.00 |  |  |  |  |  |  |  |  |  |  |  |  |  |
| CAT1 | 0.18 | 1.00 |  |  |  |  |  |  |  |  |  |  |  |  |
| CIT1 | -0.09 | 0.35 | 1.00 |  |  |  |  |  |  |  |  |  |  |  |
| CPT2 | 0.27 | 0.13 | -0.09 | 1.00 |  |  |  |  |  |  |  |  |  |  |
| CAT2 | 0.03 | 0.28 | 0.40 | -0.10 | 1.00 |  |  |  |  |  |  |  |  |  |
| CIT2 | -0.03 | 0.32 | 0.38 | -0.26 | 0.25 | 1.00 |  |  |  |  |  |  |  |  |
| PGQ_IND_C1 | -0.03 | 0.09 | 0.15 | -0.17 | 0.13 | 0.10 | 1.00 |  |  |  |  |  |  |  |
| PGQ_ACH_C1 | -0.03 | 0.17 | 0.05 | -0.04 | -0.02 | 0.17 | 0.22 | 1.00 |  |  |  |  |  |  |
| PGQ_AFF_C1 | 0.28 | 0.15 | -0.01 | 0.06 | -0.13 | 0.01 | 0.19 | 0.33 | 1.00 |  |  |  |  |  |
| PGQ_IND_C2 | 0.10 | 0.14 | 0.10 | -0.01 | 0.03 | -0.04 | 0.58 | 0.32 | 0.28 | 1.00 |  |  |  |  |
| PGQ_ACH_C2 | 0.10 | 0.23 | 0.07 | 0.07 | 0.02 | 0.08 | 0.35 | 0.66 | 0.37 | 0.47 | 1.00 |  |  |  |
| PGQ_AFF_C2 | -0.07 | 0.08 | 0.02 | 0.00 | -0.02 | 0.07 | 0.53 | 0.31 | 0.39 | 0.39 | 0.39 | 1.00 |  |  |
| IEMIND1 | 0.52 | 0.14 | 0.16 | 0.03 | 0.08 | 0.06 | 0.28 | 0.05 | 0.17 | 0.20 | 0.10 | 0.13 | 1.00 |  |
| IEMACH1 | 0.03 | 0.64 | 0.28 | 0.04 | 0.13 | 0.13 | 0.16 | 0.52 | 0.21 | 0.28 | 0.41 | 0.05 | 0.04 | 1.00 |
| IEMAFF1 | -0.01 | 0.35 | 0.58 | 0.03 | 0.01 | 0.25 | 0.22 | 0.25 | 0.42 | 0.28 | 0.31 | 0.33 | 0.07 | 0.43 |
| KONGRUENZ1 | 0.26 | 0.57 | 0.50 | 0.05 | 0.12 | 0.21 | 0.33 | 0.41 | 0.39 | 0.38 | 0.41 | 0.25 | 0.54 | 0.74 |
| IEMIND2 | 0.16 | 0.23 | -0.07 | 0.57 | 0.03 | -0.08 | 0.09 | 0.06 | 0.21 | 0.44 | 0.20 | 0.14 | 0.12 | 0.10 |
| IEMACH2 | 0.10 | 0.21 | 0.24 | -0.02 | 0.55 | 0.36 | 0.09 | 0.31 | 0.05 | 0.25 | 0.33 | 0.14 | 0.12 | 0.30 |
| IEMAFF2 | -0.12 | 0.29 | 0.27 | -0.22 | 0.14 | 0.59 | 0.38 | 0.26 | 0.07 | 0.19 | 0.27 | 0.48 | 0.12 | 0.22 |
| KONGRUENZ2 | 0.07 | 0.37 | 0.23 | 0.16 | 0.37 | 0.44 | 0.28 | 0.32 | 0.17 | 0.44 | 0.41 | 0.38 | 0.18 | 0.31 |
| INKON_IND1 | -0.02 | -0.06 | -0.15 | 0.00 | 0.01 | 0.11 | -0.23 | 0.04 | -0.03 | -0.01 | -0.05 | -0.08 | -0.43 | 0.01 |
| INKON_ACH1 | 0.06 | 0.07 | 0.08 | -0.06 | 0.11 | 0.17 | 0.16 | -0.08 | 0.03 | 0.02 | 0.01 | 0.20 | 0.09 | -0.29 |
| INKON_AFF1 | 0.08 | 0.05 | 0.10 | -0.15 | 0.18 | 0.11 | 0.09 | -0.01 | -0.02 | 0.12 | -0.02 | -0.14 | 0.17 | -0.07 |
| INKON_ALL1 | 0.07 | 0.03 | 0.03 | -0.12 | 0.16 | 0.19 | 0.02 | -0.02 | -0.01 | 0.07 | -0.03 | -0.02 | -0.06 | -0.17 |
| INKON_IND2 | 0.00 | -0.13 | -0.03 | -0.08 | -0.05 | -0.03 | 0.01 | 0.04 | -0.15 | -0.14 | -0.14 | 0.13 | -0.15 | -0.11 |
| INKON_ACH2 | 0.07 | -0.10 | -0.04 | -0.08 | 0.05 | -0.22 | -0.01 | -0.15 | -0.07 | -0.06 | -0.17 | -0.29 | -0.02 | -0.11 |
| INKON_AFF2 | 0.06 | -0.12 | -0.11 | 0.11 | 0.00 | -0.07 | -0.18 | -0.11 | 0.01 | -0.17 | -0.16 | -0.13 | -0.05 | -0.20 |
| INKON_ALL2 | 0.07 | -0.21 | -0.10 | -0.03 | 0.00 | -0.19 | -0.10 | -0.13 | -0.13 | -0.21 | -0.28 | -0.18 | -0.13 | -0.24 |
| HT1 | 0.02 | 0.18 | 0.10 | 0.23 | 0.06 | 0.07 | 0.09 | 0.18 | 0.20 | 0.24 | 0.23 | 0.07 | 0.01 | 0.21 |
| HT2 | 0.03 | 0.18 | 0.17 | 0.01 | 0.19 | 0.24 | 0.23 | 0.16 | 0.46 | 0.22 | 0.07 | 0.35 | 0.10 | 0.14 |
| SQR_BDI_T1 | -0.19 | -0.35 | -0.08 | -0.01 | -0.11 | -0.13 | -0.18 | -0.26 | -0.16 | -0.28 | -0.29 | -0.06 | -0.10 | -0.33 |
| SQR_BDI_T2 | -0.13 | -0.33 | -0.13 | -0.12 | -0.16 | -0.17 | -0.26 | -0.23 | -0.24 | -0.33 | -0.34 | -0.22 | -0.02 | -0.23 |
| SWLS_T1 | 0.10 | 0.17 | 0.00 | 0.24 | -0.01 | 0.09 | 0.22 | 0.36 | 0.21 | 0.31 | 0.32 | 0.28 | 0.12 | 0.37 |
| SWLS_T2 | 0.10 | 0.22 | 0.07 | 0.21 | 0.01 | 0.16 | 0.30 | 0.23 | 0.18 | 0.33 | 0.26 | 0.36 | 0.00 | 0.30 |

| **Pearson Correlation Matrix (Contd.)** | | | | | | | | | | | |
| --- | --- | --- | --- | --- | --- | --- | --- | --- | --- | --- | --- |
|  | **IEMAFF1** | **KONGRUENZ1** | **IEMIND2** | **IEMACH2** | **IEMAFF2** | **KONGRUENZ2** | **INKON_IND1** | **INKON_ACH1** | **INKON_AFF1** | **INKON_ALL1** | **INKON_IND2** |
| CPT1 |  |  |  |  |  |  |  |  |  |  |  |
| CAT1 |  |  |  |  |  |  |  |  |  |  |  |
| CIT1 |  |  |  |  |  |  |  |  |  |  |  |
| CPT2 |  |  |  |  |  |  |  |  |  |  |  |
| CAT2 |  |  |  |  |  |  |  |  |  |  |  |
| CIT2 |  |  |  |  |  |  |  |  |  |  |  |
| PGQ_IND_C1 |  |  |  |  |  |  |  |  |  |  |  |
| PGQ_ACH_C1 |  |  |  |  |  |  |  |  |  |  |  |
| PGQ_AFF_C1 |  |  |  |  |  |  |  |  |  |  |  |
| PGQ_IND_C2 |  |  |  |  |  |  |  |  |  |  |  |
| PGQ_ACH_C2 |  |  |  |  |  |  |  |  |  |  |  |
| PGQ_AFF_C2 |  |  |  |  |  |  |  |  |  |  |  |
| IEMIND1 |  |  |  |  |  |  |  |  |  |  |  |
| IEMACH1 |  |  |  |  |  |  |  |  |  |  |  |
| IEMAFF1 | 1.00 |  |  |  |  |  |  |  |  |  |  |
| KONGRUENZ1 | 0.74 | 1.00 |  |  |  |  |  |  |  |  |  |
| IEMIND2 | 0.07 | 0.14 | 1.00 |  |  |  |  |  |  |  |  |
| IEMACH2 | 0.20 | 0.31 | 0.18 | 1.00 |  |  |  |  |  |  |  |
| IEMAFF2 | 0.27 | 0.30 | 0.05 | 0.24 | 1.00 |  |  |  |  |  |  |
| KONGRUENZ2 | 0.27 | 0.38 | 0.61 | 0.73 | 0.64 | 1.00 |  |  |  |  |  |
| INKON_IND1 | -0.16 | -0.28 | -0.03 | 0.05 | 0.07 | 0.05 | 1.00 |  |  |  |  |
| INKON_ACH1 | -0.09 | -0.15 | -0.01 | -0.05 | 0.20 | 0.07 | 0.00 | 1.00 |  |  |  |
| INKON_AFF1 | -0.29 | -0.09 | -0.01 | 0.00 | -0.01 | -0.01 | 0.09 | 0.44 | 1.00 |  |  |
| INKON_ALL1 | -0.27 | -0.25 | -0.02 | 0.00 | 0.12 | 0.05 | 0.50 | 0.71 | 0.80 | 1.00 |  |
| INKON_IND2 | -0.09 | -0.17 | -0.37 | -0.10 | 0.09 | -0.19 | 0.26 | 0.02 | -0.05 | 0.09 | 1.00 |
| INKON_ACH2 | -0.27 | -0.19 | -0.16 | -0.41 | -0.22 | -0.40 | 0.06 | 0.10 | 0.07 | 0.11 | 0.04 |
| INKON_AFF2 | -0.17 | -0.21 | 0.03 | -0.01 | -0.46 | -0.21 | -0.06 | 0.10 | 0.19 | 0.12 | -0.03 |
| INKON_ALL2 | -0.31 | -0.34 | -0.29 | -0.31 | -0.34 | -0.47 | 0.15 | 0.13 | 0.12 | 0.19 | 0.59 |
| HT1 | 0.07 | 0.15 | 0.16 | 0.25 | -0.02 | 0.20 | 0.14 | -0.11 | 0.10 | 0.07 | 0.01 |
| HT2 | 0.32 | 0.27 | 0.15 | 0.32 | 0.20 | 0.34 | 0.15 | 0.18 | 0.13 | 0.22 | -0.11 |
| SQR_BDI_T1 | -0.10 | -0.26 | -0.01 | -0.18 | -0.11 | -0.16 | -0.20 | 0.08 | -0.18 | -0.15 | -0.11 |
| SQR_BDI_T2 | -0.28 | -0.26 | -0.20 | -0.24 | -0.16 | -0.31 | -0.08 | 0.04 | -0.02 | -0.03 | 0.09 |
| SWLS_T1 | 0.19 | 0.34 | 0.24 | 0.22 | 0.21 | 0.34 | -0.02 | -0.04 | 0.10 | 0.03 | 0.02 |
| SWLS_T2 | 0.24 | 0.27 | 0.20 | 0.17 | 0.28 | 0.32 | 0.09 | 0.02 | 0.11 | 0.11 | 0.15 |

| **Pearson Correlation Matrix (Contd.)** | | | | | | | | | |
| --- | --- | --- | --- | --- | --- | --- | --- | --- | --- |
|  | **INKON_ACH2** | **INKON_AFF2** | **INKON_ALL2** | **HT1** | **HT2** | **SQR_BDI_T1** | **SQR_BDI_T2** | **SWLS_T1** | **SWLS_T2** |
| CPT1 |  |  |  |  |  |  |  |  |  |
| CAT1 |  |  |  |  |  |  |  |  |  |
| CIT1 |  |  |  |  |  |  |  |  |  |
| CPT2 |  |  |  |  |  |  |  |  |  |
| CAT2 |  |  |  |  |  |  |  |  |  |
| CIT2 |  |  |  |  |  |  |  |  |  |
| PGQ_IND_C1 |  |  |  |  |  |  |  |  |  |
| PGQ_ACH_C1 |  |  |  |  |  |  |  |  |  |
| PGQ_AFF_C1 |  |  |  |  |  |  |  |  |  |
| PGQ_IND_C2 |  |  |  |  |  |  |  |  |  |
| PGQ_ACH_C2 |  |  |  |  |  |  |  |  |  |
| PGQ_AFF_C2 |  |  |  |  |  |  |  |  |  |
| IEMIND1 |  |  |  |  |  |  |  |  |  |
| IEMACH1 |  |  |  |  |  |  |  |  |  |
| IEMAFF1 |  |  |  |  |  |  |  |  |  |
| KONGRUENZ1 |  |  |  |  |  |  |  |  |  |
| IEMIND2 |  |  |  |  |  |  |  |  |  |
| IEMACH2 |  |  |  |  |  |  |  |  |  |
| IEMAFF2 |  |  |  |  |  |  |  |  |  |
| KONGRUENZ2 |  |  |  |  |  |  |  |  |  |
| INKON_IND1 |  |  |  |  |  |  |  |  |  |
| INKON_ACH1 |  |  |  |  |  |  |  |  |  |
| INKON_AFF1 |  |  |  |  |  |  |  |  |  |
| INKON_ALL1 |  |  |  |  |  |  |  |  |  |
| INKON_IND2 |  |  |  |  |  |  |  |  |  |
| INKON_ACH2 | 1.00 |  |  |  |  |  |  |  |  |
| INKON_AFF2 | -0.06 | 1.00 |  |  |  |  |  |  |  |
| INKON_ALL2 | 0.61 | 0.51 | 1.00 |  |  |  |  |  |  |
| HT1 | -0.13 | -0.09 | -0.13 | 1.00 |  |  |  |  |  |
| HT2 | -0.21 | 0.08 | -0.15 | 0.29 | 1.00 |  |  |  |  |
| SQR_BDI_T1 | 0.14 | 0.21 | 0.14 | -0.63 | -0.26 | 1.00 |  |  |  |
| SQR_BDI_T2 | 0.21 | 0.11 | 0.24 | -0.54 | -0.49 | 0.70 | 1.00 |  |  |
| SWLS_T1 | -0.21 | -0.17 | -0.21 | 0.46 | 0.28 | -0.47 | -0.44 | 1.00 |  |
| SWLS_T2 | -0.14 | -0.12 | -0.07 | 0.38 | 0.35 | -0.40 | -0.47 | 0.85 | 1.00 |

| **Pairwise Frequency Table** | | | | | | | | | | | | | | | |
| --- | --- | --- | --- | --- | --- | --- | --- | --- | --- | --- | --- | --- | --- | --- | --- |
|  | **CPT1** | **CAT1** | **CIT1** | **CPT2** | **CAT2** | **CIT2** | **PGQ_IND_C1** | **PGQ_ACH_C1** | **PGQ_AFF_C1** | **PGQ_IND_C2** | **PGQ_ACH_C2** | **PGQ_AFF_C2** | **IEMIND1** | **IEMACH1** | **IEMAFF1** |
| CPT1 | 74 |  |  |  |  |  |  |  |  |  |  |  |  |  |  |
| CAT1 | 74 | 74 |  |  |  |  |  |  |  |  |  |  |  |  |  |
| CIT1 | 74 | 74 | 74 |  |  |  |  |  |  |  |  |  |  |  |  |
| CPT2 | 74 | 74 | 74 | 74 |  |  |  |  |  |  |  |  |  |  |  |
| CAT2 | 74 | 74 | 74 | 74 | 74 |  |  |  |  |  |  |  |  |  |  |
| CIT2 | 74 | 74 | 74 | 74 | 74 | 74 |  |  |  |  |  |  |  |  |  |
| PGQ_IND_C1 | 74 | 74 | 74 | 74 | 74 | 74 | 74 |  |  |  |  |  |  |  |  |
| PGQ_ACH_C1 | 74 | 74 | 74 | 74 | 74 | 74 | 74 | 74 |  |  |  |  |  |  |  |
| PGQ_AFF_C1 | 74 | 74 | 74 | 74 | 74 | 74 | 74 | 74 | 74 |  |  |  |  |  |  |
| PGQ_IND_C2 | 74 | 74 | 74 | 74 | 74 | 74 | 74 | 74 | 74 | 74 |  |  |  |  |  |
| PGQ_ACH_C2 | 74 | 74 | 74 | 74 | 74 | 74 | 74 | 74 | 74 | 74 | 74 |  |  |  |  |
| PGQ_AFF_C2 | 74 | 74 | 74 | 74 | 74 | 74 | 74 | 74 | 74 | 74 | 74 | 74 |  |  |  |
| IEMIND1 | 74 | 74 | 74 | 74 | 74 | 74 | 74 | 74 | 74 | 74 | 74 | 74 | 74 |  |  |
| IEMACH1 | 74 | 74 | 74 | 74 | 74 | 74 | 74 | 74 | 74 | 74 | 74 | 74 | 74 | 74 |  |
| IEMAFF1 | 74 | 74 | 74 | 74 | 74 | 74 | 74 | 74 | 74 | 74 | 74 | 74 | 74 | 74 | 74 |
| KONGRUENZ1 | 74 | 74 | 74 | 74 | 74 | 74 | 74 | 74 | 74 | 74 | 74 | 74 | 74 | 74 | 74 |
| IEMIND2 | 74 | 74 | 74 | 74 | 74 | 74 | 74 | 74 | 74 | 74 | 74 | 74 | 74 | 74 | 74 |
| IEMACH2 | 74 | 74 | 74 | 74 | 74 | 74 | 74 | 74 | 74 | 74 | 74 | 74 | 74 | 74 | 74 |
| IEMAFF2 | 74 | 74 | 74 | 74 | 74 | 74 | 74 | 74 | 74 | 74 | 74 | 74 | 74 | 74 | 74 |
| KONGRUENZ2 | 74 | 74 | 74 | 74 | 74 | 74 | 74 | 74 | 74 | 74 | 74 | 74 | 74 | 74 | 74 |
| INKON_IND1 | 74 | 74 | 74 | 74 | 74 | 74 | 74 | 74 | 74 | 74 | 74 | 74 | 74 | 74 | 74 |
| INKON_ACH1 | 74 | 74 | 74 | 74 | 74 | 74 | 74 | 74 | 74 | 74 | 74 | 74 | 74 | 74 | 74 |
| INKON_AFF1 | 74 | 74 | 74 | 74 | 74 | 74 | 74 | 74 | 74 | 74 | 74 | 74 | 74 | 74 | 74 |
| INKON_ALL1 | 74 | 74 | 74 | 74 | 74 | 74 | 74 | 74 | 74 | 74 | 74 | 74 | 74 | 74 | 74 |
| INKON_IND2 | 74 | 74 | 74 | 74 | 74 | 74 | 74 | 74 | 74 | 74 | 74 | 74 | 74 | 74 | 74 |
| INKON_ACH2 | 74 | 74 | 74 | 74 | 74 | 74 | 74 | 74 | 74 | 74 | 74 | 74 | 74 | 74 | 74 |
| INKON_AFF2 | 74 | 74 | 74 | 74 | 74 | 74 | 74 | 74 | 74 | 74 | 74 | 74 | 74 | 74 | 74 |
| INKON_ALL2 | 74 | 74 | 74 | 74 | 74 | 74 | 74 | 74 | 74 | 74 | 74 | 74 | 74 | 74 | 74 |
| HT1 | 74 | 74 | 74 | 74 | 74 | 74 | 74 | 74 | 74 | 74 | 74 | 74 | 74 | 74 | 74 |
| HT2 | 73 | 73 | 73 | 73 | 73 | 73 | 73 | 73 | 73 | 73 | 73 | 73 | 73 | 73 | 73 |
| SQR_BDI_T1 | 74 | 74 | 74 | 74 | 74 | 74 | 74 | 74 | 74 | 74 | 74 | 74 | 74 | 74 | 74 |
| SQR_BDI_T2 | 73 | 73 | 73 | 73 | 73 | 73 | 73 | 73 | 73 | 73 | 73 | 73 | 73 | 73 | 73 |
| SWLS_T1 | 74 | 74 | 74 | 74 | 74 | 74 | 74 | 74 | 74 | 74 | 74 | 74 | 74 | 74 | 74 |
| SWLS_T2 | 74 | 74 | 74 | 74 | 74 | 74 | 74 | 74 | 74 | 74 | 74 | 74 | 74 | 74 | 74 |

| **Pairwise Frequency Table (Contd.)** | | | | | | | | | | | |
| --- | --- | --- | --- | --- | --- | --- | --- | --- | --- | --- | --- |
|  | **KONGRUENZ1** | **IEMIND2** | **IEMACH2** | **IEMAFF2** | **KONGRUENZ2** | **INKON_IND1** | **INKON_ACH1** | **INKON_AFF1** | **INKON_ALL1** | **INKON_IND2** | **INKON_ACH2** |
| CPT1 |  |  |  |  |  |  |  |  |  |  |  |
| CAT1 |  |  |  |  |  |  |  |  |  |  |  |
| CIT1 |  |  |  |  |  |  |  |  |  |  |  |
| CPT2 |  |  |  |  |  |  |  |  |  |  |  |
| CAT2 |  |  |  |  |  |  |  |  |  |  |  |
| CIT2 |  |  |  |  |  |  |  |  |  |  |  |
| PGQ_IND_C1 |  |  |  |  |  |  |  |  |  |  |  |
| PGQ_ACH_C1 |  |  |  |  |  |  |  |  |  |  |  |
| PGQ_AFF_C1 |  |  |  |  |  |  |  |  |  |  |  |
| PGQ_IND_C2 |  |  |  |  |  |  |  |  |  |  |  |
| PGQ_ACH_C2 |  |  |  |  |  |  |  |  |  |  |  |
| PGQ_AFF_C2 |  |  |  |  |  |  |  |  |  |  |  |
| IEMIND1 |  |  |  |  |  |  |  |  |  |  |  |
| IEMACH1 |  |  |  |  |  |  |  |  |  |  |  |
| IEMAFF1 |  |  |  |  |  |  |  |  |  |  |  |
| KONGRUENZ1 | 74 |  |  |  |  |  |  |  |  |  |  |
| IEMIND2 | 74 | 74 |  |  |  |  |  |  |  |  |  |
| IEMACH2 | 74 | 74 | 74 |  |  |  |  |  |  |  |  |
| IEMAFF2 | 74 | 74 | 74 | 74 |  |  |  |  |  |  |  |
| KONGRUENZ2 | 74 | 74 | 74 | 74 | 74 |  |  |  |  |  |  |
| INKON_IND1 | 74 | 74 | 74 | 74 | 74 | 74 |  |  |  |  |  |
| INKON_ACH1 | 74 | 74 | 74 | 74 | 74 | 74 | 74 |  |  |  |  |
| INKON_AFF1 | 74 | 74 | 74 | 74 | 74 | 74 | 74 | 74 |  |  |  |
| INKON_ALL1 | 74 | 74 | 74 | 74 | 74 | 74 | 74 | 74 | 74 |  |  |
| INKON_IND2 | 74 | 74 | 74 | 74 | 74 | 74 | 74 | 74 | 74 | 74 |  |
| INKON_ACH2 | 74 | 74 | 74 | 74 | 74 | 74 | 74 | 74 | 74 | 74 | 74 |
| INKON_AFF2 | 74 | 74 | 74 | 74 | 74 | 74 | 74 | 74 | 74 | 74 | 74 |
| INKON_ALL2 | 74 | 74 | 74 | 74 | 74 | 74 | 74 | 74 | 74 | 74 | 74 |
| HT1 | 74 | 74 | 74 | 74 | 74 | 74 | 74 | 74 | 74 | 74 | 74 |
| HT2 | 73 | 73 | 73 | 73 | 73 | 73 | 73 | 73 | 73 | 73 | 73 |
| SQR_BDI_T1 | 74 | 74 | 74 | 74 | 74 | 74 | 74 | 74 | 74 | 74 | 74 |
| SQR_BDI_T2 | 73 | 73 | 73 | 73 | 73 | 73 | 73 | 73 | 73 | 73 | 73 |
| SWLS_T1 | 74 | 74 | 74 | 74 | 74 | 74 | 74 | 74 | 74 | 74 | 74 |
| SWLS_T2 | 74 | 74 | 74 | 74 | 74 | 74 | 74 | 74 | 74 | 74 | 74 |

| **Pairwise Frequency Table (Contd.)** | | | | | | | | |
| --- | --- | --- | --- | --- | --- | --- | --- | --- |
|  | **INKON_AFF2** | **INKON_ALL2** | **HT1** | **HT2** | **SQR_BDI_T1** | **SQR_BDI_T2** | **SWLS_T1** | **SWLS_T2** |
| CPT1 |  |  |  |  |  |  |  |  |
| CAT1 |  |  |  |  |  |  |  |  |
| CIT1 |  |  |  |  |  |  |  |  |
| CPT2 |  |  |  |  |  |  |  |  |
| CAT2 |  |  |  |  |  |  |  |  |
| CIT2 |  |  |  |  |  |  |  |  |
| PGQ_IND_C1 |  |  |  |  |  |  |  |  |
| PGQ_ACH_C1 |  |  |  |  |  |  |  |  |
| PGQ_AFF_C1 |  |  |  |  |  |  |  |  |
| PGQ_IND_C2 |  |  |  |  |  |  |  |  |
| PGQ_ACH_C2 |  |  |  |  |  |  |  |  |
| PGQ_AFF_C2 |  |  |  |  |  |  |  |  |
| IEMIND1 |  |  |  |  |  |  |  |  |
| IEMACH1 |  |  |  |  |  |  |  |  |
| IEMAFF1 |  |  |  |  |  |  |  |  |
| KONGRUENZ1 |  |  |  |  |  |  |  |  |
| IEMIND2 |  |  |  |  |  |  |  |  |
| IEMACH2 |  |  |  |  |  |  |  |  |
| IEMAFF2 |  |  |  |  |  |  |  |  |
| KONGRUENZ2 |  |  |  |  |  |  |  |  |
| INKON_IND1 |  |  |  |  |  |  |  |  |
| INKON_ACH1 |  |  |  |  |  |  |  |  |
| INKON_AFF1 |  |  |  |  |  |  |  |  |
| INKON_ALL1 |  |  |  |  |  |  |  |  |
| INKON_IND2 |  |  |  |  |  |  |  |  |
| INKON_ACH2 |  |  |  |  |  |  |  |  |
| INKON_AFF2 | 74 |  |  |  |  |  |  |  |
| INKON_ALL2 | 74 | 74 |  |  |  |  |  |  |
| HT1 | 74 | 74 | 74 |  |  |  |  |  |
| HT2 | 73 | 73 | 73 | 73 |  |  |  |  |
| SQR_BDI_T1 | 74 | 74 | 74 | 73 | 74 |  |  |  |
| SQR_BDI_T2 | 73 | 73 | 73 | 72 | 73 | 73 |  |  |
| SWLS_T1 | 74 | 74 | 74 | 73 | 74 | 73 | 74 |  |
| SWLS_T2 | 74 | 74 | 74 | 73 | 74 | 73 | 74 | 74 |

WARNING Correlation matrix is not positive definite.

Individual significance tests are suspect.

| **Matrix of Probabilities** | | | | | | | | | | | | | | | |
| --- | --- | --- | --- | --- | --- | --- | --- | --- | --- | --- | --- | --- | --- | --- | --- |
|  | **CPT1** | **CAT1** | **CIT1** | **CPT2** | **CAT2** | **CIT2** | **PGQ_IND_C1** | **PGQ_ACH_C1** | **PGQ_AFF_C1** | **PGQ_IND_C2** | **PGQ_ACH_C2** | **PGQ_AFF_C2** | **IEMIND1** | **IEMACH1** | **IEMAFF1** |
| CPT1 | 0.00 |  |  |  |  |  |  |  |  |  |  |  |  |  |  |
| CAT1 | 0.13 | 0.00 |  |  |  |  |  |  |  |  |  |  |  |  |  |
| CIT1 | 0.47 | 0.00 | 0.00 |  |  |  |  |  |  |  |  |  |  |  |  |
| CPT2 | 0.02 | 0.27 | 0.47 | 0.00 |  |  |  |  |  |  |  |  |  |  |  |
| CAT2 | 0.78 | 0.02 | 0.00 | 0.41 | 0.00 |  |  |  |  |  |  |  |  |  |  |
| CIT2 | 0.78 | 0.00 | 0.00 | 0.03 | 0.03 | 0.00 |  |  |  |  |  |  |  |  |  |
| PGQ_IND_C1 | 0.79 | 0.42 | 0.19 | 0.16 | 0.25 | 0.39 | 0.00 |  |  |  |  |  |  |  |  |
| PGQ_ACH_C1 | 0.78 | 0.15 | 0.68 | 0.75 | 0.88 | 0.15 | 0.06 | 0.00 |  |  |  |  |  |  |  |
| PGQ_AFF_C1 | 0.02 | 0.22 | 0.92 | 0.64 | 0.26 | 0.94 | 0.10 | 0.00 | 0.00 |  |  |  |  |  |  |
| PGQ_IND_C2 | 0.39 | 0.23 | 0.39 | 0.96 | 0.81 | 0.73 | 0.00 | 0.01 | 0.01 | 0.00 |  |  |  |  |  |
| PGQ_ACH_C2 | 0.38 | 0.05 | 0.58 | 0.58 | 0.86 | 0.48 | 0.00 | 0.00 | 0.00 | 0.00 | 0.00 |  |  |  |  |
| PGQ_AFF_C2 | 0.54 | 0.49 | 0.87 | 0.98 | 0.88 | 0.56 | 0.00 | 0.01 | 0.00 | 0.00 | 0.00 | 0.00 |  |  |  |
| IEMIND1 | 0.00 | 0.22 | 0.16 | 0.83 | 0.47 | 0.63 | 0.01 | 0.65 | 0.14 | 0.08 | 0.37 | 0.29 | 0.00 |  |  |
| IEMACH1 | 0.82 | 0.00 | 0.02 | 0.71 | 0.25 | 0.27 | 0.19 | 0.00 | 0.07 | 0.02 | 0.00 | 0.65 | 0.73 | 0.00 |  |
| IEMAFF1 | 0.94 | 0.00 | 0.00 | 0.77 | 0.90 | 0.03 | 0.05 | 0.03 | 0.00 | 0.01 | 0.01 | 0.00 | 0.53 | 0.00 | 0.00 |
| KONGRUENZ1 | 0.02 | 0.00 | 0.00 | 0.67 | 0.32 | 0.07 | 0.00 | 0.00 | 0.00 | 0.00 | 0.00 | 0.03 | 0.00 | 0.00 | 0.00 |
| IEMIND2 | 0.16 | 0.05 | 0.57 | 0.00 | 0.83 | 0.50 | 0.44 | 0.58 | 0.07 | 0.00 | 0.09 | 0.24 | 0.32 | 0.40 | 0.53 |
| IEMACH2 | 0.41 | 0.07 | 0.04 | 0.85 | 0.00 | 0.00 | 0.45 | 0.01 | 0.65 | 0.03 | 0.00 | 0.22 | 0.31 | 0.01 | 0.08 |
| IEMAFF2 | 0.31 | 0.01 | 0.02 | 0.05 | 0.24 | 0.00 | 0.00 | 0.03 | 0.56 | 0.10 | 0.02 | 0.00 | 0.32 | 0.06 | 0.02 |
| KONGRUENZ2 | 0.54 | 0.00 | 0.05 | 0.17 | 0.00 | 0.00 | 0.02 | 0.01 | 0.16 | 0.00 | 0.00 | 0.00 | 0.13 | 0.01 | 0.02 |
| INKON_IND1 | 0.89 | 0.63 | 0.20 | 0.99 | 0.92 | 0.33 | 0.05 | 0.71 | 0.79 | 0.96 | 0.66 | 0.49 | 0.00 | 0.92 | 0.18 |
| INKON_ACH1 | 0.61 | 0.58 | 0.48 | 0.60 | 0.34 | 0.16 | 0.19 | 0.50 | 0.80 | 0.88 | 0.96 | 0.09 | 0.45 | 0.01 | 0.46 |
| INKON_AFF1 | 0.49 | 0.69 | 0.40 | 0.19 | 0.12 | 0.37 | 0.44 | 0.95 | 0.83 | 0.31 | 0.85 | 0.24 | 0.15 | 0.55 | 0.01 |
| INKON_ALL1 | 0.58 | 0.79 | 0.82 | 0.33 | 0.17 | 0.11 | 0.85 | 0.86 | 0.91 | 0.54 | 0.78 | 0.85 | 0.63 | 0.15 | 0.02 |
| INKON_IND2 | 1.00 | 0.25 | 0.78 | 0.51 | 0.68 | 0.82 | 0.91 | 0.71 | 0.19 | 0.22 | 0.23 | 0.29 | 0.21 | 0.35 | 0.43 |
| INKON_ACH2 | 0.57 | 0.39 | 0.76 | 0.50 | 0.69 | 0.06 | 0.91 | 0.19 | 0.53 | 0.61 | 0.15 | 0.01 | 0.89 | 0.37 | 0.02 |
| INKON_AFF2 | 0.63 | 0.32 | 0.37 | 0.36 | 0.97 | 0.56 | 0.12 | 0.34 | 0.90 | 0.15 | 0.18 | 0.29 | 0.65 | 0.08 | 0.15 |
| INKON_ALL2 | 0.53 | 0.08 | 0.39 | 0.78 | 0.97 | 0.10 | 0.39 | 0.26 | 0.28 | 0.07 | 0.02 | 0.13 | 0.28 | 0.04 | 0.01 |
| HT1 | 0.86 | 0.11 | 0.41 | 0.05 | 0.63 | 0.58 | 0.44 | 0.12 | 0.09 | 0.04 | 0.05 | 0.57 | 0.92 | 0.08 | 0.54 |
| HT2 | 0.80 | 0.14 | 0.15 | 0.91 | 0.10 | 0.04 | 0.05 | 0.17 | 0.00 | 0.07 | 0.56 | 0.00 | 0.42 | 0.23 | 0.01 |
| SQR_BDI_T1 | 0.10 | 0.00 | 0.49 | 0.94 | 0.34 | 0.25 | 0.13 | 0.03 | 0.18 | 0.01 | 0.01 | 0.61 | 0.42 | 0.00 | 0.40 |
| SQR_BDI_T2 | 0.28 | 0.00 | 0.26 | 0.32 | 0.19 | 0.16 | 0.02 | 0.06 | 0.04 | 0.00 | 0.00 | 0.06 | 0.87 | 0.05 | 0.01 |
| SWLS_T1 | 0.38 | 0.14 | 1.00 | 0.04 | 0.92 | 0.45 | 0.07 | 0.00 | 0.07 | 0.01 | 0.00 | 0.02 | 0.32 | 0.00 | 0.11 |
| SWLS_T2 | 0.40 | 0.06 | 0.54 | 0.07 | 0.92 | 0.16 | 0.01 | 0.04 | 0.12 | 0.00 | 0.02 | 0.00 | 0.99 | 0.01 | 0.04 |

| **Matrix of Probabilities (Contd.)** | | | | | | | | | | | |
| --- | --- | --- | --- | --- | --- | --- | --- | --- | --- | --- | --- |
|  | **KONGRUENZ1** | **IEMIND2** | **IEMACH2** | **IEMAFF2** | **KONGRUENZ2** | **INKON_IND1** | **INKON_ACH1** | **INKON_AFF1** | **INKON_ALL1** | **INKON_IND2** | **INKON_ACH2** |
| CPT1 |  |  |  |  |  |  |  |  |  |  |  |
| CAT1 |  |  |  |  |  |  |  |  |  |  |  |
| CIT1 |  |  |  |  |  |  |  |  |  |  |  |
| CPT2 |  |  |  |  |  |  |  |  |  |  |  |
| CAT2 |  |  |  |  |  |  |  |  |  |  |  |
| CIT2 |  |  |  |  |  |  |  |  |  |  |  |
| PGQ_IND_C1 |  |  |  |  |  |  |  |  |  |  |  |
| PGQ_ACH_C1 |  |  |  |  |  |  |  |  |  |  |  |
| PGQ_AFF_C1 |  |  |  |  |  |  |  |  |  |  |  |
| PGQ_IND_C2 |  |  |  |  |  |  |  |  |  |  |  |
| PGQ_ACH_C2 |  |  |  |  |  |  |  |  |  |  |  |
| PGQ_AFF_C2 |  |  |  |  |  |  |  |  |  |  |  |
| IEMIND1 |  |  |  |  |  |  |  |  |  |  |  |
| IEMACH1 |  |  |  |  |  |  |  |  |  |  |  |
| IEMAFF1 |  |  |  |  |  |  |  |  |  |  |  |
| KONGRUENZ1 | 0.00 |  |  |  |  |  |  |  |  |  |  |
| IEMIND2 | 0.22 | 0.00 |  |  |  |  |  |  |  |  |  |
| IEMACH2 | 0.01 | 0.13 | 0.00 |  |  |  |  |  |  |  |  |
| IEMAFF2 | 0.01 | 0.65 | 0.04 | 0.00 |  |  |  |  |  |  |  |
| KONGRUENZ2 | 0.00 | 0.00 | 0.00 | 0.00 | 0.00 |  |  |  |  |  |  |
| INKON_IND1 | 0.02 | 0.78 | 0.69 | 0.54 | 0.70 | 0.00 |  |  |  |  |  |
| INKON_ACH1 | 0.21 | 0.94 | 0.68 | 0.09 | 0.56 | 0.99 | 0.00 |  |  |  |  |
| INKON_AFF1 | 0.42 | 0.94 | 0.99 | 0.93 | 0.93 | 0.43 | 0.00 | 0.00 |  |  |  |
| INKON_ALL1 | 0.03 | 0.85 | 0.99 | 0.31 | 0.69 | 0.00 | 0.00 | 0.00 | 0.00 |  |  |
| INKON_IND2 | 0.14 | 0.00 | 0.40 | 0.46 | 0.11 | 0.03 | 0.85 | 0.65 | 0.42 | 0.00 |  |
| INKON_ACH2 | 0.10 | 0.19 | 0.00 | 0.06 | 0.00 | 0.62 | 0.38 | 0.57 | 0.34 | 0.73 | 0.00 |
| INKON_AFF2 | 0.07 | 0.77 | 0.94 | 0.00 | 0.07 | 0.59 | 0.40 | 0.11 | 0.29 | 0.80 | 0.62 |
| INKON_ALL2 | 0.00 | 0.01 | 0.01 | 0.00 | 0.00 | 0.21 | 0.26 | 0.32 | 0.10 | 0.00 | 0.00 |
| HT1 | 0.21 | 0.17 | 0.03 | 0.86 | 0.09 | 0.24 | 0.36 | 0.41 | 0.58 | 0.93 | 0.26 |
| HT2 | 0.02 | 0.21 | 0.01 | 0.09 | 0.00 | 0.21 | 0.12 | 0.27 | 0.06 | 0.37 | 0.08 |
| SQR_BDI_T1 | 0.02 | 0.94 | 0.12 | 0.33 | 0.18 | 0.09 | 0.50 | 0.13 | 0.20 | 0.37 | 0.25 |
| SQR_BDI_T2 | 0.02 | 0.09 | 0.04 | 0.17 | 0.01 | 0.48 | 0.71 | 0.85 | 0.81 | 0.44 | 0.08 |
| SWLS_T1 | 0.00 | 0.04 | 0.07 | 0.07 | 0.00 | 0.85 | 0.73 | 0.39 | 0.81 | 0.87 | 0.08 |
| SWLS_T2 | 0.02 | 0.09 | 0.16 | 0.01 | 0.00 | 0.43 | 0.90 | 0.33 | 0.34 | 0.19 | 0.23 |

| **Matrix of Probabilities (Contd.)** | | | | | | | | |
| --- | --- | --- | --- | --- | --- | --- | --- | --- |
|  | **INKON_AFF2** | **INKON_ALL2** | **HT1** | **HT2** | **SQR_BDI_T1** | **SQR_BDI_T2** | **SWLS_T1** | **SWLS_T2** |
| CPT1 |  |  |  |  |  |  |  |  |
| CAT1 |  |  |  |  |  |  |  |  |
| CIT1 |  |  |  |  |  |  |  |  |
| CPT2 |  |  |  |  |  |  |  |  |
| CAT2 |  |  |  |  |  |  |  |  |
| CIT2 |  |  |  |  |  |  |  |  |
| PGQ_IND_C1 |  |  |  |  |  |  |  |  |
| PGQ_ACH_C1 |  |  |  |  |  |  |  |  |
| PGQ_AFF_C1 |  |  |  |  |  |  |  |  |
| PGQ_IND_C2 |  |  |  |  |  |  |  |  |
| PGQ_ACH_C2 |  |  |  |  |  |  |  |  |
| PGQ_AFF_C2 |  |  |  |  |  |  |  |  |
| IEMIND1 |  |  |  |  |  |  |  |  |
| IEMACH1 |  |  |  |  |  |  |  |  |
| IEMAFF1 |  |  |  |  |  |  |  |  |
| KONGRUENZ1 |  |  |  |  |  |  |  |  |
| IEMIND2 |  |  |  |  |  |  |  |  |
| IEMACH2 |  |  |  |  |  |  |  |  |
| IEMAFF2 |  |  |  |  |  |  |  |  |
| KONGRUENZ2 |  |  |  |  |  |  |  |  |
| INKON_IND1 |  |  |  |  |  |  |  |  |
| INKON_ACH1 |  |  |  |  |  |  |  |  |
| INKON_AFF1 |  |  |  |  |  |  |  |  |
| INKON_ALL1 |  |  |  |  |  |  |  |  |
| INKON_IND2 |  |  |  |  |  |  |  |  |
| INKON_ACH2 |  |  |  |  |  |  |  |  |
| INKON_AFF2 | 0.00 |  |  |  |  |  |  |  |
| INKON_ALL2 | 0.00 | 0.00 |  |  |  |  |  |  |
| HT1 | 0.44 | 0.28 | 0.00 |  |  |  |  |  |
| HT2 | 0.51 | 0.22 | 0.01 | 0.00 |  |  |  |  |
| SQR_BDI_T1 | 0.07 | 0.23 | 0.00 | 0.03 | 0.00 |  |  |  |
| SQR_BDI_T2 | 0.36 | 0.04 | 0.00 | 0.00 | 0.00 | 0.00 |  |  |
| SWLS_T1 | 0.14 | 0.07 | 0.00 | 0.02 | 0.00 | 0.00 | 0.00 |  |
| SWLS_T2 | 0.31 | 0.58 | 0.00 | 0.00 | 0.00 | 0.00 | 0.00 | 0.00 |

> *format 12, 8*

> *corr*

> *pearson cpt1 cat1 cit1 cpt2 cat2 cit2 PGQ_IND_C1 PGQ_ach_C1 PGQ_AFF_C1 PGQ_IND_C2 PGQ_ach_C2 PGQ_AFF_C2 iemind1 iemach1 iemaff1 kongruenz1 iemind2 iemach2 iemaff2
kongruenz2 Inkon_ind1 Inkon_ach1 Inkon_aff1 inkon_all1 Inkon_ind2 Inkon_ach2 Inkon_aff2 inkon_all2 ht1 ht2 sqr_bdi_t1 sqr_bdi_t2 swls_t1 swls_t2 /prob pairwise*

[▼Correlation: Pearson](file:///\\Untitled.syo)

| **Pearson Correlation Matrix** | | | | | | | | | |
| --- | --- | --- | --- | --- | --- | --- | --- | --- | --- |
|  | **CPT1** | **CAT1** | **CIT1** | **CPT2** | **CAT2** | **CIT2** | **PGQ_IND_C1** | **PGQ_ACH_C1** | **PGQ_AFF_C1** |
| CPT1 | 1.00000000 |  |  |  |  |  |  |  |  |
| CAT1 | 0.17621487 | 1.00000000 |  |  |  |  |  |  |  |
| CIT1 | -0.08509424 | 0.34607600 | 1.00000000 |  |  |  |  |  |  |
| CPT2 | 0.26853607 | 0.13019192 | -0.08530783 | 1.00000000 |  |  |  |  |  |
| CAT2 | 0.03375409 | 0.27910366 | 0.40256530 | -0.09690142 | 1.00000000 |  |  |  |  |
| CIT2 | -0.03292017 | 0.32381179 | 0.38057131 | -0.25675681 | 0.24873713 | 1.00000000 |  |  |  |
| PGQ_IND_C1 | -0.03183335 | 0.09495503 | 0.15360413 | -0.16675780 | 0.13475730 | 0.10219514 | 1.00000000 |  |  |
| PGQ_ACH_C1 | -0.03253972 | 0.16952784 | 0.04794948 | -0.03761716 | -0.01811908 | 0.16809784 | 0.22400917 | 1.00000000 |  |
| PGQ_AFF_C1 | 0.27631092 | 0.14577432 | -0.01257196 | 0.05564845 | -0.13322592 | 0.00946587 | 0.19243220 | 0.32544965 | 1.00000000 |
| PGQ_IND_C2 | 0.10234458 | 0.14039193 | 0.10207170 | -0.00653174 | 0.02794316 | -0.04075493 | 0.58168256 | 0.32016904 | 0.28342543 |
| PGQ_ACH_C2 | 0.10310048 | 0.22526480 | 0.06593160 | 0.06533602 | 0.02088023 | 0.08327773 | 0.35005166 | 0.65751074 | 0.37152072 |
| PGQ_AFF_C2 | -0.07181397 | 0.08147769 | 0.01996246 | -0.00360714 | -0.01741185 | 0.06922981 | 0.52602479 | 0.30604764 | 0.39186359 |
| IEMIND1 | 0.52317353 | 0.14449764 | 0.16404510 | 0.02502096 | 0.08497349 | 0.05618870 | 0.28373503 | 0.05385089 | 0.17461230 |
| IEMACH1 | 0.02721682 | 0.63625407 | 0.27977627 | 0.04352994 | 0.13481510 | 0.13012655 | 0.15545664 | 0.51634907 | 0.20880606 |
| IEMAFF1 | -0.00826472 | 0.35102240 | 0.58347044 | 0.03389204 | 0.01448715 | 0.24903682 | 0.22449943 | 0.24735482 | 0.41684522 |
| KONGRUENZ1 | 0.26457295 | 0.56725059 | 0.50447334 | 0.05091613 | 0.11786416 | 0.21412012 | 0.32609376 | 0.41147843 | 0.39294394 |
| IEMIND2 | 0.16472385 | 0.23246377 | -0.06669227 | 0.57390027 | 0.02517596 | -0.07971342 | 0.09017742 | 0.06453444 | 0.21295447 |
| IEMACH2 | 0.09789853 | 0.21257759 | 0.24048289 | -0.02265515 | 0.54698798 | 0.35890900 | 0.08880161 | 0.31495202 | 0.05305330 |
| IEMAFF2 | -0.12021748 | 0.28836555 | 0.27416357 | -0.22450920 | 0.13742906 | 0.59273499 | 0.37590532 | 0.25683841 | 0.06917134 |
| KONGRUENZ2 | 0.07297892 | 0.36796496 | 0.22774029 | 0.16075563 | 0.36524886 | 0.44033702 | 0.27644830 | 0.32278708 | 0.16697331 |
| INKON_IND1 | -0.01673613 | -0.05715454 | -0.15042883 | -0.00198988 | 0.01221963 | 0.11497236 | -0.22524980 | 0.04336805 | -0.03172374 |
| INKON_ACH1 | 0.06017750 | 0.06504160 | 0.08299594 | -0.06137860 | 0.11361054 | 0.16500869 | 0.15533132 | -0.07941105 | 0.03018841 |
| INKON_AFF1 | 0.08190469 | 0.04752897 | 0.09891385 | -0.15425422 | 0.18319946 | 0.10671646 | 0.09038366 | -0.00745636 | -0.02490935 |
| INKON_ALL1 | 0.06614653 | 0.03118148 | 0.02660198 | -0.11545373 | 0.16037033 | 0.18728223 | 0.02198005 | -0.02160676 | -0.01407283 |
| INKON_IND2 | 0.00057272 | -0.13488338 | -0.03281326 | -0.07841746 | -0.04842235 | -0.02704890 | 0.01379434 | 0.04398034 | -0.15464112 |
| INKON_ACH2 | 0.06717200 | -0.10209591 | -0.03564632 | -0.07960258 | 0.04733117 | -0.22348540 | -0.01348743 | -0.15327510 | -0.07335108 |
| INKON_AFF2 | 0.05748697 | -0.11766056 | -0.10656685 | 0.10840271 | 0.00469129 | -0.06864679 | -0.18111917 | -0.11325927 | 0.01413267 |
| INKON_ALL2 | 0.07395718 | -0.20703949 | -0.10071717 | -0.03374524 | 0.00374448 | -0.19190286 | -0.10183844 | -0.13247325 | -0.12689975 |
| HT1 | 0.02011682 | 0.18485151 | 0.09802490 | 0.22926730 | 0.05743507 | 0.06591080 | 0.09157240 | 0.18107821 | 0.19789851 |
| HT2 | 0.02946795 | 0.17644743 | 0.17172663 | 0.01395519 | 0.19346887 | 0.24219316 | 0.23428618 | 0.16137820 | 0.45541651 |
| SQR_BDI_T1 | -0.19024020 | -0.34721365 | -0.08102944 | -0.00949839 | -0.11239768 | -0.13418925 | -0.17982515 | -0.25846111 | -0.15589193 |
| SQR_BDI_T2 | -0.12918388 | -0.32949060 | -0.13428486 | -0.11804846 | -0.15606919 | -0.16529354 | -0.26289552 | -0.22550062 | -0.24423916 |
| SWLS_T1 | 0.10398236 | 0.17133881 | 0.00060893 | 0.23831150 | -0.01119291 | 0.08969133 | 0.21550017 | 0.35808227 | 0.21191084 |
| SWLS_T2 | 0.09971631 | 0.21739629 | 0.07180138 | 0.21104133 | 0.01192379 | 0.16374584 | 0.29625946 | 0.23474412 | 0.18106567 |

| **Pearson Correlation Matrix (Contd.)** | | | | | | | | | |
| --- | --- | --- | --- | --- | --- | --- | --- | --- | --- |
|  | **PGQ_IND_C2** | **PGQ_ACH_C2** | **PGQ_AFF_C2** | **IEMIND1** | **IEMACH1** | **IEMAFF1** | **KONGRUENZ1** | **IEMIND2** | **IEMACH2** |
| CPT1 |  |  |  |  |  |  |  |  |  |
| CAT1 |  |  |  |  |  |  |  |  |  |
| CIT1 |  |  |  |  |  |  |  |  |  |
| CPT2 |  |  |  |  |  |  |  |  |  |
| CAT2 |  |  |  |  |  |  |  |  |  |
| CIT2 |  |  |  |  |  |  |  |  |  |
| PGQ_IND_C1 |  |  |  |  |  |  |  |  |  |
| PGQ_ACH_C1 |  |  |  |  |  |  |  |  |  |
| PGQ_AFF_C1 |  |  |  |  |  |  |  |  |  |
| PGQ_IND_C2 | 1.00000000 |  |  |  |  |  |  |  |  |
| PGQ_ACH_C2 | 0.47084673 | 1.00000000 |  |  |  |  |  |  |  |
| PGQ_AFF_C2 | 0.38887831 | 0.39390325 | 1.00000000 |  |  |  |  |  |  |
| IEMIND1 | 0.20195181 | 0.10463928 | 0.12556947 | 1.00000000 |  |  |  |  |  |
| IEMACH1 | 0.28099206 | 0.41092777 | 0.05375169 | 0.04018223 | 1.00000000 |  |  |  |  |
| IEMAFF1 | 0.28413600 | 0.30924548 | 0.32718528 | 0.07423974 | 0.42641386 | 1.00000000 |  |  |  |
| KONGRUENZ1 | 0.37974005 | 0.41151596 | 0.24600111 | 0.54285167 | 0.74042922 | 0.73655828 | 1.00000000 |  |  |
| IEMIND2 | 0.44113361 | 0.19979513 | 0.13709818 | 0.11679454 | 0.09965193 | 0.07423974 | 0.14390338 | 1.00000000 |  |
| IEMACH2 | 0.24798639 | 0.33297295 | 0.14297764 | 0.11881276 | 0.29562387 | 0.20460759 | 0.30864194 | 0.17862326 | 1.00000000 |
| IEMAFF2 | 0.19309322 | 0.27116201 | 0.48295949 | 0.11679454 | 0.21859133 | 0.26588185 | 0.29734503 | 0.05370844 | 0.23843376 |
| KONGRUENZ2 | 0.44241518 | 0.40605141 | 0.38071529 | 0.17725794 | 0.31117286 | 0.27456518 | 0.37870415 | 0.61345563 | 0.72690441 |
| INKON_IND1 | -0.00540600 | -0.05170044 | -0.08219587 | -0.43007499 | 0.01210130 | -0.15834487 | -0.27903492 | -0.03244587 | 0.04787290 |
| INKON_ACH1 | 0.01782820 | 0.00629900 | 0.19751830 | 0.08946314 | -0.29026653 | -0.08753141 | -0.14831778 | -0.00871843 | -0.04858890 |
| INKON_AFF1 | 0.11975912 | -0.02239819 | -0.13909976 | 0.16845804 | -0.07066369 | -0.29149883 | -0.09455435 | -0.00819006 | -0.00150967 |
| INKON_ALL1 | 0.07272250 | -0.03264248 | -0.02284283 | -0.05625758 | -0.16850419 | -0.27392264 | -0.24591292 | -0.02307860 | -0.00200176 |
| INKON_IND2 | -0.14281178 | -0.14240544 | 0.12568161 | -0.14864853 | -0.11000325 | -0.09370701 | -0.17409252 | -0.36522817 | -0.09872760 |
| INKON_ACH2 | -0.05997845 | -0.16876041 | -0.28992905 | -0.01708748 | -0.10583967 | -0.26755017 | -0.19146026 | -0.15521976 | -0.40876050 |
| INKON_AFF2 | -0.16910473 | -0.15841663 | -0.12586264 | -0.05423992 | -0.20379597 | -0.16798472 | -0.21225050 | 0.03388223 | -0.00929389 |
| INKON_ALL2 | -0.21450811 | -0.27533298 | -0.17642538 | -0.12707071 | -0.24320934 | -0.31336904 | -0.33786047 | -0.28831995 | -0.31437042 |
| HT1 | 0.23898403 | 0.22720332 | 0.06651791 | 0.01243424 | 0.20535983 | 0.07294304 | 0.14706300 | 0.16272280 | 0.24559633 |
| HT2 | 0.21683321 | 0.06891933 | 0.34660635 | 0.09537742 | 0.14182784 | 0.32431307 | 0.27405030 | 0.14828435 | 0.32264459 |
| SQR_BDI_T1 | -0.28419217 | -0.28648566 | -0.05990968 | -0.09610193 | -0.32917890 | -0.09903329 | -0.26419177 | -0.00878495 | -0.18222087 |
| SQR_BDI_T2 | -0.32864954 | -0.34001748 | -0.22118489 | -0.01879836 | -0.23055328 | -0.28386908 | -0.26297661 | -0.19810831 | -0.24369180 |
| SWLS_T1 | 0.30715706 | 0.32439282 | 0.27576373 | 0.11764561 | 0.36541674 | 0.18714457 | 0.33570036 | 0.23721479 | 0.21526885 |
| SWLS_T2 | 0.33137008 | 0.26325284 | 0.35730301 | 0.00090323 | 0.29971144 | 0.23947465 | 0.27014415 | 0.19585049 | 0.16570032 |

| **Pearson Correlation Matrix (Contd.)** | | | | | | | | | |
| --- | --- | --- | --- | --- | --- | --- | --- | --- | --- |
|  | **IEMAFF2** | **KONGRUENZ2** | **INKON_IND1** | **INKON_ACH1** | **INKON_AFF1** | **INKON_ALL1** | **INKON_IND2** | **INKON_ACH2** | **INKON_AFF2** |
| CPT1 |  |  |  |  |  |  |  |  |  |
| CAT1 |  |  |  |  |  |  |  |  |  |
| CIT1 |  |  |  |  |  |  |  |  |  |
| CPT2 |  |  |  |  |  |  |  |  |  |
| CAT2 |  |  |  |  |  |  |  |  |  |
| CIT2 |  |  |  |  |  |  |  |  |  |
| PGQ_IND_C1 |  |  |  |  |  |  |  |  |  |
| PGQ_ACH_C1 |  |  |  |  |  |  |  |  |  |
| PGQ_AFF_C1 |  |  |  |  |  |  |  |  |  |
| PGQ_IND_C2 |  |  |  |  |  |  |  |  |  |
| PGQ_ACH_C2 |  |  |  |  |  |  |  |  |  |
| PGQ_AFF_C2 |  |  |  |  |  |  |  |  |  |
| IEMIND1 |  |  |  |  |  |  |  |  |  |
| IEMACH1 |  |  |  |  |  |  |  |  |  |
| IEMAFF1 |  |  |  |  |  |  |  |  |  |
| KONGRUENZ1 |  |  |  |  |  |  |  |  |  |
| IEMIND2 |  |  |  |  |  |  |  |  |  |
| IEMACH2 |  |  |  |  |  |  |  |  |  |
| IEMAFF2 | 1.00000000 |  |  |  |  |  |  |  |  |
| KONGRUENZ2 | 0.64461261 | 1.00000000 |  |  |  |  |  |  |  |
| INKON_IND1 | 0.07322539 | 0.04507854 | 1.00000000 |  |  |  |  |  |  |
| INKON_ACH1 | 0.19985078 | 0.06908515 | 0.00168593 | 1.00000000 |  |  |  |  |  |
| INKON_AFF1 | -0.01038586 | -0.00996071 | 0.09318148 | 0.44027029 | 1.00000000 |  |  |  |  |
| INKON_ALL1 | 0.11941341 | 0.04653508 | 0.49869865 | 0.70976738 | 0.80388189 | 1.00000000 |  |  |  |
| INKON_IND2 | 0.08651502 | -0.18908090 | 0.25500564 | 0.02235095 | -0.05290416 | 0.09451229 | 1.00000000 |  |  |
| INKON_ACH2 | -0.22091694 | -0.39870134 | 0.05806777 | 0.10366157 | 0.06783678 | 0.11178472 | 0.04157824 | 1.00000000 |  |
| INKON_AFF2 | -0.45635320 | -0.21349149 | -0.06352562 | 0.09884497 | 0.18977462 | 0.12342962 | -0.02982978 | -0.05817173 | 1.00000000 |
| INKON_ALL2 | -0.34182524 | -0.47498065 | 0.14795203 | 0.13224976 | 0.11752701 | 0.19285147 | 0.58837261 | 0.60786805 | 0.50736478 |
| HT1 | -0.02130400 | 0.19778200 | 0.13871685 | -0.10831977 | 0.09661934 | 0.06563605 | 0.01027525 | -0.13349599 | -0.09077534 |
| HT2 | 0.20290356 | 0.33901612 | 0.14729586 | 0.18482348 | 0.13073134 | 0.22434020 | -0.10724768 | -0.20721700 | 0.07750872 |
| SQR_BDI_T1 | -0.11482757 | -0.15597376 | -0.19842212 | 0.07998752 | -0.17732113 | -0.15044536 | -0.10558325 | 0.13507553 | 0.21296047 |
| SQR_BDI_T2 | -0.16248579 | -0.31066741 | -0.08321108 | 0.04383179 | -0.02240976 | -0.02919015 | 0.09098458 | 0.20517930 | 0.10900637 |
| SWLS_T1 | 0.21443971 | 0.33520283 | -0.02165040 | -0.04018221 | 0.10153532 | 0.02843475 | 0.01971241 | -0.20733582 | -0.17396591 |
| SWLS_T2 | 0.28496924 | 0.32378531 | 0.09305358 | 0.01544500 | 0.11389613 | 0.11224621 | 0.15394183 | -0.14214685 | -0.11939004 |

| **Pearson Correlation Matrix (Contd.)** | | | | | | | |
| --- | --- | --- | --- | --- | --- | --- | --- |
|  | **INKON_ALL2** | **HT1** | **HT2** | **SQR_BDI_T1** | **SQR_BDI_T2** | **SWLS_T1** | **SWLS_T2** |
| CPT1 |  |  |  |  |  |  |  |
| CAT1 |  |  |  |  |  |  |  |
| CIT1 |  |  |  |  |  |  |  |
| CPT2 |  |  |  |  |  |  |  |
| CAT2 |  |  |  |  |  |  |  |
| CIT2 |  |  |  |  |  |  |  |
| PGQ_IND_C1 |  |  |  |  |  |  |  |
| PGQ_ACH_C1 |  |  |  |  |  |  |  |
| PGQ_AFF_C1 |  |  |  |  |  |  |  |
| PGQ_IND_C2 |  |  |  |  |  |  |  |
| PGQ_ACH_C2 |  |  |  |  |  |  |  |
| PGQ_AFF_C2 |  |  |  |  |  |  |  |
| IEMIND1 |  |  |  |  |  |  |  |
| IEMACH1 |  |  |  |  |  |  |  |
| IEMAFF1 |  |  |  |  |  |  |  |
| KONGRUENZ1 |  |  |  |  |  |  |  |
| IEMIND2 |  |  |  |  |  |  |  |
| IEMACH2 |  |  |  |  |  |  |  |
| IEMAFF2 |  |  |  |  |  |  |  |
| KONGRUENZ2 |  |  |  |  |  |  |  |
| INKON_IND1 |  |  |  |  |  |  |  |
| INKON_ACH1 |  |  |  |  |  |  |  |
| INKON_AFF1 |  |  |  |  |  |  |  |
| INKON_ALL1 |  |  |  |  |  |  |  |
| INKON_IND2 |  |  |  |  |  |  |  |
| INKON_ACH2 |  |  |  |  |  |  |  |
| INKON_AFF2 |  |  |  |  |  |  |  |
| INKON_ALL2 | 1.00000000 |  |  |  |  |  |  |
| HT1 | -0.12721031 | 1.00000000 |  |  |  |  |  |
| HT2 | -0.14557961 | 0.29298210 | 1.00000000 |  |  |  |  |
| SQR_BDI_T1 | 0.14144182 | -0.63399074 | -0.26193144 | 1.00000000 |  |  |  |
| SQR_BDI_T2 | 0.23968299 | -0.54023730 | -0.49270617 | 0.69515772 | 1.00000000 |  |  |
| SWLS_T1 | -0.21388406 | 0.46463715 | 0.28288002 | -0.46591543 | -0.44031660 | 1.00000000 |  |
| SWLS_T2 | -0.06533158 | 0.38087352 | 0.35269602 | -0.39510196 | -0.46888556 | 0.85382827 | 1.00000000 |

| **Pairwise Frequency Table** | | | | | | | | | | | | | | | |
| --- | --- | --- | --- | --- | --- | --- | --- | --- | --- | --- | --- | --- | --- | --- | --- |
|  | **CPT1** | **CAT1** | **CIT1** | **CPT2** | **CAT2** | **CIT2** | **PGQ_IND_C1** | **PGQ_ACH_C1** | **PGQ_AFF_C1** | **PGQ_IND_C2** | **PGQ_ACH_C2** | **PGQ_AFF_C2** | **IEMIND1** | **IEMACH1** | **IEMAFF1** |
| CPT1 | 74 |  |  |  |  |  |  |  |  |  |  |  |  |  |  |
| CAT1 | 74 | 74 |  |  |  |  |  |  |  |  |  |  |  |  |  |
| CIT1 | 74 | 74 | 74 |  |  |  |  |  |  |  |  |  |  |  |  |
| CPT2 | 74 | 74 | 74 | 74 |  |  |  |  |  |  |  |  |  |  |  |
| CAT2 | 74 | 74 | 74 | 74 | 74 |  |  |  |  |  |  |  |  |  |  |
| CIT2 | 74 | 74 | 74 | 74 | 74 | 74 |  |  |  |  |  |  |  |  |  |
| PGQ_IND_C1 | 74 | 74 | 74 | 74 | 74 | 74 | 74 |  |  |  |  |  |  |  |  |
| PGQ_ACH_C1 | 74 | 74 | 74 | 74 | 74 | 74 | 74 | 74 |  |  |  |  |  |  |  |
| PGQ_AFF_C1 | 74 | 74 | 74 | 74 | 74 | 74 | 74 | 74 | 74 |  |  |  |  |  |  |
| PGQ_IND_C2 | 74 | 74 | 74 | 74 | 74 | 74 | 74 | 74 | 74 | 74 |  |  |  |  |  |
| PGQ_ACH_C2 | 74 | 74 | 74 | 74 | 74 | 74 | 74 | 74 | 74 | 74 | 74 |  |  |  |  |
| PGQ_AFF_C2 | 74 | 74 | 74 | 74 | 74 | 74 | 74 | 74 | 74 | 74 | 74 | 74 |  |  |  |
| IEMIND1 | 74 | 74 | 74 | 74 | 74 | 74 | 74 | 74 | 74 | 74 | 74 | 74 | 74 |  |  |
| IEMACH1 | 74 | 74 | 74 | 74 | 74 | 74 | 74 | 74 | 74 | 74 | 74 | 74 | 74 | 74 |  |
| IEMAFF1 | 74 | 74 | 74 | 74 | 74 | 74 | 74 | 74 | 74 | 74 | 74 | 74 | 74 | 74 | 74 |
| KONGRUENZ1 | 74 | 74 | 74 | 74 | 74 | 74 | 74 | 74 | 74 | 74 | 74 | 74 | 74 | 74 | 74 |
| IEMIND2 | 74 | 74 | 74 | 74 | 74 | 74 | 74 | 74 | 74 | 74 | 74 | 74 | 74 | 74 | 74 |
| IEMACH2 | 74 | 74 | 74 | 74 | 74 | 74 | 74 | 74 | 74 | 74 | 74 | 74 | 74 | 74 | 74 |
| IEMAFF2 | 74 | 74 | 74 | 74 | 74 | 74 | 74 | 74 | 74 | 74 | 74 | 74 | 74 | 74 | 74 |
| KONGRUENZ2 | 74 | 74 | 74 | 74 | 74 | 74 | 74 | 74 | 74 | 74 | 74 | 74 | 74 | 74 | 74 |
| INKON_IND1 | 74 | 74 | 74 | 74 | 74 | 74 | 74 | 74 | 74 | 74 | 74 | 74 | 74 | 74 | 74 |
| INKON_ACH1 | 74 | 74 | 74 | 74 | 74 | 74 | 74 | 74 | 74 | 74 | 74 | 74 | 74 | 74 | 74 |
| INKON_AFF1 | 74 | 74 | 74 | 74 | 74 | 74 | 74 | 74 | 74 | 74 | 74 | 74 | 74 | 74 | 74 |
| INKON_ALL1 | 74 | 74 | 74 | 74 | 74 | 74 | 74 | 74 | 74 | 74 | 74 | 74 | 74 | 74 | 74 |
| INKON_IND2 | 74 | 74 | 74 | 74 | 74 | 74 | 74 | 74 | 74 | 74 | 74 | 74 | 74 | 74 | 74 |
| INKON_ACH2 | 74 | 74 | 74 | 74 | 74 | 74 | 74 | 74 | 74 | 74 | 74 | 74 | 74 | 74 | 74 |
| INKON_AFF2 | 74 | 74 | 74 | 74 | 74 | 74 | 74 | 74 | 74 | 74 | 74 | 74 | 74 | 74 | 74 |
| INKON_ALL2 | 74 | 74 | 74 | 74 | 74 | 74 | 74 | 74 | 74 | 74 | 74 | 74 | 74 | 74 | 74 |
| HT1 | 74 | 74 | 74 | 74 | 74 | 74 | 74 | 74 | 74 | 74 | 74 | 74 | 74 | 74 | 74 |
| HT2 | 73 | 73 | 73 | 73 | 73 | 73 | 73 | 73 | 73 | 73 | 73 | 73 | 73 | 73 | 73 |
| SQR_BDI_T1 | 74 | 74 | 74 | 74 | 74 | 74 | 74 | 74 | 74 | 74 | 74 | 74 | 74 | 74 | 74 |
| SQR_BDI_T2 | 73 | 73 | 73 | 73 | 73 | 73 | 73 | 73 | 73 | 73 | 73 | 73 | 73 | 73 | 73 |
| SWLS_T1 | 74 | 74 | 74 | 74 | 74 | 74 | 74 | 74 | 74 | 74 | 74 | 74 | 74 | 74 | 74 |
| SWLS_T2 | 74 | 74 | 74 | 74 | 74 | 74 | 74 | 74 | 74 | 74 | 74 | 74 | 74 | 74 | 74 |

| **Pairwise Frequency Table (Contd.)** | | | | | | | | | | | |
| --- | --- | --- | --- | --- | --- | --- | --- | --- | --- | --- | --- |
|  | **KONGRUENZ1** | **IEMIND2** | **IEMACH2** | **IEMAFF2** | **KONGRUENZ2** | **INKON_IND1** | **INKON_ACH1** | **INKON_AFF1** | **INKON_ALL1** | **INKON_IND2** | **INKON_ACH2** |
| CPT1 |  |  |  |  |  |  |  |  |  |  |  |
| CAT1 |  |  |  |  |  |  |  |  |  |  |  |
| CIT1 |  |  |  |  |  |  |  |  |  |  |  |
| CPT2 |  |  |  |  |  |  |  |  |  |  |  |
| CAT2 |  |  |  |  |  |  |  |  |  |  |  |
| CIT2 |  |  |  |  |  |  |  |  |  |  |  |
| PGQ_IND_C1 |  |  |  |  |  |  |  |  |  |  |  |
| PGQ_ACH_C1 |  |  |  |  |  |  |  |  |  |  |  |
| PGQ_AFF_C1 |  |  |  |  |  |  |  |  |  |  |  |
| PGQ_IND_C2 |  |  |  |  |  |  |  |  |  |  |  |
| PGQ_ACH_C2 |  |  |  |  |  |  |  |  |  |  |  |
| PGQ_AFF_C2 |  |  |  |  |  |  |  |  |  |  |  |
| IEMIND1 |  |  |  |  |  |  |  |  |  |  |  |
| IEMACH1 |  |  |  |  |  |  |  |  |  |  |  |
| IEMAFF1 |  |  |  |  |  |  |  |  |  |  |  |
| KONGRUENZ1 | 74 |  |  |  |  |  |  |  |  |  |  |
| IEMIND2 | 74 | 74 |  |  |  |  |  |  |  |  |  |
| IEMACH2 | 74 | 74 | 74 |  |  |  |  |  |  |  |  |
| IEMAFF2 | 74 | 74 | 74 | 74 |  |  |  |  |  |  |  |
| KONGRUENZ2 | 74 | 74 | 74 | 74 | 74 |  |  |  |  |  |  |
| INKON_IND1 | 74 | 74 | 74 | 74 | 74 | 74 |  |  |  |  |  |
| INKON_ACH1 | 74 | 74 | 74 | 74 | 74 | 74 | 74 |  |  |  |  |
| INKON_AFF1 | 74 | 74 | 74 | 74 | 74 | 74 | 74 | 74 |  |  |  |
| INKON_ALL1 | 74 | 74 | 74 | 74 | 74 | 74 | 74 | 74 | 74 |  |  |
| INKON_IND2 | 74 | 74 | 74 | 74 | 74 | 74 | 74 | 74 | 74 | 74 |  |
| INKON_ACH2 | 74 | 74 | 74 | 74 | 74 | 74 | 74 | 74 | 74 | 74 | 74 |
| INKON_AFF2 | 74 | 74 | 74 | 74 | 74 | 74 | 74 | 74 | 74 | 74 | 74 |
| INKON_ALL2 | 74 | 74 | 74 | 74 | 74 | 74 | 74 | 74 | 74 | 74 | 74 |
| HT1 | 74 | 74 | 74 | 74 | 74 | 74 | 74 | 74 | 74 | 74 | 74 |
| HT2 | 73 | 73 | 73 | 73 | 73 | 73 | 73 | 73 | 73 | 73 | 73 |
| SQR_BDI_T1 | 74 | 74 | 74 | 74 | 74 | 74 | 74 | 74 | 74 | 74 | 74 |
| SQR_BDI_T2 | 73 | 73 | 73 | 73 | 73 | 73 | 73 | 73 | 73 | 73 | 73 |
| SWLS_T1 | 74 | 74 | 74 | 74 | 74 | 74 | 74 | 74 | 74 | 74 | 74 |
| SWLS_T2 | 74 | 74 | 74 | 74 | 74 | 74 | 74 | 74 | 74 | 74 | 74 |

| **Pairwise Frequency Table (Contd.)** | | | | | | | | |
| --- | --- | --- | --- | --- | --- | --- | --- | --- |
|  | **INKON_AFF2** | **INKON_ALL2** | **HT1** | **HT2** | **SQR_BDI_T1** | **SQR_BDI_T2** | **SWLS_T1** | **SWLS_T2** |
| CPT1 |  |  |  |  |  |  |  |  |
| CAT1 |  |  |  |  |  |  |  |  |
| CIT1 |  |  |  |  |  |  |  |  |
| CPT2 |  |  |  |  |  |  |  |  |
| CAT2 |  |  |  |  |  |  |  |  |
| CIT2 |  |  |  |  |  |  |  |  |
| PGQ_IND_C1 |  |  |  |  |  |  |  |  |
| PGQ_ACH_C1 |  |  |  |  |  |  |  |  |
| PGQ_AFF_C1 |  |  |  |  |  |  |  |  |
| PGQ_IND_C2 |  |  |  |  |  |  |  |  |
| PGQ_ACH_C2 |  |  |  |  |  |  |  |  |
| PGQ_AFF_C2 |  |  |  |  |  |  |  |  |
| IEMIND1 |  |  |  |  |  |  |  |  |
| IEMACH1 |  |  |  |  |  |  |  |  |
| IEMAFF1 |  |  |  |  |  |  |  |  |
| KONGRUENZ1 |  |  |  |  |  |  |  |  |
| IEMIND2 |  |  |  |  |  |  |  |  |
| IEMACH2 |  |  |  |  |  |  |  |  |
| IEMAFF2 |  |  |  |  |  |  |  |  |
| KONGRUENZ2 |  |  |  |  |  |  |  |  |
| INKON_IND1 |  |  |  |  |  |  |  |  |
| INKON_ACH1 |  |  |  |  |  |  |  |  |
| INKON_AFF1 |  |  |  |  |  |  |  |  |
| INKON_ALL1 |  |  |  |  |  |  |  |  |
| INKON_IND2 |  |  |  |  |  |  |  |  |
| INKON_ACH2 |  |  |  |  |  |  |  |  |
| INKON_AFF2 | 74 |  |  |  |  |  |  |  |
| INKON_ALL2 | 74 | 74 |  |  |  |  |  |  |
| HT1 | 74 | 74 | 74 |  |  |  |  |  |
| HT2 | 73 | 73 | 73 | 73 |  |  |  |  |
| SQR_BDI_T1 | 74 | 74 | 74 | 73 | 74 |  |  |  |
| SQR_BDI_T2 | 73 | 73 | 73 | 72 | 73 | 73 |  |  |
| SWLS_T1 | 74 | 74 | 74 | 73 | 74 | 73 | 74 |  |
| SWLS_T2 | 74 | 74 | 74 | 73 | 74 | 73 | 74 | 74 |

WARNING Correlation matrix is not positive definite.

Individual significance tests are suspect.

| **Matrix of Probabilities** | | | | | | | | | | |
| --- | --- | --- | --- | --- | --- | --- | --- | --- | --- | --- |
|  | **CPT1** | **CAT1** | **CIT1** | **CPT2** | **CAT2** | **CIT2** | **PGQ_IND_C1** | **PGQ_ACH_C1** | **PGQ_AFF_C1** | **PGQ_IND_C2** |
| CPT1 | 0.00000000 |  |  |  |  |  |  |  |  |  |
| CAT1 | 0.13314103 | 0.00000000 |  |  |  |  |  |  |  |  |
| CIT1 | 0.47099868 | 0.00252528 | 0.00000000 |  |  |  |  |  |  |  |
| CPT2 | 0.02070209 | 0.26889963 | 0.46988197 | 0.00000000 |  |  |  |  |  |  |
| CAT2 | 0.77525981 | 0.01603446 | 0.00037691 | 0.41146318 | 0.00000000 |  |  |  |  |  |
| CIT2 | 0.78067139 | 0.00488736 | 0.00082319 | 0.02722577 | 0.03259805 | 0.00000000 |  |  |  |  |
| PGQ_IND_C1 | 0.78773961 | 0.42096493 | 0.19133949 | 0.15559382 | 0.25232461 | 0.38625951 | 0.00000000 |  |  |  |
| PGQ_ACH_C1 | 0.78314365 | 0.14874432 | 0.68497170 | 0.75033429 | 0.87822068 | 0.15225158 | 0.05502832 | 0.00000000 |  |  |
| PGQ_AFF_C1 | 0.01717010 | 0.21524118 | 0.91533556 | 0.63769685 | 0.25780486 | 0.93620230 | 0.10047121 | 0.00466314 | 0.00000000 |  |
| PGQ_IND_C2 | 0.38556172 | 0.23284744 | 0.38683651 | 0.95595353 | 0.81317665 | 0.73027345 | 0.00000005 | 0.00542057 | 0.01440456 | 0.00000000 |
| PGQ_ACH_C2 | 0.38204354 | 0.05364507 | 0.57676445 | 0.58021917 | 0.85983846 | 0.48055347 | 0.00223304 | 0.00000000 | 0.00111797 | 0.00002299 |
| PGQ_AFF_C2 | 0.54316382 | 0.49012268 | 0.86594074 | 0.97566699 | 0.88293979 | 0.55780672 | 0.00000149 | 0.00800504 | 0.00055493 | 0.00061675 |
| IEMIND1 | 0.00000173 | 0.21932977 | 0.16252600 | 0.83241355 | 0.47163060 | 0.63443029 | 0.01429350 | 0.64861495 | 0.13676229 | 0.08443621 |
| IEMACH1 | 0.81794831 | 0.00000000 | 0.01577081 | 0.71268064 | 0.25211933 | 0.26914215 | 0.18597561 | 0.00000249 | 0.07420070 | 0.01530372 |
| IEMAFF1 | 0.94428391 | 0.00216646 | 0.00000005 | 0.77436563 | 0.90249677 | 0.03238239 | 0.05448481 | 0.03360841 | 0.00022035 | 0.01415076 |
| KONGRUENZ1 | 0.02272948 | 0.00000014 | 0.00000459 | 0.66659929 | 0.31724460 | 0.06696910 | 0.00457750 | 0.00027037 | 0.00053399 | 0.00084697 |
| IEMIND2 | 0.16077046 | 0.04625283 | 0.57236590 | 0.00000009 | 0.83139056 | 0.49959822 | 0.44481510 | 0.58488379 | 0.06850465 | 0.00008356 |
| IEMACH2 | 0.40664426 | 0.06900714 | 0.03902846 | 0.84806053 | 0.00000046 | 0.00168819 | 0.45182056 | 0.00627365 | 0.65348331 | 0.03314358 |
| IEMAFF2 | 0.30760989 | 0.01271853 | 0.01808963 | 0.05447402 | 0.24295485 | 0.00000003 | 0.00096495 | 0.02717520 | 0.55814018 | 0.09928612 |
| KONGRUENZ2 | 0.53662459 | 0.00125783 | 0.05100121 | 0.17123491 | 0.00137512 | 0.00008636 | 0.01711266 | 0.00503244 | 0.15505265 | 0.00007923 |
| INKON_IND1 | 0.88745244 | 0.62860786 | 0.20079108 | 0.98657531 | 0.91769989 | 0.32934642 | 0.05366143 | 0.71370301 | 0.78845344 | 0.96353919 |
| INKON_ACH1 | 0.61053127 | 0.58193052 | 0.48204491 | 0.60341219 | 0.33514544 | 0.16003792 | 0.18633498 | 0.50123165 | 0.79847011 | 0.88016115 |
| INKON_AFF1 | 0.48784368 | 0.68759107 | 0.40177134 | 0.18944462 | 0.11820175 | 0.36548196 | 0.44377021 | 0.94972597 | 0.83315026 | 0.30947146 |
| INKON_ALL1 | 0.57552003 | 0.79198742 | 0.82199267 | 0.32731195 | 0.17227693 | 0.11008580 | 0.85253657 | 0.85501356 | 0.90527224 | 0.53806059 |
| INKON_IND2 | 0.99613600 | 0.25187700 | 0.78136591 | 0.50661872 | 0.68203062 | 0.81905239 | 0.90713843 | 0.70983901 | 0.18832336 | 0.22481199 |
| INKON_ACH2 | 0.56959993 | 0.38672330 | 0.76302063 | 0.50019665 | 0.68882443 | 0.05561387 | 0.90919566 | 0.19230371 | 0.53454375 | 0.61171453 |
| INKON_AFF2 | 0.62660902 | 0.31808718 | 0.36615835 | 0.35791117 | 0.96835687 | 0.56113617 | 0.12250993 | 0.33665167 | 0.90487132 | 0.14977569 |
| INKON_ALL2 | 0.53116343 | 0.07673857 | 0.39320164 | 0.77531723 | 0.97474083 | 0.10142814 | 0.38792820 | 0.26052779 | 0.28129656 | 0.06646419 |
| HT1 | 0.86491384 | 0.11486408 | 0.40603590 | 0.04942429 | 0.62692092 | 0.57688490 | 0.43777440 | 0.12259596 | 0.09099635 | 0.04030245 |
| HT2 | 0.80453490 | 0.13536104 | 0.14630010 | 0.90671665 | 0.10100441 | 0.03897718 | 0.04603707 | 0.17257478 | 0.00005156 | 0.06538210 |
| SQR_BDI_T1 | 0.10448015 | 0.00243834 | 0.49252114 | 0.93598356 | 0.34036405 | 0.25434811 | 0.12524977 | 0.02618608 | 0.18473117 | 0.01413086 |
| SQR_BDI_T2 | 0.27603512 | 0.00441986 | 0.25735413 | 0.31989103 | 0.18732401 | 0.16225508 | 0.02463382 | 0.05508439 | 0.03730475 | 0.00452791 |
| SWLS_T1 | 0.37796347 | 0.14438976 | 0.99589171 | 0.04088520 | 0.92459384 | 0.44728323 | 0.06518711 | 0.00173343 | 0.06990338 | 0.00776866 |
| SWLS_T2 | 0.39794444 | 0.06280137 | 0.54323470 | 0.07108620 | 0.91968571 | 0.16330450 | 0.01037878 | 0.04409442 | 0.12262230 | 0.00392679 |

| **Matrix of Probabilities (Contd.)** | | | | | | | | | | |
| --- | --- | --- | --- | --- | --- | --- | --- | --- | --- | --- |
|  | **PGQ_ACH_C2** | **PGQ_AFF_C2** | **IEMIND1** | **IEMACH1** | **IEMAFF1** | **KONGRUENZ1** | **IEMIND2** | **IEMACH2** | **IEMAFF2** | **KONGRUENZ2** |
| CPT1 |  |  |  |  |  |  |  |  |  |  |
| CAT1 |  |  |  |  |  |  |  |  |  |  |
| CIT1 |  |  |  |  |  |  |  |  |  |  |
| CPT2 |  |  |  |  |  |  |  |  |  |  |
| CAT2 |  |  |  |  |  |  |  |  |  |  |
| CIT2 |  |  |  |  |  |  |  |  |  |  |
| PGQ_IND_C1 |  |  |  |  |  |  |  |  |  |  |
| PGQ_ACH_C1 |  |  |  |  |  |  |  |  |  |  |
| PGQ_AFF_C1 |  |  |  |  |  |  |  |  |  |  |
| PGQ_IND_C2 |  |  |  |  |  |  |  |  |  |  |
| PGQ_ACH_C2 | 0.00000000 |  |  |  |  |  |  |  |  |  |
| PGQ_AFF_C2 | 0.00051600 | 0.00000000 |  |  |  |  |  |  |  |  |
| IEMIND1 | 0.37494130 | 0.28641185 | 0.00000000 |  |  |  |  |  |  |  |
| IEMACH1 | 0.00027605 | 0.64921964 | 0.73392192 | 0.00000000 |  |  |  |  |  |  |
| IEMAFF1 | 0.00734025 | 0.00443554 | 0.52959118 | 0.00015171 | 0.00000000 |  |  |  |  |  |
| KONGRUENZ1 | 0.00026998 | 0.03462316 | 0.00000058 | 0.00000000 | 0.00000000 | 0.00000000 |  |  |  |  |
| IEMIND2 | 0.08787847 | 0.24410208 | 0.32168725 | 0.39825067 | 0.52959118 | 0.22125140 | 0.00000000 |  |  |  |
| IEMACH2 | 0.00374612 | 0.22426843 | 0.31333792 | 0.01055218 | 0.08034509 | 0.00746188 | 0.12783618 | 0.00000000 |  |  |
| IEMAFF2 | 0.01944551 | 0.00001312 | 0.32168725 | 0.06133440 | 0.02204198 | 0.01008833 | 0.64948337 | 0.04077874 | 0.00000000 |  |
| KONGRUENZ2 | 0.00033134 | 0.00081913 | 0.13082339 | 0.00696344 | 0.01791451 | 0.00087748 | 0.00000001 | 0.00000000 | 0.00000000 | 0.00000000 |
| INKON_IND1 | 0.66177406 | 0.48629275 | 0.00013113 | 0.91849413 | 0.17783127 | 0.01606161 | 0.78375387 | 0.68544843 | 0.53524608 | 0.70292695 |
| INKON_ACH1 | 0.95752149 | 0.09163175 | 0.44844452 | 0.01211686 | 0.45834211 | 0.20725659 | 0.94123053 | 0.68099587 | 0.08778827 | 0.55863192 |
| INKON_AFF1 | 0.84976363 | 0.23721899 | 0.15136240 | 0.54965859 | 0.01174016 | 0.42293646 | 0.94478642 | 0.98981480 | 0.93001668 | 0.93287469 |
| INKON_ALL1 | 0.78247570 | 0.84681702 | 0.63401431 | 0.15124874 | 0.01819539 | 0.03469014 | 0.84525545 | 0.98649511 | 0.31088028 | 0.69379651 |
| INKON_IND2 | 0.22614756 | 0.28597829 | 0.20623396 | 0.35081527 | 0.42712313 | 0.13795267 | 0.00137605 | 0.40266265 | 0.46359753 | 0.10665010 |
| INKON_ACH2 | 0.15061897 | 0.01222183 | 0.88510559 | 0.36945687 | 0.02119144 | 0.10223370 | 0.18665535 | 0.00029948 | 0.05855926 | 0.00043405 |
| INKON_AFF2 | 0.17763226 | 0.28527928 | 0.64624566 | 0.08157828 | 0.15253165 | 0.06944565 | 0.77442921 | 0.93735907 | 0.00004380 | 0.06779373 |
| INKON_ALL2 | 0.01758377 | 0.13267081 | 0.28064357 | 0.03679690 | 0.00655489 | 0.00323998 | 0.01273327 | 0.00637572 | 0.00287520 | 0.00001903 |
| HT1 | 0.05156544 | 0.57337278 | 0.91625962 | 0.07921539 | 0.53682539 | 0.21116899 | 0.16598662 | 0.03493151 | 0.85702353 | 0.09119069 |
| HT2 | 0.56233735 | 0.00266450 | 0.42216423 | 0.23133603 | 0.00512304 | 0.01896679 | 0.21056624 | 0.00536983 | 0.08513378 | 0.00334647 |
| SQR_BDI_T1 | 0.01333881 | 0.61212362 | 0.41535089 | 0.00418631 | 0.40120037 | 0.02293309 | 0.94078298 | 0.12021365 | 0.32995994 | 0.18449790 |
| SQR_BDI_T2 | 0.00324839 | 0.06003298 | 0.87457102 | 0.04971906 | 0.01494082 | 0.02458794 | 0.09293389 | 0.03774620 | 0.16960797 | 0.00747136 |
| SWLS_T1 | 0.00480675 | 0.01740052 | 0.31814911 | 0.00136759 | 0.11035223 | 0.00345563 | 0.04185049 | 0.06548309 | 0.06655297 | 0.00350707 |
| SWLS_T2 | 0.02344125 | 0.00177706 | 0.99390613 | 0.00947965 | 0.03988169 | 0.01992462 | 0.09446023 | 0.15826956 | 0.01385807 | 0.00489106 |

| **Matrix of Probabilities (Contd.)** | | | | | | | | | | |
| --- | --- | --- | --- | --- | --- | --- | --- | --- | --- | --- |
|  | **INKON_IND1** | **INKON_ACH1** | **INKON_AFF1** | **INKON_ALL1** | **INKON_IND2** | **INKON_ACH2** | **INKON_AFF2** | **INKON_ALL2** | **HT1** | **HT2** |
| CPT1 |  |  |  |  |  |  |  |  |  |  |
| CAT1 |  |  |  |  |  |  |  |  |  |  |
| CIT1 |  |  |  |  |  |  |  |  |  |  |
| CPT2 |  |  |  |  |  |  |  |  |  |  |
| CAT2 |  |  |  |  |  |  |  |  |  |  |
| CIT2 |  |  |  |  |  |  |  |  |  |  |
| PGQ_IND_C1 |  |  |  |  |  |  |  |  |  |  |
| PGQ_ACH_C1 |  |  |  |  |  |  |  |  |  |  |
| PGQ_AFF_C1 |  |  |  |  |  |  |  |  |  |  |
| PGQ_IND_C2 |  |  |  |  |  |  |  |  |  |  |
| PGQ_ACH_C2 |  |  |  |  |  |  |  |  |  |  |
| PGQ_AFF_C2 |  |  |  |  |  |  |  |  |  |  |
| IEMIND1 |  |  |  |  |  |  |  |  |  |  |
| IEMACH1 |  |  |  |  |  |  |  |  |  |  |
| IEMAFF1 |  |  |  |  |  |  |  |  |  |  |
| KONGRUENZ1 |  |  |  |  |  |  |  |  |  |  |
| IEMIND2 |  |  |  |  |  |  |  |  |  |  |
| IEMACH2 |  |  |  |  |  |  |  |  |  |  |
| IEMAFF2 |  |  |  |  |  |  |  |  |  |  |
| KONGRUENZ2 |  |  |  |  |  |  |  |  |  |  |
| INKON_IND1 | 0.00000000 |  |  |  |  |  |  |  |  |  |
| INKON_ACH1 | 0.98862575 | 0.00000000 |  |  |  |  |  |  |  |  |
| INKON_AFF1 | 0.42973162 | 0.00008660 | 0.00000000 |  |  |  |  |  |  |  |
| INKON_ALL1 | 0.00000612 | 0.00000000 | 0.00000000 | 0.00000000 |  |  |  |  |  |  |
| INKON_IND2 | 0.02833015 | 0.85007684 | 0.65439529 | 0.42314369 | 0.00000000 |  |  |  |  |  |
| INKON_ACH2 | 0.62312329 | 0.37944454 | 0.56577741 | 0.34302072 | 0.72503890 | 0.00000000 |  |  |  |  |
| INKON_AFF2 | 0.59077852 | 0.40210083 | 0.10534746 | 0.29476845 | 0.80081462 | 0.62250020 | 0.00000000 |  |  |  |
| INKON_ALL2 | 0.20839164 | 0.26134005 | 0.31864065 | 0.09971825 | 0.00000004 | 0.00000001 | 0.00000396 | 0.00000000 |  |  |
| HT1 | 0.23852526 | 0.35828127 | 0.41283243 | 0.57847762 | 0.93076012 | 0.25683257 | 0.44178958 | 0.28011116 | 0.00000000 |  |
| HT2 | 0.21365742 | 0.11748994 | 0.27027481 | 0.05638105 | 0.36646355 | 0.07856869 | 0.51453901 | 0.21910030 | 0.01188643 | 0.00000000 |
| SQR_BDI_T1 | 0.09012701 | 0.49811986 | 0.13068398 | 0.20074103 | 0.37062432 | 0.25119584 | 0.06849668 | 0.22933698 | 0.00000000 | 0.02518483 |
| SQR_BDI_T2 | 0.48398971 | 0.71271500 | 0.85072929 | 0.80634155 | 0.44394700 | 0.08161799 | 0.35861080 | 0.04111373 | 0.00000081 | 0.00001100 |
| SWLS_T1 | 0.85472391 | 0.73392201 | 0.38934962 | 0.80995103 | 0.86760466 | 0.07630810 | 0.13824379 | 0.06727781 | 0.00003041 | 0.01530984 |
| SWLS_T2 | 0.43036782 | 0.89608485 | 0.33392404 | 0.34101932 | 0.19035349 | 0.22700041 | 0.31097567 | 0.58024496 | 0.00081470 | 0.00221028 |

| **Matrix of Probabilities (Contd.)** | | | | |
| --- | --- | --- | --- | --- |
|  | **SQR_BDI_T1** | **SQR_BDI_T2** | **SWLS_T1** | **SWLS_T2** |
| CPT1 |  |  |  |  |
| CAT1 |  |  |  |  |
| CIT1 |  |  |  |  |
| CPT2 |  |  |  |  |
| CAT2 |  |  |  |  |
| CIT2 |  |  |  |  |
| PGQ_IND_C1 |  |  |  |  |
| PGQ_ACH_C1 |  |  |  |  |
| PGQ_AFF_C1 |  |  |  |  |
| PGQ_IND_C2 |  |  |  |  |
| PGQ_ACH_C2 |  |  |  |  |
| PGQ_AFF_C2 |  |  |  |  |
| IEMIND1 |  |  |  |  |
| IEMACH1 |  |  |  |  |
| IEMAFF1 |  |  |  |  |
| KONGRUENZ1 |  |  |  |  |
| IEMIND2 |  |  |  |  |
| IEMACH2 |  |  |  |  |
| IEMAFF2 |  |  |  |  |
| KONGRUENZ2 |  |  |  |  |
| INKON_IND1 |  |  |  |  |
| INKON_ACH1 |  |  |  |  |
| INKON_AFF1 |  |  |  |  |
| INKON_ALL1 |  |  |  |  |
| INKON_IND2 |  |  |  |  |
| INKON_ACH2 |  |  |  |  |
| INKON_AFF2 |  |  |  |  |
| INKON_ALL2 |  |  |  |  |
| HT1 |  |  |  |  |
| HT2 |  |  |  |  |
| SQR_BDI_T1 | 0.00000000 |  |  |  |
| SQR_BDI_T2 | 0.00000000 | 0.00000000 |  |  |
| SWLS_T1 | 0.00002872 | 0.00009688 | 0.00000000 |  |
| SWLS_T2 | 0.00049430 | 0.00002864 | 0.00000000 | 0.00000000 |

> *format 12, 2*

> *cstats cpt1 cat1 cit1 cpt2 cat2 cit2 PGQ_IND_C1 PGQ_ach_C1 PGQ_AFF_C1 PGQ_IND_C2 PGQ_ach_C2 PGQ_AFF_C2 iemind1 iemach1 iemaff1 kongruenz1 iemind2 iemach2 iemaff2
kongruenz2 Inkon_ind1 Inkon_ach1 Inkon_aff1 inkon_all1 Inkon_ind2 Inkon_ach2 Inkon_aff2 inkon_all2 ht1 ht2 sqr_bdi_t1 sqr_bdi_t2 swls_t1 swls_t2 /mean sd n*

[▼Descriptive Statistics](file:///\\Untitled.syo)

|  | **CPT1** | **CAT1** | **CIT1** | **CPT2** | **CAT2** | **CIT2** | **PGQ_IND_C1** | **PGQ_ACH_C1** | **PGQ_AFF_C1** | **PGQ_IND_C2** | **PGQ_ACH_C2** | **PGQ_AFF_C2** | **IEMIND1** |
| --- | --- | --- | --- | --- | --- | --- | --- | --- | --- | --- | --- | --- | --- |
| N of Cases | 74 | 74 | 74 | 74 | 74 | 74 | 74 | 74 | 74 | 74 | 74 | 74 | 74 |
| Arithmetic Mean | 10.02 | 8.29 | 10.87 | 9.26 | 8.89 | 10.43 | 4.07 | 4.21 | 3.93 | 3.97 | 4.22 | 4.03 | 0.31 |
| Standard Deviation | 3.93 | 3.48 | 4.48 | 4.15 | 4.38 | 4.78 | 0.80 | 0.69 | 0.77 | 0.83 | 0.62 | 0.64 | 0.47 |

|  | **IEMACH1** | **IEMAFF1** | **KONGRUENZ1** | **IEMIND2** | **IEMACH2** | **IEMAFF2** | **KONGRUENZ2** | **INKON_IND1** | **INKON_ACH1** | **INKON_AFF1** | **INKON_ALL1** |
| --- | --- | --- | --- | --- | --- | --- | --- | --- | --- | --- | --- |
| N of Cases | 74 | 74 | 74 | 74 | 74 | 74 | 74 | 74 | 74 | 74 | 74 |
| Arithmetic Mean | 0.41 | 0.30 | 1.01 | 0.31 | 0.39 | 0.31 | 1.01 | 0.45 | 0.34 | 0.33 | 0.37 |
| Standard Deviation | 0.49 | 0.46 | 0.96 | 0.47 | 0.49 | 0.47 | 0.94 | 0.44 | 0.45 | 0.55 | 0.33 |

|  | **INKON_IND2** | **INKON_ACH2** | **INKON_AFF2** | **INKON_ALL2** | **HT1** | **HT2** | **SQR_BDI_T1** | **SQR_BDI_T2** | **SWLS_T1** | **SWLS_T2** |
| --- | --- | --- | --- | --- | --- | --- | --- | --- | --- | --- |
| N of Cases | 74 | 74 | 74 | 74 | 74 | 73 | 74 | 73 | 74 | 74 |
| Arithmetic Mean | 0.35 | 0.27 | 0.39 | 0.34 | 3.54 | 3.65 | 2.88 | 2.69 | 4.76 | 4.86 |
| Standard Deviation | 0.51 | 0.54 | 0.49 | 0.29 | 0.80 | 0.70 | 1.09 | 0.98 | 1.03 | 1.06 |

> *!! Generating Figure 2*

> *format 12, 6*

> *varlabel iemaff1 /"T1"*

> *varlabel iemaff2 /"T2"*

> *varlabel inkon_aff1 /"T1"*

> *varlabel inkon_aff2 /"T2"*

> *label condition / 1 = "Control", 2 = "Feedback", 3 = "Feedback + CET"*

> *begin*

> *thick 1.5*

> *write "Power" / LOC={0.3in, 3.5in}*

> *write "Achievement" / LOC={0.3in, -0.7in}*

> *write "Affiliation" / LOC={0.3in, -4.9in}*

> *LINE IEMind1 IEMind2 / REPEAT OVERLAY GROUP = {CONDITION} serror scale = left xlabel = " " ylabel = " " ymin = 0 ymax = 1 legend = none COLOR = {Black, Black, Black}
DASH={ 12, 4, 1}*

> *LINE IEMACH1 IEMACH2 / REPEAT OVERLAY GROUP = {CONDITION} serror location = {0in, -4.2in} scale = left xlabel = " " ylabel = "Congruence index (+/- SEM)" ymin = 0 ymax =
1 legend = none COLOR = {Black, Black, Black} DASH={ 12, 4, 1}*

> *LINE IEMAff1 IEMAff2 / REPEAT OVERLAY GROUP = {CONDITION} serror location = {0in, -8.4in} scale = L xlabel = " " ylabel = " " ymin = 0 ymax = 1 legend=none COLOR =
{Black, Black, Black} DASH={ 12, 4, 1}*

> *LINE inkon_ind1 inkon_ind2 / REPEAT OVERLAY GROUP = {CONDITION} serror location = {4.2in, 0in} scale = right xlabel = " " ytick = 5 ylabel = " " ymin = -0.75 ymax = 2
legend = none COLOR = {Black, Black, Black} DASH={ 12, 4, 1}*

> *LINE inkon_ACH1 inkon_ACH2 / REPEAT OVERLAY GROUP = {CONDITION} serror location = {4.2in, -4.2in} scale = right xlabel = " " ytick = 5 ylabel = "Incongruence index (+/-
SEM)" ymin = -0.75 ymax = 2 legend = none COLOR = {Black, Black, Black} DASH={ 12, 4, 1}*

> *LINE inkon_Aff1 inkon_Aff2 / REPEAT OVERLAY GROUP = {CONDITION} serror location = {4.2in, -8.4in} scale = right xlabel = " " ytick = 5 ylabel = " " ymin = -0.75 ymax =
2 ltitle= "Condition" LLABEL = {'Control', 'Feedback', 'Feedback + CET'} COLOR = {Black, Black, Black} DASH={ 12, 4, 1}*

> *LINE inkon_Aff1 inkon_Aff2 / REPEAT OVERLAY GROUP = {CONDITION} serror location = {4.2in, -8.4in} scale = bottom xlabel = " " ytick = 5 ylabel = " " ymin = -0.75 ymax =
2 legend = none COLOR = {Black, Black, Black} DASH={ 12, 4, 1}*

> *end*

[▼Begin/End Plot](file:///\\Untitled.syo)


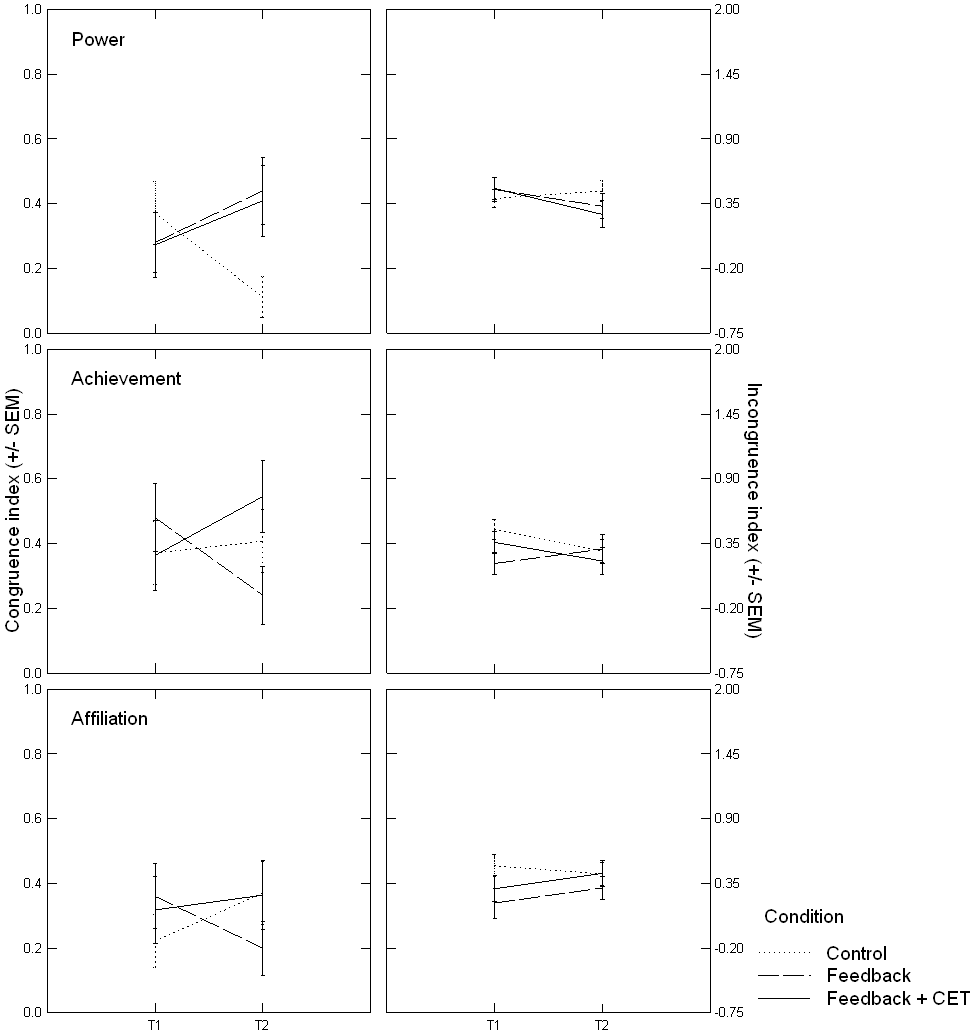


> *!! Generating Figure 3*

> *begin*

> *LINE bdi_t1 bdi_t2/ REPEAT OVERLAY GROUP = {CONDITION} serror scale = left xlabel = " " ylabel = "BDI (+/- SEM)" ypower=0.5 legend = none COLOR = {Black, Black, Black}
DASH={ 12, 4, 1}*

> *LINE ht1 ht2 / REPEAT OVERLAY GROUP = {CONDITION} serror location = {0in, -4.2in} scale = left xlabel = " " ylabel = "HT (+/- SEM)" ymax = 5 legend = none COLOR =
{Black, Black, Black} DASH={ 12, 4, 1}*

> *LINE swls_t1 swls_t2 / REPEAT OVERLAY GROUP = {CONDITION} serror location = {0in, -8.4in} scale = left xlabel = " " ymax = 7 ylabel = "SWLS (+/- SEM)" ltitle=
"Condition" LLABEL = {'Control', 'Feedback', 'Feedback + CET'} COLOR = {Black, Black, Black} DASH={ 12, 4, 1}*

> *end*

[▼Begin/End Plot](file:///\\Untitled.syo)


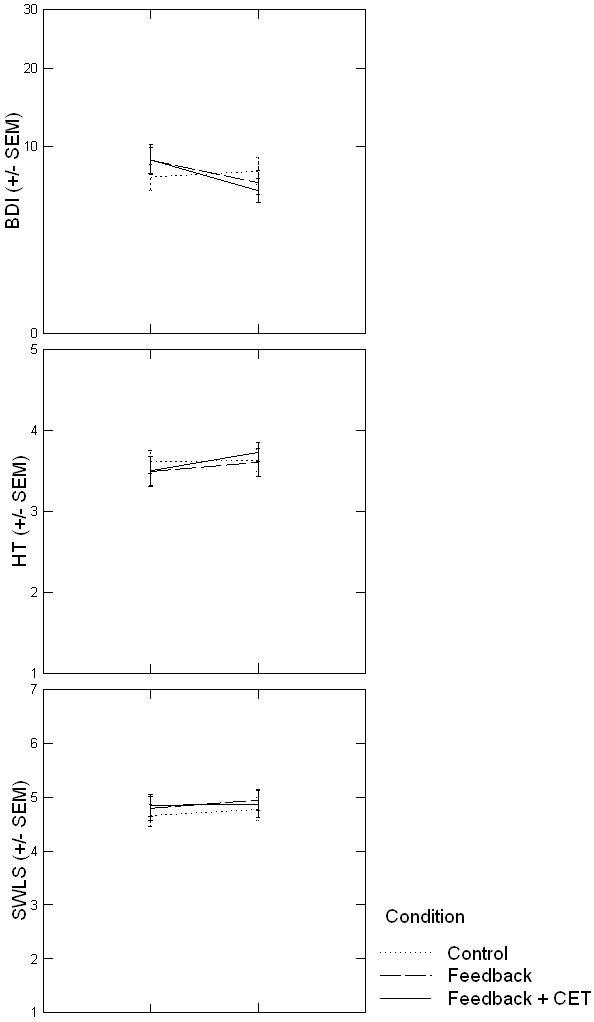


> *!! Generating Figure 4 (residualized T2 scores need to be computed first)*

> *format 12, 6*

> *regress*

> *model kongruenz2 = constant kongruenz1*

> *estimate*

[▼OLS Regression](file:///\\Untitled.syo)

| Dependent Variable | KONGRUENZ2 |
| --- | --- |
| N | 74 |
| Multiple R | 0.378704 |
| Squared Multiple R | 0.143417 |
| Adjusted Squared Multiple R | 0.131520 |
| Standard Error of Estimate | 0.879286 |

| **Regression Coefficients B = (X'X)^-1^X'Y** | | | | | | |
| --- | --- | --- | --- | --- | --- | --- |
| **Effect** | **Coefficient** | **Standard Error** | **Std. Coefficient** | **Tolerance** | **t** | **p-Value** |
| CONSTANT | 0.635465 | 0.149344 | 0.000000 | . | 4.255034 | 0.000062 |
| KONGRUENZ1 | 0.373008 | 0.107433 | 0.378704 | 1.000000 | 3.472015 | 0.000877 |

| **Analysis of Variance** | | | | | |
| --- | --- | --- | --- | --- | --- |
| **Source** | **SS** | **df** | **Mean Squares** | **F-Ratio** | **p-Value** |
| Regression | 9.320156 | 1 | 9.320156 | 12.054885 | 0.000877 |
| Residual | 55.666330 | 72 | 0.773143 |  |  |

| Durbin-Watson D-Statistic | 1.701053 |
| --- | --- |
| First Order Autocorrelation | 0.136711 |

| **Information Criteria** | |
| --- | --- |
| AIC | 194.935872 |
| AIC (Corrected) | 195.278729 |
| Schwarz's BIC | 201.848067 |

> *let reskongruenz = kongruenz2 - (0.635465 + 0.373008*kongruenz1)*

> *regress*

> *model inkon_all2 = constant inkon_all1*

> *estimate*

[▼OLS Regression](file:///\\Untitled.syo)

| Dependent Variable | INKON_ALL2 |
| --- | --- |
| N | 74 |
| Multiple R | 0.192851 |
| Squared Multiple R | 0.037192 |
| Adjusted Squared Multiple R | 0.023819 |
| Standard Error of Estimate | 0.287916 |

| **Regression Coefficients B = (X'X)^-1^X'Y** | | | | | | |
| --- | --- | --- | --- | --- | --- | --- |
| **Effect** | **Coefficient** | **Standard Error** | **Std. Coefficient** | **Tolerance** | **t** | **p-Value** |
| CONSTANT | 0.273501 | 0.050950 | 0.000000 | . | 5.368049 | 0.000001 |
| INKON_ALL1 | 0.171780 | 0.103004 | 0.192851 | 1.000000 | 1.667705 | 0.099718 |

| **Analysis of Variance** | | | | | |
| --- | --- | --- | --- | --- | --- |
| **Source** | **SS** | **df** | **Mean Squares** | **F-Ratio** | **p-Value** |
| Regression | 0.230552 | 1 | 0.230552 | 2.781241 | 0.099718 |
| Residual | 5.968470 | 72 | 0.082895 |  |  |

| Durbin-Watson D-Statistic | 2.140047 |
| --- | --- |
| First Order Autocorrelation | -0.085651 |

| **Information Criteria** | |
| --- | --- |
| AIC | 29.702387 |
| AIC (Corrected) | 30.045244 |
| Schwarz's BIC | 36.614582 |

> *let resinkonall = inkon_all2 - (0.273501 + 0.171780*inkon_all1)*

> *regress*

> *model sqr_bdi_t2 = constant sqr_bdi_t1*

> *estimate*

[▼OLS Regression](file:///\\Untitled.syo)

1 case(s) are deleted due to missing data.

| Dependent Variable | SQR_BDI_T2 |
| --- | --- |
| N | 73 |
| Multiple R | 0.695158 |
| Squared Multiple R | 0.483244 |
| Adjusted Squared Multiple R | 0.475966 |
| Standard Error of Estimate | 0.712276 |

| **Regression Coefficients B = (X'X)^-1^X'Y** | | | | | | |
| --- | --- | --- | --- | --- | --- | --- |
| **Effect** | **Coefficient** | **Standard Error** | **Std. Coefficient** | **Tolerance** | **t** | **p-Value** |
| CONSTANT | 0.898630 | 0.235429 | 0.000000 | . | 3.816986 | 0.000286 |
| SQR_BDI_T1 | 0.627624 | 0.077025 | 0.695158 | 1.000000 | 8.148354 | 0.000000 |

| **Analysis of Variance** | | | | | |
| --- | --- | --- | --- | --- | --- |
| **Source** | **SS** | **df** | **Mean Squares** | **F-Ratio** | **p-Value** |
| Regression | 33.684998 | 1 | 33.684998 | 66.395666 | 0.000000 |
| Residual | 36.020948 | 71 | 0.507337 |  |  |

WARNING

| Case | 406.000000 | is an Outlier | (Studentized Residual | : | 3.572117) |
| --- | --- | --- | --- | --- | --- |

| Durbin-Watson D-Statistic | 1.705335 |
| --- | --- |
| First Order Autocorrelation | 0.099159 |

| **Information Criteria** | |
| --- | --- |
| AIC | 161.600835 |
| AIC (Corrected) | 161.948661 |
| Schwarz's BIC | 168.472213 |

> *let resbdi = sqr_bdi_t2 - (0.898630 + 0.627624*sqr_bdi_t1)*

> *regress*

> *model ht2 = constant ht1*

> *estimate*

[▼OLS Regression](file:///\\Untitled.syo)

1 case(s) are deleted due to missing data.

| Dependent Variable | HT2 |
| --- | --- |
| N | 73 |
| Multiple R | 0.292982 |
| Squared Multiple R | 0.085839 |
| Adjusted Squared Multiple R | 0.072963 |
| Standard Error of Estimate | 0.675449 |

| **Regression Coefficients B = (X'X)^-1^X'Y** | | | | | | |
| --- | --- | --- | --- | --- | --- | --- |
| **Effect** | **Coefficient** | **Standard Error** | **Std. Coefficient** | **Tolerance** | **t** | **p-Value** |
| CONSTANT | 2.744871 | 0.358751 | 0.000000 | . | 7.651183 | 0.000000 |
| HT1 | 0.255568 | 0.098980 | 0.292982 | 1.000000 | 2.582015 | 0.011886 |

| **Analysis of Variance** | | | | | |
| --- | --- | --- | --- | --- | --- |
| **Source** | **SS** | **df** | **Mean Squares** | **F-Ratio** | **p-Value** |
| Regression | 3.041600 | 1 | 3.041600 | 6.666803 | 0.011886 |
| Residual | 32.392380 | 71 | 0.456231 |  |  |

WARNING

| Case | 105.000000 | is an Outlier | (Studentized Residual | : | -3.537074) |
| --- | --- | --- | --- | --- | --- |

| Durbin-Watson D-Statistic | 1.787679 |
| --- | --- |
| First Order Autocorrelation | 0.055845 |

| **Information Criteria** | |
| --- | --- |
| AIC | 153.849881 |
| AIC (Corrected) | 154.197707 |
| Schwarz's BIC | 160.721260 |

> *let resht = ht2 - (2.744871 + 0.255568*ht1)*

> *regress*

> *model swls_t2 = constant swls_t1*

> *estimate*

[▼OLS Regression](file:///\\Untitled.syo)

| Dependent Variable | SWLS_T2 |
| --- | --- |
| N | 74 |
| Multiple R | 0.853828 |
| Squared Multiple R | 0.729023 |
| Adjusted Squared Multiple R | 0.725259 |
| Standard Error of Estimate | 0.553284 |

| **Regression Coefficients B = (X'X)^-1^X'Y** | | | | | | |
| --- | --- | --- | --- | --- | --- | --- |
| **Effect** | **Coefficient** | **Standard Error** | **Std. Coefficient** | **Tolerance** | **t** | **p-Value** |
| CONSTANT | 0.705340 | 0.305327 | 0.000000 | . | 2.310114 | 0.023751 |
| SWLS_T1 | 0.872813 | 0.062712 | 0.853828 | 1.000000 | 13.917792 | 0.000000 |

| **Analysis of Variance** | | | | | |
| --- | --- | --- | --- | --- | --- |
| **Source** | **SS** | **df** | **Mean Squares** | **F-Ratio** | **p-Value** |
| Regression | 59.297526 | 1 | 59.297526 | 193.704933 | 0.000000 |
| Residual | 22.040852 | 72 | 0.306123 |  |  |

WARNING

| Case | 207.000000 | is an Outlier | (Studentized Residual | : | -3.284301) |
| --- | --- | --- | --- | --- | --- |

| Durbin-Watson D-Statistic | 2.263446 |
| --- | --- |
| First Order Autocorrelation | -0.132901 |

| **Information Criteria** | |
| --- | --- |
| AIC | 126.376513 |
| AIC (Corrected) | 126.719370 |
| Schwarz's BIC | 133.288708 |

> *let resswls = swls_t2 - (0.705340 + 0.872813*swls_t1)*

> *format 12, 8*

> *regress*

> *model inkon_ag2 = constant inkon_ag1*

> *estimate*

[▼OLS Regression](file:///\\Untitled.syo)

| Dependent Variable | INKON_AG2 |
| --- | --- |
| N | 74 |
| Multiple R | 0.21139690 |
| Squared Multiple R | 0.04468865 |
| Adjusted Squared Multiple R | 0.03142044 |
| Standard Error of Estimate | 0.37141137 |

| **Regression Coefficients B = (X'X)^-1^X'Y** | | | | | | |
| --- | --- | --- | --- | --- | --- | --- |
| **Effect** | **Coefficient** | **Standard Error** | **Std. Coefficient** | **Tolerance** | **t** | **p-Value** |
| CONSTANT | 0.21142554 | 0.06911031 | 0.00000000 | . | 3.05924746 | 0.00311558 |
| INKON_AG1 | 0.25265670 | 0.13766972 | 0.21139690 | 1.00000000 | 1.83523802 | 0.07060058 |

| **Analysis of Variance** | | | | | |
| --- | --- | --- | --- | --- | --- |
| **Source** | **SS** | **df** | **Mean Squares** | **F-Ratio** | **p-Value** |
| Regression | 0.46461711 | 1 | 0.46461711 | 3.36809858 | 0.07060058 |
| Residual | 9.93214148 | 72 | 0.13794641 |  |  |

| Durbin-Watson D-Statistic | 2.05305643 |
| --- | --- |
| First Order Autocorrelation | -0.03364721 |

| **Information Criteria** | |
| --- | --- |
| AIC | 67.38951835 |
| AIC (Corrected) | 67.73237549 |
| Schwarz's BIC | 74.30171363 |

> *Let resinkonag = inkon_ag2 - (0.21142554 + 0.25265670 * inkon_ag1)*

> *regress*

> *model agkon2 = constant agkon1*

> *estimate*

[▼OLS Regression](file:///\\Untitled.syo)

| Dependent Variable | AGKON2 |
| --- | --- |
| N | 74 |
| Multiple R | 0.28928132 |
| Squared Multiple R | 0.08368368 |
| Adjusted Squared Multiple R | 0.07095706 |
| Standard Error of Estimate | 0.70865039 |

| **Regression Coefficients B = (X'X)^-1^X'Y** | | | | | | |
| --- | --- | --- | --- | --- | --- | --- |
| **Effect** | **Coefficient** | **Standard Error** | **Std. Coefficient** | **Tolerance** | **t** | **p-Value** |
| CONSTANT | 0.48283841 | 0.11890283 | 0.00000000 | . | 4.06078141 | 0.00012294 |
| AGKON1 | 0.30698033 | 0.11971447 | 0.28928132 | 1.00000000 | 2.56427100 | 0.01242553 |

| **Analysis of Variance** | | | | | |
| --- | --- | --- | --- | --- | --- |
| **Source** | **SS** | **df** | **Mean Squares** | **F-Ratio** | **p-Value** |
| Regression | 3.30211276 | 1 | 3.30211276 | 6.57548576 | 0.01242553 |
| Residual | 36.15734670 | 72 | 0.50218537 |  |  |

| Durbin-Watson D-Statistic | 1.66559408 |
| --- | --- |
| First Order Autocorrelation | 0.15535272 |

| **Information Criteria** | |
| --- | --- |
| AIC | 163.00521758 |
| AIC (Corrected) | 163.34807472 |
| Schwarz's BIC | 169.91741286 |

> *Let resagkon = agkon2 - (0.48283841 + 0.30698033 * agkon1)*

> *begin*

> *thick=1.5*

> *write "r = .22, p = .06" /LOC = {0.4in, 3.55in}*

> *write "r = -.22, p = .06" /LOC = {0.4in, -0.65in}*

> *write "r = .09, p = .44" /LOC = {0.4in, -8.2in}*

> *write "r = .21, p = .07" /LOC = {4.6in, 3.55in}*

> *write "r = -.27, p = .02" /LOC = {4.6in, -0.65in}*

> *write "r = .01, p = .92" /LOC = {4.6in, -8.2in}*

> *write "r = -.16, p = .18" /LOC = {10.6in, 3.55in}*

> *write "r = .17, p = .16" /LOC = {10.6in, -0.65in}*

> *write "r = .20, p = .09" /LOC = {10.6in, -8.2in}*

> *write "r = -.26, p = .02" /LOC = {14.8in, 3.55in}*

> *write "r = .27, p = .02" /LOC = {14.8in, -0.65in}*

> *write "r = .18, p = .11" /LOC = {14.8in, -8.2in}*

> *plot resht*reskongruenz /Smooth=linear short scale = left COLOR = {Black} SYMBOL = {'CIRCLE'} xlabel= " " ylabel= "HT change"*

> *plot resbdi*reskongruenz /Smooth=linear short location = {0in, -4.2in} scale = left COLOR = {Black} SYMBOL = {'CIRCLE'} xlabel= " " ylabel= "BDI change"*

> *plot resswls*reskongruenz /Smooth=linear short location={0in, -8.4in} scale =L COLOR = {Black} SYMBOL = {'CIRCLE'} xlabel= "Overall congruence change" ylabel= "SWLS
change"*

> *plot resht*resagkon /Smooth=linear short location= {4.2in, 0in} scale = none COLOR = {Black} SYMBOL = {'CIRCLE'} xlabel= " " ylabel= " "*

> *plot resbdi*resagkon /Smooth=linear short location = {4.2in, -4.2in} scale = none COLOR = {Black} SYMBOL = {'CIRCLE'} xlabel= " " ylabel= " "*

> *plot resswls*resagkon /Smooth=linear short location={4.2in, -8.4in} scale =bottom COLOR = {Black} SYMBOL = {'CIRCLE'} xlabel= "Agentic congruence change" ylabel= " "*

> *plot resht*resinkonall /Smooth=linear short location= {8.4in, 0in} scale = none COLOR = {Black} SYMBOL = {'CIRCLE'} xlabel= " " ylabel= " "*

> *plot resbdi*resinkonall /Smooth=linear short location = {8.4in, -4.2in} scale = none COLOR = {Black} SYMBOL = {'CIRCLE'} xlabel= " " ylabel= " "*

> *plot resswls*resinkonall /Smooth=linear short location={8.4in, -8.4in} scale =bottom COLOR = {Black} SYMBOL = {'CIRCLE'} xlabel= "Overall incongruence change" ylabel= "
"*

> *plot resht*resinkonag /Smooth=linear short location= {12.6in, 0in} scale = none COLOR = {Black} SYMBOL = {'CIRCLE'} xlabel= " " ylabel= " "*

> *plot resbdi*resinkonag /Smooth=linear short location = {12.6in, -4.2in} scale = none COLOR = {Black} SYMBOL = {'CIRCLE'} xlabel= " " ylabel= " "*

> *plot resswls*resinkonag /Smooth=linear short location={12.6in, -8.4in} scale =bottom COLOR = {Black} SYMBOL = {'CIRCLE'} xlabel= "Agentic incongruence change" ylabel= "
"*

> *end*

[▼Begin/End Plot](file:///\\Untitled.syo)


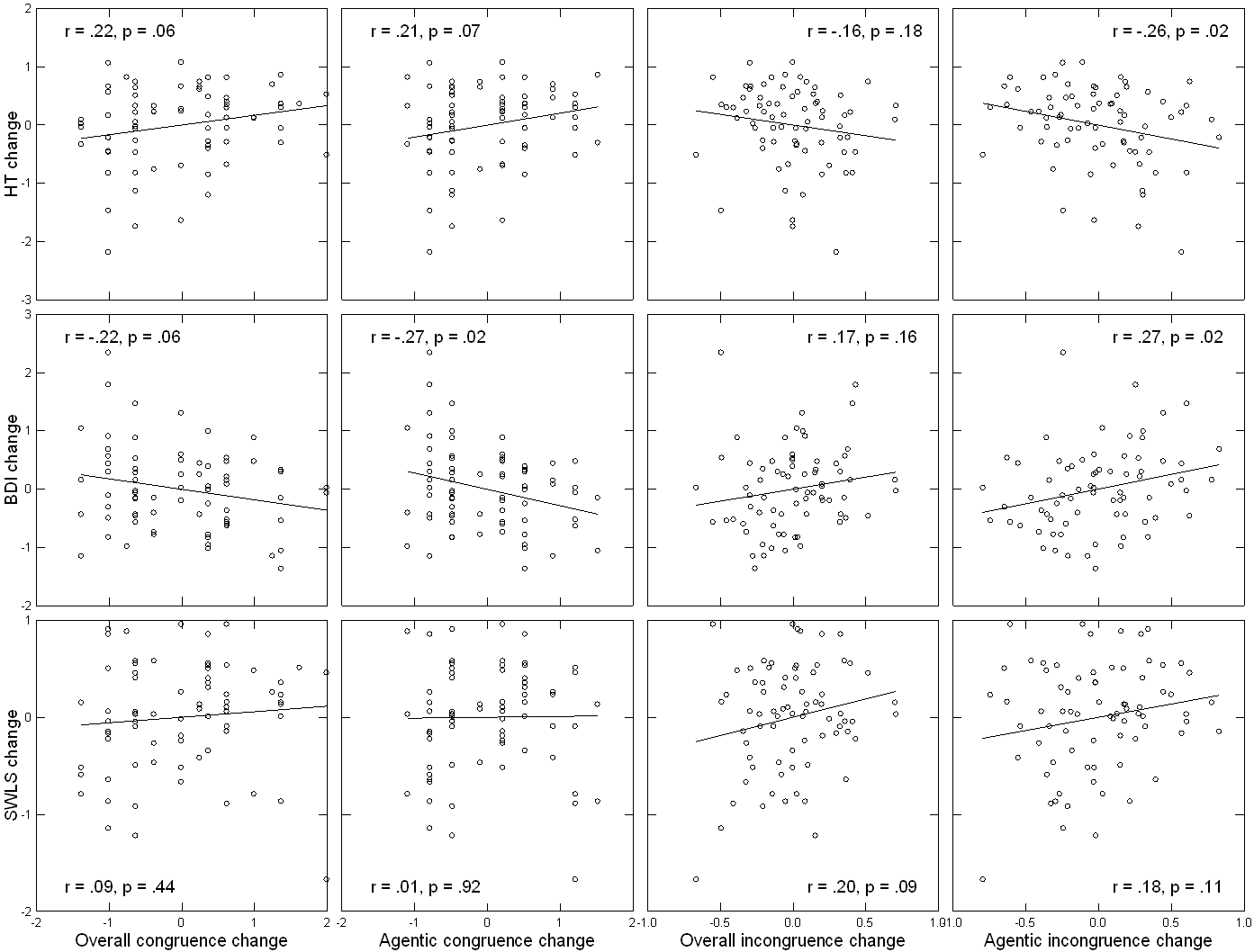


> *setcor*

> *model ht2 | ht1 = kongruenz2 | kongruenz1*

> *estimate*

[▼Set and Canonical Correlations](file:///\\Untitled.syo)

The categorical values encountered during processing are

| **Variables** | **Levels** | | |
| --- | --- | --- | --- |
| CONDITION (3 levels) | Control | Feedback | Feedback + CET |
| TREAT (2 levels) | CG | FB/FB+CET |  |

1 Cases deleted due to missing data.

**Bipartial Set Correlation Analysis (Y|YPARTIAL vs. X|XPARTIAL)**

Number of Cases on which Analysis is based: 73

Dependent Set y Partialled by these Variables

HT1

Independent Set x Partialled by these Variables

KONGRUENZ1

| RAO F | : | 3.74871428 |  |  |
| --- | --- | --- | --- | --- |
| df | : | 1.00000000 | , | 70.00000000 |
| p-Value | : | 0.05688662 |  |  |

| R-square | : | 0.04786489 | Shrunk R-square | : | 0.03426296 |
| --- | --- | --- | --- | --- | --- |
| T-square | : | 0.04786489 | Shrunk T-square | : | 0.03426296 |
| P-square | : | 0.04786489 | Shrunk P-square | : | 0.03426296 |

| **Within Basic Set y Correlations** | |
| --- | --- |
|  | **HT2** |
| HT2 | 1.00000000 |

| **Within Basic Set x Correlations** | |
| --- | --- |
|  | **KONGRUENZ2** |
| KONGRUENZ2 | 1.00000000 |

| **Between Basic y (col) and Basic x (row) Correlations** | |
| --- | --- |
|  | **HT2** |
| KONGRUENZ2 | 0.21878045 |

| **Estimated (from x-set) y Intercorrelations (R-square on diagonal)** | |
| --- | --- |
|  | **HT2** |
| HT2 | 0.04786489 |

| **Significance Tests for Prediction of Each Basic y Variable** | | |
| --- | --- | --- |
| **Variable** | **F-Ratio** | **p-Value** |
| HT2 | 3.74871428 | 0.05688662 |

| **Betas Predicting Basic y (col) from Basic x (row) Variables** | |
| --- | --- |
|  | **HT2** |
| KONGRUENZ2 | 0.21878045 |

| **Standard Error of Betas** | |
| --- | --- |
|  | **HT2** |
| KONGRUENZ2 | 0.11299711 |

| **t-Statistic for Betas** | |
| --- | --- |
|  | **HT2** |
| KONGRUENZ2 | 1.93615967 |

| **p-Value for Betas** | |
| --- | --- |
|  | **HT2** |
| KONGRUENZ2 | 0.05688662 |

> *!! bp r = .22, p = .06*

> *setcor*

> *model sqr_bdi_t2 | sqr_bdi_t1 = kongruenz2 | kongruenz1*

> *estimate*

[▼Set and Canonical Correlations](file:///\\Untitled.syo)

The categorical values encountered during processing are

| **Variables** | **Levels** | | |
| --- | --- | --- | --- |
| CONDITION (3 levels) | Control | Feedback | Feedback + CET |
| TREAT (2 levels) | CG | FB/FB+CET |  |

1 Cases deleted due to missing data.

**Bipartial Set Correlation Analysis (Y|YPARTIAL vs. X|XPARTIAL)**

Number of Cases on which Analysis is based: 73

Dependent Set y Partialled by these Variables

SQR_BDI_T1

Independent Set x Partialled by these Variables

KONGRUENZ1

| RAO F | : | 3.62605768 |  |  |
| --- | --- | --- | --- | --- |
| df | : | 1.00000000 | , | 70.00000000 |
| p-Value | : | 0.06099178 |  |  |

| R-square | : | 0.04886794 | Shrunk R-square | : | 0.03528034 |
| --- | --- | --- | --- | --- | --- |
| T-square | : | 0.04886794 | Shrunk T-square | : | 0.03528034 |
| P-square | : | 0.04886794 | Shrunk P-square | : | 0.03528034 |

| **Within Basic Set y Correlations** | |
| --- | --- |
|  | **SQR_BDI_T2** |
| SQR_BDI_T2 | 1.00000000 |

| **Within Basic Set x Correlations** | |
| --- | --- |
|  | **KONGRUENZ2** |
| KONGRUENZ2 | 1.00000000 |

| **Between Basic y (col) and Basic x (row) Correlations** | |
| --- | --- |
|  | **SQR_BDI_T2** |
| KONGRUENZ2 | -0.22106094 |

| **Estimated (from x-set) y Intercorrelations (R-square on diagonal)** | |
| --- | --- |
|  | **SQR_BDI_T2** |
| SQR_BDI_T2 | 0.04886794 |

| **Significance Tests for Prediction of Each Basic y Variable** | | |
| --- | --- | --- |
| **Variable** | **F-Ratio** | **p-Value** |
| SQR_BDI_T2 | 3.62605768 | 0.06099178 |

| **Betas Predicting Basic y (col) from Basic x (row) Variables** | |
| --- | --- |
|  | **SQR_BDI_T2** |
| KONGRUENZ2 | -0.22106094 |

| **Standard Error of Betas** | |
| --- | --- |
|  | **SQR_BDI_T2** |
| KONGRUENZ2 | 0.11608996 |

| **t-Statistic for Betas** | |
| --- | --- |
|  | **SQR_BDI_T2** |
| KONGRUENZ2 | -1.90422102 |

| **p-Value for Betas** | |
| --- | --- |
|  | **SQR_BDI_T2** |
| KONGRUENZ2 | 0.06099178 |

> *!! bp r = -.22, p = .06*

> *setcor*

> *model swls_t2 | swls_t1 = kongruenz2 | kongruenz1*

> *estimate*

[▼Set and Canonical Correlations](file:///\\Untitled.syo)

The categorical values encountered during processing are

| **Variables** | **Levels** | | |
| --- | --- | --- | --- |
| CONDITION (3 levels) | Control | Feedback | Feedback + CET |
| TREAT (2 levels) | CG | FB/FB+CET |  |

**Bipartial Set Correlation Analysis (Y|YPARTIAL vs. X|XPARTIAL)**

Number of Cases on which Analysis is based: 74

Dependent Set y Partialled by these Variables

SWLS_T1

Independent Set x Partialled by these Variables

KONGRUENZ1

| RAO F | : | 0.59293849 |  |  |
| --- | --- | --- | --- | --- |
| df | : | 1.00000000 | , | 71.00000000 |
| p-Value | : | 0.44383961 |  |  |

| R-square | : | 0.00827377 | Shrunk R-square | : | 0.00000000 |
| --- | --- | --- | --- | --- | --- |
| T-square | : | 0.00827377 | Shrunk T-square | : | 0.00000000 |
| P-square | : | 0.00827377 | Shrunk P-square | : | 0.00000000 |

| **Within Basic Set y Correlations** | |
| --- | --- |
|  | **SWLS_T2** |
| SWLS_T2 | 1.00000000 |

| **Within Basic Set x Correlations** | |
| --- | --- |
|  | **KONGRUENZ2** |
| KONGRUENZ2 | 1.00000000 |

| **Between Basic y (col) and Basic x (row) Correlations** | |
| --- | --- |
|  | **SWLS_T2** |
| KONGRUENZ2 | 0.09096029 |

| **Estimated (from x-set) y Intercorrelations (R-square on diagonal)** | |
| --- | --- |
|  | **SWLS_T2** |
| SWLS_T2 | 0.00827377 |

| **Significance Tests for Prediction of Each Basic y Variable** | | |
| --- | --- | --- |
| **Variable** | **F-Ratio** | **p-Value** |
| SWLS_T2 | 0.59293849 | 0.44383961 |

| **Betas Predicting Basic y (col) from Basic x (row) Variables** | |
| --- | --- |
|  | **SWLS_T2** |
| KONGRUENZ2 | 0.09096029 |

| **Standard Error of Betas** | |
| --- | --- |
|  | **SWLS_T2** |
| KONGRUENZ2 | 0.11812641 |

| **t-Statistic for Betas** | |
| --- | --- |
|  | **SWLS_T2** |
| KONGRUENZ2 | 0.77002499 |

| **p-Value for Betas** | |
| --- | --- |
|  | **SWLS_T2** |
| KONGRUENZ2 | 0.44383961 |

> *!! bp r = .09, p = .44*

> *setcor*

> *model ht2 | ht1 = agkon2 | agkon1*

> *estimate*

[▼Set and Canonical Correlations](file:///\\Untitled.syo)

The categorical values encountered during processing are

| **Variables** | **Levels** | | |
| --- | --- | --- | --- |
| CONDITION (3 levels) | Control | Feedback | Feedback + CET |
| TREAT (2 levels) | CG | FB/FB+CET |  |

1 Cases deleted due to missing data.

**Bipartial Set Correlation Analysis (Y|YPARTIAL vs. X|XPARTIAL)**

Number of Cases on which Analysis is based: 73

Dependent Set y Partialled by these Variables

HT1

Independent Set x Partialled by these Variables

AGKON1

| RAO F | : | 3.43856662 |  |  |
| --- | --- | --- | --- | --- |
| df | : | 1.00000000 | , | 70.00000000 |
| p-Value | : | 0.06790358 |  |  |

| R-square | : | 0.04609729 | Shrunk R-square | : | 0.03247011 |
| --- | --- | --- | --- | --- | --- |
| T-square | : | 0.04609729 | Shrunk T-square | : | 0.03247011 |
| P-square | : | 0.04609729 | Shrunk P-square | : | 0.03247011 |

| **Within Basic Set y Correlations** | |
| --- | --- |
|  | **HT2** |
| HT2 | 1.00000000 |

| **Within Basic Set x Correlations** | |
| --- | --- |
|  | **AGKON2** |
| AGKON2 | 1.00000000 |

| **Between Basic y (col) and Basic x (row) Correlations** | |
| --- | --- |
|  | **HT2** |
| AGKON2 | 0.21470280 |

| **Estimated (from x-set) y Intercorrelations (R-square on diagonal)** | |
| --- | --- |
|  | **HT2** |
| HT2 | 0.04609729 |

| **Significance Tests for Prediction of Each Basic y Variable** | | |
| --- | --- | --- |
| **Variable** | **F-Ratio** | **p-Value** |
| HT2 | 3.43856662 | 0.06790358 |

| **Betas Predicting Basic y (col) from Basic x (row) Variables** | |
| --- | --- |
|  | **HT2** |
| AGKON2 | 0.21470280 |

| **Standard Error of Betas** | |
| --- | --- |
|  | **HT2** |
| AGKON2 | 0.11578412 |

| **t-Statistic for Betas** | |
| --- | --- |
|  | **HT2** |
| AGKON2 | 1.85433724 |

| **p-Value for Betas** | |
| --- | --- |
|  | **HT2** |
| AGKON2 | 0.06790358 |

> *!! bp r = .21, p = .07*

> *setcor*

> *model sqr_bdi_t2 | sqr_bdi_t1 = agkon2 | agkon1*

> *estimate*

[▼Set and Canonical Correlations](file:///\\Untitled.syo)

The categorical values encountered during processing are

| **Variables** | **Levels** | | |
| --- | --- | --- | --- |
| CONDITION (3 levels) | Control | Feedback | Feedback + CET |
| TREAT (2 levels) | CG | FB/FB+CET |  |

1 Cases deleted due to missing data.

**Bipartial Set Correlation Analysis (Y|YPARTIAL vs. X|XPARTIAL)**

Number of Cases on which Analysis is based: 73

Dependent Set y Partialled by these Variables

SQR_BDI_T1

Independent Set x Partialled by these Variables

AGKON1

| RAO F | : | 5.72640149 |  |  |
| --- | --- | --- | --- | --- |
| df | : | 1.00000000 | , | 70.00000000 |
| p-Value | : | 0.01939776 |  |  |

| R-square | : | 0.07538450 | Shrunk R-square | : | 0.06217571 |
| --- | --- | --- | --- | --- | --- |
| T-square | : | 0.07538450 | Shrunk T-square | : | 0.06217571 |
| P-square | : | 0.07538450 | Shrunk P-square | : | 0.06217571 |

| **Within Basic Set y Correlations** | |
| --- | --- |
|  | **SQR_BDI_T2** |
| SQR_BDI_T2 | 1.00000000 |

| **Within Basic Set x Correlations** | |
| --- | --- |
|  | **AGKON2** |
| AGKON2 | 1.00000000 |

| **Between Basic y (col) and Basic x (row) Correlations** | |
| --- | --- |
|  | **SQR_BDI_T2** |
| AGKON2 | -0.27456238 |

| **Estimated (from x-set) y Intercorrelations (R-square on diagonal)** | |
| --- | --- |
|  | **SQR_BDI_T2** |
| SQR_BDI_T2 | 0.07538450 |

| **Significance Tests for Prediction of Each Basic y Variable** | | |
| --- | --- | --- |
| **Variable** | **F-Ratio** | **p-Value** |
| SQR_BDI_T2 | 5.72640149 | 0.01939776 |

| **Betas Predicting Basic y (col) from Basic x (row) Variables** | |
| --- | --- |
|  | **SQR_BDI_T2** |
| AGKON2 | -0.27456238 |

| **Standard Error of Betas** | |
| --- | --- |
|  | **SQR_BDI_T2** |
| AGKON2 | 0.11473611 |

| **t-Statistic for Betas** | |
| --- | --- |
|  | **SQR_BDI_T2** |
| AGKON2 | -2.39299007 |

| **p-Value for Betas** | |
| --- | --- |
|  | **SQR_BDI_T2** |
| AGKON2 | 0.01939776 |

> *!! bp r = -.27, p = .02*

> *setcor*

> *model swls_t2 | swls_t1 = agkon2 | agkon1*

> *estimate*

[▼Set and Canonical Correlations](file:///\\Untitled.syo)

The categorical values encountered during processing are

| **Variables** | **Levels** | | |
| --- | --- | --- | --- |
| CONDITION (3 levels) | Control | Feedback | Feedback + CET |
| TREAT (2 levels) | CG | FB/FB+CET |  |

**Bipartial Set Correlation Analysis (Y|YPARTIAL vs. X|XPARTIAL)**

Number of Cases on which Analysis is based: 74

Dependent Set y Partialled by these Variables

SWLS_T1

Independent Set x Partialled by these Variables

AGKON1

| RAO F | : | 0.00904232 |  |  |
| --- | --- | --- | --- | --- |
| df | : | 1.00000000 | , | 71.00000000 |
| p-Value | : | 0.92451033 |  |  |

| R-square | : | 0.00012465 | Shrunk R-square | : | 0.00000000 |
| --- | --- | --- | --- | --- | --- |
| T-square | : | 0.00012465 | Shrunk T-square | : | 0.00000000 |
| P-square | : | 0.00012465 | Shrunk P-square | : | 0.00000000 |

| **Within Basic Set y Correlations** | |
| --- | --- |
|  | **SWLS_T2** |
| SWLS_T2 | 1.00000000 |

| **Within Basic Set x Correlations** | |
| --- | --- |
|  | **AGKON2** |
| AGKON2 | 1.00000000 |

| **Between Basic y (col) and Basic x (row) Correlations** | |
| --- | --- |
|  | **SWLS_T2** |
| AGKON2 | 0.01116449 |

| **Estimated (from x-set) y Intercorrelations (R-square on diagonal)** | |
| --- | --- |
|  | **SWLS_T2** |
| SWLS_T2 | 0.00012465 |

| **Significance Tests for Prediction of Each Basic y Variable** | | |
| --- | --- | --- |
| **Variable** | **F-Ratio** | **p-Value** |
| SWLS_T2 | 0.00904232 | 0.92451033 |

| **Betas Predicting Basic y (col) from Basic x (row) Variables** | |
| --- | --- |
|  | **SWLS_T2** |
| AGKON2 | 0.01116449 |

| **Standard Error of Betas** | |
| --- | --- |
|  | **SWLS_T2** |
| AGKON2 | 0.11740834 |

| **t-Statistic for Betas** | |
| --- | --- |
|  | **SWLS_T2** |
| AGKON2 | 0.09509111 |

| **p-Value for Betas** | |
| --- | --- |
|  | **SWLS_T2** |
| AGKON2 | 0.92451033 |

> *!! bp r = .01, p = .92*

> *setcor*

> *model ht2 | ht1 = inkon_all2 | inkon_all1*

> *estimate*

[▼Set and Canonical Correlations](file:///\\Untitled.syo)

The categorical values encountered during processing are

| **Variables** | **Levels** | | |
| --- | --- | --- | --- |
| CONDITION (3 levels) | Control | Feedback | Feedback + CET |
| TREAT (2 levels) | CG | FB/FB+CET |  |

1 Cases deleted due to missing data.

**Bipartial Set Correlation Analysis (Y|YPARTIAL vs. X|XPARTIAL)**

Number of Cases on which Analysis is based: 73

Dependent Set y Partialled by these Variables

HT1

Independent Set x Partialled by these Variables

INKON_ALL1

| RAO F | : | 1.85246345 |  |  |
| --- | --- | --- | --- | --- |
| df | : | 1.00000000 | , | 70.00000000 |
| p-Value | : | 0.17786277 |  |  |

| R-square | : | 0.02459381 | Shrunk R-square | : | 0.01065944 |
| --- | --- | --- | --- | --- | --- |
| T-square | : | 0.02459381 | Shrunk T-square | : | 0.01065944 |
| P-square | : | 0.02459381 | Shrunk P-square | : | 0.01065944 |

| **Within Basic Set y Correlations** | |
| --- | --- |
|  | **HT2** |
| HT2 | 1.00000000 |

| **Within Basic Set x Correlations** | |
| --- | --- |
|  | **INKON_ALL2** |
| INKON_ALL2 | 1.00000000 |

| **Between Basic y (col) and Basic x (row) Correlations** | |
| --- | --- |
|  | **HT2** |
| INKON_ALL2 | -0.15682414 |

| **Estimated (from x-set) y Intercorrelations (R-square on diagonal)** | |
| --- | --- |
|  | **HT2** |
| HT2 | 0.02459381 |

| **Significance Tests for Prediction of Each Basic y Variable** | | |
| --- | --- | --- |
| **Variable** | **F-Ratio** | **p-Value** |
| HT2 | 1.85246345 | 0.17786277 |

| **Betas Predicting Basic y (col) from Basic x (row) Variables** | |
| --- | --- |
|  | **HT2** |
| INKON_ALL2 | -0.15682414 |

| **Standard Error of Betas** | |
| --- | --- |
|  | **HT2** |
| INKON_ALL2 | 0.11522271 |

| **t-Statistic for Betas** | |
| --- | --- |
|  | **HT2** |
| INKON_ALL2 | -1.36105233 |

| **p-Value for Betas** | |
| --- | --- |
|  | **HT2** |
| INKON_ALL2 | 0.17786277 |

> *!! bp r = -.16, p = .18*

> *setcor*

> *model sqr_bdi_t2 | sqr_bdi_t1 = inkon_all2 | inkon_all1*

> *estimate*

[▼Set and Canonical Correlations](file:///\\Untitled.syo)

The categorical values encountered during processing are

| **Variables** | **Levels** | | |
| --- | --- | --- | --- |
| CONDITION (3 levels) | Control | Feedback | Feedback + CET |
| TREAT (2 levels) | CG | FB/FB+CET |  |

1 Cases deleted due to missing data.

**Bipartial Set Correlation Analysis (Y|YPARTIAL vs. X|XPARTIAL)**

Number of Cases on which Analysis is based: 73

Dependent Set y Partialled by these Variables

SQR_BDI_T1

Independent Set x Partialled by these Variables

INKON_ALL1

| RAO F | : | 2.05718987 |  |  |
| --- | --- | --- | --- | --- |
| df | : | 1.00000000 | , | 70.00000000 |
| p-Value | : | 0.15594170 |  |  |

| R-square | : | 0.02824595 | Shrunk R-square | : | 0.01436374 |
| --- | --- | --- | --- | --- | --- |
| T-square | : | 0.02824595 | Shrunk T-square | : | 0.01436374 |
| P-square | : | 0.02824595 | Shrunk P-square | : | 0.01436374 |

| **Within Basic Set y Correlations** | |
| --- | --- |
|  | **SQR_BDI_T2** |
| SQR_BDI_T2 | 1.00000000 |

| **Within Basic Set x Correlations** | |
| --- | --- |
|  | **INKON_ALL2** |
| INKON_ALL2 | 1.00000000 |

| **Between Basic y (col) and Basic x (row) Correlations** | |
| --- | --- |
|  | **SQR_BDI_T2** |
| INKON_ALL2 | 0.16806530 |

| **Estimated (from x-set) y Intercorrelations (R-square on diagonal)** | |
| --- | --- |
|  | **SQR_BDI_T2** |
| SQR_BDI_T2 | 0.02824595 |

| **Significance Tests for Prediction of Each Basic y Variable** | | |
| --- | --- | --- |
| **Variable** | **F-Ratio** | **p-Value** |
| SQR_BDI_T2 | 2.05718987 | 0.15594170 |

| **Betas Predicting Basic y (col) from Basic x (row) Variables** | |
| --- | --- |
|  | **SQR_BDI_T2** |
| INKON_ALL2 | 0.16806530 |

| **Standard Error of Betas** | |
| --- | --- |
|  | **SQR_BDI_T2** |
| INKON_ALL2 | 0.11717659 |

| **t-Statistic for Betas** | |
| --- | --- |
|  | **SQR_BDI_T2** |
| INKON_ALL2 | 1.43429072 |

| **p-Value for Betas** | |
| --- | --- |
|  | **SQR_BDI_T2** |
| INKON_ALL2 | 0.15594170 |

> *!! bp r = .17, p = .16*

> *setcor*

> *model swls_t2 | swls_t1 = inkon_all2 | inkon_all1*

> *estimate*

[▼Set and Canonical Correlations](file:///\\Untitled.syo)

The categorical values encountered during processing are

| **Variables** | **Levels** | | |
| --- | --- | --- | --- |
| CONDITION (3 levels) | Control | Feedback | Feedback + CET |
| TREAT (2 levels) | CG | FB/FB+CET |  |

**Bipartial Set Correlation Analysis (Y|YPARTIAL vs. X|XPARTIAL)**

Number of Cases on which Analysis is based: 74

Dependent Set y Partialled by these Variables

SWLS_T1

Independent Set x Partialled by these Variables

INKON_ALL1

| RAO F | : | 2.93613507 |  |  |
| --- | --- | --- | --- | --- |
| df | : | 1.00000000 | , | 71.00000000 |
| p-Value | : | 0.09098112 |  |  |

| R-square | : | 0.03857772 | Shrunk R-square | : | 0.02503656 |
| --- | --- | --- | --- | --- | --- |
| T-square | : | 0.03857772 | Shrunk T-square | : | 0.02503656 |
| P-square | : | 0.03857772 | Shrunk P-square | : | 0.02503656 |

| **Within Basic Set y Correlations** | |
| --- | --- |
|  | **SWLS_T2** |
| SWLS_T2 | 1.00000000 |

| **Within Basic Set x Correlations** | |
| --- | --- |
|  | **INKON_ALL2** |
| INKON_ALL2 | 1.00000000 |

| **Between Basic y (col) and Basic x (row) Correlations** | |
| --- | --- |
|  | **SWLS_T2** |
| INKON_ALL2 | 0.19641212 |

| **Estimated (from x-set) y Intercorrelations (R-square on diagonal)** | |
| --- | --- |
|  | **SWLS_T2** |
| SWLS_T2 | 0.03857772 |

| **Significance Tests for Prediction of Each Basic y Variable** | | |
| --- | --- | --- |
| **Variable** | **F-Ratio** | **p-Value** |
| SWLS_T2 | 2.93613507 | 0.09098112 |

| **Betas Predicting Basic y (col) from Basic x (row) Variables** | |
| --- | --- |
|  | **SWLS_T2** |
| INKON_ALL2 | 0.19641212 |

| **Standard Error of Betas** | |
| --- | --- |
|  | **SWLS_T2** |
| INKON_ALL2 | 0.11462524 |

| **t-Statistic for Betas** | |
| --- | --- |
|  | **SWLS_T2** |
| INKON_ALL2 | 1.71351541 |

| **p-Value for Betas** | |
| --- | --- |
|  | **SWLS_T2** |
| INKON_ALL2 | 0.09098112 |

> *!! bp r = .20, p = .09*

> *setcor*

> *model ht2 | ht1 = inkon_ag2 | inkon_ag1*

> *estimate*

[▼Set and Canonical Correlations](file:///\\Untitled.syo)

The categorical values encountered during processing are

| **Variables** | **Levels** | | |
| --- | --- | --- | --- |
| CONDITION (3 levels) | Control | Feedback | Feedback + CET |
| TREAT (2 levels) | CG | FB/FB+CET |  |

1 Cases deleted due to missing data.

**Bipartial Set Correlation Analysis (Y|YPARTIAL vs. X|XPARTIAL)**

Number of Cases on which Analysis is based: 73

Dependent Set y Partialled by these Variables

HT1

Independent Set x Partialled by these Variables

INKON_AG1

| RAO F | : | 5.29738438 |  |  |
| --- | --- | --- | --- | --- |
| df | : | 1.00000000 | , | 70.00000000 |
| p-Value | : | 0.02433793 |  |  |

| R-square | : | 0.06629570 | Shrunk R-square | : | 0.05295707 |
| --- | --- | --- | --- | --- | --- |
| T-square | : | 0.06629570 | Shrunk T-square | : | 0.05295707 |
| P-square | : | 0.06629570 | Shrunk P-square | : | 0.05295707 |

| **Within Basic Set y Correlations** | |
| --- | --- |
|  | **HT2** |
| HT2 | 1.00000000 |

| **Within Basic Set x Correlations** | |
| --- | --- |
|  | **INKON_AG2** |
| INKON_AG2 | 1.00000000 |

| **Between Basic y (col) and Basic x (row) Correlations** | |
| --- | --- |
|  | **HT2** |
| INKON_AG2 | -0.25747952 |

| **Estimated (from x-set) y Intercorrelations (R-square on diagonal)** | |
| --- | --- |
|  | **HT2** |
| HT2 | 0.06629570 |

| **Significance Tests for Prediction of Each Basic y Variable** | | |
| --- | --- | --- |
| **Variable** | **F-Ratio** | **p-Value** |
| HT2 | 5.29738438 | 0.02433793 |

| **Betas Predicting Basic y (col) from Basic x (row) Variables** | |
| --- | --- |
|  | **HT2** |
| INKON_AG2 | -0.25747952 |

| **Standard Error of Betas** | |
| --- | --- |
|  | **HT2** |
| INKON_AG2 | 0.11186957 |

| **t-Statistic for Betas** | |
| --- | --- |
|  | **HT2** |
| INKON_AG2 | -2.30160474 |

| **p-Value for Betas** | |
| --- | --- |
|  | **HT2** |
| INKON_AG2 | 0.02433793 |

> *!! bp r = -.26, p = .02*

> *setcor*

> *model sqr_bdi_t2 | sqr_bdi_t1 = inkon_ag2 | inkon_ag1*

> *estimate*

[▼Set and Canonical Correlations](file:///\\Untitled.syo)

The categorical values encountered during processing are

| **Variables** | **Levels** | | |
| --- | --- | --- | --- |
| CONDITION (3 levels) | Control | Feedback | Feedback + CET |
| TREAT (2 levels) | CG | FB/FB+CET |  |

1 Cases deleted due to missing data.

**Bipartial Set Correlation Analysis (Y|YPARTIAL vs. X|XPARTIAL)**

Number of Cases on which Analysis is based: 73

Dependent Set y Partialled by these Variables

SQR_BDI_T1

Independent Set x Partialled by these Variables

INKON_AG1

| RAO F | : | 5.30767388 |  |  |
| --- | --- | --- | --- | --- |
| df | : | 1.00000000 | , | 70.00000000 |
| p-Value | : | 0.02420495 |  |  |

| R-square | : | 0.07029892 | Shrunk R-square | : | 0.05701747 |
| --- | --- | --- | --- | --- | --- |
| T-square | : | 0.07029892 | Shrunk T-square | : | 0.05701747 |
| P-square | : | 0.07029892 | Shrunk P-square | : | 0.05701747 |

| **Within Basic Set y Correlations** | |
| --- | --- |
|  | **SQR_BDI_T2** |
| SQR_BDI_T2 | 1.00000000 |

| **Within Basic Set x Correlations** | |
| --- | --- |
|  | **INKON_AG2** |
| INKON_AG2 | 1.00000000 |

| **Between Basic y (col) and Basic x (row) Correlations** | |
| --- | --- |
|  | **SQR_BDI_T2** |
| INKON_AG2 | 0.26513943 |

| **Estimated (from x-set) y Intercorrelations (R-square on diagonal)** | |
| --- | --- |
|  | **SQR_BDI_T2** |
| SQR_BDI_T2 | 0.07029892 |

| **Significance Tests for Prediction of Each Basic y Variable** | | |
| --- | --- | --- |
| **Variable** | **F-Ratio** | **p-Value** |
| SQR_BDI_T2 | 5.30767388 | 0.02420495 |

| **Betas Predicting Basic y (col) from Basic x (row) Variables** | |
| --- | --- |
|  | **SQR_BDI_T2** |
| INKON_AG2 | 0.26513943 |

| **Standard Error of Betas** | |
| --- | --- |
|  | **SQR_BDI_T2** |
| INKON_AG2 | 0.11508592 |

| **t-Statistic for Betas** | |
| --- | --- |
|  | **SQR_BDI_T2** |
| INKON_AG2 | 2.30383894 |

| **p-Value for Betas** | |
| --- | --- |
|  | **SQR_BDI_T2** |
| INKON_AG2 | 0.02420495 |

> *!! bp r = .27, p = .02*

> *setcor*

> *model swls_t2 | swls_t1 = inkon_ag2 | inkon_ag1*

> *estimate*

[▼Set and Canonical Correlations](file:///\\Untitled.syo)

The categorical values encountered during processing are

| **Variables** | **Levels** | | |
| --- | --- | --- | --- |
| CONDITION (3 levels) | Control | Feedback | Feedback + CET |
| TREAT (2 levels) | CG | FB/FB+CET |  |

**Bipartial Set Correlation Analysis (Y|YPARTIAL vs. X|XPARTIAL)**

Number of Cases on which Analysis is based: 74

Dependent Set y Partialled by these Variables

SWLS_T1

Independent Set x Partialled by these Variables

INKON_AG1

| RAO F | : | 2.58119762 |  |  |
| --- | --- | --- | --- | --- |
| df | : | 1.00000000 | , | 71.00000000 |
| p-Value | : | 0.11257845 |  |  |

| R-square | : | 0.03342348 | Shrunk R-square | : | 0.01980972 |
| --- | --- | --- | --- | --- | --- |
| T-square | : | 0.03342348 | Shrunk T-square | : | 0.01980972 |
| P-square | : | 0.03342348 | Shrunk P-square | : | 0.01980972 |

| **Within Basic Set y Correlations** | |
| --- | --- |
|  | **SWLS_T2** |
| SWLS_T2 | 1.00000000 |

| **Within Basic Set x Correlations** | |
| --- | --- |
|  | **INKON_AG2** |
| INKON_AG2 | 1.00000000 |

| **Between Basic y (col) and Basic x (row) Correlations** | |
| --- | --- |
|  | **SWLS_T2** |
| INKON_AG2 | 0.18282089 |

| **Estimated (from x-set) y Intercorrelations (R-square on diagonal)** | |
| --- | --- |
|  | **SWLS_T2** |
| SWLS_T2 | 0.03342348 |

| **Significance Tests for Prediction of Each Basic y Variable** | | |
| --- | --- | --- |
| **Variable** | **F-Ratio** | **p-Value** |
| SWLS_T2 | 2.58119762 | 0.11257845 |

| **Betas Predicting Basic y (col) from Basic x (row) Variables** | |
| --- | --- |
|  | **SWLS_T2** |
| INKON_AG2 | 0.18282089 |

| **Standard Error of Betas** | |
| --- | --- |
|  | **SWLS_T2** |
| INKON_AG2 | 0.11379290 |

| **t-Statistic for Betas** | |
| --- | --- |
|  | **SWLS_T2** |
| INKON_AG2 | 1.60661060 |

| **p-Value for Betas** | |
| --- | --- |
|  | **SWLS_T2** |
| INKON_AG2 | 0.11257845 |

> *!! bp r = .18, p = .11*

> *!!*

> *!!*

> *!!*

> *!! Testing hypothesis 1*

> *!!*

> *!!*

> *!!*

> *let hypoth1 = .*

> *if condition = 1 then let hypoth1 = 0*

> *if condition = 3 then let hypoth1 = 1*

> *categ condition treat hypoth1*

> *!! Testing congruence scores*

> *glm*

> *model iemach1 iemach2 iemaff1 iemaff2 iemind1 iemind2 = constant condition /repeat={3, 2} names={motive, time}*

> *estimate*

[▼General Linear Model](file:///\\Untitled.syo)

Effects coding used for categorical variables in model.

The categorical values encountered during processing are

| **Variables** | **Levels** | | |
| --- | --- | --- | --- |
| CONDITION (3 levels) | Control | Feedback | Feedback + CET |
| TREAT (2 levels) | CG | FB/FB+CET |  |
| HYPOTH1 (2 levels) | 0.00000000 | 1.00000000 |  |

N of Cases Processed: 74

| **Dependent Variable Means** | | | | | |
| --- | --- | --- | --- | --- | --- |
| **IEMACH1** | **IEMACH2** | **T1** | **T2** | **IEMIND1** | **IEMIND2** |
| 0.40540541 | 0.39189189 | 0.29729730 | 0.31081081 | 0.31081081 | 0.31081081 |

| **Repeated Measures Factors and Levels of Dependent Variables** | | | | | | |
| --- | --- | --- | --- | --- | --- | --- |
| **Within Factor** | **1** | **2** | **3** | **4** | **5** | **6** |
| MOTIVE | 1.00000000 | 1.00000000 | 2.00000000 | 2.00000000 | 3.00000000 | 3.00000000 |
| TIME | 1.00000000 | 2.00000000 | 1.00000000 | 2.00000000 | 1.00000000 | 2.00000000 |

**Univariate and Multivariate Repeated Measures Analysis**

| **Between Subjects** | | | | | |
| --- | --- | --- | --- | --- | --- |
| **Source** | **SS** | **df** | **Mean Squares** | **F-Ratio** | **p-Value** |
| CONDITION | 0.36248370 | 2 | 0.18124185 | 0.42948534 | 0.65252351 |
| Error | 29.96184063 | 71 | 0.42199776 |  |  |

| **Within Subjects** | | | | | | | |
| --- | --- | --- | --- | --- | --- | --- | --- |
| **Source** | **SS** | **df** | **Mean Squares** | **F-Ratio** | **p-Value** | **G-G** | **H-F** |
| MOTIVE | 0.82212820 | 2 | 0.41106410 | 1.95856856 | 0.14485221 | 0.14628611 | 0.14485221 |
| MOTIVE*CONDITION | 0.37273516 | 4 | 0.09318379 | 0.44398633 | 0.77663196 | 0.77109756 | 0.77663196 |
| Error | 29.80294052 | 142 | 0.20987986 |  |  |  |  |

| Greenhouse-Geisser Epsilon | 0.97077046 |
| --- | --- |
| Huynh-Feldt Epsilon | 1.00000000 |

| **Within Subjects** | | | | | | | |
| --- | --- | --- | --- | --- | --- | --- | --- |
| **Source** | **SS** | **df** | **Mean Squares** | **F-Ratio** | **p-Value** | **G-G** | **H-F** |
| TIME | 0.00334230 | 1 | 0.00334230 | 0.01837121 | 0.89256816 | . | . |
| TIME*CONDITION | 0.74953984 | 2 | 0.37476992 | 2.05995225 | 0.13501062 | . | . |
| Error | 12.91712682 | 71 | 0.18193136 |  |  |  |  |

| Greenhouse-Geisser Epsilon | . |
| --- | --- |
| Huynh-Feldt Epsilon | . |

| **Within Subjects** | | | | | | | |
| --- | --- | --- | --- | --- | --- | --- | --- |
| **Source** | **SS** | **df** | **Mean Squares** | **F-Ratio** | **p-Value** | **G-G** | **H-F** |
| MOTIVE*TIME | 0.00871150 | 2 | 0.00435575 | 0.02823020 | 0.97217000 | 0.97118322 | 0.97217000 |
| MOTIVE*TIME*CONDITION | 2.41007796 | 4 | 0.60251949 | 3.90501029 | 0.00486523 | 0.00504831 | 0.00486523 |
| Error | 21.90974186 | 142 | 0.15429396 |  |  |  |  |

| Greenhouse-Geisser Epsilon | 0.98841087 |
| --- | --- |
| Huynh-Feldt Epsilon | 1.00000000 |

**Multivariate Repeated Measures Analysis**

| **Test of: MOTIVE** | | | | | |
| --- | --- | --- | --- | --- | --- |
| **Statistic** | **Value** | **Hypothesis df** | **Error df** | **F-Ratio** | **p-Value** |
| Wilks's Lambda | 0.94124453 | 2 | 70 | 2.18481106 | 0.12011197 |
| Pillai Trace | 0.05875547 | 2 | 70 | 2.18481106 | 0.12011197 |
| Hotelling-Lawley Trace | 0.06242317 | 2 | 70 | 2.18481106 | 0.12011197 |

| **Test of: MOTIVE*CONDITION** | | | | | |
| --- | --- | --- | --- | --- | --- |
| **Statistic** | **Value** | **Hypothesis df** | **Error df** | **F-Ratio** | **p-Value** |
| Wilks's Lambda | 0.97886463 | 4 | 140 | 0.37583713 | 0.82558383 |
| Pillai Trace | 0.02115071 | 4 | 142 | 0.37943784 | 0.82304269 |
| Hotelling-Lawley Trace | 0.02157604 | 4 | 138 | 0.37218669 | 0.82815388 |

| **THETA** | **S** | **M** | **N** | **p-Value** |
| --- | --- | --- | --- | --- |
| 0.02039845 | 2 | -0.50000000 | 34.00000000 | 0.73061700 |

| **Test of: MOTIVE*TIME** | | | | | |
| --- | --- | --- | --- | --- | --- |
| **Statistic** | **Value** | **Hypothesis df** | **Error df** | **F-Ratio** | **p-Value** |
| Wilks's Lambda | 0.99916880 | 2 | 70 | 0.02911612 | 0.97131543 |
| Pillai Trace | 0.00083120 | 2 | 70 | 0.02911612 | 0.97131543 |
| Hotelling-Lawley Trace | 0.00083189 | 2 | 70 | 0.02911612 | 0.97131543 |

| **Test of: MOTIVE*TIME*CONDITION** | | | | | |
| --- | --- | --- | --- | --- | --- |
| **Statistic** | **Value** | **Hypothesis df** | **Error df** | **F-Ratio** | **p-Value** |
| Wilks's Lambda | 0.82857612 | 4 | 140 | 3.45048636 | 0.01008943 |
| Pillai Trace | 0.17458577 | 4 | 142 | 3.39528140 | 0.01098326 |
| Hotelling-Lawley Trace | 0.20307366 | 4 | 138 | 3.50302055 | 0.00931021 |

| **THETA** | **S** | **M** | **N** | **p-Value** |
| --- | --- | --- | --- | --- |
| 0.15406228 | 2 | -0.50000000 | 34.00000000 | 0.01182638 |

> *calc 0.749540 /( 0.749540 + 12.917127)*

[▼CALCULATE](file:///\\Untitled.syo)

0.05484439

> *calc 2.410078 / (2.410078+21.909742)*

[▼CALCULATE](file:///\\Untitled.syo)

0.09909934

> *!! Robust for destructive testing including all congruence-index-constituting variables?*

> *categ condition treat*

> *glm*

> *model iemach1 iemach2 iemaff1 iemaff2 iemind1 iemind2 = constant condition PGQ_ACH_C2 PGQ_ach_C1 PGQ_AFF_C1 PGQ_AFF_C2 PGQ_IND_C2 PGQ_IND_C1 cat1 cpt1 cit1 cat2 cpt2
cit2/repeat={3, 2} names={motive, time}*

> *estimate*

[▼General Linear Model](file:///\\Untitled.syo)

Effects coding used for categorical variables in model.

The categorical values encountered during processing are

| **Variables** | **Levels** | | |
| --- | --- | --- | --- |
| CONDITION (3 levels) | Control | Feedback | Feedback + CET |
| TREAT (2 levels) | CG | FB/FB+CET |  |
| HYPOTH1 (2 levels) | 0.00000000 | 1.00000000 |  |

N of Cases Processed: 74

| **Dependent Variable Means** | | | | | |
| --- | --- | --- | --- | --- | --- |
| **IEMACH1** | **IEMACH2** | **T1** | **T2** | **IEMIND1** | **IEMIND2** |
| 0.40540541 | 0.39189189 | 0.29729730 | 0.31081081 | 0.31081081 | 0.31081081 |

| **Repeated Measures Factors and Levels of Dependent Variables** | | | | | | |
| --- | --- | --- | --- | --- | --- | --- |
| **Within Factor** | **1** | **2** | **3** | **4** | **5** | **6** |
| MOTIVE | 1.00000000 | 1.00000000 | 2.00000000 | 2.00000000 | 3.00000000 | 3.00000000 |
| TIME | 1.00000000 | 2.00000000 | 1.00000000 | 2.00000000 | 1.00000000 | 2.00000000 |

**Univariate and Multivariate Repeated Measures Analysis**

| **Between Subjects** | | | | | |
| --- | --- | --- | --- | --- | --- |
| **Source** | **SS** | **df** | **Mean Squares** | **F-Ratio** | **p-Value** |
| CONDITION | 0.14596241 | 2 | 0.07298121 | 0.49311548 | 0.61321514 |
| PGQ_ACH_C2 | 0.02855023 | 1 | 0.02855023 | 0.19290664 | 0.66211281 |
| PGQ_ACH_C1 | 0.48780500 | 1 | 0.48780500 | 3.29597455 | 0.07453278 |
| PGQ_AFF_C1 | 0.08640935 | 1 | 0.08640935 | 0.58384606 | 0.44785384 |
| PGQ_AFF_C2 | 0.38778093 | 1 | 0.38778093 | 2.62013733 | 0.11084927 |
| PGQ_IND_C2 | 1.28763335 | 1 | 1.28763335 | 8.70021170 | 0.00455573 |
| PGQ_IND_C1 | 0.00023067 | 1 | 0.00023067 | 0.00155861 | 0.96864161 |
| CAT1 | 1.34892173 | 1 | 1.34892173 | 9.11432173 | 0.00374253 |
| CPT1 | 0.25823825 | 1 | 0.25823825 | 1.74485029 | 0.19162591 |
| CIT1 | 0.93933008 | 1 | 0.93933008 | 6.34681494 | 0.01448812 |
| CAT2 | 0.22790005 | 1 | 0.22790005 | 1.53986281 | 0.21954973 |
| CPT2 | 0.44229066 | 1 | 0.44229066 | 2.98844571 | 0.08909080 |
| CIT2 | 1.02669610 | 1 | 1.02669610 | 6.93712494 | 0.01076407 |
| Error | 8.73201368 | 59 | 0.14800023 |  |  |

| **Within Subjects** | | | | | | | |
| --- | --- | --- | --- | --- | --- | --- | --- |
| **Source** | **SS** | **df** | **Mean Squares** | **F-Ratio** | **p-Value** | **G-G** | **H-F** |
| MOTIVE | 0.24493832 | 2 | 0.12246916 | 0.98414303 | 0.37680584 | 0.37126271 | 0.37680584 |
| MOTIVE*CONDITION | 0.25340776 | 4 | 0.06335194 | 0.50908628 | 0.72913969 | 0.71417804 | 0.72913969 |
| MOTIVE*PGQ_ACH_C2 | 0.29364264 | 2 | 0.14682132 | 1.17983315 | 0.31092877 | 0.30845503 | 0.31092877 |
| MOTIVE*PGQ_ACH_C1 | 1.01186227 | 2 | 0.50593114 | 4.06558348 | 0.01961173 | 0.02264782 | 0.01961173 |
| MOTIVE*PGQ_AFF_C1 | 0.06177957 | 2 | 0.03088978 | 0.24822547 | 0.78059037 | 0.76235151 | 0.78059037 |
| MOTIVE*PGQ_AFF_C2 | 1.27969405 | 2 | 0.63984702 | 5.14171061 | 0.00722731 | 0.00890768 | 0.00722731 |
| MOTIVE*PGQ_IND_C2 | 0.48405181 | 2 | 0.24202591 | 1.94488234 | 0.14756092 | 0.15141721 | 0.14756092 |
| MOTIVE*PGQ_IND_C1 | 0.40789582 | 2 | 0.20394791 | 1.63889352 | 0.19858382 | 0.20083116 | 0.19858382 |
| MOTIVE*CAT1 | 0.55168386 | 2 | 0.27584193 | 2.21662260 | 0.11349484 | 0.11807011 | 0.11349484 |
| MOTIVE*CPT1 | 1.18314757 | 2 | 0.59157379 | 4.75379443 | 0.01033743 | 0.01244290 | 0.01033743 |
| MOTIVE*CIT1 | 1.33211989 | 2 | 0.66605995 | 5.35235355 | 0.00595617 | 0.00743646 | 0.00595617 |
| MOTIVE*CAT2 | 2.11458999 | 2 | 1.05729500 | 8.49625725 | 0.00035708 | 0.00054197 | 0.00035708 |
| MOTIVE*CPT2 | 1.13803379 | 2 | 0.56901689 | 4.57253077 | 0.01222836 | 0.01455807 | 0.01222836 |
| MOTIVE*CIT2 | 0.64719688 | 2 | 0.32359844 | 2.60038645 | 0.07849571 | 0.08333930 | 0.07849571 |
| Error | 14.68420812 | 118 | 0.12444244 |  |  |  |  |

| Greenhouse-Geisser Epsilon | 0.92050915 |
| --- | --- |
| Huynh-Feldt Epsilon | 1.00000000 |

| **Within Subjects** | | | | | | | |
| --- | --- | --- | --- | --- | --- | --- | --- |
| **Source** | **SS** | **df** | **Mean Squares** | **F-Ratio** | **p-Value** | **G-G** | **H-F** |
| TIME | 0.00018005 | 1 | 0.00018005 | 0.00170917 | 0.96716275 | . | . |
| TIME*CONDITION | 0.10655220 | 2 | 0.05327610 | 0.50574090 | 0.60564900 | . | . |
| TIME*PGQ_ACH_C2 | 0.03893330 | 1 | 0.03893330 | 0.36958719 | 0.54556250 | . | . |
| TIME*PGQ_ACH_C1 | 0.25422887 | 1 | 0.25422887 | 2.41335113 | 0.12565274 | . | . |
| TIME*PGQ_AFF_C1 | 0.22376534 | 1 | 0.22376534 | 2.12416605 | 0.15029395 | . | . |
| TIME*PGQ_AFF_C2 | 0.32955157 | 1 | 0.32955157 | 3.12837661 | 0.08210936 | . | . |
| TIME*PGQ_IND_C2 | 0.67473938 | 1 | 0.67473938 | 6.40518548 | 0.01406543 | . | . |
| TIME*PGQ_IND_C1 | 0.47671342 | 1 | 0.47671342 | 4.52535892 | 0.03758651 | . | . |
| TIME*CAT1 | 0.77811815 | 1 | 0.77811815 | 7.38654244 | 0.00861325 | . | . |
| TIME*CPT1 | 0.54550239 | 1 | 0.54550239 | 5.17836081 | 0.02651680 | . | . |
| TIME*CIT1 | 2.13690463 | 1 | 2.13690463 | 20.28526988 | 0.00003206 | . | . |
| TIME*CAT2 | 1.56931456 | 1 | 1.56931456 | 14.89723449 | 0.00028399 | . | . |
| TIME*CPT2 | 0.86541504 | 1 | 0.86541504 | 8.21523689 | 0.00574947 | . | . |
| TIME*CIT2 | 2.16448921 | 1 | 2.16448921 | 20.54712560 | 0.00002896 | . | . |
| Error | 6.21521793 | 59 | 0.10534268 |  |  |  |  |

| Greenhouse-Geisser Epsilon | . |
| --- | --- |
| Huynh-Feldt Epsilon | . |

| **Within Subjects** | | | | | | | |
| --- | --- | --- | --- | --- | --- | --- | --- |
| **Source** | **SS** | **df** | **Mean Squares** | **F-Ratio** | **p-Value** | **G-G** | **H-F** |
| MOTIVE*TIME | 0.03232745 | 2 | 0.01616373 | 0.16457295 | 0.84845023 | 0.84645998 | 0.84845023 |
| MOTIVE*TIME*CONDITION | 1.07465308 | 4 | 0.26866327 | 2.73542769 | 0.03209635 | 0.03260564 | 0.03209635 |
| MOTIVE*TIME*PGQ_ACH_C2 | 0.02088565 | 2 | 0.01044283 | 0.10632490 | 0.89921851 | 0.89750390 | 0.89921851 |
| MOTIVE*TIME*PGQ_ACH_C1 | 0.13410338 | 2 | 0.06705169 | 0.68269491 | 0.50723780 | 0.50588889 | 0.50723780 |
| MOTIVE*TIME*PGQ_AFF_C1 | 1.13617109 | 2 | 0.56808554 | 5.78403193 | 0.00401475 | 0.00413836 | 0.00401475 |
| MOTIVE*TIME*PGQ_AFF_C2 | 0.41904192 | 2 | 0.20952096 | 2.13326310 | 0.12299617 | 0.12353136 | 0.12299617 |
| MOTIVE*TIME*PGQ_IND_C2 | 0.89101058 | 2 | 0.44550529 | 4.53596621 | 0.01265057 | 0.01291916 | 0.01265057 |
| MOTIVE*TIME*PGQ_IND_C1 | 0.59666556 | 2 | 0.29833278 | 3.03751140 | 0.05172029 | 0.05225479 | 0.05172029 |
| MOTIVE*TIME*CAT1 | 2.47096589 | 2 | 1.23548294 | 12.57921958 | 0.00001116 | 0.00001211 | 0.00001116 |
| MOTIVE*TIME*CPT1 | 2.79141848 | 2 | 1.39570924 | 14.21058310 | 0.00000295 | 0.00000324 | 0.00000295 |
| MOTIVE*TIME*CIT1 | 0.57856433 | 2 | 0.28928216 | 2.94536146 | 0.05646119 | 0.05700871 | 0.05646119 |
| MOTIVE*TIME*CAT2 | 0.63722301 | 2 | 0.31861150 | 3.24398170 | 0.04251323 | 0.04301401 | 0.04251323 |
| MOTIVE*TIME*CPT2 | 1.86655736 | 2 | 0.93327868 | 9.50229021 | 0.00014917 | 0.00015816 | 0.00014917 |
| MOTIVE*TIME*CIT2 | 0.23338647 | 2 | 0.11669323 | 1.18812633 | 0.30841115 | 0.30814839 | 0.30841115 |
| Error | 11.58950969 | 118 | 0.09821618 |  |  |  |  |

| Greenhouse-Geisser Epsilon | 0.99029015 |
| --- | --- |
| Huynh-Feldt Epsilon | 1.00000000 |

**Multivariate Repeated Measures Analysis**

| **Test of: MOTIVE** | | | | | |
| --- | --- | --- | --- | --- | --- |
| **Statistic** | **Value** | **Hypothesis df** | **Error df** | **F-Ratio** | **p-Value** |
| Wilks's Lambda | 0.96887178 | 2 | 58 | 0.93172134 | 0.39968959 |
| Pillai Trace | 0.03112822 | 2 | 58 | 0.93172134 | 0.39968959 |
| Hotelling-Lawley Trace | 0.03212832 | 2 | 58 | 0.93172134 | 0.39968959 |

| **Test of: MOTIVE*CONDITION** | | | | | |
| --- | --- | --- | --- | --- | --- |
| **Statistic** | **Value** | **Hypothesis df** | **Error df** | **F-Ratio** | **p-Value** |
| Wilks's Lambda | 0.96324205 | 4 | 116 | 0.54814909 | 0.70072348 |
| Pillai Trace | 0.03676222 | 4 | 118 | 0.55239648 | 0.69763839 |
| Hotelling-Lawley Trace | 0.03815622 | 4 | 114 | 0.54372619 | 0.70393777 |

| **THETA** | **S** | **M** | **N** | **p-Value** |
| --- | --- | --- | --- | --- |
| 0.03664563 | 2 | -0.50000000 | 28.00000000 | 0.62151698 |

| **Test of: MOTIVE*PGQ_ACH_C2** | | | | | |
| --- | --- | --- | --- | --- | --- |
| **Statistic** | **Value** | **Hypothesis df** | **Error df** | **F-Ratio** | **p-Value** |
| Wilks's Lambda | 0.96995659 | 2 | 58 | 0.89824524 | 0.41287315 |
| Pillai Trace | 0.03004341 | 2 | 58 | 0.89824524 | 0.41287315 |
| Hotelling-Lawley Trace | 0.03097397 | 2 | 58 | 0.89824524 | 0.41287315 |

| **Test of: MOTIVE*PGQ_ACH_C1** | | | | | |
| --- | --- | --- | --- | --- | --- |
| **Statistic** | **Value** | **Hypothesis df** | **Error df** | **F-Ratio** | **p-Value** |
| Wilks's Lambda | 0.86812899 | 2 | 58 | 4.40517419 | 0.01655554 |
| Pillai Trace | 0.13187101 | 2 | 58 | 4.40517419 | 0.01655554 |
| Hotelling-Lawley Trace | 0.15190256 | 2 | 58 | 4.40517419 | 0.01655554 |

| **Test of: MOTIVE*PGQ_AFF_C1** | | | | | |
| --- | --- | --- | --- | --- | --- |
| **Statistic** | **Value** | **Hypothesis df** | **Error df** | **F-Ratio** | **p-Value** |
| Wilks's Lambda | 0.98920084 | 2 | 58 | 0.31659448 | 0.72987732 |
| Pillai Trace | 0.01079916 | 2 | 58 | 0.31659448 | 0.72987732 |
| Hotelling-Lawley Trace | 0.01091705 | 2 | 58 | 0.31659448 | 0.72987732 |

| **Test of: MOTIVE*PGQ_AFF_C2** | | | | | |
| --- | --- | --- | --- | --- | --- |
| **Statistic** | **Value** | **Hypothesis df** | **Error df** | **F-Ratio** | **p-Value** |
| Wilks's Lambda | 0.80247842 | 2 | 58 | 7.13804333 | 0.00169265 |
| Pillai Trace | 0.19752158 | 2 | 58 | 7.13804333 | 0.00169265 |
| Hotelling-Lawley Trace | 0.24613943 | 2 | 58 | 7.13804333 | 0.00169265 |

| **Test of: MOTIVE*PGQ_IND_C2** | | | | | |
| --- | --- | --- | --- | --- | --- |
| **Statistic** | **Value** | **Hypothesis df** | **Error df** | **F-Ratio** | **p-Value** |
| Wilks's Lambda | 0.92487647 | 2 | 58 | 2.35553860 | 0.10385520 |
| Pillai Trace | 0.07512353 | 2 | 58 | 2.35553860 | 0.10385520 |
| Hotelling-Lawley Trace | 0.08122547 | 2 | 58 | 2.35553860 | 0.10385520 |

| **Test of: MOTIVE*PGQ_IND_C1** | | | | | |
| --- | --- | --- | --- | --- | --- |
| **Statistic** | **Value** | **Hypothesis df** | **Error df** | **F-Ratio** | **p-Value** |
| Wilks's Lambda | 0.95057477 | 2 | 58 | 1.50785792 | 0.22993347 |
| Pillai Trace | 0.04942523 | 2 | 58 | 1.50785792 | 0.22993347 |
| Hotelling-Lawley Trace | 0.05199510 | 2 | 58 | 1.50785792 | 0.22993347 |

| **Test of: MOTIVE*CAT1** | | | | | |
| --- | --- | --- | --- | --- | --- |
| **Statistic** | **Value** | **Hypothesis df** | **Error df** | **F-Ratio** | **p-Value** |
| Wilks's Lambda | 0.93478663 | 2 | 58 | 2.02312226 | 0.14146934 |
| Pillai Trace | 0.06521337 | 2 | 58 | 2.02312226 | 0.14146934 |
| Hotelling-Lawley Trace | 0.06976284 | 2 | 58 | 2.02312226 | 0.14146934 |

| **Test of: MOTIVE*CPT1** | | | | | |
| --- | --- | --- | --- | --- | --- |
| **Statistic** | **Value** | **Hypothesis df** | **Error df** | **F-Ratio** | **p-Value** |
| Wilks's Lambda | 0.88178556 | 2 | 58 | 3.88781469 | 0.02603313 |
| Pillai Trace | 0.11821444 | 2 | 58 | 3.88781469 | 0.02603313 |
| Hotelling-Lawley Trace | 0.13406258 | 2 | 58 | 3.88781469 | 0.02603313 |

| **Test of: MOTIVE*CIT1** | | | | | |
| --- | --- | --- | --- | --- | --- |
| **Statistic** | **Value** | **Hypothesis df** | **Error df** | **F-Ratio** | **p-Value** |
| Wilks's Lambda | 0.80234752 | 2 | 58 | 7.14393943 | 0.00168466 |
| Pillai Trace | 0.19765248 | 2 | 58 | 7.14393943 | 0.00168466 |
| Hotelling-Lawley Trace | 0.24634274 | 2 | 58 | 7.14393943 | 0.00168466 |

| **Test of: MOTIVE*CAT2** | | | | | |
| --- | --- | --- | --- | --- | --- |
| **Statistic** | **Value** | **Hypothesis df** | **Error df** | **F-Ratio** | **p-Value** |
| Wilks's Lambda | 0.72059429 | 2 | 58 | 11.24456008 | 0.00007465 |
| Pillai Trace | 0.27940571 | 2 | 58 | 11.24456008 | 0.00007465 |
| Hotelling-Lawley Trace | 0.38774345 | 2 | 58 | 11.24456008 | 0.00007465 |

| **Test of: MOTIVE*CPT2** | | | | | |
| --- | --- | --- | --- | --- | --- |
| **Statistic** | **Value** | **Hypothesis df** | **Error df** | **F-Ratio** | **p-Value** |
| Wilks's Lambda | 0.88224171 | 2 | 58 | 3.87081042 | 0.02642652 |
| Pillai Trace | 0.11775829 | 2 | 58 | 3.87081042 | 0.02642652 |
| Hotelling-Lawley Trace | 0.13347622 | 2 | 58 | 3.87081042 | 0.02642652 |

| **Test of: MOTIVE*CIT2** | | | | | |
| --- | --- | --- | --- | --- | --- |
| **Statistic** | **Value** | **Hypothesis df** | **Error df** | **F-Ratio** | **p-Value** |
| Wilks's Lambda | 0.89456160 | 2 | 58 | 3.41811407 | 0.03950911 |
| Pillai Trace | 0.10543840 | 2 | 58 | 3.41811407 | 0.03950911 |
| Hotelling-Lawley Trace | 0.11786600 | 2 | 58 | 3.41811407 | 0.03950911 |

| **Test of: MOTIVE*TIME** | | | | | |
| --- | --- | --- | --- | --- | --- |
| **Statistic** | **Value** | **Hypothesis df** | **Error df** | **F-Ratio** | **p-Value** |
| Wilks's Lambda | 0.99492417 | 2 | 58 | 0.14794991 | 0.86279877 |
| Pillai Trace | 0.00507583 | 2 | 58 | 0.14794991 | 0.86279877 |
| Hotelling-Lawley Trace | 0.00510172 | 2 | 58 | 0.14794991 | 0.86279877 |

| **Test of: MOTIVE*TIME*CONDITION** | | | | | |
| --- | --- | --- | --- | --- | --- |
| **Statistic** | **Value** | **Hypothesis df** | **Error df** | **F-Ratio** | **p-Value** |
| Wilks's Lambda | 0.83193515 | 4 | 116 | 2.79459228 | 0.02936139 |
| Pillai Trace | 0.17352711 | 4 | 118 | 2.80269674 | 0.02891311 |
| Hotelling-Lawley Trace | 0.19545104 | 4 | 114 | 2.78517727 | 0.02987877 |

| **THETA** | **S** | **M** | **N** | **p-Value** |
| --- | --- | --- | --- | --- |
| 0.13221303 | 2 | -0.50000000 | 28.00000000 | 0.05705104 |

| **Test of: MOTIVE*TIME*PGQ_ACH_C2** | | | | | |
| --- | --- | --- | --- | --- | --- |
| **Statistic** | **Value** | **Hypothesis df** | **Error df** | **F-Ratio** | **p-Value** |
| Wilks's Lambda | 0.99612410 | 2 | 58 | 0.11283851 | 0.89349052 |
| Pillai Trace | 0.00387590 | 2 | 58 | 0.11283851 | 0.89349052 |
| Hotelling-Lawley Trace | 0.00389098 | 2 | 58 | 0.11283851 | 0.89349052 |

| **Test of: MOTIVE*TIME*PGQ_ACH_C1** | | | | | |
| --- | --- | --- | --- | --- | --- |
| **Statistic** | **Value** | **Hypothesis df** | **Error df** | **F-Ratio** | **p-Value** |
| Wilks's Lambda | 0.97937519 | 2 | 58 | 0.61071534 | 0.54641657 |
| Pillai Trace | 0.02062481 | 2 | 58 | 0.61071534 | 0.54641657 |
| Hotelling-Lawley Trace | 0.02105915 | 2 | 58 | 0.61071534 | 0.54641657 |

| **Test of: MOTIVE*TIME*PGQ_AFF_C1** | | | | | |
| --- | --- | --- | --- | --- | --- |
| **Statistic** | **Value** | **Hypothesis df** | **Error df** | **F-Ratio** | **p-Value** |
| Wilks's Lambda | 0.83289679 | 2 | 58 | 5.81823977 | 0.00497903 |
| Pillai Trace | 0.16710321 | 2 | 58 | 5.81823977 | 0.00497903 |
| Hotelling-Lawley Trace | 0.20062896 | 2 | 58 | 5.81823977 | 0.00497903 |

| **Test of: MOTIVE*TIME*PGQ_AFF_C2** | | | | | |
| --- | --- | --- | --- | --- | --- |
| **Statistic** | **Value** | **Hypothesis df** | **Error df** | **F-Ratio** | **p-Value** |
| Wilks's Lambda | 0.92570758 | 2 | 58 | 2.32738754 | 0.10659596 |
| Pillai Trace | 0.07429242 | 2 | 58 | 2.32738754 | 0.10659596 |
| Hotelling-Lawley Trace | 0.08025474 | 2 | 58 | 2.32738754 | 0.10659596 |

| **Test of: MOTIVE*TIME*PGQ_IND_C2** | | | | | |
| --- | --- | --- | --- | --- | --- |
| **Statistic** | **Value** | **Hypothesis df** | **Error df** | **F-Ratio** | **p-Value** |
| Wilks's Lambda | 0.85975006 | 2 | 58 | 4.73073326 | 0.01249667 |
| Pillai Trace | 0.14024994 | 2 | 58 | 4.73073326 | 0.01249667 |
| Hotelling-Lawley Trace | 0.16312873 | 2 | 58 | 4.73073326 | 0.01249667 |

| **Test of: MOTIVE*TIME*PGQ_IND_C1** | | | | | |
| --- | --- | --- | --- | --- | --- |
| **Statistic** | **Value** | **Hypothesis df** | **Error df** | **F-Ratio** | **p-Value** |
| Wilks's Lambda | 0.91124662 | 2 | 58 | 2.82453511 | 0.06752148 |
| Pillai Trace | 0.08875338 | 2 | 58 | 2.82453511 | 0.06752148 |
| Hotelling-Lawley Trace | 0.09739776 | 2 | 58 | 2.82453511 | 0.06752148 |

| **Test of: MOTIVE*TIME*CAT1** | | | | | |
| --- | --- | --- | --- | --- | --- |
| **Statistic** | **Value** | **Hypothesis df** | **Error df** | **F-Ratio** | **p-Value** |
| Wilks's Lambda | 0.70061749 | 2 | 58 | 12.39205829 | 0.00003303 |
| Pillai Trace | 0.29938251 | 2 | 58 | 12.39205829 | 0.00003303 |
| Hotelling-Lawley Trace | 0.42731235 | 2 | 58 | 12.39205829 | 0.00003303 |

| **Test of: MOTIVE*TIME*CPT1** | | | | | |
| --- | --- | --- | --- | --- | --- |
| **Statistic** | **Value** | **Hypothesis df** | **Error df** | **F-Ratio** | **p-Value** |
| Wilks's Lambda | 0.65162869 | 2 | 58 | 15.50387210 | 0.00000404 |
| Pillai Trace | 0.34837131 | 2 | 58 | 15.50387210 | 0.00000404 |
| Hotelling-Lawley Trace | 0.53461628 | 2 | 58 | 15.50387210 | 0.00000404 |

| **Test of: MOTIVE*TIME*CIT1** | | | | | |
| --- | --- | --- | --- | --- | --- |
| **Statistic** | **Value** | **Hypothesis df** | **Error df** | **F-Ratio** | **p-Value** |
| Wilks's Lambda | 0.91622336 | 2 | 58 | 2.65167065 | 0.07907504 |
| Pillai Trace | 0.08377664 | 2 | 58 | 2.65167065 | 0.07907504 |
| Hotelling-Lawley Trace | 0.09143692 | 2 | 58 | 2.65167065 | 0.07907504 |

| **Test of: MOTIVE*TIME*CAT2** | | | | | |
| --- | --- | --- | --- | --- | --- |
| **Statistic** | **Value** | **Hypothesis df** | **Error df** | **F-Ratio** | **p-Value** |
| Wilks's Lambda | 0.90259627 | 2 | 58 | 3.12953661 | 0.05120502 |
| Pillai Trace | 0.09740373 | 2 | 58 | 3.12953661 | 0.05120502 |
| Hotelling-Lawley Trace | 0.10791506 | 2 | 58 | 3.12953661 | 0.05120502 |

| **Test of: MOTIVE*TIME*CPT2** | | | | | |
| --- | --- | --- | --- | --- | --- |
| **Statistic** | **Value** | **Hypothesis df** | **Error df** | **F-Ratio** | **p-Value** |
| Wilks's Lambda | 0.74289465 | 2 | 58 | 10.03649060 | 0.00018067 |
| Pillai Trace | 0.25710535 | 2 | 58 | 10.03649060 | 0.00018067 |
| Hotelling-Lawley Trace | 0.34608588 | 2 | 58 | 10.03649060 | 0.00018067 |

| **Test of: MOTIVE*TIME*CIT2** | | | | | |
| --- | --- | --- | --- | --- | --- |
| **Statistic** | **Value** | **Hypothesis df** | **Error df** | **F-Ratio** | **p-Value** |
| Wilks's Lambda | 0.95741018 | 2 | 58 | 1.29004780 | 0.28303695 |
| Pillai Trace | 0.04258982 | 2 | 58 | 1.29004780 | 0.28303695 |
| Hotelling-Lawley Trace | 0.04448441 | 2 | 58 | 1.29004780 | 0.28303695 |

> *calc 1.074653/(1.074653+11.589510)*

[▼CALCULATE](file:///\\Untitled.syo)

0.08485780

> *!! Testing for possible Time 1 effects driving the overall interaction*

> *glm*

> *model iemach1 iemaff1 iemind1 = constant condition /repeat={3} names={motive}*

> *estimate*

[▼General Linear Model](file:///\\Untitled.syo)

Effects coding used for categorical variables in model.

The categorical values encountered during processing are

| **Variables** | **Levels** | | |
| --- | --- | --- | --- |
| CONDITION (3 levels) | Control | Feedback | Feedback + CET |
| TREAT (2 levels) | CG | FB/FB+CET |  |
| HYPOTH1 (2 levels) | 0.00000000 | 1.00000000 |  |

N of Cases Processed: 74

| **Dependent Variable Means** | | |
| --- | --- | --- |
| **IEMACH1** | **T1** | **IEMIND1** |
| 0.40540541 | 0.29729730 | 0.31081081 |

| **Repeated Measures Factors and Levels of Dependent Variables** | | | |
| --- | --- | --- | --- |
| **Within Factor** | **1** | **2** | **3** |
| MOTIVE | 1.00000000 | 2.00000000 | 3.00000000 |

**Univariate and Multivariate Repeated Measures Analysis**

| **Between Subjects** | | | | | |
| --- | --- | --- | --- | --- | --- |
| **Source** | **SS** | **df** | **Mean Squares** | **F-Ratio** | **p-Value** |
| CONDITION | 0.14299269 | 2 | 0.07149634 | 0.22880546 | 0.79606753 |
| Error | 22.18583614 | 71 | 0.31247657 |  |  |

| **Within Subjects** | | | | | | | |
| --- | --- | --- | --- | --- | --- | --- | --- |
| **Source** | **SS** | **df** | **Mean Squares** | **F-Ratio** | **p-Value** | **G-G** | **H-F** |
| MOTIVE | 0.49932614 | 2 | 0.24966307 | 1.34590811 | 0.26360322 | 0.26321832 | 0.26350865 |
| MOTIVE*CONDITION | 0.47912397 | 4 | 0.11978099 | 0.64572710 | 0.63079015 | 0.61853158 | 0.62658171 |
| Error | 26.34069585 | 142 | 0.18549786 |  |  |  |  |

| Greenhouse-Geisser Epsilon | 0.92258985 |
| --- | --- |
| Huynh-Feldt Epsilon | 0.97276876 |

**Multivariate Repeated Measures Analysis**

| **Test of: MOTIVE** | | | | | |
| --- | --- | --- | --- | --- | --- |
| **Statistic** | **Value** | **Hypothesis df** | **Error df** | **F-Ratio** | **p-Value** |
| Wilks's Lambda | 0.95621442 | 2 | 70 | 1.60266892 | 0.20865807 |
| Pillai Trace | 0.04378558 | 2 | 70 | 1.60266892 | 0.20865807 |
| Hotelling-Lawley Trace | 0.04579054 | 2 | 70 | 1.60266892 | 0.20865807 |

| **Test of: MOTIVE*CONDITION** | | | | | |
| --- | --- | --- | --- | --- | --- |
| **Statistic** | **Value** | **Hypothesis df** | **Error df** | **F-Ratio** | **p-Value** |
| Wilks's Lambda | 0.96818235 | 4 | 140 | 0.57045860 | 0.68449898 |
| Pillai Trace | 0.03194941 | 4 | 142 | 0.57630834 | 0.68026886 |
| Hotelling-Lawley Trace | 0.03272720 | 4 | 138 | 0.56454423 | 0.68878169 |

| **THETA** | **S** | **M** | **N** | **p-Value** |
| --- | --- | --- | --- | --- |
| 0.02708491 | 2 | -0.50000000 | 34.00000000 | 0.66243997 |

> *!! Follow-up tests per motive domain*

> *glm*

> *model iemach1 iemach2 = constant condition /repeat={2} names={time}*

> *estimate*

[▼General Linear Model](file:///\\Untitled.syo)

Effects coding used for categorical variables in model.

The categorical values encountered during processing are

| **Variables** | **Levels** | | |
| --- | --- | --- | --- |
| CONDITION (3 levels) | Control | Feedback | Feedback + CET |
| TREAT (2 levels) | CG | FB/FB+CET |  |
| HYPOTH1 (2 levels) | 0.00000000 | 1.00000000 |  |

N of Cases Processed: 74

| **Dependent Variable Means** | |
| --- | --- |
| **IEMACH1** | **IEMACH2** |
| 0.40540541 | 0.39189189 |

| **Repeated Measures Factors and Levels of Dependent Variables** | | |
| --- | --- | --- |
| **Within Factor** | **1** | **2** |
| TIME | 1.00000000 | 2.00000000 |

**Univariate Repeated Measures Analysis**

| **Between Subjects** | | | | | |
| --- | --- | --- | --- | --- | --- |
| **Source** | **SS** | **df** | **Mean Squares** | **F-Ratio** | **p-Value** |
| CONDITION | 0.21730549 | 2 | 0.10865274 | 0.33890700 | 0.71369506 |
| Error | 22.76242424 | 71 | 0.32059752 |  |  |

| **Within Subjects** | | | | | | | |
| --- | --- | --- | --- | --- | --- | --- | --- |
| **Source** | **SS** | **df** | **Mean Squares** | **F-Ratio** | **p-Value** | **G-G** | **H-F** |
| TIME | 0.00182503 | 1 | 0.00182503 | 0.01136857 | 0.91538838 | . | . |
| TIME*CONDITION | 1.09539813 | 2 | 0.54769906 | 3.41175310 | 0.03848028 | . | . |
| Error | 11.39784512 | 71 | 0.16053303 |  |  |  |  |

| Greenhouse-Geisser Epsilon | . |
| --- | --- |
| Huynh-Feldt Epsilon | . |

Since your repeated measure has only two levels, Greenhouse-Geisser and

Huynh-Feldt Epsilon corrections are not necessary.

> *calc 1.095398/(1.095398+11.397845)*

[▼CALCULATE](file:///\\Untitled.syo)

0.08767924

> *glm*

> *model iemind1 iemind2 = constant condition /repeat={2} names={time}*

> *estimate*

[▼General Linear Model](file:///\\Untitled.syo)

Effects coding used for categorical variables in model.

The categorical values encountered during processing are

| **Variables** | **Levels** | | |
| --- | --- | --- | --- |
| CONDITION (3 levels) | Control | Feedback | Feedback + CET |
| TREAT (2 levels) | CG | FB/FB+CET |  |
| HYPOTH1 (2 levels) | 0.00000000 | 1.00000000 |  |

N of Cases Processed: 74

| **Dependent Variable Means** | |
| --- | --- |
| **IEMIND1** | **IEMIND2** |
| 0.31081081 | 0.31081081 |

| **Repeated Measures Factors and Levels of Dependent Variables** | | |
| --- | --- | --- |
| **Within Factor** | **1** | **2** |
| TIME | 1.00000000 | 2.00000000 |

**Univariate Repeated Measures Analysis**

| **Between Subjects** | | | | | |
| --- | --- | --- | --- | --- | --- |
| **Source** | **SS** | **df** | **Mean Squares** | **F-Ratio** | **p-Value** |
| CONDITION | 0.42596870 | 2 | 0.21298435 | 0.87527473 | 0.42119530 |
| Error | 17.27673401 | 71 | 0.24333428 |  |  |

| **Within Subjects** | | | | | | | |
| --- | --- | --- | --- | --- | --- | --- | --- |
| **Source** | **SS** | **df** | **Mean Squares** | **F-Ratio** | **p-Value** | **G-G** | **H-F** |
| TIME | 0.00561971 | 1 | 0.00561971 | 0.03174714 | 0.85909117 | . | . |
| TIME*CONDITION | 1.43195286 | 2 | 0.71597643 | 4.04472756 | 0.02170010 | . | . |
| Error | 12.56804714 | 71 | 0.17701475 |  |  |  |  |

| Greenhouse-Geisser Epsilon | . |
| --- | --- |
| Huynh-Feldt Epsilon | . |

Since your repeated measure has only two levels, Greenhouse-Geisser and

Huynh-Feldt Epsilon corrections are not necessary.

> *calc 1.431953/(1.431953+12.568047)*

[▼CALCULATE](file:///\\Untitled.syo)

0.10228236

> *glm*

> *model iemaff1 iemaff2 = constant condition /repeat={2} names={time}*

> *estimate*

[▼General Linear Model](file:///\\Untitled.syo)

Effects coding used for categorical variables in model.

The categorical values encountered during processing are

| **Variables** | **Levels** | | |
| --- | --- | --- | --- |
| CONDITION (3 levels) | Control | Feedback | Feedback + CET |
| TREAT (2 levels) | CG | FB/FB+CET |  |
| HYPOTH1 (2 levels) | 0.00000000 | 1.00000000 |  |

N of Cases Processed: 74

| **Dependent Variable Means** | |
| --- | --- |
| **T1** | **T2** |
| 0.29729730 | 0.31081081 |

| **Repeated Measures Factors and Levels of Dependent Variables** | | |
| --- | --- | --- |
| **Within Factor** | **1** | **2** |
| TIME | 1.00000000 | 2.00000000 |

**Univariate Repeated Measures Analysis**

| **Between Subjects** | | | | | |
| --- | --- | --- | --- | --- | --- |
| **Source** | **SS** | **df** | **Mean Squares** | **F-Ratio** | **p-Value** |
| CONDITION | 0.09194467 | 2 | 0.04597234 | 0.16547188 | 0.84781958 |
| Error | 19.72562290 | 71 | 0.27782567 |  |  |

| **Within Subjects** | | | | | | | |
| --- | --- | --- | --- | --- | --- | --- | --- |
| **Source** | **SS** | **df** | **Mean Squares** | **F-Ratio** | **p-Value** | **G-G** | **H-F** |
| TIME | 0.00460906 | 1 | 0.00460906 | 0.03013016 | 0.86268917 | . | . |
| TIME*CONDITION | 0.63226681 | 2 | 0.31613341 | 2.06661638 | 0.13416298 | . | . |
| Error | 10.86097643 | 71 | 0.15297150 |  |  |  |  |

| Greenhouse-Geisser Epsilon | . |
| --- | --- |
| Huynh-Feldt Epsilon | . |

Since your repeated measure has only two levels, Greenhouse-Geisser and

Huynh-Feldt Epsilon corrections are not necessary.

> *calc 0.632267/(0.632267+10.860976)*

[▼CALCULATE](file:///\\Untitled.syo)

0.05501206

> *graph off*

> *by condition*

> *testing*

> *ttest iemach2 iemach1*

[▼Hypothesis Testing: Paired t-test](file:///\\Untitled.syo)

**Results for CONDITION = Control**

**H0: Mean Difference = 0 vs. H1: Mean Difference <> 0**

| **Variable** | **N** | **Mean** |
| --- | --- | --- |
| IEMACH2 | 27.00000000 | 0.40740741 |
| IEMACH1 | 27.00000000 | 0.37037037 |

| **Variable** | **Mean Difference** | **95.00% Confidence Interval** | | **Standard Deviation of Difference** | **t** | **df** | **p-Value** |
| --- | --- | --- | --- | --- | --- | --- | --- |
|  |  | **Lower Limit** | **Upper Limit** |  |  |  |  |
| IEMACH2 | 0.03703704 | -0.21983636 | 0.29391043 | 0.64934769 | 0.29637449 | 26.00000000 | 0.76929864 |
| IEMACH1 |  |  |  |  |  |  |  |

**Results for CONDITION = Feedback + CET**

**H0: Mean Difference = 0 vs. H1: Mean Difference <> 0**

| **Variable** | **N** | **Mean** |
| --- | --- | --- |
| IEMACH2 | 22.00000000 | 0.54545455 |
| IEMACH1 | 22.00000000 | 0.36363636 |

| **Variable** | **Mean Difference** | **95.00% Confidence Interval** | | **Standard Deviation of Difference** | **t** | **df** | **p-Value** |
| --- | --- | --- | --- | --- | --- | --- | --- |
|  |  | **Lower Limit** | **Upper Limit** |  |  |  |  |
| IEMACH2 | 0.18181818 | -0.04034873 | 0.40398509 | 0.50108108 | 1.70192589 | 21.00000000 | 0.10352892 |
| IEMACH1 |  |  |  |  |  |  |  |

**Results for CONDITION = Feedback**

**H0: Mean Difference = 0 vs. H1: Mean Difference <> 0**

| **Variable** | **N** | **Mean** |
| --- | --- | --- |
| IEMACH2 | 25.00000000 | 0.24000000 |
| IEMACH1 | 25.00000000 | 0.48000000 |

| **Variable** | **Mean Difference** | **95.00% Confidence Interval** | | **Standard Deviation of Difference** | **t** | **df** | **p-Value** |
| --- | --- | --- | --- | --- | --- | --- | --- |
|  |  | **Lower Limit** | **Upper Limit** |  |  |  |  |
| IEMACH2 | -0.24000000 | -0.45580656 | -0.02419344 | 0.52281290 | -2.29527617 | 24.00000000 | 0.03076031 |
| IEMACH1 |  |  |  |  |  |  |  |

> *ttest iemind2 iemind1*

[▼Hypothesis Testing: Paired t-test](file:///\\Untitled.syo)

**Results for CONDITION = Control**

**H0: Mean Difference = 0 vs. H1: Mean Difference <> 0**

| **Variable** | **N** | **Mean** |
| --- | --- | --- |
| IEMIND2 | 27.00000000 | 0.11111111 |
| IEMIND1 | 27.00000000 | 0.37037037 |

| **Variable** | **Mean Difference** | **95.00% Confidence Interval** | | **Standard Deviation of Difference** | **t** | **df** | **p-Value** |
| --- | --- | --- | --- | --- | --- | --- | --- |
|  |  | **Lower Limit** | **Upper Limit** |  |  |  |  |
| IEMIND2 | -0.25925926 | -0.49438442 | -0.02413410 | 0.59437055 | -2.26651644 | 26.00000000 | 0.03197247 |
| IEMIND1 |  |  |  |  |  |  |  |

**Results for CONDITION = Feedback + CET**

**H0: Mean Difference = 0 vs. H1: Mean Difference <> 0**

| **Variable** | **N** | **Mean** |
| --- | --- | --- |
| IEMIND2 | 22.00000000 | 0.40909091 |
| IEMIND1 | 22.00000000 | 0.27272727 |

| **Variable** | **Mean Difference** | **95.00% Confidence Interval** | | **Standard Deviation of Difference** | **t** | **df** | **p-Value** |
| --- | --- | --- | --- | --- | --- | --- | --- |
|  |  | **Lower Limit** | **Upper Limit** |  |  |  |  |
| IEMIND2 | 0.13636364 | -0.11202652 | 0.38475379 | 0.56022568 | 1.14168659 | 21.00000000 | 0.26643600 |
| IEMIND1 |  |  |  |  |  |  |  |

**Results for CONDITION = Feedback**

**H0: Mean Difference = 0 vs. H1: Mean Difference <> 0**

| **Variable** | **N** | **Mean** |
| --- | --- | --- |
| IEMIND2 | 25.00000000 | 0.44000000 |
| IEMIND1 | 25.00000000 | 0.28000000 |

| **Variable** | **Mean Difference** | **95.00% Confidence Interval** | | **Standard Deviation of Difference** | **t** | **df** | **p-Value** |
| --- | --- | --- | --- | --- | --- | --- | --- |
|  |  | **Lower Limit** | **Upper Limit** |  |  |  |  |
| IEMIND2 | 0.16000000 | -0.09778085 | 0.41778085 | 0.62449980 | 1.28102523 | 24.00000000 | 0.21242542 |
| IEMIND1 |  |  |  |  |  |  |  |

> *ttest iemaff2 iemaff1*

[▼Hypothesis Testing: Paired t-test](file:///\\Untitled.syo)

**Results for CONDITION = Control**

**H0: Mean Difference = 0 vs. H1: Mean Difference <> 0**

| **Variable** | **N** | **Mean** |
| --- | --- | --- |
| T2 | 27.00000000 | 0.37037037 |
| T1 | 27.00000000 | 0.22222222 |

| **Variable** | **Mean Difference** | **95.00% Confidence Interval** | | **Standard Deviation of Difference** | **t** | **df** | **p-Value** |
| --- | --- | --- | --- | --- | --- | --- | --- |
|  |  | **Lower Limit** | **Upper Limit** |  |  |  |  |
| T2 | 0.14814815 | -0.06300048 | 0.35929678 | 0.53376051 | 1.44222051 | 26.00000000 | 0.16117894 |
| T1 |  |  |  |  |  |  |  |

**Results for CONDITION = Feedback + CET**

**H0: Mean Difference = 0 vs. H1: Mean Difference <> 0**

| **Variable** | **N** | **Mean** |
| --- | --- | --- |
| T2 | 22.00000000 | 0.36363636 |
| T1 | 22.00000000 | 0.31818182 |

| **Variable** | **Mean Difference** | **95.00% Confidence Interval** | | **Standard Deviation of Difference** | **t** | **df** | **p-Value** |
| --- | --- | --- | --- | --- | --- | --- | --- |
|  |  | **Lower Limit** | **Upper Limit** |  |  |  |  |
| T2 | 0.04545455 | -0.24406873 | 0.33497782 | 0.65299841 | 0.32649500 | 21.00000000 | 0.74728150 |
| T1 |  |  |  |  |  |  |  |

**Results for CONDITION = Feedback**

**H0: Mean Difference = 0 vs. H1: Mean Difference <> 0**

| **Variable** | **N** | **Mean** |
| --- | --- | --- |
| T2 | 25.00000000 | 0.20000000 |
| T1 | 25.00000000 | 0.36000000 |

| **Variable** | **Mean Difference** | **95.00% Confidence Interval** | | **Standard Deviation of Difference** | **t** | **df** | **p-Value** |
| --- | --- | --- | --- | --- | --- | --- | --- |
|  |  | **Lower Limit** | **Upper Limit** |  |  |  |  |
| T2 | -0.16000000 | -0.35507208 | 0.03507208 | 0.47258156 | -1.69282948 | 24.00000000 | 0.10343122 |
| T1 |  |  |  |  |  |  |  |

> *by*

> *by treat*

> *testing*

> *ttest iemach2 iemach1*

[▼Hypothesis Testing: Paired t-test](file:///\\Untitled.syo)

**Results for TREAT = CG**

**H0: Mean Difference = 0 vs. H1: Mean Difference <> 0**

| **Variable** | **N** | **Mean** |
| --- | --- | --- |
| IEMACH2 | 27.00000000 | 0.40740741 |
| IEMACH1 | 27.00000000 | 0.37037037 |

| **Variable** | **Mean Difference** | **95.00% Confidence Interval** | | **Standard Deviation of Difference** | **t** | **df** | **p-Value** |
| --- | --- | --- | --- | --- | --- | --- | --- |
|  |  | **Lower Limit** | **Upper Limit** |  |  |  |  |
| IEMACH2 | 0.03703704 | -0.21983636 | 0.29391043 | 0.64934769 | 0.29637449 | 26.00000000 | 0.76929864 |
| IEMACH1 |  |  |  |  |  |  |  |

**Results for TREAT = FB/FB+CET**

**H0: Mean Difference = 0 vs. H1: Mean Difference <> 0**

| **Variable** | **N** | **Mean** |
| --- | --- | --- |
| IEMACH2 | 47.00000000 | 0.38297872 |
| IEMACH1 | 47.00000000 | 0.42553191 |

| **Variable** | **Mean Difference** | **95.00% Confidence Interval** | | **Standard Deviation of Difference** | **t** | **df** | **p-Value** |
| --- | --- | --- | --- | --- | --- | --- | --- |
|  |  | **Lower Limit** | **Upper Limit** |  |  |  |  |
| IEMACH2 | -0.04255319 | -0.20403863 | 0.11893224 | 0.54999790 | -0.53042018 | 46.00000000 | 0.59837208 |
| IEMACH1 |  |  |  |  |  |  |  |

> *ttest iemind2 iemind1*

[▼Hypothesis Testing: Paired t-test](file:///\\Untitled.syo)

**Results for TREAT = CG**

**H0: Mean Difference = 0 vs. H1: Mean Difference <> 0**

| **Variable** | **N** | **Mean** |
| --- | --- | --- |
| IEMIND2 | 27.00000000 | 0.11111111 |
| IEMIND1 | 27.00000000 | 0.37037037 |

| **Variable** | **Mean Difference** | **95.00% Confidence Interval** | | **Standard Deviation of Difference** | **t** | **df** | **p-Value** |
| --- | --- | --- | --- | --- | --- | --- | --- |
|  |  | **Lower Limit** | **Upper Limit** |  |  |  |  |
| IEMIND2 | -0.25925926 | -0.49438442 | -0.02413410 | 0.59437055 | -2.26651644 | 26.00000000 | 0.03197247 |
| IEMIND1 |  |  |  |  |  |  |  |

**Results for TREAT = FB/FB+CET**

**H0: Mean Difference = 0 vs. H1: Mean Difference <> 0**

| **Variable** | **N** | **Mean** |
| --- | --- | --- |
| IEMIND2 | 47.00000000 | 0.42553191 |
| IEMIND1 | 47.00000000 | 0.27659574 |

| **Variable** | **Mean Difference** | **95.00% Confidence Interval** | | **Standard Deviation of Difference** | **t** | **df** | **p-Value** |
| --- | --- | --- | --- | --- | --- | --- | --- |
|  |  | **Lower Limit** | **Upper Limit** |  |  |  |  |
| IEMIND2 | 0.14893617 | -0.02399573 | 0.32186807 | 0.58898304 | 1.73358972 | 46.00000000 | 0.08968975 |
| IEMIND1 |  |  |  |  |  |  |  |

> *ttest iemaff2 iemaff1*

[▼Hypothesis Testing: Paired t-test](file:///\\Untitled.syo)

**Results for TREAT = CG**

**H0: Mean Difference = 0 vs. H1: Mean Difference <> 0**

| **Variable** | **N** | **Mean** |
| --- | --- | --- |
| T2 | 27.00000000 | 0.37037037 |
| T1 | 27.00000000 | 0.22222222 |

| **Variable** | **Mean Difference** | **95.00% Confidence Interval** | | **Standard Deviation of Difference** | **t** | **df** | **p-Value** |
| --- | --- | --- | --- | --- | --- | --- | --- |
|  |  | **Lower Limit** | **Upper Limit** |  |  |  |  |
| T2 | 0.14814815 | -0.06300048 | 0.35929678 | 0.53376051 | 1.44222051 | 26.00000000 | 0.16117894 |
| T1 |  |  |  |  |  |  |  |

**Results for TREAT = FB/FB+CET**

**H0: Mean Difference = 0 vs. H1: Mean Difference <> 0**

| **Variable** | **N** | **Mean** |
| --- | --- | --- |
| T2 | 47.00000000 | 0.27659574 |
| T1 | 47.00000000 | 0.34042553 |

| **Variable** | **Mean Difference** | **95.00% Confidence Interval** | | **Standard Deviation of Difference** | **t** | **df** | **p-Value** |
| --- | --- | --- | --- | --- | --- | --- | --- |
|  |  | **Lower Limit** | **Upper Limit** |  |  |  |  |
| T2 | -0.06382979 | -0.23041985 | 0.10276028 | 0.56738360 | -0.77125066 | 46.00000000 | 0.44450218 |
| T1 |  |  |  |  |  |  |  |

> *by*

> *plength long*

> *glm*

> *model iemach1 = constant condition*

> *estimate*

[▼General Linear Model](file:///\\Untitled.syo)

Effects coding used for categorical variables in model.

The categorical values encountered during processing are

| **Variables** | **Levels** | | |
| --- | --- | --- | --- |
| CONDITION (3 levels) | Control | Feedback | Feedback + CET |
| TREAT (2 levels) | CG | FB/FB+CET |  |
| HYPOTH1 (2 levels) | 0.00000000 | 1.00000000 |  |

| Dependent Variable | IEMACH1 |
| --- | --- |
| N | 74 |
| Multiple R | 0.10866546 |
| Squared Multiple R | 0.01180818 |

| **Estimates of Effects B = (X'X)^-1^X'Y** | | |
| --- | --- | --- |
| **Factor** | **Level** | **IEMACH1** |
| CONSTANT |  | 0.40466891 |
| CONDITION | Control | -0.03429854 |
| CONDITION | Feedback | 0.07533109 |

| **Analysis of Variance** | | | | | |
| --- | --- | --- | --- | --- | --- |
| **Source** | **Type III SS** | **df** | **Mean Squares** | **F-Ratio** | **p-Value** |
| CONDITION | 0.21063245 | 2 | 0.10531623 | 0.42419952 | 0.65594057 |
| Error | 17.62720539 | 71 | 0.24827050 |  |  |

| **Least Squares Means** | | | | |
| --- | --- | --- | --- | --- |
| **Factor** | **Level** | **LS Mean** | **Standard Error** | **N** |
| CONDITION | Control | 0.37037037 | 0.09589162 | 27.00000000 |
| CONDITION | Feedback | 0.48000000 | 0.09965350 | 25.00000000 |
| CONDITION | Feedback + CET | 0.36363636 | 0.10623099 | 22.00000000 |

| Durbin-Watson D-Statistic | 1.99168023 |
| --- | --- |
| First Order Autocorrelation | -0.00626645 |

| **Information Criteria** | |
| --- | --- |
| AIC | 111.84090274 |
| AIC (Corrected) | 112.42061288 |
| Schwarz's BIC | 121.05716311 |

> *HYPOTHESIS*

[▼Hypothesis Tests](file:///\\Untitled.syo)

Post Hoc Test of IEMACH1

Using least squares means.

Using model MSE of 0.24827050 with 71 df.

| **Tukey's Honestly-Significant-Difference Test** | | | | | |
| --- | --- | --- | --- | --- | --- |
| **CONDITION(i)** | **CONDITION(j)** | **Difference** | **p-Value** | **95% Confidence Interval** | |
|  |  |  |  | **Lower** | **Upper** |
| Control | Feedback | -0.10962963 | 0.70871640 | -0.44069940 | 0.22144014 |
| Control | Feedback + CET | 0.00673401 | 0.99878787 | -0.33585589 | 0.34932391 |
| Feedback | Feedback + CET | 0.11636364 | 0.70493214 | -0.23232462 | 0.46505189 |

> *post condition /tukey*

> *test*

> *glm*

> *model iemind1 = constant condition*

> *estimate*

[▼General Linear Model](file:///\\Untitled.syo)

Effects coding used for categorical variables in model.

The categorical values encountered during processing are

| **Variables** | **Levels** | | |
| --- | --- | --- | --- |
| CONDITION (3 levels) | Control | Feedback | Feedback + CET |
| TREAT (2 levels) | CG | FB/FB+CET |  |
| HYPOTH1 (2 levels) | 0.00000000 | 1.00000000 |  |

| Dependent Variable | IEMIND1 |
| --- | --- |
| N | 74 |
| Multiple R | 0.09773646 |
| Squared Multiple R | 0.00955242 |

| **Estimates of Effects B = (X'X)^-1^X'Y** | | |
| --- | --- | --- |
| **Factor** | **Level** | **IEMIND1** |
| CONSTANT |  | 0.30769921 |
| CONDITION | Control | 0.06267116 |
| CONDITION | Feedback | -0.02769921 |

| **Analysis of Variance** | | | | | |
| --- | --- | --- | --- | --- | --- |
| **Source** | **Type III SS** | **df** | **Mean Squares** | **F-Ratio** | **p-Value** |
| CONDITION | 0.15141869 | 2 | 0.07570935 | 0.34238131 | 0.71124324 |
| Error | 15.69993266 | 71 | 0.22112581 |  |  |

| **Least Squares Means** | | | | |
| --- | --- | --- | --- | --- |
| **Factor** | **Level** | **LS Mean** | **Standard Error** | **N** |
| CONDITION | Control | 0.37037037 | 0.09049776 | 27.00000000 |
| CONDITION | Feedback | 0.28000000 | 0.09404803 | 25.00000000 |
| CONDITION | Feedback + CET | 0.27272727 | 0.10025554 | 22.00000000 |

| Durbin-Watson D-Statistic | 2.40092429 |
| --- | --- |
| First Order Autocorrelation | -0.21558430 |

| **Information Criteria** | |
| --- | --- |
| AIC | 103.27266133 |
| AIC (Corrected) | 103.85237148 |
| Schwarz's BIC | 112.48892170 |

> *HYPOTHESIS*

[▼Hypothesis Tests](file:///\\Untitled.syo)

Post Hoc Test of IEMIND1

Using least squares means.

Using model MSE of 0.22112581 with 71 df.

| **Tukey's Honestly-Significant-Difference Test** | | | | | |
| --- | --- | --- | --- | --- | --- |
| **CONDITION(i)** | **CONDITION(j)** | **Difference** | **p-Value** | **95% Confidence Interval** | |
|  |  |  |  | **Lower** | **Upper** |
| Control | Feedback | 0.09037037 | 0.76869847 | -0.22207686 | 0.40281760 |
| Control | Feedback + CET | 0.09764310 | 0.75075721 | -0.22567627 | 0.42096246 |
| Feedback | Feedback + CET | 0.00727273 | 0.99846635 | -0.32180196 | 0.33634742 |

> *post condition /tukey*

> *test*

> *glm*

> *model iemaff1 = constant condition*

> *estimate*

[▼General Linear Model](file:///\\Untitled.syo)

Effects coding used for categorical variables in model.

The categorical values encountered during processing are

| **Variables** | **Levels** | | |
| --- | --- | --- | --- |
| CONDITION (3 levels) | Control | Feedback | Feedback + CET |
| TREAT (2 levels) | CG | FB/FB+CET |  |
| HYPOTH1 (2 levels) | 0.00000000 | 1.00000000 |  |

| Dependent Variable | T1 |
| --- | --- |
| N | 74 |
| Multiple R | 0.12970127 |
| Squared Multiple R | 0.01682242 |

| **Estimates of Effects B = (X'X)^-1^X'Y** | | |
| --- | --- | --- |
| **Factor** | **Level** | **T1** |
| CONSTANT |  | 0.30013468 |
| CONDITION | Control | -0.07791246 |
| CONDITION | Feedback | 0.05986532 |

| **Analysis of Variance** | | | | | |
| --- | --- | --- | --- | --- | --- |
| **Source** | **Type III SS** | **df** | **Mean Squares** | **F-Ratio** | **p-Value** |
| CONDITION | 0.26006552 | 2 | 0.13003276 | 0.60741409 | 0.54756390 |
| Error | 15.19939394 | 71 | 0.21407597 |  |  |

| **Least Squares Means** | | | | |
| --- | --- | --- | --- | --- |
| **Factor** | **Level** | **LS Mean** | **Standard Error** | **N** |
| CONDITION | Control | 0.22222222 | 0.08904347 | 27.00000000 |
| CONDITION | Feedback | 0.36000000 | 0.09253669 | 25.00000000 |
| CONDITION | Feedback + CET | 0.31818182 | 0.09864444 | 22.00000000 |

| Durbin-Watson D-Statistic | 1.28720728 |
| --- | --- |
| First Order Autocorrelation | 0.35050854 |

| **Information Criteria** | |
| --- | --- |
| AIC | 100.87499706 |
| AIC (Corrected) | 101.45470721 |
| Schwarz's BIC | 110.09125743 |

> *HYPOTHESIS*

[▼Hypothesis Tests](file:///\\Untitled.syo)

Post Hoc Test of T1

Using least squares means.

Using model MSE of 0.21407597 with 71 df.

| **Tukey's Honestly-Significant-Difference Test** | | | | | |
| --- | --- | --- | --- | --- | --- |
| **CONDITION(i)** | **CONDITION(j)** | **Difference** | **p-Value** | **95% Confidence Interval** | |
|  |  |  |  | **Lower** | **Upper** |
| Control | Feedback | -0.13777778 | 0.53393981 | -0.44520401 | 0.16964846 |
| Control | Feedback + CET | -0.09595960 | 0.75126704 | -0.41408325 | 0.22216405 |
| Feedback | Feedback + CET | 0.04181818 | 0.94871752 | -0.28196831 | 0.36560467 |

> *post condition /tukey*

> *test*

> *glm*

> *model iemach2 = constant condition*

> *estimate*

[▼General Linear Model](file:///\\Untitled.syo)

Effects coding used for categorical variables in model.

The categorical values encountered during processing are

| **Variables** | **Levels** | | |
| --- | --- | --- | --- |
| CONDITION (3 levels) | Control | Feedback | Feedback + CET |
| TREAT (2 levels) | CG | FB/FB+CET |  |
| HYPOTH1 (2 levels) | 0.00000000 | 1.00000000 |  |

| Dependent Variable | IEMACH2 |
| --- | --- |
| N | 74 |
| Multiple R | 0.24998585 |
| Squared Multiple R | 0.06249292 |

| **Estimates of Effects B = (X'X)^-1^X'Y** | | |
| --- | --- | --- |
| **Factor** | **Level** | **IEMACH2** |
| CONSTANT |  | 0.39762065 |
| CONDITION | Control | 0.00978676 |
| CONDITION | Feedback | -0.15762065 |

| **Analysis of Variance** | | | | | |
| --- | --- | --- | --- | --- | --- |
| **Source** | **Type III SS** | **df** | **Mean Squares** | **F-Ratio** | **p-Value** |
| CONDITION | 1.10207116 | 2 | 0.55103558 | 2.36638087 | 0.10118047 |
| Error | 16.53306397 | 71 | 0.23286006 |  |  |

| **Least Squares Means** | | | | |
| --- | --- | --- | --- | --- |
| **Factor** | **Level** | **LS Mean** | **Standard Error** | **N** |
| CONDITION | Control | 0.40740741 | 0.09286790 | 27.00000000 |
| CONDITION | Feedback | 0.24000000 | 0.09651115 | 25.00000000 |
| CONDITION | Feedback + CET | 0.54545455 | 0.10288123 | 22.00000000 |

| Durbin-Watson D-Statistic | 1.91251503 |
| --- | --- |
| First Order Autocorrelation | 0.03698086 |

| **Information Criteria** | |
| --- | --- |
| AIC | 107.09889274 |
| AIC (Corrected) | 107.67860288 |
| Schwarz's BIC | 116.31515311 |

> *HYPOTHESIS*

[▼Hypothesis Tests](file:///\\Untitled.syo)

Post Hoc Test of IEMACH2

Using least squares means.

Using model MSE of 0.23286006 with 71 df.

| **Tukey's Honestly-Significant-Difference Test** | | | | | |
| --- | --- | --- | --- | --- | --- |
| **CONDITION(i)** | **CONDITION(j)** | **Difference** | **p-Value** | **95% Confidence Interval** | |
|  |  |  |  | **Lower** | **Upper** |
| Control | Feedback | 0.16740741 | 0.42814736 | -0.15322282 | 0.48803763 |
| Control | Feedback + CET | -0.13804714 | 0.58178585 | -0.46983424 | 0.19373996 |
| Feedback | Feedback + CET | -0.30545455 | 0.08427866 | -0.64314770 | 0.03223861 |

> *post condition /tukey*

> *test*

> *calc 1.102071/(1.102071+16.533064)*

[▼CALCULATE](file:///\\Untitled.syo)

0.06249292

> *glm*

> *model iemind2 = constant condition*

> *estimate*

[▼General Linear Model](file:///\\Untitled.syo)

Effects coding used for categorical variables in model.

The categorical values encountered during processing are

| **Variables** | **Levels** | | |
| --- | --- | --- | --- |
| CONDITION (3 levels) | Control | Feedback | Feedback + CET |
| TREAT (2 levels) | CG | FB/FB+CET |  |
| HYPOTH1 (2 levels) | 0.00000000 | 1.00000000 |  |

| Dependent Variable | IEMIND2 |
| --- | --- |
| N | 74 |
| Multiple R | 0.32811068 |
| Squared Multiple R | 0.10765662 |

| **Estimates of Effects B = (X'X)^-1^X'Y** | | |
| --- | --- | --- |
| **Factor** | **Level** | **IEMIND2** |
| CONSTANT |  | 0.32006734 |
| CONDITION | Control | -0.20895623 |
| CONDITION | Feedback | 0.11993266 |

| **Analysis of Variance** | | | | | |
| --- | --- | --- | --- | --- | --- |
| **Source** | **Type III SS** | **df** | **Mean Squares** | **F-Ratio** | **p-Value** |
| CONDITION | 1.70650287 | 2 | 0.85325143 | 4.28289153 | 0.01753421 |
| Error | 14.14484848 | 71 | 0.19922322 |  |  |

| **Least Squares Means** | | | | |
| --- | --- | --- | --- | --- |
| **Factor** | **Level** | **LS Mean** | **Standard Error** | **N** |
| CONDITION | Control | 0.11111111 | 0.08589900 | 27.00000000 |
| CONDITION | Feedback | 0.44000000 | 0.08926886 | 25.00000000 |
| CONDITION | Feedback + CET | 0.40909091 | 0.09516092 | 22.00000000 |

| Durbin-Watson D-Statistic | 1.79001605 |
| --- | --- |
| First Order Autocorrelation | 0.09771209 |

| **Information Criteria** | |
| --- | --- |
| AIC | 95.55402251 |
| AIC (Corrected) | 96.13373265 |
| Schwarz's BIC | 104.77028288 |

> *HYPOTHESIS*

[▼Hypothesis Tests](file:///\\Untitled.syo)

Post Hoc Test of IEMIND2

Using least squares means.

Using model MSE of 0.19922322 with 71 df.

| **Tukey's Honestly-Significant-Difference Test** | | | | | |
| --- | --- | --- | --- | --- | --- |
| **CONDITION(i)** | **CONDITION(j)** | **Difference** | **p-Value** | **95% Confidence Interval** | |
|  |  |  |  | **Lower** | **Upper** |
| Control | Feedback | -0.32888889 | 0.02617976 | -0.62545870 | -0.03231908 |
| Control | Feedback + CET | -0.29797980 | 0.05887093 | -0.60486926 | 0.00890966 |
| Feedback | Feedback + CET | 0.03090909 | 0.96955962 | -0.28144323 | 0.34326141 |

> *post condition /tukey*

> *test*

> *calc 1.706503/(1.706503+14.144848)*

[▼CALCULATE](file:///\\Untitled.syo)

0.10765663

> *glm*

> *model iemaff2 = constant condition*

> *estimate*

[▼General Linear Model](file:///\\Untitled.syo)

Effects coding used for categorical variables in model.

The categorical values encountered during processing are

| **Variables** | **Levels** | | |
| --- | --- | --- | --- |
| CONDITION (3 levels) | Control | Feedback | Feedback + CET |
| TREAT (2 levels) | CG | FB/FB+CET |  |
| HYPOTH1 (2 levels) | 0.00000000 | 1.00000000 |  |

| Dependent Variable | T2 |
| --- | --- |
| N | 74 |
| Multiple R | 0.17111739 |
| Squared Multiple R | 0.02928116 |

| **Estimates of Effects B = (X'X)^-1^X'Y** | | |
| --- | --- | --- |
| **Factor** | **Level** | **T2** |
| CONSTANT |  | 0.31133558 |
| CONDITION | Control | 0.05903479 |
| CONDITION | Feedback | -0.11133558 |

| **Analysis of Variance** | | | | | |
| --- | --- | --- | --- | --- | --- |
| **Source** | **Type III SS** | **df** | **Mean Squares** | **F-Ratio** | **p-Value** |
| CONDITION | 0.46414596 | 2 | 0.23207298 | 1.07083654 | 0.34819119 |
| Error | 15.38720539 | 71 | 0.21672120 |  |  |

| **Least Squares Means** | | | | |
| --- | --- | --- | --- | --- |
| **Factor** | **Level** | **LS Mean** | **Standard Error** | **N** |
| CONDITION | Control | 0.37037037 | 0.08959191 | 27.00000000 |
| CONDITION | Feedback | 0.20000000 | 0.09310665 | 25.00000000 |
| CONDITION | Feedback + CET | 0.36363636 | 0.09925202 | 22.00000000 |

| Durbin-Watson D-Statistic | 1.77576273 |
| --- | --- |
| First Order Autocorrelation | 0.10636144 |

| **Information Criteria** | |
| --- | --- |
| AIC | 101.78377555 |
| AIC (Corrected) | 102.36348570 |
| Schwarz's BIC | 111.00003593 |

> *HYPOTHESIS*

[▼Hypothesis Tests](file:///\\Untitled.syo)

Post Hoc Test of T2

Using least squares means.

Using model MSE of 0.21672120 with 71 df.

| **Tukey's Honestly-Significant-Difference Test** | | | | | |
| --- | --- | --- | --- | --- | --- |
| **CONDITION(i)** | **CONDITION(j)** | **Difference** | **p-Value** | **95% Confidence Interval** | |
|  |  |  |  | **Lower** | **Upper** |
| Control | Feedback | 0.17037037 | 0.38968235 | -0.13894939 | 0.47969013 |
| Control | Feedback + CET | 0.00673401 | 0.99861082 | -0.31334906 | 0.32681707 |
| Feedback | Feedback + CET | -0.16363636 | 0.45568606 | -0.48941715 | 0.16214442 |

> *post condition /tukey*

> *test*

> *!! Testing incongruence scores*

> *plength short*

> *glm*

> *model inkon_ach1 inkon_ach2 inkon_aff1 inkon_aff2 inkon_ind1 inkon_ind2 = constant condition /repeat={3, 2} names={motive, time}*

> *estimate*

[▼General Linear Model](file:///\\Untitled.syo)

Effects coding used for categorical variables in model.

The categorical values encountered during processing are

| **Variables** | **Levels** | | |
| --- | --- | --- | --- |
| CONDITION (3 levels) | Control | Feedback | Feedback + CET |
| TREAT (2 levels) | CG | FB/FB+CET |  |
| HYPOTH1 (2 levels) | 0.00000000 | 1.00000000 |  |

N of Cases Processed: 74

| **Dependent Variable Means** | | | | | |
| --- | --- | --- | --- | --- | --- |
| **INKON_ACH1** | **INKON_ACH2** | **T1** | **T2** | **INKON_IND1** | **INKON_IND2** |
| 0.33877977 | 0.26605341 | 0.33486522 | 0.39177101 | 0.44518095 | 0.35487061 |

| **Repeated Measures Factors and Levels of Dependent Variables** | | | | | | |
| --- | --- | --- | --- | --- | --- | --- |
| **Within Factor** | **1** | **2** | **3** | **4** | **5** | **6** |
| MOTIVE | 1.00000000 | 1.00000000 | 2.00000000 | 2.00000000 | 3.00000000 | 3.00000000 |
| TIME | 1.00000000 | 2.00000000 | 1.00000000 | 2.00000000 | 1.00000000 | 2.00000000 |

**Univariate and Multivariate Repeated Measures Analysis**

| **Between Subjects** | | | | | |
| --- | --- | --- | --- | --- | --- |
| **Source** | **SS** | **df** | **Mean Squares** | **F-Ratio** | **p-Value** |
| CONDITION | 1.29066688 | 2 | 0.64533344 | 1.92889019 | 0.15284802 |
| Error | 23.75390497 | 71 | 0.33456204 |  |  |

| **Within Subjects** | | | | | | | |
| --- | --- | --- | --- | --- | --- | --- | --- |
| **Source** | **SS** | **df** | **Mean Squares** | **F-Ratio** | **p-Value** | **G-G** | **H-F** |
| MOTIVE | 0.72683947 | 2 | 0.36341974 | 1.34550028 | 0.26370875 | 0.26362212 | 0.26370875 |
| MOTIVE*CONDITION | 0.52676128 | 4 | 0.13169032 | 0.48756120 | 0.74485355 | 0.74020883 | 0.74485355 |
| Error | 38.35421167 | 142 | 0.27010008 |  |  |  |  |

| Greenhouse-Geisser Epsilon | 0.97500552 |
| --- | --- |
| Huynh-Feldt Epsilon | 1.00000000 |

| **Within Subjects** | | | | | | | |
| --- | --- | --- | --- | --- | --- | --- | --- |
| **Source** | **SS** | **df** | **Mean Squares** | **F-Ratio** | **p-Value** | **G-G** | **H-F** |
| TIME | 0.14726319 | 1 | 0.14726319 | 0.62617566 | 0.43139719 | . | . |
| TIME*CONDITION | 0.29407753 | 2 | 0.14703876 | 0.62522140 | 0.53806304 | . | . |
| Error | 16.69768852 | 71 | 0.23517871 |  |  |  |  |

| Greenhouse-Geisser Epsilon | . |
| --- | --- |
| Huynh-Feldt Epsilon | . |

| **Within Subjects** | | | | | | | |
| --- | --- | --- | --- | --- | --- | --- | --- |
| **Source** | **SS** | **df** | **Mean Squares** | **F-Ratio** | **p-Value** | **G-G** | **H-F** |
| MOTIVE*TIME | 0.56057655 | 2 | 0.28028827 | 1.51682238 | 0.22294124 | 0.22315030 | 0.22294124 |
| MOTIVE*TIME*CONDITION | 1.29245134 | 4 | 0.32311284 | 1.74857397 | 0.14263120 | 0.14348159 | 0.14263120 |
| Error | 26.23968069 | 142 | 0.18478648 |  |  |  |  |

| Greenhouse-Geisser Epsilon | 0.98813831 |
| --- | --- |
| Huynh-Feldt Epsilon | 1.00000000 |

**Multivariate Repeated Measures Analysis**

| **Test of: MOTIVE** | | | | | |
| --- | --- | --- | --- | --- | --- |
| **Statistic** | **Value** | **Hypothesis df** | **Error df** | **F-Ratio** | **p-Value** |
| Wilks's Lambda | 0.96059497 | 2 | 70 | 1.43575185 | 0.24485603 |
| Pillai Trace | 0.03940503 | 2 | 70 | 1.43575185 | 0.24485603 |
| Hotelling-Lawley Trace | 0.04102148 | 2 | 70 | 1.43575185 | 0.24485603 |

| **Test of: MOTIVE*CONDITION** | | | | | |
| --- | --- | --- | --- | --- | --- |
| **Statistic** | **Value** | **Hypothesis df** | **Error df** | **F-Ratio** | **p-Value** |
| Wilks's Lambda | 0.97648428 | 4 | 140 | 0.41892830 | 0.79478099 |
| Pillai Trace | 0.02352320 | 4 | 142 | 0.42250620 | 0.79220205 |
| Hotelling-Lawley Trace | 0.02407436 | 4 | 138 | 0.41528276 | 0.79740492 |

| **THETA** | **S** | **M** | **N** | **p-Value** |
| --- | --- | --- | --- | --- |
| 0.02320067 | 2 | -0.50000000 | 34.00000000 | 0.70485139 |

| **Test of: MOTIVE*TIME** | | | | | |
| --- | --- | --- | --- | --- | --- |
| **Statistic** | **Value** | **Hypothesis df** | **Error df** | **F-Ratio** | **p-Value** |
| Wilks's Lambda | 0.95578951 | 2 | 70 | 1.61894120 | 0.20543723 |
| Pillai Trace | 0.04421049 | 2 | 70 | 1.61894120 | 0.20543723 |
| Hotelling-Lawley Trace | 0.04625546 | 2 | 70 | 1.61894120 | 0.20543723 |

| **Test of: MOTIVE*TIME*CONDITION** | | | | | |
| --- | --- | --- | --- | --- | --- |
| **Statistic** | **Value** | **Hypothesis df** | **Error df** | **F-Ratio** | **p-Value** |
| Wilks's Lambda | 0.90471856 | 4 | 140 | 1.79690515 | 0.13280949 |
| Pillai Trace | 0.09660756 | 4 | 142 | 1.80181885 | 0.13174795 |
| Hotelling-Lawley Trace | 0.10385032 | 4 | 138 | 1.79141798 | 0.13399494 |

| **THETA** | **S** | **M** | **N** | **p-Value** |
| --- | --- | --- | --- | --- |
| 0.08003910 | 2 | -0.50000000 | 34.00000000 | 0.16059759 |

> *calc 0.294078/(0.294078 + 16.697689)*

[▼CALCULATE](file:///\\Untitled.syo)

0.01730709

> *calc 1.292451/(1.292451 + 26.239681)*

[▼CALCULATE](file:///\\Untitled.syo)

0.04694337

> *glm*

> *model inkon_ach1 inkon_ach2 inkon_aff1 inkon_aff2 inkon_ind1 inkon_ind2 = constant treat /repeat={3, 2} names={motive, time}*

> *estimate*

[▼General Linear Model](file:///\\Untitled.syo)

Effects coding used for categorical variables in model.

The categorical values encountered during processing are

| **Variables** | **Levels** | | |
| --- | --- | --- | --- |
| CONDITION (3 levels) | Control | Feedback | Feedback + CET |
| TREAT (2 levels) | CG | FB/FB+CET |  |
| HYPOTH1 (2 levels) | 0.00000000 | 1.00000000 |  |

N of Cases Processed: 74

| **Dependent Variable Means** | | | | | |
| --- | --- | --- | --- | --- | --- |
| **INKON_ACH1** | **INKON_ACH2** | **T1** | **T2** | **INKON_IND1** | **INKON_IND2** |
| 0.33877977 | 0.26605341 | 0.33486522 | 0.39177101 | 0.44518095 | 0.35487061 |

| **Repeated Measures Factors and Levels of Dependent Variables** | | | | | | |
| --- | --- | --- | --- | --- | --- | --- |
| **Within Factor** | **1** | **2** | **3** | **4** | **5** | **6** |
| MOTIVE | 1.00000000 | 1.00000000 | 2.00000000 | 2.00000000 | 3.00000000 | 3.00000000 |
| TIME | 1.00000000 | 2.00000000 | 1.00000000 | 2.00000000 | 1.00000000 | 2.00000000 |

**Univariate and Multivariate Repeated Measures Analysis**

| **Between Subjects** | | | | | |
| --- | --- | --- | --- | --- | --- |
| **Source** | **SS** | **df** | **Mean Squares** | **F-Ratio** | **p-Value** |
| TREAT | 1.15723534 | 1 | 1.15723534 | 3.48808016 | 0.06588229 |
| Error | 23.88733651 | 72 | 0.33176856 |  |  |

| **Within Subjects** | | | | | | | |
| --- | --- | --- | --- | --- | --- | --- | --- |
| **Source** | **SS** | **df** | **Mean Squares** | **F-Ratio** | **p-Value** | **G-G** | **H-F** |
| MOTIVE | 0.57412352 | 2 | 0.28706176 | 1.07000137 | 0.34571914 | 0.34440241 | 0.34571914 |
| MOTIVE*TREAT | 0.24841163 | 2 | 0.12420582 | 0.46296794 | 0.63034637 | 0.62516182 | 0.63034637 |
| Error | 38.63256131 | 144 | 0.26828168 |  |  |  |  |

| Greenhouse-Geisser Epsilon | 0.97346794 |
| --- | --- |
| Huynh-Feldt Epsilon | 1.00000000 |

| **Within Subjects** | | | | | | | |
| --- | --- | --- | --- | --- | --- | --- | --- |
| **Source** | **SS** | **df** | **Mean Squares** | **F-Ratio** | **p-Value** | **G-G** | **H-F** |
| TIME | 0.17424624 | 1 | 0.17424624 | 0.74038870 | 0.39239264 | . | . |
| TIME*TREAT | 0.04697815 | 1 | 0.04697815 | 0.19961460 | 0.65637314 | . | . |
| Error | 16.94478790 | 72 | 0.23534428 |  |  |  |  |

| Greenhouse-Geisser Epsilon | . |
| --- | --- |
| Huynh-Feldt Epsilon | . |

| **Within Subjects** | | | | | | | |
| --- | --- | --- | --- | --- | --- | --- | --- |
| **Source** | **SS** | **df** | **Mean Squares** | **F-Ratio** | **p-Value** | **G-G** | **H-F** |
| MOTIVE*TIME | 0.28773519 | 2 | 0.14386759 | 0.78210477 | 0.45937544 | 0.45782860 | 0.45937544 |
| MOTIVE*TIME*TREAT | 1.04343840 | 2 | 0.52171920 | 2.83621254 | 0.06192844 | 0.06270722 | 0.06192844 |
| Error | 26.48869362 | 144 | 0.18394926 |  |  |  |  |

| Greenhouse-Geisser Epsilon | 0.98644287 |
| --- | --- |
| Huynh-Feldt Epsilon | 1.00000000 |

**Multivariate Repeated Measures Analysis**

| **Test of: MOTIVE** | | | | | |
| --- | --- | --- | --- | --- | --- |
| **Statistic** | **Value** | **Hypothesis df** | **Error df** | **F-Ratio** | **p-Value** |
| Wilks's Lambda | 0.96736304 | 2 | 71 | 1.19770140 | 0.30791221 |
| Pillai Trace | 0.03263696 | 2 | 71 | 1.19770140 | 0.30791221 |
| Hotelling-Lawley Trace | 0.03373807 | 2 | 71 | 1.19770140 | 0.30791221 |

| **Test of: MOTIVE*TREAT** | | | | | |
| --- | --- | --- | --- | --- | --- |
| **Statistic** | **Value** | **Hypothesis df** | **Error df** | **F-Ratio** | **p-Value** |
| Wilks's Lambda | 0.98906191 | 2 | 71 | 0.39259628 | 0.67675816 |
| Pillai Trace | 0.01093809 | 2 | 71 | 0.39259628 | 0.67675816 |
| Hotelling-Lawley Trace | 0.01105905 | 2 | 71 | 0.39259628 | 0.67675816 |

| **Test of: MOTIVE*TIME** | | | | | |
| --- | --- | --- | --- | --- | --- |
| **Statistic** | **Value** | **Hypothesis df** | **Error df** | **F-Ratio** | **p-Value** |
| Wilks's Lambda | 0.97905521 | 2 | 71 | 0.75944635 | 0.47168824 |
| Pillai Trace | 0.02094479 | 2 | 71 | 0.75944635 | 0.47168824 |
| Hotelling-Lawley Trace | 0.02139285 | 2 | 71 | 0.75944635 | 0.47168824 |

| **Test of: MOTIVE*TIME*TREAT** | | | | | |
| --- | --- | --- | --- | --- | --- |
| **Statistic** | **Value** | **Hypothesis df** | **Error df** | **F-Ratio** | **p-Value** |
| Wilks's Lambda | 0.92034835 | 2 | 71 | 3.07235130 | 0.05251756 |
| Pillai Trace | 0.07965165 | 2 | 71 | 3.07235130 | 0.05251756 |
| Hotelling-Lawley Trace | 0.08654511 | 2 | 71 | 3.07235130 | 0.05251756 |

> *calc 1.043438 / (1.043438 + 26.488694)*

[▼CALCULATE](file:///\\Untitled.syo)

0.03789892

> *!! Destructive testing*

> *glm*

> *model inkon_ach1 inkon_ach2 inkon_ind1 inkon_ind2 = constant treat PGQ_ACH_C2 PGQ_ach_C1 PGQ_AFF_C1 PGQ_AFF_C2 PGQ_IND_C2 PGQ_IND_C1 zpowt1 zpowt2 zacht1 zacht2 zafft1
zafft2/repeat={2, 2} names={motive, time}*

> *estimate*

[▼General Linear Model](file:///\\Untitled.syo)

Effects coding used for categorical variables in model.

The categorical values encountered during processing are

| **Variables** | **Levels** | | |
| --- | --- | --- | --- |
| CONDITION (3 levels) | Control | Feedback | Feedback + CET |
| TREAT (2 levels) | CG | FB/FB+CET |  |
| HYPOTH1 (2 levels) | 0.00000000 | 1.00000000 |  |

N of Cases Processed: 74

| **Dependent Variable Means** | | | |
| --- | --- | --- | --- |
| **INKON_ACH1** | **INKON_ACH2** | **INKON_IND1** | **INKON_IND2** |
| 0.33877977 | 0.26605341 | 0.44518095 | 0.35487061 |

| **Repeated Measures Factors and Levels of Dependent Variables** | | | | |
| --- | --- | --- | --- | --- |
| **Within Factor** | **1** | **2** | **3** | **4** |
| MOTIVE | 1.00000000 | 1.00000000 | 2.00000000 | 2.00000000 |
| TIME | 1.00000000 | 2.00000000 | 1.00000000 | 2.00000000 |

**Univariate and Multivariate Repeated Measures Analysis**

| **Between Subjects** | | | | | |
| --- | --- | --- | --- | --- | --- |
| **Source** | **SS** | **df** | **Mean Squares** | **F-Ratio** | **p-Value** |
| TREAT | 0.17479584 | 1 | 0.17479584 | 0.53525959 | 0.46725191 |
| PGQ_ACH_C2 | 0.27191186 | 1 | 0.27191186 | 0.83264815 | 0.36515989 |
| PGQ_ACH_C1 | 0.06934740 | 1 | 0.06934740 | 0.21235552 | 0.64659342 |
| PGQ_AFF_C1 | 0.11588574 | 1 | 0.11588574 | 0.35486515 | 0.55361247 |
| PGQ_AFF_C2 | 0.07431737 | 1 | 0.07431737 | 0.22757457 | 0.63506115 |
| PGQ_IND_C2 | 0.01943291 | 1 | 0.01943291 | 0.05950744 | 0.80810836 |
| PGQ_IND_C1 | 0.00715824 | 1 | 0.00715824 | 0.02191994 | 0.88279671 |
| ZPOWT1 | 0.22110984 | 1 | 0.22110984 | 0.67708225 | 0.41385168 |
| ZPOWT2 | 0.34304134 | 1 | 0.34304134 | 1.05046072 | 0.30951538 |
| ZACHT1 | 0.01742871 | 1 | 0.01742871 | 0.05337018 | 0.81808534 |
| ZACHT2 | 0.19762122 | 1 | 0.19762122 | 0.60515543 | 0.43967455 |
| ZAFFT1 | 0.00813847 | 1 | 0.00813847 | 0.02492160 | 0.87509268 |
| ZAFFT2 | 0.03932925 | 1 | 0.03932925 | 0.12043398 | 0.72977858 |
| Error | 19.59376464 | 60 | 0.32656274 |  |  |

| **Within Subjects** | | | | | | | |
| --- | --- | --- | --- | --- | --- | --- | --- |
| **Source** | **SS** | **df** | **Mean Squares** | **F-Ratio** | **p-Value** | **G-G** | **H-F** |
| MOTIVE | 0.00010394 | 1 | 0.00010394 | 0.00038187 | 0.98447389 | . | . |
| MOTIVE*TREAT | 0.13870896 | 1 | 0.13870896 | 0.50963705 | 0.47806398 | . | . |
| MOTIVE*PGQ_ACH_C2 | 0.26104664 | 1 | 0.26104664 | 0.95912357 | 0.33134025 | . | . |
| MOTIVE*PGQ_ACH_C1 | 0.68922190 | 1 | 0.68922190 | 2.53230222 | 0.11679285 | . | . |
| MOTIVE*PGQ_AFF_C1 | 0.31838628 | 1 | 0.31838628 | 1.16979783 | 0.28377063 | . | . |
| MOTIVE*PGQ_AFF_C2 | 0.76895451 | 1 | 0.76895451 | 2.82525150 | 0.09799161 | . | . |
| MOTIVE*PGQ_IND_C2 | 0.03905300 | 1 | 0.03905300 | 0.14348644 | 0.70617646 | . | . |
| MOTIVE*PGQ_IND_C1 | 0.73388064 | 1 | 0.73388064 | 2.69638497 | 0.10580768 | . | . |
| MOTIVE*ZPOWT1 | 0.00000031 | 1 | 0.00000031 | 0.00000113 | 0.99915657 | . | . |
| MOTIVE*ZPOWT2 | 0.00015115 | 1 | 0.00015115 | 0.00055536 | 0.98127687 | . | . |
| MOTIVE*ZACHT1 | 0.00014635 | 1 | 0.00014635 | 0.00053771 | 0.98157687 | . | . |
| MOTIVE*ZACHT2 | 0.00276003 | 1 | 0.00276003 | 0.01014075 | 0.92012332 | . | . |
| MOTIVE*ZAFFT1 | 0.16633932 | 1 | 0.16633932 | 0.61115502 | 0.43742740 | . | . |
| MOTIVE*ZAFFT2 | 0.17899663 | 1 | 0.17899663 | 0.65765983 | 0.42059260 | . | . |
| Error | 16.33032343 | 60 | 0.27217206 |  |  |  |  |

| Greenhouse-Geisser Epsilon | . |
| --- | --- |
| Huynh-Feldt Epsilon | . |

| **Within Subjects** | | | | | | | |
| --- | --- | --- | --- | --- | --- | --- | --- |
| **Source** | **SS** | **df** | **Mean Squares** | **F-Ratio** | **p-Value** | **G-G** | **H-F** |
| TIME | 0.08635983 | 1 | 0.08635983 | 0.50836819 | 0.47861006 | . | . |
| TIME*TREAT | 0.00156047 | 1 | 0.00156047 | 0.00918592 | 0.92396452 | . | . |
| TIME*PGQ_ACH_C2 | 0.19248735 | 1 | 0.19248735 | 1.13310138 | 0.29137984 | . | . |
| TIME*PGQ_ACH_C1 | 0.40236697 | 1 | 0.40236697 | 2.36858454 | 0.12905679 | . | . |
| TIME*PGQ_AFF_C1 | 0.05915244 | 1 | 0.05915244 | 0.34820838 | 0.55734501 | . | . |
| TIME*PGQ_AFF_C2 | 0.23800274 | 1 | 0.23800274 | 1.40103352 | 0.24121891 | . | . |
| TIME*PGQ_IND_C2 | 0.39316753 | 1 | 0.39316753 | 2.31443085 | 0.13343118 | . | . |
| TIME*PGQ_IND_C1 | 0.57163054 | 1 | 0.57163054 | 3.36497614 | 0.07155613 | . | . |
| TIME*ZPOWT1 | 0.09862709 | 1 | 0.09862709 | 0.58058096 | 0.44906958 | . | . |
| TIME*ZPOWT2 | 0.36103101 | 1 | 0.36103101 | 2.12525516 | 0.15010321 | . | . |
| TIME*ZACHT1 | 0.00009896 | 1 | 0.00009896 | 0.00058253 | 0.98082456 | . | . |
| TIME*ZACHT2 | 0.03877200 | 1 | 0.03877200 | 0.22823633 | 0.63457057 | . | . |
| TIME*ZAFFT1 | 0.34059676 | 1 | 0.34059676 | 2.00496630 | 0.16195520 | . | . |
| TIME*ZAFFT2 | 2.14838667 | 1 | 2.14838667 | 12.64675245 | 0.00074154 | . | . |
| Error | 10.19259299 | 60 | 0.16987655 |  |  |  |  |

| Greenhouse-Geisser Epsilon | . |
| --- | --- |
| Huynh-Feldt Epsilon | . |

| **Within Subjects** | | | | | | | |
| --- | --- | --- | --- | --- | --- | --- | --- |
| **Source** | **SS** | **df** | **Mean Squares** | **F-Ratio** | **p-Value** | **G-G** | **H-F** |
| MOTIVE*TIME | 0.46294477 | 1 | 0.46294477 | 2.63447853 | 0.10980940 | . | . |
| MOTIVE*TIME*TREAT | 0.33463282 | 1 | 0.33463282 | 1.90429406 | 0.17271908 | . | . |
| MOTIVE*TIME*PGQ_ACH_C2 | 0.00249668 | 1 | 0.00249668 | 0.01420787 | 0.90551765 | . | . |
| MOTIVE*TIME*PGQ_ACH_C1 | 0.00176651 | 1 | 0.00176651 | 0.01005268 | 0.92046978 | . | . |
| MOTIVE*TIME*PGQ_AFF_C1 | 0.34219923 | 1 | 0.34219923 | 1.94735222 | 0.16801587 | . | . |
| MOTIVE*TIME*PGQ_AFF_C2 | 1.95694887 | 1 | 1.95694887 | 11.13640351 | 0.00145740 | . | . |
| MOTIVE*TIME*PGQ_IND_C2 | 0.36315512 | 1 | 0.36315512 | 2.06660582 | 0.15575132 | . | . |
| MOTIVE*TIME*PGQ_IND_C1 | 0.04619157 | 1 | 0.04619157 | 0.26286227 | 0.61004296 | . | . |
| MOTIVE*TIME*ZPOWT1 | 0.01996513 | 1 | 0.01996513 | 0.11361549 | 0.73724117 | . | . |
| MOTIVE*TIME*ZPOWT2 | 0.00141848 | 1 | 0.00141848 | 0.00807216 | 0.92870948 | . | . |
| MOTIVE*TIME*ZACHT1 | 0.00144157 | 1 | 0.00144157 | 0.00820356 | 0.92813319 | . | . |
| MOTIVE*TIME*ZACHT2 | 0.10517849 | 1 | 0.10517849 | 0.59853893 | 0.44217364 | . | . |
| MOTIVE*TIME*ZAFFT1 | 0.11118709 | 1 | 0.11118709 | 0.63273208 | 0.42949060 | . | . |
| MOTIVE*TIME*ZAFFT2 | 0.06855481 | 1 | 0.06855481 | 0.39012466 | 0.53460192 | . | . |
| Error | 10.54352350 | 60 | 0.17572539 |  |  |  |  |

| Greenhouse-Geisser Epsilon | . |
| --- | --- |
| Huynh-Feldt Epsilon | . |

> *calc 0.334633/(0.334633+10.543523)*

[▼CALCULATE](file:///\\Untitled.syo)

0.03076192

> *glm*

> *model inkon_ach1 inkon_ach2 = constant treat /repeat={2} names={time}*

> *estimate*

[▼General Linear Model](file:///\\Untitled.syo)

Effects coding used for categorical variables in model.

The categorical values encountered during processing are

| **Variables** | **Levels** | | |
| --- | --- | --- | --- |
| CONDITION (3 levels) | Control | Feedback | Feedback + CET |
| TREAT (2 levels) | CG | FB/FB+CET |  |
| HYPOTH1 (2 levels) | 0.00000000 | 1.00000000 |  |

N of Cases Processed: 74

| **Dependent Variable Means** | |
| --- | --- |
| **INKON_ACH1** | **INKON_ACH2** |
| 0.33877977 | 0.26605341 |

| **Repeated Measures Factors and Levels of Dependent Variables** | | |
| --- | --- | --- |
| **Within Factor** | **1** | **2** |
| TIME | 1.00000000 | 2.00000000 |

**Univariate Repeated Measures Analysis**

| **Between Subjects** | | | | | |
| --- | --- | --- | --- | --- | --- |
| **Source** | **SS** | **df** | **Mean Squares** | **F-Ratio** | **p-Value** |
| TREAT | 0.46337439 | 1 | 0.46337439 | 1.70823402 | 0.19537354 |
| Error | 19.53067049 | 72 | 0.27125931 |  |  |

| **Within Subjects** | | | | | | | |
| --- | --- | --- | --- | --- | --- | --- | --- |
| **Source** | **SS** | **df** | **Mean Squares** | **F-Ratio** | **p-Value** | **G-G** | **H-F** |
| TIME | 0.31661111 | 1 | 0.31661111 | 1.42201418 | 0.23698734 | . | . |
| TIME*TREAT | 0.25607884 | 1 | 0.25607884 | 1.15014200 | 0.28710077 | . | . |
| Error | 16.03078237 | 72 | 0.22264976 |  |  |  |  |

| Greenhouse-Geisser Epsilon | . |
| --- | --- |
| Huynh-Feldt Epsilon | . |

Since your repeated measure has only two levels, Greenhouse-Geisser and

Huynh-Feldt Epsilon corrections are not necessary.

> *calc 0.256079/(0.256079+16.030782)*

[▼CALCULATE](file:///\\Untitled.syo)

0.01572304

> *glm*

> *model inkon_ind1 inkon_ind2 = constant treat /repeat={2} names={time}*

> *estimate*

[▼General Linear Model](file:///\\Untitled.syo)

Effects coding used for categorical variables in model.

The categorical values encountered during processing are

| **Variables** | **Levels** | | |
| --- | --- | --- | --- |
| CONDITION (3 levels) | Control | Feedback | Feedback + CET |
| TREAT (2 levels) | CG | FB/FB+CET |  |
| HYPOTH1 (2 levels) | 0.00000000 | 1.00000000 |  |

N of Cases Processed: 74

| **Dependent Variable Means** | |
| --- | --- |
| **INKON_IND1** | **INKON_IND2** |
| 0.44518095 | 0.35487061 |

| **Repeated Measures Factors and Levels of Dependent Variables** | | |
| --- | --- | --- |
| **Within Factor** | **1** | **2** |
| TIME | 1.00000000 | 2.00000000 |

**Univariate Repeated Measures Analysis**

| **Between Subjects** | | | | | |
| --- | --- | --- | --- | --- | --- |
| **Source** | **SS** | **df** | **Mean Squares** | **F-Ratio** | **p-Value** |
| TREAT | 0.05887588 | 1 | 0.05887588 | 0.20754594 | 0.65006869 |
| Error | 20.42469906 | 72 | 0.28367638 |  |  |

| **Within Subjects** | | | | | | | |
| --- | --- | --- | --- | --- | --- | --- | --- |
| **Source** | **SS** | **df** | **Mean Squares** | **F-Ratio** | **p-Value** | **G-G** | **H-F** |
| TIME | 0.11395410 | 1 | 0.11395410 | 0.69953023 | 0.40570888 | . | . |
| TIME*TREAT | 0.50110841 | 1 | 0.50110841 | 3.07615511 | 0.08370346 | . | . |
| Error | 11.72886428 | 72 | 0.16290089 |  |  |  |  |

| Greenhouse-Geisser Epsilon | . |
| --- | --- |
| Huynh-Feldt Epsilon | . |

Since your repeated measure has only two levels, Greenhouse-Geisser and

Huynh-Feldt Epsilon corrections are not necessary.

> *calc 0.501108/(0.501108+11.728864)*

[▼CALCULATE](file:///\\Untitled.syo)

0.04097377

> *glm*

> *model inkon_aff1 inkon_aff2 = constant treat /repeat={2} names={time}*

> *estimate*

[▼General Linear Model](file:///\\Untitled.syo)

Effects coding used for categorical variables in model.

The categorical values encountered during processing are

| **Variables** | **Levels** | | |
| --- | --- | --- | --- |
| CONDITION (3 levels) | Control | Feedback | Feedback + CET |
| TREAT (2 levels) | CG | FB/FB+CET |  |
| HYPOTH1 (2 levels) | 0.00000000 | 1.00000000 |  |

N of Cases Processed: 74

| **Dependent Variable Means** | |
| --- | --- |
| **T1** | **T2** |
| 0.33486522 | 0.39177101 |

| **Repeated Measures Factors and Levels of Dependent Variables** | | |
| --- | --- | --- |
| **Within Factor** | **1** | **2** |
| TIME | 1.00000000 | 2.00000000 |

**Univariate Repeated Measures Analysis**

| **Between Subjects** | | | | | |
| --- | --- | --- | --- | --- | --- |
| **Source** | **SS** | **df** | **Mean Squares** | **F-Ratio** | **p-Value** |
| TREAT | 0.88339671 | 1 | 0.88339671 | 2.81878540 | 0.09750206 |
| Error | 22.56452828 | 72 | 0.31339623 |  |  |

| **Within Subjects** | | | | | | | |
| --- | --- | --- | --- | --- | --- | --- | --- |
| **Source** | **SS** | **df** | **Mean Squares** | **F-Ratio** | **p-Value** | **G-G** | **H-F** |
| TIME | 0.03141622 | 1 | 0.03141622 | 0.14431491 | 0.70514682 | . | . |
| TIME*TREAT | 0.33322931 | 1 | 0.33322931 | 1.53073644 | 0.22002187 | . | . |
| Error | 15.67383487 | 72 | 0.21769215 |  |  |  |  |

| Greenhouse-Geisser Epsilon | . |
| --- | --- |
| Huynh-Feldt Epsilon | . |

Since your repeated measure has only two levels, Greenhouse-Geisser and

Huynh-Feldt Epsilon corrections are not necessary.

> *calc 0.333229/(0.333229+15.673835)*

[▼CALCULATE](file:///\\Untitled.syo)

0.02081762

> *by treat*

> *testing*

> *ttest inkon_ind2 inkon_ind1*

[▼Hypothesis Testing: Paired t-test](file:///\\Untitled.syo)

**Results for TREAT = CG**

**H0: Mean Difference = 0 vs. H1: Mean Difference <> 0**

| **Variable** | **N** | **Mean** |
| --- | --- | --- |
| INKON_IND2 | 27.00000000 | 0.45795755 |
| INKON_IND1 | 27.00000000 | 0.39472418 |

| **Variable** | **Mean Difference** | **95.00% Confidence Interval** | | **Standard Deviation of Difference** | **t** | **df** | **p-Value** |
| --- | --- | --- | --- | --- | --- | --- | --- |
|  |  | **Lower Limit** | **Upper Limit** |  |  |  |  |
| INKON_IND2 | 0.06323337 | -0.14704756 | 0.27351430 | 0.53156707 | 0.61811621 | 26.00000000 | 0.54187596 |
| INKON_IND1 |  |  |  |  |  |  |  |

**Results for TREAT = FB/FB+CET**

**H0: Mean Difference = 0 vs. H1: Mean Difference <> 0**

| **Variable** | **N** | **Mean** |
| --- | --- | --- |
| INKON_IND2 | 47.00000000 | 0.29565044 |
| INKON_IND1 | 47.00000000 | 0.47416675 |

| **Variable** | **Mean Difference** | **95.00% Confidence Interval** | | **Standard Deviation of Difference** | **t** | **df** | **p-Value** |
| --- | --- | --- | --- | --- | --- | --- | --- |
|  |  | **Lower Limit** | **Upper Limit** |  |  |  |  |
| INKON_IND2 | -0.17851630 | -0.35227866 | -0.00475395 | 0.59181145 | -2.06796628 | 46.00000000 | 0.04429334 |
| INKON_IND1 |  |  |  |  |  |  |  |

> *by*

> *testing*

> *ttest inkon_ind2 inkon_ind2 * treat*

[▼Hypothesis Testing: Two-sample t-test](file:///\\Untitled.syo)

**H0: Mean1 = Mean2 vs. H1: Mean1 <> Mean2**

Grouping Variable = TREAT

| **Variable** | **TREAT** | **N** | **Mean** | **Standard Deviation** |
| --- | --- | --- | --- | --- |
| INKON_IND2 | CG | 27.00000000 | 0.45795755 | 0.47217745 |
|  | FB/FB+CET | 47.00000000 | 0.29565044 | 0.52097342 |
| INKON_IND2 | CG | 27.00000000 | 0.45795755 | 0.47217745 |
|  | FB/FB+CET | 47.00000000 | 0.29565044 | 0.52097342 |

**Separate Variance**

| **Variable** | **TREAT** | **Mean Difference** | **95.00% Confidence Interval** | | **t** | **df** | **p-Value** |
| --- | --- | --- | --- | --- | --- | --- | --- |
|  |  |  | **Lower Limit** | **Upper Limit** |  |  |  |
| INKON_IND2 | CG | 0.16230711 | -0.07474118 | 0.39935540 | 1.37016988 | 58.82130605 | 0.17584095 |
|  | FB/FB+CET |  |  |  |  |  |  |
| INKON_IND2 | CG | 0.16230711 | -0.07474118 | 0.39935540 | 1.37016988 | 58.82130605 | 0.17584095 |
|  | FB/FB+CET |  |  |  |  |  |  |

**Pooled Variance**

| **Variable** | **TREAT** | **Mean Difference** | **95.00% Confidence Interval** | | **t** | **df** | **p-Value** |
| --- | --- | --- | --- | --- | --- | --- | --- |
|  |  |  | **Lower Limit** | **Upper Limit** |  |  |  |
| INKON_IND2 | CG | 0.16230711 | -0.08026230 | 0.40487651 | 1.33385873 | 72.00000000 | 0.18645418 |
|  | FB/FB+CET |  |  |  |  |  |  |
| INKON_IND2 | CG | 0.16230711 | -0.08026230 | 0.40487651 | 1.33385873 | 72.00000000 | 0.18645418 |
|  | FB/FB+CET |  |  |  |  |  |  |

> *vari inkon_ind2 inkon_ind2 *treat*

[▼Hypothesis Testing: Equality of Two Variances](file:///\\Untitled.syo)

**H0: Variance1 = Variance2 vs. H1: Variance1 <> Variance2**

Grouping Variable = TREAT

| **Variable** | **TREAT** | **N** | **Mean** | **Variance** |
| --- | --- | --- | --- | --- |
| INKON_IND2 | CG | 27.00000000 | 0.45795755 | 0.22295154 |
|  | FB/FB+CET | 47.00000000 | 0.29565044 | 0.27141330 |
| INKON_IND2 | CG | 27.00000000 | 0.45795755 | 0.22295154 |
|  | FB/FB+CET | 47.00000000 | 0.29565044 | 0.27141330 |

| **Variable** | **TREAT** | **95.00% Confidence Interval** | | **F-Ratio** | **df** | **p-Value** |
| --- | --- | --- | --- | --- | --- | --- |
|  |  | **Lower Limit** | **Upper Limit** |  |  |  |
| INKON_IND2 | CG | 0.42497250 | 1.69791411 | 0.82144663 | 26, 46 | 0.59954807 |
|  | FB/FB+CET |  |  |  |  |  |
| INKON_IND2 | CG | 0.42497250 | 1.69791411 | 0.82144663 | 26, 46 | 0.59954807 |
|  | FB/FB+CET |  |  |  |  |  |

> *!!*

> *!!*

> *!!*

> *!! Testing hypothesis 2*

> *!!*

> *!!*

> *!!*

> *glm*

> *model ht1 sqr_bdi_t1 swls_t1 = constant condition /repeat=3 names=domain*

> *estimate*

[▼General Linear Model](file:///\\Untitled.syo)

Effects coding used for categorical variables in model.

The categorical values encountered during processing are

| **Variables** | **Levels** | | |
| --- | --- | --- | --- |
| CONDITION (3 levels) | Control | Feedback | Feedback + CET |
| TREAT (2 levels) | CG | FB/FB+CET |  |
| HYPOTH1 (2 levels) | 0.00000000 | 1.00000000 |  |

N of Cases Processed: 74

| **Dependent Variable Means** | | |
| --- | --- | --- |
| **HT1** | **SQR_BDI_T1** | **SWLS_T1** |
| 3.53603604 | 2.87720761 | 4.75945946 |

| **Repeated Measures Factors and Levels of Dependent Variables** | | | |
| --- | --- | --- | --- |
| **Within Factor** | **1** | **2** | **3** |
| DOMAIN | 1.00000000 | 2.00000000 | 3.00000000 |

**Univariate and Multivariate Repeated Measures Analysis**

| **Between Subjects** | | | | | |
| --- | --- | --- | --- | --- | --- |
| **Source** | **SS** | **df** | **Mean Squares** | **F-Ratio** | **p-Value** |
| CONDITION | 0.04365764 | 2 | 0.02182882 | 0.04231689 | 0.95859013 |
| Error | 36.62476765 | 71 | 0.51584180 |  |  |

| **Within Subjects** | | | | | | | |
| --- | --- | --- | --- | --- | --- | --- | --- |
| **Source** | **SS** | **df** | **Mean Squares** | **F-Ratio** | **p-Value** | **G-G** | **H-F** |
| DOMAIN | 135.23689060 | 2 | 67.61844530 | 55.07274297 | 0.00000000 | 0.00000000 | 0.00000000 |
| DOMAIN*CONDITION | 0.79663420 | 4 | 0.19915855 | 0.16220733 | 0.95712631 | 0.91580291 | 0.92219267 |
| Error | 174.34793901 | 142 | 1.22780239 |  |  |  |  |

| Greenhouse-Geisser Epsilon | 0.72151824 |
| --- | --- |
| Huynh-Feldt Epsilon | 0.75322939 |

**Multivariate Repeated Measures Analysis**

| **Test of: DOMAIN** | | | | | |
| --- | --- | --- | --- | --- | --- |
| **Statistic** | **Value** | **Hypothesis df** | **Error df** | **F-Ratio** | **p-Value** |
| Wilks's Lambda | 0.32982723 | 2 | 70 | 71.11616402 | 0.00000000 |
| Pillai Trace | 0.67017277 | 2 | 70 | 71.11616402 | 0.00000000 |
| Hotelling-Lawley Trace | 2.03189040 | 2 | 70 | 71.11616402 | 0.00000000 |

| **Test of: DOMAIN*CONDITION** | | | | | |
| --- | --- | --- | --- | --- | --- |
| **Statistic** | **Value** | **Hypothesis df** | **Error df** | **F-Ratio** | **p-Value** |
| Wilks's Lambda | 0.97995320 | 4 | 140 | 0.35618323 | 0.83938183 |
| Pillai Trace | 0.02006771 | 4 | 142 | 0.35981214 | 0.83685555 |
| Hotelling-Lawley Trace | 0.02043555 | 4 | 138 | 0.35251326 | 0.84192918 |

| **THETA** | **S** | **M** | **N** | **p-Value** |
| --- | --- | --- | --- | --- |
| 0.01896502 | 2 | -0.50000000 | 34.00000000 | 0.74147049 |

> *glm*

> *model ht1 ht2 = constant condition /repeat=2 names=time*

> *estimate*

[▼General Linear Model](file:///\\Untitled.syo)

Effects coding used for categorical variables in model.

The categorical values encountered during processing are

| **Variables** | **Levels** | | |
| --- | --- | --- | --- |
| CONDITION (3 levels) | Control | Feedback | Feedback + CET |
| TREAT (2 levels) | CG | FB/FB+CET |  |
| HYPOTH1 (2 levels) | 0.00000000 | 1.00000000 |  |

1 case(s) are deleted due to missing data.

N of Cases Processed: 73

| **Dependent Variable Means** | |
| --- | --- |
| **HT1** | **HT2** |
| 3.53538813 | 3.64840183 |

| **Repeated Measures Factors and Levels of Dependent Variables** | | |
| --- | --- | --- |
| **Within Factor** | **1** | **2** |
| TIME | 1.00000000 | 2.00000000 |

**Univariate Repeated Measures Analysis**

| **Between Subjects** | | | | | |
| --- | --- | --- | --- | --- | --- |
| **Source** | **SS** | **df** | **Mean Squares** | **F-Ratio** | **p-Value** |
| CONDITION | 0.15695741 | 2 | 0.07847871 | 0.10415117 | 0.90122844 |
| Error | 52.74553498 | 70 | 0.75350764 |  |  |

| **Within Subjects** | | | | | | | |
| --- | --- | --- | --- | --- | --- | --- | --- |
| **Source** | **SS** | **df** | **Mean Squares** | **F-Ratio** | **p-Value** | **G-G** | **H-F** |
| TIME | 0.53465429 | 1 | 0.53465429 | 1.29757419 | 0.25853991 | . | . |
| TIME*CONDITION | 0.25689242 | 2 | 0.12844621 | 0.31173132 | 0.73318936 | . | . |
| Error | 28.84289830 | 70 | 0.41204140 |  |  |  |  |

| Greenhouse-Geisser Epsilon | . |
| --- | --- |
| Huynh-Feldt Epsilon | . |

Since your repeated measure has only two levels, Greenhouse-Geisser and

Huynh-Feldt Epsilon corrections are not necessary.

> *calc 0.256892/(0.256892+28.842898)*

[▼CALCULATE](file:///\\Untitled.syo)

0.00882797

> *glm*

> *model swls_t1 swls_t2 = constant condition /repeat=2 names=time*

> *estimate*

[▼General Linear Model](file:///\\Untitled.syo)

Effects coding used for categorical variables in model.

The categorical values encountered during processing are

| **Variables** | **Levels** | | |
| --- | --- | --- | --- |
| CONDITION (3 levels) | Control | Feedback | Feedback + CET |
| TREAT (2 levels) | CG | FB/FB+CET |  |
| HYPOTH1 (2 levels) | 0.00000000 | 1.00000000 |  |

N of Cases Processed: 74

| **Dependent Variable Means** | |
| --- | --- |
| **SWLS_T1** | **SWLS_T2** |
| 4.75945946 | 4.85945946 |

| **Repeated Measures Factors and Levels of Dependent Variables** | | |
| --- | --- | --- |
| **Within Factor** | **1** | **2** |
| TIME | 1.00000000 | 2.00000000 |

**Univariate Repeated Measures Analysis**

| **Between Subjects** | | | | | |
| --- | --- | --- | --- | --- | --- |
| **Source** | **SS** | **df** | **Mean Squares** | **F-Ratio** | **p-Value** |
| CONDITION | 0.76344497 | 2 | 0.38172249 | 0.18466670 | 0.83177937 |
| Error | 146.76331178 | 71 | 2.06708890 |  |  |

| **Within Subjects** | | | | | | | |
| --- | --- | --- | --- | --- | --- | --- | --- |
| **Source** | **SS** | **df** | **Mean Squares** | **F-Ratio** | **p-Value** | **G-G** | **H-F** |
| TIME | 0.34419824 | 1 | 0.34419824 | 2.11468774 | 0.15029830 | . | . |
| TIME*CONDITION | 0.09364848 | 2 | 0.04682424 | 0.28767914 | 0.75087223 | . | . |
| Error | 11.55635152 | 71 | 0.16276551 |  |  |  |  |

| Greenhouse-Geisser Epsilon | . |
| --- | --- |
| Huynh-Feldt Epsilon | . |

Since your repeated measure has only two levels, Greenhouse-Geisser and

Huynh-Feldt Epsilon corrections are not necessary.

> *calc 0.093648/(0.093648+11.556352)*

[▼CALCULATE](file:///\\Untitled.syo)

0.00803845

> *glm*

> *model sqr_bdi_t1 sqr_bdi_t2 = constant condition /repeat=2 names=time*

> *estimate*

[▼General Linear Model](file:///\\Untitled.syo)

Effects coding used for categorical variables in model.

The categorical values encountered during processing are

| **Variables** | **Levels** | | |
| --- | --- | --- | --- |
| CONDITION (3 levels) | Control | Feedback | Feedback + CET |
| TREAT (2 levels) | CG | FB/FB+CET |  |
| HYPOTH1 (2 levels) | 0.00000000 | 1.00000000 |  |

1 case(s) are deleted due to missing data.

N of Cases Processed: 73

| **Dependent Variable Means** | |
| --- | --- |
| **SQR_BDI_T1** | **SQR_BDI_T2** |
| 2.85850305 | 2.69269447 |

| **Repeated Measures Factors and Levels of Dependent Variables** | | |
| --- | --- | --- |
| **Within Factor** | **1** | **2** |
| TIME | 1.00000000 | 2.00000000 |

**Univariate Repeated Measures Analysis**

| **Between Subjects** | | | | | |
| --- | --- | --- | --- | --- | --- |
| **Source** | **SS** | **df** | **Mean Squares** | **F-Ratio** | **p-Value** |
| CONDITION | 0.71490379 | 2 | 0.35745190 | 0.19164002 | 0.82603572 |
| Error | 130.56580436 | 70 | 1.86522578 |  |  |

| **Within Subjects** | | | | | | | |
| --- | --- | --- | --- | --- | --- | --- | --- |
| **Source** | **SS** | **df** | **Mean Squares** | **F-Ratio** | **p-Value** | **G-G** | **H-F** |
| TIME | 1.12601018 | 1 | 1.12601018 | 3.39141590 | 0.06977337 | . | . |
| TIME*CONDITION | 0.69809928 | 2 | 0.34904964 | 1.05129822 | 0.35493576 | . | . |
| Error | 23.24124053 | 70 | 0.33201772 |  |  |  |  |

| Greenhouse-Geisser Epsilon | . |
| --- | --- |
| Huynh-Feldt Epsilon | . |

Since your repeated measure has only two levels, Greenhouse-Geisser and

Huynh-Feldt Epsilon corrections are not necessary.

> *calc 0.698099/(0.698099+23.241241)*

[▼CALCULATE](file:///\\Untitled.syo)

0.02916116

> *select*

> *glm*

> *model sqr_bdi_t2 = constant sqr_bdi_t1 treat*

> *estimate*

[▼General Linear Model](file:///\\Untitled.syo)

Effects coding used for categorical variables in model.

The categorical values encountered during processing are

| **Variables** | **Levels** | | |
| --- | --- | --- | --- |
| CONDITION (3 levels) | Control | Feedback | Feedback + CET |
| TREAT (2 levels) | CG | FB/FB+CET |  |
| HYPOTH1 (2 levels) | 0.00000000 | 1.00000000 |  |

1 case(s) are deleted due to missing data.

| Dependent Variable | SQR_BDI_T2 |
| --- | --- |
| N | 73 |
| Multiple R | 0.70922756 |
| Squared Multiple R | 0.50300374 |

| **Estimates of Effects B = (X'X)^-1^X'Y** | | |
| --- | --- | --- |
| **Factor** | **Level** | **SQR_BDI_T2** |
| CONSTANT |  | 0.93481469 |
| SQR_BDI_T1 |  | 0.62791864 |
| TREAT | CG | 0.14226383 |

| **Analysis of Variance** | | | | | |
| --- | --- | --- | --- | --- | --- |
| **Source** | **Type III SS** | **df** | **Mean Squares** | **F-Ratio** | **p-Value** |
| SQR_BDI_T1 | 33.71647380 | 1 | 33.71647380 | 68.12668244 | 0.00000000 |
| TREAT | 1.37735342 | 1 | 1.37735342 | 2.78304664 | 0.09973433 |
| Error | 34.64359457 | 70 | 0.49490849 |  |  |

WARNING

| Case | 406.00000000 | is an Outlier | (Studentized Residual | : | 3.35156089) |
| --- | --- | --- | --- | --- | --- |

| Durbin-Watson D-Statistic | 1.73663883 |
| --- | --- |
| First Order Autocorrelation | 0.08862915 |

| **Information Criteria** | |
| --- | --- |
| AIC | 160.75472453 |
| AIC (Corrected) | 161.34295982 |
| Schwarz's BIC | 169.91656229 |

> *calc 1.377353 /(1.377353+34.643595)*

[▼CALCULATE](file:///\\Untitled.syo)

0.03823756

> *by treat*

> *testing*

> *ttest sqr_bdi_t1 sqr_bdi_t2*

[▼Hypothesis Testing: Paired t-test](file:///\\Untitled.syo)

**Results for TREAT = CG**

**H0: Mean Difference = 0 vs. H1: Mean Difference <> 0**

| **Variable** | **N** | **Mean** |
| --- | --- | --- |
| SQR_BDI_T1 | 27.00000000 | 2.85522085 |
| SQR_BDI_T2 | 27.00000000 | 2.86992492 |

| **Variable** | **Mean Difference** | **95.00% Confidence Interval** | | **Standard Deviation of Difference** | **t** | **df** | **p-Value** |
| --- | --- | --- | --- | --- | --- | --- | --- |
|  |  | **Lower Limit** | **Upper Limit** |  |  |  |  |
| SQR_BDI_T1 | -0.01470406 | -0.36770583 | 0.33829770 | 0.89234964 | -0.08562177 | 26.00000000 | 0.93242304 |
| SQR_BDI_T2 |  |  |  |  |  |  |  |

**Results for TREAT = FB/FB+CET**

**H0: Mean Difference = 0 vs. H1: Mean Difference <> 0**

| **Variable** | **N** | **Mean** |
| --- | --- | --- |
| SQR_BDI_T1 | 46.00000000 | 2.86042956 |
| SQR_BDI_T2 | 46.00000000 | 2.58866790 |

| **Variable** | **Mean Difference** | **95.00% Confidence Interval** | | **Standard Deviation of Difference** | **t** | **df** | **p-Value** |
| --- | --- | --- | --- | --- | --- | --- | --- |
|  |  | **Lower Limit** | **Upper Limit** |  |  |  |  |
| SQR_BDI_T1 | 0.27176166 | 0.04699615 | 0.49652718 | 0.75687965 | 2.43523165 | 45.00000000 | 0.01890876 |
| SQR_BDI_T2 |  |  |  |  |  |  |  |

> *by*

> *testing*

> *ttest sqr_bdi_t1 sqr_bdi_t2 * treat*

[▼Hypothesis Testing: Two-sample t-test](file:///\\Untitled.syo)

**H0: Mean1 = Mean2 vs. H1: Mean1 <> Mean2**

Grouping Variable = TREAT

| **Variable** | **TREAT** | **N** | **Mean** | **Standard Deviation** |
| --- | --- | --- | --- | --- |
| SQR_BDI_T1 | CG | 27.00000000 | 2.85522085 | 1.03567657 |
|  | FB/FB+CET | 47.00000000 | 2.88983831 | 1.13726621 |
| SQR_BDI_T2 | CG | 27.00000000 | 2.86992492 | 1.10271232 |
|  | FB/FB+CET | 46.00000000 | 2.58866790 | 0.90363134 |

**Separate Variance**

| **Variable** | **TREAT** | **Mean Difference** | **95.00% Confidence Interval** | | **t** | **df** | **p-Value** |
| --- | --- | --- | --- | --- | --- | --- | --- |
|  |  |  | **Lower Limit** | **Upper Limit** |  |  |  |
| SQR_BDI_T1 | CG | -0.03461745 | -0.55358399 | 0.48434908 | -0.13349455 | 58.60230508 | 0.89425965 |
|  | FB/FB+CET |  |  |  |  |  |  |
| SQR_BDI_T2 | CG | 0.28125702 | -0.22301228 | 0.78552632 | 1.12245237 | 46.37270328 | 0.26744824 |
|  | FB/FB+CET |  |  |  |  |  |  |

**Pooled Variance**

| **Variable** | **TREAT** | **Mean Difference** | **95.00% Confidence Interval** | | **t** | **df** | **p-Value** |
| --- | --- | --- | --- | --- | --- | --- | --- |
|  |  |  | **Lower Limit** | **Upper Limit** |  |  |  |
| SQR_BDI_T1 | CG | -0.03461745 | -0.56494213 | 0.49570722 | -0.13012526 | 72.00000000 | 0.89683019 |
|  | FB/FB+CET |  |  |  |  |  |  |
| SQR_BDI_T2 | CG | 0.28125702 | -0.19307837 | 0.75559242 | 1.18230812 | 71.00000000 | 0.24102781 |
|  | FB/FB+CET |  |  |  |  |  |  |

> *vari sqr_bdi_t1 sqr_bdi_t2 * treat*

[▼Hypothesis Testing: Equality of Two Variances](file:///\\Untitled.syo)

**H0: Variance1 = Variance2 vs. H1: Variance1 <> Variance2**

Grouping Variable = TREAT

| **Variable** | **TREAT** | **N** | **Mean** | **Variance** |
| --- | --- | --- | --- | --- |
| SQR_BDI_T1 | CG | 27.00000000 | 2.85522085 | 1.07262595 |
|  | FB/FB+CET | 47.00000000 | 2.88983831 | 1.29337444 |
| SQR_BDI_T2 | CG | 27.00000000 | 2.86992492 | 1.21597447 |
|  | FB/FB+CET | 46.00000000 | 2.58866790 | 0.81654960 |

| **Variable** | **TREAT** | **95.00% Confidence Interval** | | **F-Ratio** | **df** | **p-Value** |
| --- | --- | --- | --- | --- | --- | --- |
|  |  | **Lower Limit** | **Upper Limit** |  |  |  |
| SQR_BDI_T1 | CG | 0.42904762 | 1.71419565 | 0.82932360 | 26, 46 | 0.61828925 |
|  | FB/FB+CET |  |  |  |  |  |
| SQR_BDI_T2 | CG | 0.76750078 | 3.08381520 | 1.48916179 | 26, 45 | 0.23690497 |
|  | FB/FB+CET |  |  |  |  |  |

> *!!*

> *!!*

> *!!*

> *!! Testing hypothesis 3*

> *!!*

> *!!*

> *!!*

> *!!*

> *!! See analyses above in preparation for Figure 4*

> *!!*

> *categ /off*

> *select treat =1*

> *setcor*

> *model sqr_bdi_t2 | sqr_bdi_t1 = agkon2 | agkon1*

> *estimate*

[▼Set and Canonical Correlations](file:///\\Untitled.syo)

Data for the following results were selected according to

select treat =1

1 Cases deleted due to missing data.

**Bipartial Set Correlation Analysis (Y|YPARTIAL vs. X|XPARTIAL)**

Number of Cases on which Analysis is based: 46

Dependent Set y Partialled by these Variables

SQR_BDI_T1

Independent Set x Partialled by these Variables

AGKON1

| RAO F | : | 4.08900055 |  |  |
| --- | --- | --- | --- | --- |
| df | : | 1.00000000 | , | 43.00000000 |
| p-Value | : | 0.04941256 |  |  |

| R-square | : | 0.08678123 | Shrunk R-square | : | 0.06554358 |
| --- | --- | --- | --- | --- | --- |
| T-square | : | 0.08678123 | Shrunk T-square | : | 0.06554358 |
| P-square | : | 0.08678123 | Shrunk P-square | : | 0.06554358 |

| **Within Basic Set y Correlations** | |
| --- | --- |
|  | **SQR_BDI_T2** |
| SQR_BDI_T2 | 1.00000000 |

| **Within Basic Set x Correlations** | |
| --- | --- |
|  | **AGKON2** |
| AGKON2 | 1.00000000 |

| **Between Basic y (col) and Basic x (row) Correlations** | |
| --- | --- |
|  | **SQR_BDI_T2** |
| AGKON2 | -0.29458653 |

| **Estimated (from x-set) y Intercorrelations (R-square on diagonal)** | |
| --- | --- |
|  | **SQR_BDI_T2** |
| SQR_BDI_T2 | 0.08678123 |

| **Significance Tests for Prediction of Each Basic y Variable** | | |
| --- | --- | --- |
| **Variable** | **F-Ratio** | **p-Value** |
| SQR_BDI_T2 | 4.08900055 | 0.04941256 |

| **Betas Predicting Basic y (col) from Basic x (row) Variables** | |
| --- | --- |
|  | **SQR_BDI_T2** |
| AGKON2 | -0.29458653 |

| **Standard Error of Betas** | |
| --- | --- |
|  | **SQR_BDI_T2** |
| AGKON2 | 0.14568147 |

| **t-Statistic for Betas** | |
| --- | --- |
|  | **SQR_BDI_T2** |
| AGKON2 | -2.02212773 |

| **p-Value for Betas** | |
| --- | --- |
|  | **SQR_BDI_T2** |
| AGKON2 | 0.04941256 |

> *setcor*

> *model sqr_bdi_t2 | sqr_bdi_t1 = inkon_ind2 | inkon_ind1*

> *estimate*

[▼Set and Canonical Correlations](file:///\\Untitled.syo)

Data for the following results were selected according to

select treat =1

1 Cases deleted due to missing data.

**Bipartial Set Correlation Analysis (Y|YPARTIAL vs. X|XPARTIAL)**

Number of Cases on which Analysis is based: 46

Dependent Set y Partialled by these Variables

SQR_BDI_T1

Independent Set x Partialled by these Variables

INKON_IND1

| RAO F | : | 3.40089639 |  |  |
| --- | --- | --- | --- | --- |
| df | : | 1.00000000 | , | 43.00000000 |
| p-Value | : | 0.07205795 |  |  |

| R-square | : | 0.07057903 | Shrunk R-square | : | 0.04896459 |
| --- | --- | --- | --- | --- | --- |
| T-square | : | 0.07057903 | Shrunk T-square | : | 0.04896459 |
| P-square | : | 0.07057903 | Shrunk P-square | : | 0.04896459 |

| **Within Basic Set y Correlations** | |
| --- | --- |
|  | **SQR_BDI_T2** |
| SQR_BDI_T2 | 1.00000000 |

| **Within Basic Set x Correlations** | |
| --- | --- |
|  | **INKON_IND2** |
| INKON_IND2 | 1.00000000 |

| **Between Basic y (col) and Basic x (row) Correlations** | |
| --- | --- |
|  | **SQR_BDI_T2** |
| INKON_IND2 | 0.26566714 |

| **Estimated (from x-set) y Intercorrelations (R-square on diagonal)** | |
| --- | --- |
|  | **SQR_BDI_T2** |
| SQR_BDI_T2 | 0.07057903 |

| **Significance Tests for Prediction of Each Basic y Variable** | | |
| --- | --- | --- |
| **Variable** | **F-Ratio** | **p-Value** |
| SQR_BDI_T2 | 3.40089639 | 0.07205795 |

| **Betas Predicting Basic y (col) from Basic x (row) Variables** | |
| --- | --- |
|  | **SQR_BDI_T2** |
| INKON_IND2 | 0.26566714 |

| **Standard Error of Betas** | |
| --- | --- |
|  | **SQR_BDI_T2** |
| INKON_IND2 | 0.14405925 |

| **t-Statistic for Betas** | |
| --- | --- |
|  | **SQR_BDI_T2** |
| INKON_IND2 | 1.84415194 |

| **p-Value for Betas** | |
| --- | --- |
|  | **SQR_BDI_T2** |
| INKON_IND2 | 0.07205795 |

> *select treat =0*

> *setcor*

> *model sqr_bdi_t2 | sqr_bdi_t1 = agkon2 | agkon1*

> *estimate*

[▼Set and Canonical Correlations](file:///\\Untitled.syo)

Data for the following results were selected according to

select treat =0

**Bipartial Set Correlation Analysis (Y|YPARTIAL vs. X|XPARTIAL)**

Number of Cases on which Analysis is based: 27

Dependent Set y Partialled by these Variables

SQR_BDI_T1

Independent Set x Partialled by these Variables

AGKON1

| RAO F | : | 0.89906050 |  |  |
| --- | --- | --- | --- | --- |
| df | : | 1.00000000 | , | 24.00000000 |
| p-Value | : | 0.35248429 |  |  |

| R-square | : | 0.03546651 | Shrunk R-square | : | 0.00000000 |
| --- | --- | --- | --- | --- | --- |
| T-square | : | 0.03546651 | Shrunk T-square | : | 0.00000000 |
| P-square | : | 0.03546651 | Shrunk P-square | : | 0.00000000 |

| **Within Basic Set y Correlations** | |
| --- | --- |
|  | **SQR_BDI_T2** |
| SQR_BDI_T2 | 1.00000000 |

| **Within Basic Set x Correlations** | |
| --- | --- |
|  | **AGKON2** |
| AGKON2 | 1.00000000 |

| **Between Basic y (col) and Basic x (row) Correlations** | |
| --- | --- |
|  | **SQR_BDI_T2** |
| AGKON2 | -0.18832555 |

| **Estimated (from x-set) y Intercorrelations (R-square on diagonal)** | |
| --- | --- |
|  | **SQR_BDI_T2** |
| SQR_BDI_T2 | 0.03546651 |

| **Significance Tests for Prediction of Each Basic y Variable** | | |
| --- | --- | --- |
| **Variable** | **F-Ratio** | **p-Value** |
| SQR_BDI_T2 | 0.89906050 | 0.35248429 |

| **Betas Predicting Basic y (col) from Basic x (row) Variables** | |
| --- | --- |
|  | **SQR_BDI_T2** |
| AGKON2 | -0.18832555 |

| **Standard Error of Betas** | |
| --- | --- |
|  | **SQR_BDI_T2** |
| AGKON2 | 0.19861626 |

| **t-Statistic for Betas** | |
| --- | --- |
|  | **SQR_BDI_T2** |
| AGKON2 | -0.94818801 |

| **p-Value for Betas** | |
| --- | --- |
|  | **SQR_BDI_T2** |
| AGKON2 | 0.35248429 |

> *setcor*

> *model sqr_bdi_t2 | sqr_bdi_t1 = inkon_ind2 | inkon_ind1*

> *estimate*

[▼Set and Canonical Correlations](file:///\\Untitled.syo)

Data for the following results were selected according to

select treat =0

**Bipartial Set Correlation Analysis (Y|YPARTIAL vs. X|XPARTIAL)**

Number of Cases on which Analysis is based: 27

Dependent Set y Partialled by these Variables

SQR_BDI_T1

Independent Set x Partialled by these Variables

INKON_IND1

| RAO F | : | 0.18429025 |  |  |
| --- | --- | --- | --- | --- |
| df | : | 1.00000000 | , | 24.00000000 |
| p-Value | : | 0.67154116 |  |  |

| R-square | : | 0.00761984 | Shrunk R-square | : | 0.00000000 |
| --- | --- | --- | --- | --- | --- |
| T-square | : | 0.00761984 | Shrunk T-square | : | 0.00000000 |
| P-square | : | 0.00761984 | Shrunk P-square | : | 0.00000000 |

| **Within Basic Set y Correlations** | |
| --- | --- |
|  | **SQR_BDI_T2** |
| SQR_BDI_T2 | 1.00000000 |

| **Within Basic Set x Correlations** | |
| --- | --- |
|  | **INKON_IND2** |
| INKON_IND2 | 1.00000000 |

| **Between Basic y (col) and Basic x (row) Correlations** | |
| --- | --- |
|  | **SQR_BDI_T2** |
| INKON_IND2 | 0.08729167 |

| **Estimated (from x-set) y Intercorrelations (R-square on diagonal)** | |
| --- | --- |
|  | **SQR_BDI_T2** |
| SQR_BDI_T2 | 0.00761984 |

| **Significance Tests for Prediction of Each Basic y Variable** | | |
| --- | --- | --- |
| **Variable** | **F-Ratio** | **p-Value** |
| SQR_BDI_T2 | 0.18429025 | 0.67154116 |

| **Betas Predicting Basic y (col) from Basic x (row) Variables** | |
| --- | --- |
|  | **SQR_BDI_T2** |
| INKON_IND2 | 0.08729167 |

| **Standard Error of Betas** | |
| --- | --- |
|  | **SQR_BDI_T2** |
| INKON_IND2 | 0.20333944 |

| **t-Statistic for Betas** | |
| --- | --- |
|  | **SQR_BDI_T2** |
| INKON_IND2 | 0.42929041 |

| **p-Value for Betas** | |
| --- | --- |
|  | **SQR_BDI_T2** |
| INKON_IND2 | 0.67154116 |

> *select*

> *glm*

> *model sqr_bdi_t2 = constant treat sqr_bdi_t1*

> *estimate*

[▼General Linear Model](file:///\\Untitled.syo)

1 case(s) are deleted due to missing data.

| Dependent Variable | SQR_BDI_T2 |
| --- | --- |
| N | 73 |
| Multiple R | 0.70922756 |
| Squared Multiple R | 0.50300374 |
| Adjusted Squared Multiple R | 0.48880384 |
| Standard Error of Estimate | 0.70349733 |

| **Regression Coefficients B = (X'X)^-1^X'Y** | | | | | | |
| --- | --- | --- | --- | --- | --- | --- |
| **Effect** | **Coefficient** | **Standard Error** | **Std. Coefficient** | **Tolerance** | **t** | **p-Value** |
| CONSTANT | 1.07707852 | 0.25595144 | 0.00000000 | . | 4.20813626 | 0.00007516 |
| TREAT | -0.28452767 | 0.17055492 | -0.14056881 | 0.99999460 | -1.66824658 | 0.09973433 |
| SQR_BDI_T1 | 0.62791864 | 0.07607549 | 0.69548431 | 0.99999460 | 8.25388893 | 0.00000000 |

| **Analysis of Variance** | | | | | |
| --- | --- | --- | --- | --- | --- |
| **Source** | **Type III SS** | **df** | **Mean Squares** | **F-Ratio** | **p-Value** |
| Regression | 35.06235114 | 2 | 17.53117557 | 35.42306464 | 0.00000000 |
| Residual | 34.64359457 | 70 | 0.49490849 |  |  |

WARNING

| Case | 406.00000000 | is an Outlier | (Studentized Residual | : | 3.35156089) |
| --- | --- | --- | --- | --- | --- |

| Durbin-Watson D-Statistic | 1.73663883 |
| --- | --- |
| First Order Autocorrelation | 0.08862915 |

| **Information Criteria** | |
| --- | --- |
| AIC | 160.75472453 |
| AIC (Corrected) | 161.34295982 |
| Schwarz's BIC | 169.91656229 |

> *categ treat*

> *glm*

> *model agkon2 = constant treat agkon1*

> *estimate*

[▼General Linear Model](file:///\\Untitled.syo)

Effects coding used for categorical variables in model.

The categorical values encountered during processing are

| **Variables** | **Levels** | |
| --- | --- | --- |
| TREAT (2 levels) | CG | FB/FB+CET |

| Dependent Variable | AGKON2 |
| --- | --- |
| N | 74 |
| Multiple R | 0.35115285 |
| Squared Multiple R | 0.12330833 |

| **Estimates of Effects B = (X'X)^-1^X'Y** | | |
| --- | --- | --- |
| **Factor** | **Level** | **AGKON2** |
| CONSTANT |  | 0.43793041 |
| TREAT | CG | -0.15103299 |
| AGKON1 |  | 0.31268848 |

| **Analysis of Variance** | | | | | |
| --- | --- | --- | --- | --- | --- |
| **Source** | **Type III SS** | **df** | **Mean Squares** | **F-Ratio** | **p-Value** |
| TREAT | 1.56356716 | 1 | 1.56356716 | 3.20905290 | 0.07749349 |
| AGKON1 | 3.42355695 | 1 | 3.42355695 | 7.02648125 | 0.00989607 |
| Error | 34.59377954 | 71 | 0.48723633 |  |  |

| Durbin-Watson D-Statistic | 1.74826567 |
| --- | --- |
| First Order Autocorrelation | 0.11565751 |

| **Information Criteria** | |
| --- | --- |
| AIC | 161.73395342 |
| AIC (Corrected) | 162.31366356 |
| Schwarz's BIC | 170.95021379 |

> *calc 3.423557/(3.423557 +34.593780)*

[▼CALCULATE](file:///\\Untitled.syo)

0.09005252

> *categ treat*

> *select*

> *glm*

> *model inkon_ind2 = constant treat inkon_ind1*

> *estimate*

[▼General Linear Model](file:///\\Untitled.syo)

Effects coding used for categorical variables in model.

The categorical values encountered during processing are

| **Variables** | **Levels** | |
| --- | --- | --- |
| TREAT (2 levels) | CG | FB/FB+CET |

| Dependent Variable | INKON_IND2 |
| --- | --- |
| N | 74 |
| Multiple R | 0.31122518 |
| Squared Multiple R | 0.09686111 |

| **Estimates of Effects B = (X'X)^-1^X'Y** | | |
| --- | --- | --- |
| **Factor** | **Level** | **INKON_IND2** |
| CONSTANT |  | 0.24063339 |
| TREAT | CG | 0.09360361 |
| INKON_IND1 |  | 0.31343544 |

| **Analysis of Variance** | | | | | |
| --- | --- | --- | --- | --- | --- |
| **Source** | **Type III SS** | **df** | **Mean Squares** | **F-Ratio** | **p-Value** |
| TREAT | 0.59634820 | 1 | 0.59634820 | 2.50256051 | 0.11810614 |
| INKON_IND1 | 1.36279142 | 1 | 1.36279142 | 5.71892056 | 0.01943533 |
| Error | 16.91896049 | 71 | 0.23829522 |  |  |

| Durbin-Watson D-Statistic | 2.33135602 |
| --- | --- |
| First Order Autocorrelation | -0.16721543 |

| **Information Criteria** | |
| --- | --- |
| AIC | 108.80626975 |
| AIC (Corrected) | 109.38597989 |
| Schwarz's BIC | 118.02253012 |

> *calc 1.362791/(1.362791+16.918960)*

[▼CALCULATE](file:///\\Untitled.syo)

0.07454379

> *setcor*

> *model sqr_bdi_t2 | sqr_bdi_t1 = inkon_ind2 | inkon_ind1*

> *estimate*

[▼Set and Canonical Correlations](file:///\\Untitled.syo)

The categorical values encountered during processing are

| **Variables** | **Levels** | |
| --- | --- | --- |
| TREAT (2 levels) | CG | FB/FB+CET |

1 Cases deleted due to missing data.

**Bipartial Set Correlation Analysis (Y|YPARTIAL vs. X|XPARTIAL)**

Number of Cases on which Analysis is based: 73

Dependent Set y Partialled by these Variables

SQR_BDI_T1

Independent Set x Partialled by these Variables

INKON_IND1

| RAO F | : | 3.55939995 |  |  |
| --- | --- | --- | --- | --- |
| df | : | 1.00000000 | , | 70.00000000 |
| p-Value | : | 0.06335701 |  |  |

| R-square | : | 0.04802250 | Shrunk R-square | : | 0.03442282 |
| --- | --- | --- | --- | --- | --- |
| T-square | : | 0.04802250 | Shrunk T-square | : | 0.03442282 |
| P-square | : | 0.04802250 | Shrunk P-square | : | 0.03442282 |

| **Within Basic Set y Correlations** | |
| --- | --- |
|  | **SQR_BDI_T2** |
| SQR_BDI_T2 | 1.00000000 |

| **Within Basic Set x Correlations** | |
| --- | --- |
|  | **INKON_IND2** |
| INKON_IND2 | 1.00000000 |

| **Between Basic y (col) and Basic x (row) Correlations** | |
| --- | --- |
|  | **SQR_BDI_T2** |
| INKON_IND2 | 0.21914037 |

| **Estimated (from x-set) y Intercorrelations (R-square on diagonal)** | |
| --- | --- |
|  | **SQR_BDI_T2** |
| SQR_BDI_T2 | 0.04802250 |

| **Significance Tests for Prediction of Each Basic y Variable** | | |
| --- | --- | --- |
| **Variable** | **F-Ratio** | **p-Value** |
| SQR_BDI_T2 | 3.55939995 | 0.06335701 |

| **Betas Predicting Basic y (col) from Basic x (row) Variables** | |
| --- | --- |
|  | **SQR_BDI_T2** |
| INKON_IND2 | 0.21914037 |

| **Standard Error of Betas** | |
| --- | --- |
|  | **SQR_BDI_T2** |
| INKON_IND2 | 0.11615395 |

| **t-Statistic for Betas** | |
| --- | --- |
|  | **SQR_BDI_T2** |
| INKON_IND2 | 1.88663721 |

| **p-Value for Betas** | |
| --- | --- |
|  | **SQR_BDI_T2** |
| INKON_IND2 | 0.06335701 |

> *select*

> *categ /off*

> *glm*

> *model sqr_bdi_t2 = constant sqr_bdi_t1 agkon1 agkon2 treat*

> *estimate*

[▼General Linear Model](file:///\\Untitled.syo)

1 case(s) are deleted due to missing data.

| Dependent Variable | SQR_BDI_T2 |
| --- | --- |
| N | 73 |
| Multiple R | 0.73103451 |
| Squared Multiple R | 0.53441145 |
| Adjusted Squared Multiple R | 0.50702389 |
| Standard Error of Estimate | 0.69084656 |

| **Regression Coefficients B = (X'X)^-1^X'Y** | | | | | | |
| --- | --- | --- | --- | --- | --- | --- |
| **Effect** | **Coefficient** | **Standard Error** | **Std. Coefficient** | **Tolerance** | **t** | **p-Value** |
| CONSTANT | 1.12125590 | 0.30357347 | 0.00000000 | . | 3.69352396 | 0.00044258 |
| SQR_BDI_T1 | 0.62549956 | 0.07885451 | 0.69280493 | 0.89757846 | 7.93232485 | 0.00000000 |
| AGKON1 | 0.12551350 | 0.12738933 | 0.08888622 | 0.84127749 | 0.98527479 | 0.32798194 |
| AGKON2 | -0.25118357 | 0.12001710 | -0.18485640 | 0.87764848 | -2.09289826 | 0.04009188 |
| TREAT | -0.21252073 | 0.17083803 | -0.10499431 | 0.96115926 | -1.24398957 | 0.21777451 |

| **Analysis of Variance** | | | | | |
| --- | --- | --- | --- | --- | --- |
| **Source** | **Type III SS** | **df** | **Mean Squares** | **F-Ratio** | **p-Value** |
| Regression | 37.25165580 | 4 | 9.31291395 | 19.51292572 | 0.00000000 |
| Residual | 32.45428992 | 68 | 0.47726897 |  |  |

| Durbin-Watson D-Statistic | 1.72296767 |
| --- | --- |
| First Order Autocorrelation | 0.10205878 |

| **Information Criteria** | |
| --- | --- |
| AIC | 159.98926903 |
| AIC (Corrected) | 161.26199630 |
| Schwarz's BIC | 173.73202568 |

> *!! Sobel test from http://quantpsy.org/sobel/sobel.htm*

> *!! Sobel test statistic: -1.36, SE = 0.056, p = .17*

> *!!*

> *!!*

> *!!*

> *!! Additional analyses*

> *!!*

> *!!*

> *!!*

> *categ condition*

> *glm*

> *model cpt1 cpt2 cat1 cat2 cit1 cit2 = constant condition /repeat={3,2} names={domain, time}*

> *estimate*

[▼General Linear Model](file:///\\Untitled.syo)

Effects coding used for categorical variables in model.

The categorical values encountered during processing are

| **Variables** | **Levels** | | |
| --- | --- | --- | --- |
| CONDITION (3 levels) | Control | Feedback | Feedback + CET |

N of Cases Processed: 74

| **Dependent Variable Means** | | | | | |
| --- | --- | --- | --- | --- | --- |
| **CPT1** | **CPT2** | **CAT1** | **CAT2** | **CIT1** | **CIT2** |
| 10.02356219 | 9.25674348 | 8.28784544 | 8.89398305 | 10.87390467 | 10.42541606 |

| **Repeated Measures Factors and Levels of Dependent Variables** | | | | | | |
| --- | --- | --- | --- | --- | --- | --- |
| **Within Factor** | **1** | **2** | **3** | **4** | **5** | **6** |
| DOMAIN | 1.00000000 | 1.00000000 | 2.00000000 | 2.00000000 | 3.00000000 | 3.00000000 |
| TIME | 1.00000000 | 2.00000000 | 1.00000000 | 2.00000000 | 1.00000000 | 2.00000000 |

**Univariate and Multivariate Repeated Measures Analysis**

| **Between Subjects** | | | | | |
| --- | --- | --- | --- | --- | --- |
| **Source** | **SS** | **df** | **Mean Squares** | **F-Ratio** | **p-Value** |
| CONDITION | 8.31886132 | 2 | 4.15943066 | 0.13777095 | 0.87153060 |
| Error | 2.14355481E+003 | 71 | 30.19091276 |  |  |

| **Within Subjects** | | | | | | | |
| --- | --- | --- | --- | --- | --- | --- | --- |
| **Source** | **SS** | **df** | **Mean Squares** | **F-Ratio** | **p-Value** | **G-G** | **H-F** |
| DOMAIN | 298.49101354 | 2 | 149.24550677 | 7.43863678 | 0.00084666 | 0.00193700 | 0.00161852 |
| DOMAIN*CONDITION | 125.15685412 | 4 | 31.28921353 | 1.55950487 | 0.18839501 | 0.19959137 | 0.19715700 |
| Error | 2.84902497E+003 | 142 | 20.06355615 |  |  |  |  |

| Greenhouse-Geisser Epsilon | 0.81522874 |
| --- | --- |
| Huynh-Feldt Epsilon | 0.85523019 |

| **Within Subjects** | | | | | | | |
| --- | --- | --- | --- | --- | --- | --- | --- |
| **Source** | **SS** | **df** | **Mean Squares** | **F-Ratio** | **p-Value** | **G-G** | **H-F** |
| TIME | 3.57047259 | 1 | 3.57047259 | 0.38191128 | 0.53855945 | . | . |
| TIME*CONDITION | 12.11558570 | 2 | 6.05779285 | 0.64796448 | 0.52617471 | . | . |
| Error | 663.77603347 | 71 | 9.34895822 |  |  |  |  |

| Greenhouse-Geisser Epsilon | . |
| --- | --- |
| Huynh-Feldt Epsilon | . |

| **Within Subjects** | | | | | | | |
| --- | --- | --- | --- | --- | --- | --- | --- |
| **Source** | **SS** | **df** | **Mean Squares** | **F-Ratio** | **p-Value** | **G-G** | **H-F** |
| DOMAIN*TIME | 37.78178513 | 2 | 18.89089256 | 1.36972985 | 0.25751303 | 0.25751533 | 0.25751303 |
| DOMAIN*TIME*CONDITION | 41.59033252 | 4 | 10.39758313 | 0.75390191 | 0.55699952 | 0.55581323 | 0.55699952 |
| Error | 1.95842031E+003 | 142 | 13.79169229 |  |  |  |  |

| Greenhouse-Geisser Epsilon | 0.99024459 |
| --- | --- |
| Huynh-Feldt Epsilon | 1.00000000 |

**Multivariate Repeated Measures Analysis**

| **Test of: DOMAIN** | | | | | |
| --- | --- | --- | --- | --- | --- |
| **Statistic** | **Value** | **Hypothesis df** | **Error df** | **F-Ratio** | **p-Value** |
| Wilks's Lambda | 0.73058153 | 2 | 70 | 12.90704221 | 0.00001692 |
| Pillai Trace | 0.26941847 | 2 | 70 | 12.90704221 | 0.00001692 |
| Hotelling-Lawley Trace | 0.36877263 | 2 | 70 | 12.90704221 | 0.00001692 |

| **Test of: DOMAIN*CONDITION** | | | | | |
| --- | --- | --- | --- | --- | --- |
| **Statistic** | **Value** | **Hypothesis df** | **Error df** | **F-Ratio** | **p-Value** |
| Wilks's Lambda | 0.92303094 | 4 | 140 | 1.43006235 | 0.22717019 |
| Pillai Trace | 0.07823902 | 4 | 142 | 1.44528134 | 0.22219199 |
| Hotelling-Lawley Trace | 0.08201144 | 4 | 138 | 1.41469729 | 0.23229391 |

| **THETA** | **S** | **M** | **N** | **p-Value** |
| --- | --- | --- | --- | --- |
| 0.05525556 | 2 | -0.50000000 | 34.00000000 | 0.33833737 |

| **Test of: DOMAIN*TIME** | | | | | |
| --- | --- | --- | --- | --- | --- |
| **Statistic** | **Value** | **Hypothesis df** | **Error df** | **F-Ratio** | **p-Value** |
| Wilks's Lambda | 0.95901675 | 2 | 70 | 1.49571299 | 0.23116214 |
| Pillai Trace | 0.04098325 | 2 | 70 | 1.49571299 | 0.23116214 |
| Hotelling-Lawley Trace | 0.04273466 | 2 | 70 | 1.49571299 | 0.23116214 |

| **Test of: DOMAIN*TIME*CONDITION** | | | | | |
| --- | --- | --- | --- | --- | --- |
| **Statistic** | **Value** | **Hypothesis df** | **Error df** | **F-Ratio** | **p-Value** |
| Wilks's Lambda | 0.95793674 | 4 | 140 | 0.76017447 | 0.55289758 |
| Pillai Trace | 0.04213061 | 4 | 142 | 0.76391025 | 0.55042174 |
| Hotelling-Lawley Trace | 0.04383996 | 4 | 138 | 0.75623937 | 0.55551088 |

| **THETA** | **S** | **M** | **N** | **p-Value** |
| --- | --- | --- | --- | --- |
| 0.04046633 | 2 | -0.50000000 | 34.00000000 | 0.49867145 |

> *glm*

> *model PGQ_ach_C1 PGQ_AFF_C1 PGQ_IND_C1 PGQ_ach_C2 PGQ_AFF_C2 PGQ_IND_C2 = constant condition /repeat={2,3} names={time, domain}*

> *estimate*

[▼General Linear Model](file:///\\Untitled.syo)

Effects coding used for categorical variables in model.

The categorical values encountered during processing are

| **Variables** | **Levels** | | |
| --- | --- | --- | --- |
| CONDITION (3 levels) | Control | Feedback | Feedback + CET |

N of Cases Processed: 74

| **Dependent Variable Means** | | | | | |
| --- | --- | --- | --- | --- | --- |
| **PGQ_ACH_C1** | **PGQ_AFF_C1** | **PGQ_IND_C1** | **PGQ_ACH_C2** | **PGQ_AFF_C2** | **PGQ_IND_C2** |
| 4.20608108 | 3.93243243 | 4.06756757 | 4.21959459 | 4.03378378 | 3.96959459 |

| **Repeated Measures Factors and Levels of Dependent Variables** | | | | | | |
| --- | --- | --- | --- | --- | --- | --- |
| **Within Factor** | **1** | **2** | **3** | **4** | **5** | **6** |
| TIME | 1.00000000 | 1.00000000 | 1.00000000 | 2.00000000 | 2.00000000 | 2.00000000 |
| DOMAIN | 1.00000000 | 2.00000000 | 3.00000000 | 1.00000000 | 2.00000000 | 3.00000000 |

**Univariate and Multivariate Repeated Measures Analysis**

| **Between Subjects** | | | | | |
| --- | --- | --- | --- | --- | --- |
| **Source** | **SS** | **df** | **Mean Squares** | **F-Ratio** | **p-Value** |
| CONDITION | 2.00864270 | 2 | 1.00432135 | 0.65305444 | 0.52355126 |
| Error | 109.18969627 | 71 | 1.53788305 |  |  |

| **Within Subjects** | | | | | | | |
| --- | --- | --- | --- | --- | --- | --- | --- |
| **Source** | **SS** | **df** | **Mean Squares** | **F-Ratio** | **p-Value** | **G-G** | **H-F** |
| TIME | 0.01502991 | 1 | 0.01502991 | 0.07203806 | 0.78917046 | . | . |
| TIME*CONDITION | 0.56857050 | 2 | 0.28428525 | 1.36257390 | 0.26261271 | . | . |
| Error | 14.81332702 | 71 | 0.20863841 |  |  |  |  |

| Greenhouse-Geisser Epsilon | . |
| --- | --- |
| Huynh-Feldt Epsilon | . |

| **Within Subjects** | | | | | | | |
| --- | --- | --- | --- | --- | --- | --- | --- |
| **Source** | **SS** | **df** | **Mean Squares** | **F-Ratio** | **p-Value** | **G-G** | **H-F** |
| DOMAIN | 4.66106246 | 2 | 2.33053123 | 5.00055780 | 0.00796804 | 0.00864156 | 0.00796804 |
| DOMAIN*CONDITION | 0.91798746 | 4 | 0.22949686 | 0.49242521 | 0.74129954 | 0.73535554 | 0.74129954 |
| Error | 66.17970398 | 142 | 0.46605425 |  |  |  |  |

| Greenhouse-Geisser Epsilon | 0.96804525 |
| --- | --- |
| Huynh-Feldt Epsilon | 1.00000000 |

| **Within Subjects** | | | | | | | |
| --- | --- | --- | --- | --- | --- | --- | --- |
| **Source** | **SS** | **df** | **Mean Squares** | **F-Ratio** | **p-Value** | **G-G** | **H-F** |
| TIME*DOMAIN | 0.69875125 | 2 | 0.34937562 | 1.33338967 | 0.26686199 | 0.26601185 | 0.26647397 |
| TIME*DOMAIN*CONDITION | 0.76294431 | 4 | 0.19073608 | 0.72794293 | 0.57426995 | 0.55943590 | 0.56603849 |
| Error | 37.20693182 | 142 | 0.26202065 |  |  |  |  |

| Greenhouse-Geisser Epsilon | 0.89093912 |
| --- | --- |
| Huynh-Feldt Epsilon | 0.93804184 |

**Multivariate Repeated Measures Analysis**

| **Test of: DOMAIN** | | | | | |
| --- | --- | --- | --- | --- | --- |
| **Statistic** | **Value** | **Hypothesis df** | **Error df** | **F-Ratio** | **p-Value** |
| Wilks's Lambda | 0.86184291 | 2 | 70 | 5.61064892 | 0.00549521 |
| Pillai Trace | 0.13815709 | 2 | 70 | 5.61064892 | 0.00549521 |
| Hotelling-Lawley Trace | 0.16030425 | 2 | 70 | 5.61064892 | 0.00549521 |

| **Test of: DOMAIN*CONDITION** | | | | | |
| --- | --- | --- | --- | --- | --- |
| **Statistic** | **Value** | **Hypothesis df** | **Error df** | **F-Ratio** | **p-Value** |
| Wilks's Lambda | 0.97225367 | 4 | 140 | 0.49590464 | 0.73875704 |
| Pillai Trace | 0.02792520 | 4 | 142 | 0.50269111 | 0.73379879 |
| Hotelling-Lawley Trace | 0.02835419 | 4 | 138 | 0.48910978 | 0.74372084 |

| **THETA** | **S** | **M** | **N** | **p-Value** |
| --- | --- | --- | --- | --- |
| 0.01797369 | 2 | -0.50000000 | 34.00000000 | 0.74780419 |

| **Test of: TIME*DOMAIN** | | | | | |
| --- | --- | --- | --- | --- | --- |
| **Statistic** | **Value** | **Hypothesis df** | **Error df** | **F-Ratio** | **p-Value** |
| Wilks's Lambda | 0.97252175 | 2 | 70 | 0.98891242 | 0.37711713 |
| Pillai Trace | 0.02747825 | 2 | 70 | 0.98891242 | 0.37711713 |
| Hotelling-Lawley Trace | 0.02825464 | 2 | 70 | 0.98891242 | 0.37711713 |

| **Test of: TIME*DOMAIN*CONDITION** | | | | | |
| --- | --- | --- | --- | --- | --- |
| **Statistic** | **Value** | **Hypothesis df** | **Error df** | **F-Ratio** | **p-Value** |
| Wilks's Lambda | 0.96841791 | 4 | 140 | 0.56613225 | 0.68762704 |
| Pillai Trace | 0.03167858 | 4 | 142 | 0.57134443 | 0.68385292 |
| Hotelling-Lawley Trace | 0.03251242 | 4 | 138 | 0.56083929 | 0.69146396 |

| **THETA** | **S** | **M** | **N** | **p-Value** |
| --- | --- | --- | --- | --- |
| 0.02826509 | 2 | -0.50000000 | 34.00000000 | 0.64857225 |
