# Supplementary material for: Enhancing Congruence between Implicit Motives and Explicit Goal Commitments: Results of a Randomized Controlled Trial
Source: Front Psychol. 2017 Sep 12;8:1540. doi: 10.3389/fpsyg.2017.01540 (PMC5600949; doi:10.3389/fpsyg.2017.01540)
Supplement: Supplementary file 1 [file Presentation1.ZIP › Roch et al CET training materials.doc]

Optimizing the fit between motives and goals

# Training materials for

The following pages will provide you with exercises aimed at helping you become more aware of your motives and goals. Please read the instructions carefully and do not hesitate to ask questions if you have difficulty understanding what this is about. Please note that this is not a test, just an opportunity for you to achieve a better fit between your motives and goals. Depending on your motivational profile, you can work on up to four different exercises.

The objective of the first part of this training package is to help you become aware of the emotional power of your motives and how they affect your life in unique ways.


# *Peak experiences*

Many people recall peak experiences from their lives.

A peak experience is a situation in which you may have experienced feelings of wholeness, perfection, richness, vitality, beauty, uniqueness or insight.

In general, peak experiences can vary widely. For instance, a heart-to-heart talk with a friend can be a peak experience for one person, whereas for another it is the competition for first place in a contest or self-absorbed work on a painting or sculpture.

**On the next three pages, please try to remember and write down such peak experiences that you remember from your life. Please try to list one unforgettable peak experience for each of the three motivational domains achievement, affiliation, and power and describe it in a couple of sentences.**

When describing each peak experience, please try to address the following questions:

-	What exactly happened?

-	Where did it happen?

-	Who was involved?

-	What did you do and what did you think and feel in this situation?

-	What kind of impact did the experience have on you?

# Peak experience in the domain of achievement

Please consider these questions:

- What exactly happened?
- Where did it happen?
- Who was involved?
- What did you do and what did you think and feel in this situation?
- What kind of impact did the experience have on you?

# Peak experience in the domain of affiliation


Please consider these questions:

- What exactly happened?
- Where did it happen?
- Who was involved?
- What did you do and what did you think and feel in this situation?
- What kind of impact did the experience have on you?

# Peak experience in the domain of power and influence

Please consider these questions:

- What exactly happened?
- Where did it happen?
- Who was involved?
- What did you do and what did you think and feel in this situation?
- What kind of impact did the experience have on you?

Before we move on to the **second part of the training**, in which you will work on your motivational profile, you need to determine if and where your profile features discrepancies and congruence and what specific shape they have. Please copy your personal motivational profile into the empty table below.

Please write down what kind of a discrepancy or congruence you observe in each of the three domains achievement (Ach), affiliation (Aff), and power (Pow).

Discrepancy in the domain:	____________________________

____________________________

____________________________

Congruence in the domain:	____________________________

____________________________

____________________________

First you will work on the discrepancies. If your profile features no discrepancies, you can skip this exercise and continue with the next exercise on p. 12.

In the following, your task is to determine **what kind of discrepancy** (or discrepancies) you have.

**Weak motive, strong goal commitment in…:**	____________________

____________________

____________________

In this domain/these domains you can identify ***opportunities for change!***

**Strong motive, weak goal commitment in…:**	____________________

____________________

____________________

In this domain/these domains you can ***discover potentials!***

If you have identified a discrepancy of the type „**opportunity for change**“, please complete the exercise on p. 8. If you have identified a discrepancy of the type „**discover potentials**“, please complete the exercise on p. 10. If you have identifies both types of discrepancies, please work through both exercises consecutively.

***Opportunities for change***

You have just learned about various strategies for compensating motivational deficits by taking one’s motives and peak experiences into account.

**Now you can apply these strategies (search for additional incentives, specific aspects of peak experiences) to your own motivational profile.**

**Please describe, for each motivational domain that features this type of discrepancy, what you would like to do to compensate for the motivational deficit. Please try to think of ways in which you can tap a stronger motive. Or focus on aspects of peak experiences in this domain that can help you enrich the goal with emotionally engaging aspects and incentives.**

The next page provides you with space for writing down your thoughts and solutions.

# Exercise for *opportunities for change*

Please complete this exercise only for those domains that feature a discrepancy of the type weak motive/strong goal commitment.

Achievement:

Affiliation:

Power:

# *Discovering potentials*

We covered the issue of learning about one’s own potentials. Based onthe peak experiences you wrote down in a previous exercise, you already cam in touch with the emotions that accompany the satisfaction of a motive.

**Now your task is to recall your peak experiences and write down in a couple of sentences how you can transfer features of those situations to other situations, particularly those associated with your goal pursuits. Try to use your understanding of your peak experiences to enrich your goal in the same corresponding with enticing aspects and incentives from the peak experience situation. Try to make your goal more concrete and attractive in this manner.**

The next page provides you with space for writing down your thoughts and solutions.

# Exercise for *discovering potentials*

Please complete this exercise only for those domains that feature a discrepancy of the type strong motive/weak goal commitment.

Achievement:

Affiliation:

Power:

Now you will work on case of congruence. If your profile features no such cases, you can skip this exercise and go to p. 15.

In the following, your task is to determine **what kind of high/high congruence** (or low/low congruence) you have.

**Strong motive, strong goal commitment in…:**	____________________

____________________

____________________

In this domain/these domains you can try to ***maximize your potential!***

**Weak motive, weak goal commitment in…:**	____________________

____________________

____________________

This type of low/low congruence currently has little motivational relevance for you. However, this may change if you ever strongly commit to a goal in this domain.

If you have identified a case of high/high congruence of the type „**maximizing potentials**“, please complete the exercise on p. 13. For cases of low/low congruence (weak motive, weak goal commitment) you do not need to complete an exercise.

# *Maximizing potentials*

You have a good fit between a motive and a goal commitment in one or more domains. This provides you with an excellent starting point for pursuing and realizing the respective goal(s).

To maximize this potential, you will have an opportunity in the following to fully understand the specialness of this fit. Please try to think of two or three aspects that will make it particularly likely that you will attain your goal(s). Please focus on the actions and activities that are particularly likely to connect your motive(s) with your goal(s) and the emotions and feelings that will result from this.

The next page provides you with space for writing down your thoughts and observations.

# Exercise for *maximizing potentials*

Please complete this exercise only for those domains that feature a congruence of the type strong motive/strong goal commitment.

Achievement:

Affiliation:

Power:

# Now you have finished the training and completed all exercises.

Please return this package to the trainer. Thank you for your participation and best of success for realizing your goals!

**After the study is completed (on the last day of the study) your package will be returned to you for future use and reference.**
