# Supplementary material for: Enhancing Congruence between Implicit Motives and Explicit Goal Commitments: Results of a Randomized Controlled Trial
Source: Front Psychol. 2017 Sep 12;8:1540. doi: 10.3389/fpsyg.2017.01540 (PMC5600949; doi:10.3389/fpsyg.2017.01540)
Supplement: Supplementary file 1 [file Presentation1.ZIP › Roch et al peak experiences exercise.doc]

Raising awareness of one’s motives

# Training materials for

The following pages will provide you with an exercise aimed at helping you become more aware of your motives. Please read the instructions carefully and do not hesitate to ask questions if you have difficulty understanding what this is about. Please note that this is not a test, just an opportunity for you to become acquainted with your motives.

The goal of this exercise is to help you experience the emotional power of motives and how it affects your life in unique ways. Because motives are closely related to peak experiences, we will focus on the latter in the following exercise.


# *Peak experiences*

Many people recall peak experiences from their lives.

A peak experience is a situation in which you may have experienced feelings of wholeness, perfection, richness, vitality, beauty, uniqueness or insight.

In general, peak experiences can vary widely. For instance, a heart-to-heart talk with a friend can be a peak experience for one person, whereas for another it is the competition for first place in a contest or self-absorbed work on a painting or sculpture.

**On the next three pages, please try to remember and write down such peak experiences that you remember from your life. Please try to list one unforgettable peak experience for each of the three motivational domains achievement, affiliation, and power and describe it in a couple of sentences.**

When describing each peak experience, please try to address the following questions:

-	What exactly happened?

-	Where did it happen?

-	Who was involved?

-	What did you do and what did you think and feel in this situation?

-	What kind of impact did the experience have on you?

# Peak experience in the domain of achievement

Please consider these questions:

- What exactly happened?
- Where did it happen?
- Who was involved?
- What did you do and what did you think and feel in this situation?
- What kind of impact did the experience have on you?

# Peak experience in the domain of affiliation


Please consider these questions:

- What exactly happened?
- Where did it happen?
- Who was involved?
- What did you do and what did you think and feel in this situation?
- What kind of impact did the experience have on you?

# Peak experience in the domain of power and influence

Please consider these questions:

- What exactly happened?
- Where did it happen?
- Who was involved?
- What did you do and what did you think and feel in this situation?
- What kind of impact did the experience have on you?

You have finished this set of exercise materials. Please return this pachage to the trainer. Thank you for your participation and best of success for realizing your goals!

**After the study is completed (on the last day of the study) your package will be returned to you for future use and reference.**
