# Supplementary material for: Enhancing Congruence between Implicit Motives and Explicit Goal Commitments: Results of a Randomized Controlled Trial
Source: Front Psychol. 2017 Sep 12;8:1540. doi: 10.3389/fpsyg.2017.01540 (PMC5600949; doi:10.3389/fpsyg.2017.01540)
Supplement: Supplementary file 1 [file Presentation1.ZIP › Roch et al sample feedback sheet.doc]

# Individual feedback about the motivational structure of John Doe

**Achievement: „Dissertation“**

There is a discrepancy resulting from an important personal goal and a weak implicit motive in the achievement domain. Here, you may be able to discover an opportunity for change.

**Affiliation: „Taking care of friendships“**

This is a case of positive congruence. This means that in the domain of affiliation, you have an important personal goal that is matched by a strong implicit motive. This may provide an opportunity to maximize a potential.

**Power: “Office in volunteer organization”**

There is a discrepancy resulting from an unimportant personal goal and a strong implicit motive in the power domain. This may provide an opportunity to discover a potential.

The next pages provide additional information about motives and goals.

# Motives and goals

Motives (implicit motives) and goals (explicit motives) have in common that they both activate behavior, but in different way. Motives are characterized by the pleasure and satisfaction associated with getting a motive-related reward. In contrast, goals are directed towards future states and provide us with a sense of meaning and purpose in life. Motives and goals can be divided into the domains achievement, affiliation, and power.

# The structure of motivation

Often, there is a difference in strength between motives and goals within a given domain. We distinguish between two types of discrepancies:

One discrepancy results from having a weak motive in a given domain, but pursuing a goal (or goals) in the same domain that one feels strongly committed to. What is missing, at a gut-level but not necessarily at a conscious level, is the expectation of pleasure that automatically energizes behavior aimed at realizing the goal. But this deficit can be compensated by suitable strategies.

The other discrepancy results from having a strong motive in a domain in which one feels no or only a weak sense of commitment to pursue a matching goal. In this case, although one has the motivational potential to realize a goal, one may take the goal seriously enough and may not end up doing it. But here, too, there are ways to counteract this discrepancy.

Of course, there can also be congruence between motives and goals. Again, there are two ways in which this can happen:

In the case of positive congruence in a motivational domain, there is a strong motive and one also feels strongly committed to a corresponding goal. This represents an optimal fit, because it is easy to succeed in realizing the goal one pursues.

In the case of negative congruence in a motivational domain, there is a weak motive and one also feels only weakly committed to a corresponding goal. However, one should keep in mind that perhaps one day one will have a strong commitment to a goal in this domain and will need to rely on strategies for dealing with its weak motivational foundation.

# The „Big Three“ of motivation

## The achievement motive

The achievement motive is based on a capacity to expect pleasure from the successful and self-directed mastery of challenging tasks. The achievement motive can be activated by environmental cues predicting such mastery-pleasure experiences, such as an encounter with an unexpected challenge.

People with a strong achievement motive respond to challenging tasks in a unique way: instead of shying away from the effort that needs to be invested into mastering it, they dive right in, because they expect to eventually succeed. But they also insist on doing it on their own terms and shun advice and instructions from others. And they frequently gather feedback about how well they are doing now relative to how well they were doing before to gauge whether they are improving. In work-related contexts, they can be very self-propelled and attracted to challenging problems.

People with a weak achievement motive, on the other hand, do not like challenging tasks, because success is neither certain nor quick. In general they prefer either very easy tasks that promise quick success or very difficult tasks, which allow them to blame high task difficulty, and not themselves, for failure. In work contexts, people with a weak achievement motive can do well when they are clearly told what is expected of them, and social comparison allows them to gauge their own performance. But they also experience competitive situations as anxiety-inducing and try to avoid them.

## The affiliation motive

The affiliation motive is based on the capacity for drawing pleasure from establishing, maintaining, or restoring positive relationships with other people.The affiliation motive is engaged by environmental cues signaling opportunities for such positive interactions, like others’ smiles or positive attention.

People with a strong affiliation motive are drawn to others who signal openness and acceptance to them. They are also able to tolerate unfriendliness up to a certain extent, as long as they can hope for an eventual improvement of the relationship. Affiliation-motivated individuals are happy in the company of friends and close others and also thrive in collegial work contexts.

People with a weak affiliation motive are not particularly sensitive to others’ signals of liking and friendship, and neither are they keen on signaling to others that they like them. In general, they tend to be more socially independent, because they do not get much pleasure from being close to others.

## The power motive

The power motive determines the degree to which a person can find pleasure in having physical, mental, or emotional impact on others, including groups of people and to what extent the person experiences others‘ impact on herself or himself as aversive. Typical triggers that set the power motive in motion are cues related to reputation, social influence, and being able to alter others’ emotions.

People with a strong power motive are quick to learn behaviors that allow them to experience pleasurable feelings of influence on others, and these behaviors can be subtle. For instance, power-motivated people are known to speak fluently, gesticulate and be facially expressive to convey an image of competence and thus to convince others. But they can also be funny to make others laugh. They reap emotional well-being from such impact episodes. They are also more likely to end up in high-level management positions where they have ample opportunity to influence others much of the time, but without being exposed to others’ influence. On the downside, they can be assertive in an aggressive way and manipulate others.

People with a weak power motive shy away from positions of power and influence, which make them uncomfortable, and are also less adept at reading others’ emotional state quickly. As a consequence, they are less likely to occupy positions where they can wield influence on others, and when they do, they are relatively ineffective. But they are also less intent on having their way with others and can therefore be more relaxed with others.
